# Comparative Genomics of *Wolbachia*–*Cardinium* Dual Endosymbiosis in a Plant-Parasitic Nematode

**DOI:** 10.3389/fmicb.2018.02482

**Published:** 2018-10-16

**Authors:** Amanda M. V. Brown, Sulochana K. Wasala, Dana K. Howe, Amy B. Peetz, Inga A. Zasada, Dee R. Denver

**Affiliations:** ^1^Department of Biological Sciences, Texas Tech University, Lubbock, TX, United States; ^2^Department of Integrative Biology, Oregon State University, Corvallis, OR, United States; ^3^USDA-ARS Horticultural Crops Research Laboratory, Corvallis, OR, United States

**Keywords:** *Cardinium*, *Wolbachia*, plant-parasitic nematode, *Pratylenchus penetrans*, horizontal gene transfer, symbiosis, endosymbiont, genomics

## Abstract

*Wolbachia* and *Cardinium* are among the most important and widespread of all endosymbionts, occurring in nematodes and more than half of insect and arachnid species, sometimes as coinfections. These symbionts are of significant interest as potential biocontrol agents due to their abilities to cause major effects on host biology and reproduction through cytoplasmic incompatibility, sex ratio distortion, or obligate mutualism. The ecological and metabolic effects of coinfections are not well understood. This study examined a *Wolbachia*–*Cardinium* coinfection in the plant-parasitic nematode (PPN), *Pratylenchus penetrans*, producing the first detailed study of such a coinfection using fluorescence *in situ* hybridization (FISH), polymerase chain reaction (PCR), and comparative genomic analysis. Results from FISH and single-nematode PCR showed 123/127 individuals in a focal population carried *Cardinium* (denoted strain cPpe), and 48% were coinfected with *Wolbachia* strain wPpe. Both endosymbionts showed dispersed tissue distribution with highest densities in the anterior intestinal walls and gonads. Phylogenomic analyses confirmed an early place of cPpe and long distance from a sister strain in another PPN, *Heterodera glycines*, supporting a long history of both *Cardinium* and *Wolbachia* in PPNs. The genome of cPpe was 1.36 Mbp with 35.8% GC content, 1,131 predicted genes, 41% having no known function, and missing biotin and lipoate synthetic capacity and a plasmid present in other strains, despite having a slightly larger genome compared to other sequenced *Cardinium*. The larger genome revealed expansions of gene families likely involved in host–cellular interactions. More than 2% of the genes of cPpe and wPpe were identified as candidate horizontally transferred genes, with some of these from eukaryotes, including nematodes. A model of the possible *Wolbachia*–*Cardinium* interaction is proposed with possible complementation in function for pathways such as methionine and fatty acid biosynthesis and biotin transport.

## Introduction

Coinfecting symbionts can interact ecologically and metabolically to impact their host’s health and survival. These impacts can range from parasitic or commensal to facultatively or obligately beneficial. Extensive studies of partner symbioses aided by complete genomic data have revealed intimate microbial interactions that are primary (obligate) supplementing host nutrition (Bennett and Moran, 2013; Koga and Moran, 2014; Mao et al., 2017; Meseguer et al., 2017; Sudakaran et al., 2017) or secondary (facultative) providing benefits under some circumstances (Burke et al., 2010; Oliver et al., 2010; Burke and Moran, 2011; De Clerck et al., 2014; Manzano-Marín and Latorre, 2014; Rao et al., 2015). Many of these well-studied dual symbioses occur in insects, in which specialized diets drive adaptations in hosts and their microbes. Belowground plant-feeding organisms can experience similar pressures due to plant-diet specialization. For example, the plant-parasitic nematode (PPN), *Xiphinema americanum*, has evolved one or more obligate endosymbionts with nutrient supplementation roles (Brown et al., 2015; Palomares-Rius et al., 2016), whereas other PPNs appear to have evolved microbial partners to degrade plant compounds (Cheng et al., 2013).

*Wolbachia pipientis* (α-proteobacteria) and *Cardinium hertigii* (Bacteroidetes) (hereafter denoted *Wolbachia* and *Cardinium*) are among the most widespread endosymbionts in animals, co-occurring extensively (Gotoh et al., 2007; Nakamura et al., 2009, 2012; White et al., 2011, 2009; Ros et al., 2012; Zhao et al., 2013; Mee et al., 2015; Zhang et al., 2016a; Nguyen et al., 2017; Zélé et al., 2018). Their effects on host biology make these endosymbionts a major focus in studies of biocontrol, especially for their independently acquired phenotype known as cytoplasmic incompatibility (CI), in which infected males mating with uninfected females fail to produce viable offspring, therein increasing the relative success of the maternally inherited endosymbionts. Yet, *Wolbachia* and *Cardinium* can have other phenotypes, including neutral and obligate or facultatively beneficial roles (Gill et al., 2014; Comandatore et al., 2015; Moriyama et al., 2015; An et al., 2017; Zélé et al., 2018); in the majority of hosts, their role is not known. *Cardinium* is generally less well characterized than *Wolbachia*. It was first described in parasitoid wasps (Zchori-Fein et al., 2004) and subsequent surveys showed it at high levels in spiders and mites (60% of species; Weinert et al., 2015) and planthoppers (47% of species; Nakamura et al., 2009), in which it had a positive association with *Wolbachia*. Only two full *Cardinium* genomes have been sequenced (Penz et al., 2012; Santos-Garcia et al., 2014) from a whitefly (*Bemisia* sp.) and its parasitoid wasp (*Encarsia* sp.), providing limited understanding of this group. Comparative genomic analyses with the closest available outgroup, an endosymbiont of *Acanthamoeba* spp., *Candidatus* Amoebophilus asiaticus (hereafter *Amoebophilus*) (Schmitz-Esser et al., 2010) leave many questions about the broader patterns in this widespread clade (Amoebophilaceae) (Santos-Garcia et al., 2014) unanswered, including: what is the genomic basis of shared phenotypes (e.g., CI), and is there potentially metabolic synergy between *Wolbachia* and *Cardinium* (Zhang et al., 2016b)?

This study focuses on a recently discovered coinfection of *Wolbachia* and *Cardinium* in a PPN (Denver et al., 2016). Nematodes are major contributors to terrestrial carbon flow and plant-parasitic species cost an estimated US$80 billion in global annual agricultural damage (Nicol et al., 2011). Despite this economic impact (Jones et al., 2013), PPN endosymbionts have received limited attention. While experimental work has shown that bacterial symbionts play essential roles in entomopathogenic and filarial nematodes (Chaston et al., 2011; Landmann et al., 2014), PPN endosymbionts have been difficult to study experimentally. Comparative genomics, polymerase chain reaction (PCR), fluorescence *in situ* hybridization (FISH), and transmission electron microscopy (TEM) have confirmed *Wolbachia* occur in at least three species of PPN: the burrowing nematodes *Radopholus similis* and *Radopholus arabocoffeae* (Haegeman et al., 2009) and the root lesion nematode *P. penetrans* (Brown et al., 2016; Denver et al., 2016). Results hinted at an intermediate role for this PPN *Wolbachia* (strain wPpe) between parasitic and beneficial (Brown et al., 2016). TEM and light microscopy have described a *Cardinium*-like endosymbiont in four species of PPN, the potato cyst nematode *Globodera rostochiensis* (Shepherd et al., 1973; Walsh et al., 1983a,b), the cereal cyst nematode *Heterodera avenae* (Yang et al., 2017), the pea cyst nematode *Heterodera goettingiana* (Shepherd et al., 1973), and the soybean cyst nematode *Heterodera glycines* (Endo, 1979; Noel and Atibalentja, 2006), with the present study adding a fifth record in *P. penetrans* (Denver et al., 2016). The *Cardinium* strain in *H. glycines* was originally named *Candidatus* Paenicardinium endonii (Noel and Atibalentja, 2006) and subsequently re-assigned to *Candidatus C. hertigii* (Nakamura et al., 2009). Prior to the present study, genomic and FISH analyses were unavailable. Early work suggested the *H. glycines Cardinium* strain might confer a benefit to its host based on occurrence at 100% prevalence, lack of pathological signs, and close association with ribosomes and host nuclei in the esophageal glands (Shepherd et al., 1973; Endo, 1979; Walsh et al., 1983a,b; Noel and Atibalentja, 2006). Occurrence in the esophageal glands has significance because of the mode of infection in PPNs by which they secrete plant cell-wall degrading enzymes from these glands to enter roots. Several studies suggest bacterial genes for these enzymes arose through transfer from ancient microbial partners (Haegeman et al., 2011; Danchin et al., 2016).

The goal of this study was to examine a *Wolbachia*–*Cardinium* coinfection in depth, using comparative genomics and other tools to infer potential interactions and roles based on gene repertoire, tissue distribution, and evolutionary patterns. By studying this coinfection in a PPN we were able to explore the critical early history of these widespread endosymbionts because of their phylogenetic place near the root of their respective clades. Our results represent the first genomic analysis of a *Wolbachia*–*Cardinium* dual infection, and one of very few published *Cardinium* genomes.

## Materials and Methods

### Nematode Sample Collection and DNA Isolation

*Pratylenchus penetrans* were identified morphologically after collection from cultivated raspberry (*Rubus idaeus*) in Washington, DC, United States. Nematodes were cultured on cucumber (*Cucurbita pepo*) and raspberry plants at the USDA-ARS in Corvallis, OR, United States, and then specimens were extracted from roots by intermittent mist (mist chamber) for further study. Nematodes of mixed stages (*N* = 60) were transferred to water in preparation for FISH microscopy (see below). For single-individual PCR, adult females (*N* = 100) and males (*N* = 27) were placed into single 0.2 μl microcentrifuge tubes in a lysis buffer (Williams et al., 1992) containing proteinase K (880 μl of dH_2_O, 50 μl of 50 mM KCl, 50 μl of 0.05% gelatin, 4.5 μl of 0.45% Tween 20, 10 μl of 10 mM Tris, 3.3 μl of 60 μg/ml Proteinase K, and 2.5 μl of 2.5 mM MgCl_2_) and freeze-thawed five times (freeze at -80°C for at least 10 min and thawed at room temperature for 5 min) before being digested at 60°C for 90 min followed by a 95°C for 15 min. For Illumina sequencing, nematodes of mixed stages (*N* = 14,000) were prepared for DNA isolation and sequencing by grinding for 2 min with a motorized micropestle to disrupt the cuticles. DNA was then isolated using the Qiagen DNeasy Blood & Tissue Kit (Valencia, CA, United States) following the manufacturer’s directions.

### Fluorescence *in situ* Hybridization and Confocal Microscopy

Fluorescence *in situ* Hybridization was performed following an established protocol (Brown et al., 2015) using two probes designed to target the 16S rRNA of the endosymbionts *Cardinium* and *Wolbachia*. For *Cardinium*, a probe was designed (this study) to match all available *Cardinium* isolates and with mismatches to other major groups of bacteria, using the fluorophore Alexa 532 (yellow) with the sequence 5′-TCC GTC CCG AAG GAA CCC T-3′. Another probe matching the same region of the 16S rRNA was designed to match *Wolbachia*, using the fluorophore ATTO 633 (red) with probe sequence 5′-TGA AAT CCG GCC GAA CCG AC-3′. The protocol was as described previously (Brown et al., 2015), but with 35% vol/vol formamide to increase specificity. Nematodes were viewed using the Zeiss LSM 780 NLO Confocal Microscope at the Center for Genome Research and Bioinformatics (CGRB; Oregon State University, Corvallis, OR, United States). Negative controls included preparations without adding probes to check for autofluorescence, and *Caenorhabditis elegans* and *P. penetrans* without *Cardinium* and *Wolbachia* to check for non-specific binding of the probes.

### Polymerase Chain Reaction (PCR) and Sequencing to Assess Prevalence

Polymerase chain reaction was performed on DNA from individual nematodes using *Cardinium*- and *Wolbachia*-specific 16S rRNA primers (primer and thermal cycler conditions shown in **Supplementary Table 1**). Sequencing was performed to confirm amplification of the correct target for a subset of positive PCR products using the BigDye^®^ Terminator v. 3.1 Cycle Sequencing Kit (Applied Biosystems) using an ABI Prism^®^ 3730 Genetic Analyzer at the CGRB. For samples with negative PCR results for endosymbionts, PCR was performed using universal nematode primers (18S rRNA), to test for possible false negatives due to inhibitors or improper extraction or digestion of nematode DNA.

### DNA Library Preparation and Genome Sequencing

Bulk genomic DNA was sheared for 50 s using the Diagenode Bioruptor Pico (Denville, NJ, United States), with final peak fragment sizes of ∼600–700 base pairs (bp). Genomic library preparation was performed using Illumina TruSeq DNA Sample Preparation Kit (San Diego, CA, United States) following the manufacturer’s directions, with gel-excision of adapter-ligated fragments of ∼650–750 bp. Sequencing was performed with the Illumina MiSeq system with 301 cycles X 2 (paired-end) at the CGRB.

### Genome Assembly and Annotation

The focus of genome assembly in the present study was *Cardinium*, because the *Wolbachia* genome from this population was assembled and analyzed previously (Brown et al., 2016). Prior to assembly, Illumina reads were trimmed and quality filtered using FASTX-Toolkit v.0.014^1^. Initial assembly was performed using Velvet v.1.2.10 (Zerbino and Birney, 2008) with a range of kmers and CLC Genomics Workbench (Aurus, Denmark) then contigs were searched against the non-redundant (nr) database using BLAST+ v.2.2.29 (NCBI; National Center for Biotechnology Information). Resulting contigs with highest hits to *Cardinium, Amoebophilus*, or related Bacteroidetes, were then gap-filled to remove some of the “N” regions using GapFiller v.1-11 (Boetzer and Pirovano, 2012). Annotation was performed with Prokka v1.10 (Seemann, 2014). Resulting annotated contigs were aligned in CLC and Mauve 2.3.1 multiple sequence aligner (Darling et al., 2010), to identify redundant regions. Reads were then mapped back to scaffolds in CLC to check for regions with low coverage or unpaired reads suggestive of assembly errors. Duplicated regions with low coverage were collapsed and read pairing was used to re-join overlapping contigs to yield a final set of long scaffolds. Finally, unmapped reads were mapped back onto a set of reference genomes from *Cardinium*, the *Cardinium* plasmids, and *Amoebophilus* (listed in **Supplementary Table 4**), to look for additional genes or gene fragments that may have been missed in the final assembly. These methods provide several opportunities to obtain and extract the entire *Cardinium* genome from the data while conservatively guarding against erroneously including contigs from other contaminant bacteria.

### Phylogenomics and Ortholog Analysis

Phylogenomic analyses were performed for *Cardinium* using a set of 37 highly conserved single-copy genes (**Supplementary Table 2**) identified in previous studies (Santos-Garcia et al., 2014) with all available full genomes from GenBank from the Bacteroidetes Order Cytophagales, comprising the *Cardinium* family Amoebophilaceae, as well as families Cytophagaceae, Cyclobacteriaceae, and Flammeovirgaceae. Gene sequences homologous to these 37 genes in the draft genome of *Cardinium* from *P. penetrans* were identified using OrthoMCL (Li et al., 2003) (inflation value 1.5 and 60% match cutoff and *e*-value of 1*e-*3). Translation-guided alignment was performed for each gene in Geneious v.5.4.4 (created by Biomatters), and final alignments for nucleotides and amino acids were each concatenated into supermatrices for phylogenetic analysis. Because only two full *Cardinium* genomes were available, we also performed 16S rRNA and DNA gyrase subunit B (*gyrB*) phylogenetic analyses for nucleotides, including more taxa. Ambiguous and poorly aligned positions were removed using several different levels of stringency with Gblocks 0.91b (Castresana, 2000). Phylogenetic analysis was performed with maximum likelihood (ML) using RAxML-HPC2 v.8.0.24 (Stamatakis, 2006) under the GTR model with empirical base frequencies and likelihoods evaluated using the GAMMA and CAT among site rate variation estimators with free parameters estimated by RAxML and 1,000 bootstrap replicates. ML analysis of protein sequences was performed with the PROTCATGAMMAJTT and PROTCATDAHOFF substitution models with empirical base frequencies and 1,000 bootstrap replicates. Bayesian analysis was performed with MrBayes v3.2.6-svn run on XSEDE (CIPRES Science Gateway V 3.1) with the GTR+I+G model for 1,000,000 generations sampled every 500 generations, with two runs of four chains, with default priors and a burnin of 25%. To better address possible site-specific amino-acid or nucleotide difference, further analysis was performed using the CAT and CAT+GTR infinite mixture replacement models in PhyloBayes v3.2e64 (Lartillot et al., 2013). Orthologs obtained using OrthoMCL were analyzed against databases for orthologous groups of proteins (COGs) and pathways (MetaCyc, KEGG pathways, UniProtKB, and EMBL-EBI InterPro). Ortholog predictions were used as a basis for further analysis of repetitive genes or elements, eukaryote-domain containing genes, antifeeding phage-derived homologs, and potential insect toxin-related genes. Similarly, ortholog predictions served to identify potential candidate horizontally transferred genes (HGTs). In each case, predicted genes without matches to genes in outgroup taxa were subjected to several tests to evaluate alternate, possible HGT origin. First, the candidate HGT genes and their flanking gene regions were submitted to blastn and blastx searches against the full nt and nr databases, and the top hits (up to 100) were downloaded for phylogenetic analysis using RAxML in Geneious. Phylogenetic results for candidate HGT genes and their flanking regions were categorized into (1) non-HGT candidates for which the gene tree matched the species tree [e.g., either (Bacteroidetes (Cytophagales (Amoebophilaceae (*Candidatus* Cardinium)))); or (Alphaproteobacteria (Rickettsiales (Anaplasmataceae (*Wolbachia*))))], (2) recent HGT candidates for which the closest blast and phylogenetic matches to the gene were outside the phylum of the host genome and flanking matches, (3) older HGT candidates for which the closest matches were within the genus, but the next-closest matches were all outside the phylum of these host genomes. To further characterize candidate HGTs, an additional criterion was considered: the extent to which the blast bit scores of the phylum mismatch hits were greater than that of the flanking matched-phylum regions.

## Results

### Localization of *Cardinium* and *Wolbachia* in *P. penetrans* by Fluorescence *in situ* Hybridization (FISH)

**FIGURE 1:**
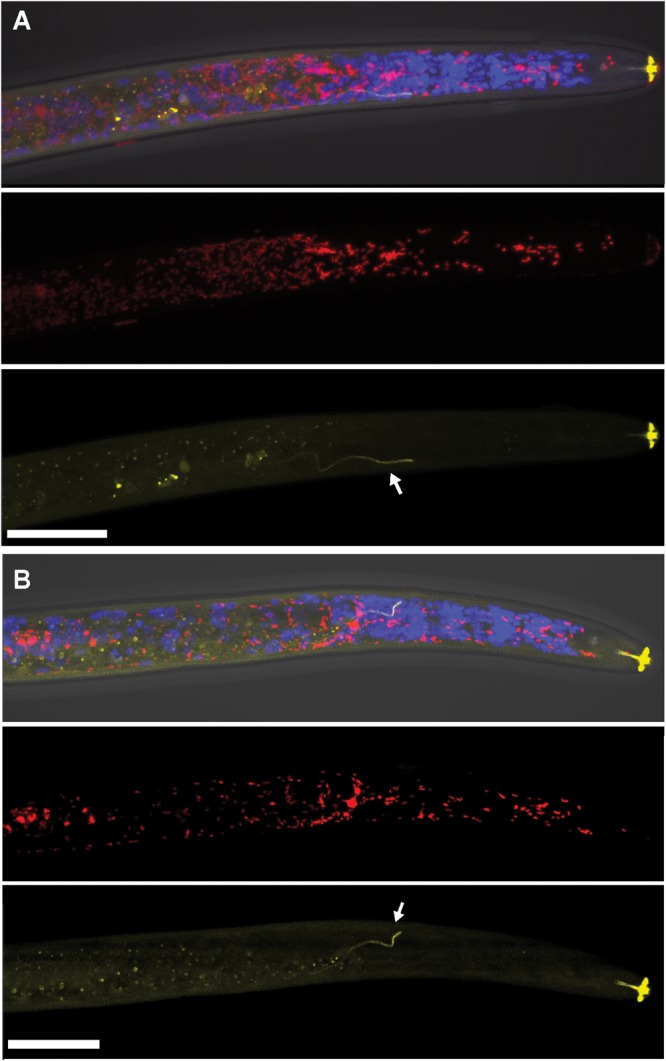
Localization of coinfecting *Wolbachia* wPpe (red) and *Cardinium* cPpe (yellow) in the anterior body of *Pratylenchus penetrans* by fluorescence *in situ* hybridization (FISH) and confocal microscopy. **(A,B)** Split panels showing confocal *z*-stacks combining signals from brightfield DIC, blue (DAPI), and red, and yellow FISH probes (top); wPpe (red FISH probe) alone (middle); and cPpe (yellow FISH probe) alone (bottom). Arrows point to excretory ducts. Yellow autofluorescence can be seen in lip region and excretory duct (arrows). Scale bars = 20 μm.

**FIGURE 2:**
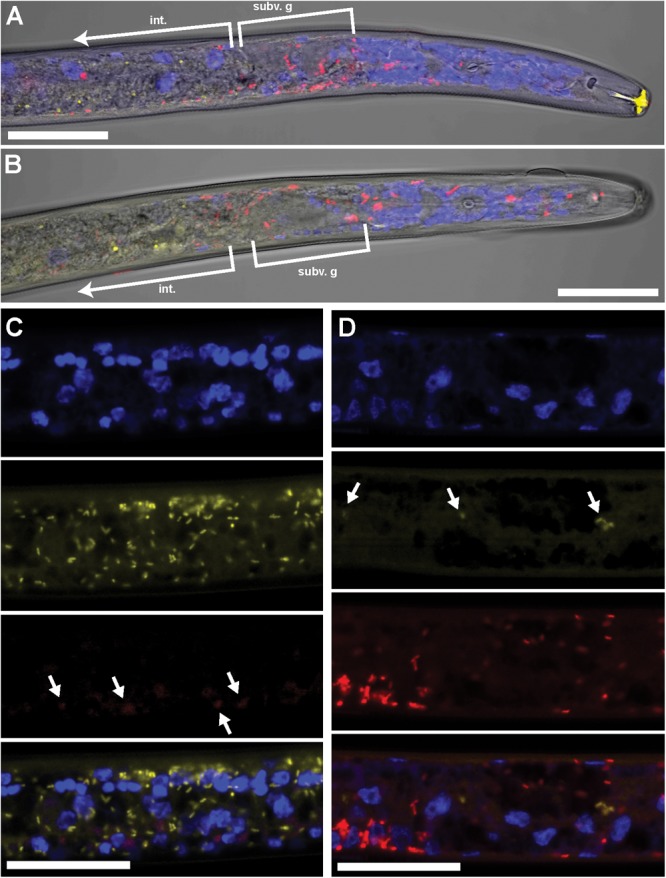
Coinfecting *Wolbachia* wPpe (red) and *Cardinium* cPpe (yellow) in *P. penetrans* in esophageal region and ovaries by FISH and confocal microscopy. **(A,B)** Confocal sections of esophageal regions combining brightfield DIC, blue DAPI, and red and yellow FISH probes, localizing *Wolbachia* wPpe (red) around subventral glands (subv. g) and elsewhere, with cPpe (yellow) in intestinal wall (int). Yellow autofluorescence can be seen in lip region. **(C,D)** Split panels of ovaries showing DAPI alone (top), yellow cPpe FISH probe alone (second panel), red wPpe FISH probe alone (third) panel, and all three signals (bottom), with arrows in **(C)** showing low levels of *Wolbachia* (red) and **(D)** low levels of cPpe (yellow). Scale bars = 20 μm.

**FIGURE 3:**
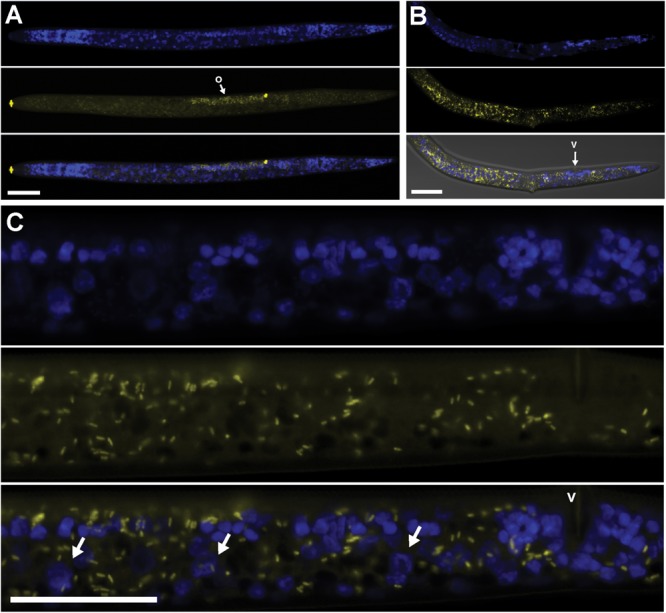
Localization of *Cardinium* cPpe in whole body and ovaries of *P. penetrans* by FISH and confocal microscopy. **(A–C)** Split panels showing nematode nuclei (blue DAPI stain) alone (top), cPpe (yellow FISH probe) alone (middle), and combined images (bottom). **(A)** Whole female with o = ovaries, and yellow autofluorescence of lip and spermathecal regions. **(B)** Female with combined brightfield DIC image (bottom), v = vulva. **(C)** High magnification of **(A)** showing ovaries and developing oocytes (arrows), v = vulva. Scale bars = 20 μm.

Using FISH, we confirmed the presence of *Cardinium* (hereafter, cPpe) and *Wolbachia* (hereafter, wPpe) in *P. penetrans*. FISH results (**Figures 1–3**) showed probe localization to coccoid-to-rod-shaped bacterial cells of both cPpe and wPpe distributed throughout most tissues of the body. These cells could be seen in tissues from the pharynx to the posterior tip of the body with cells most densely packed in the ovaries, anterior intestine, and esophageal regions. For nematodes with both cPpe and wPpe (yellow and red, respectively, in **Figures 1A,B, 2A–D**) generally one endosymbiont appeared more abundant than the other. For example, in the nematode pictured in **Figure 2C**, cPpe appeared more abundant, whereas in the nematode in **Figure 2D**, wPpe appeared more abundant. In coinfections in the anterior esophageal regions, cPpe tended to occur posterior to the esophageal glands (**Figures 2A,B**). Both endosymbionts appeared to localize to developing oocytes and ovarian walls (**Figures 2C,D**). In single infections with cPpe, the endosymbiont appeared to occur at high densities (**Figure 3**) including in the ovaries. Negative controls (*C. elegans* nematodes analyzed with probe and no-probe individuals of *P. penetrans*) showed no evidence of non-specific probe binding. Autofluorescence in the lips, excretory duct, spermathecal region, and cuticle were observed in the yellow-to-green range, beyond the emission range of the yellow Alexa532 probe, allowing this to be distinguished from non-specific probe binding.

### Coinfection Prevalence and Estimated Intensity of *Cardinium* and *Wolbachia*

Polymerase chain reaction analysis with *Cardinium*-specific primers for the partial 16S rRNA gene amplified in individual nematodes showed cPpe was found at 96% (123/127) in this population. *Wolbachia*-specific two-step PCR showed 48% of the nematodes (62/127) were coinfected with both wPpe and cPpe. All of the 12 cPpe PCR product sequences (GenBank Accession X) were identical and matched the 16S rRNA from the only previously published PPN *Cardinium* strain (NCBI DQ314214.1 from *Candidatus* Paenicardinium endonii) as its top blastn hit, with 97% sequence identity, confirming the specificity of these screening primers. Sequencing of wPpe products also revealed this *Wolbachia* 16S rRNA was identical to our original published sequence (GenBank Accession X). In this same group, all individuals that were infected with *Wolbachia* were coinfected with *Cardinium*. While relative read data cannot reveal coinfection due to the need for pooling nematodes for sequencing, it can provide an indirect method for approximating levels of endosymbiont per nematode (average intensity) when combined with PCR results. For example, Illumina read coverage for this population was 15.01X *Cardinium*, 16.70X *Wolbachia*, and 32.97X for a single-copy RNA polymerase II large subunit (RPB1) gene in *P. penetrans*. Thus, assuming the number of cells in each nematode is similar to that in *C. elegans* (∼1,000) and incorporating our prevalence estimates, the average number of *Cardinium* and *Wolbachia* cells per infected nematode would be 437 and 243, respectively (i.e., 15.01 × 0.96 divided by 32.97/1000 and 16.70 × 0.48 divided by 32.97/1000).

### Genome Features of *Cardinium* cPpe

**FIGURE 4:**
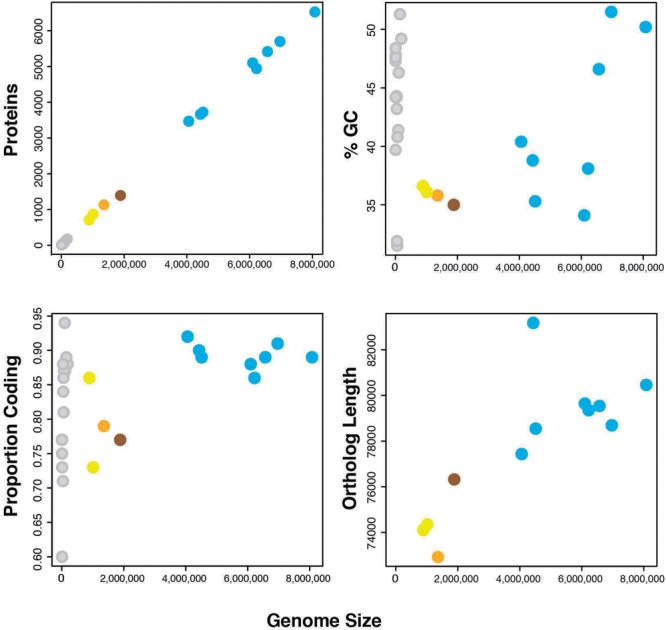
Genome features of *Cardinium* strains and outgroups (number of proteins, %GC, proportion of the genome that is coding, and sum of ortholog lengths for 37 genes in phylogenomic analyses). Colors: orange = *Cardinium* cPpe from *P. penetrans*, yellow = *Cardinium* strains cEper1 and cBtQ1, brown = *Candidatus* Amoebophilus asiaticus, blue = outgroup Bacteroidetes, and gray = plasmids from the above bacteria. (Full data are given in **Supplementary Table 4**.)

Genome sequencing, initial assembly, and final assembly details are listed in **Supplementary Table 3** (data deposited in NCBI SRA accession SRR3097580). The final cPpe assembly consisted of 27 scaffolds with an N50 of 163,560 bp and a total length of 1,358,214 bp, serving as the draft genome. This genome revealed 35.8% GC, 1,131 predicted proteins, and a full set of rRNA and tRNA genes (3 and 35, respectively), with 79% of the genome comprising coding regions and 41% of predicted proteins having no known function. There was no evidence for a plasmid (see further details below). Genome feature comparisons between *Cardinium* cPpe and available relatives with sequenced genomes showed generally positive relationships between genome size and number of protein coding genes, % GC content, proportion of genome that is coding, and ortholog length with a few exceptions (**Figure 4** and **Supplementary Table 4**). Two related bacteroidetes, *Flavobacterium johnsonae* and *Marivirga tractuosa* had somewhat lower GC content, relative to their genome size, and *Cytophaga huchinsonii* had a longer ortholog length relative to genome size, compared with the pattern for others. *Cardinium* cEper1 had a higher proportion coding sequence relative to genome size.

### Phylogenomic Place of *Cardinium* cPpe

**FIGURE 5:**
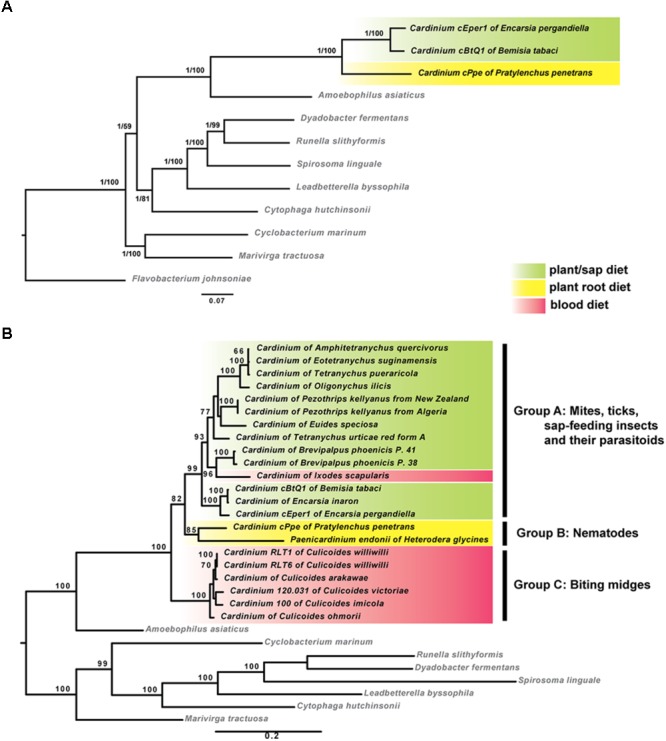
Maximum-likelihood phylogenies of *Cardinium* strains and outgroup species. **(A)** Tree generated from 37 protein coding genes comprising 72,194 nucleotide alignment positions generated with RAxML under the GTR model, showing bootstrap values (1,000 replicates) and posterior probabilities from MrBayes. **(B)** Tree generated from 16S rRNA and *gyrB* genes comprising 2,426 nucleotide positions calculated as described for **(A)**. Shading indicates major *Cardinium* groups. For both **(A,B)**, similar analyses with varied alignment filtering stringency in Gblocks, different outgroups, different models, or using amino acid sequences, produced similar supported clades. (List of 37 genes in **Supplementary Table 2**; accession numbers in **Supplementary Table 4**; additional phylogenies in **Supplementary Figures 1, 2**.)

Phylogenomic analyses based on concatenated sequences from 37 protein-coding genes showed that cPpe from *P. penetrans* formed a consistent and well-supported early branch basal to *Cardinium* strains cBtQ1 and cEper1 (**Figure 5A**). This strongly supported topology was obtained from both ML and Bayesian analyses performed on every alignment stringency and model or parameter adjustment, including nucleotide and amino acid alignments (**Figure 5A** and **Supplementary Figure 1**). To include a wider range of strains including the only other available *Cardinium* strain from a PPN, *Cardinium* from *H. glycines*, further phylogenetic analyses were performed using two genes, 16S rRNA and *gyrB* (**Figure 5B**). Results consistently placed cPpe from *P. penetrans* as an early branch basal to *Cardinium* group A (comprising most strains from mites, spiders, and insects), with *Cardinium* group C (comprising strains from biting midges) as a sister group, and again, this topology was strongly supported regardless of evolutionary model, analysis method, or alignment stringency (**Figure 5B** and **Supplementary Figure 2**).

### Gene Repertoire and Ortholog Analysis of *Cardinium* cPpe and Outgroups

**FIGURE 6:**
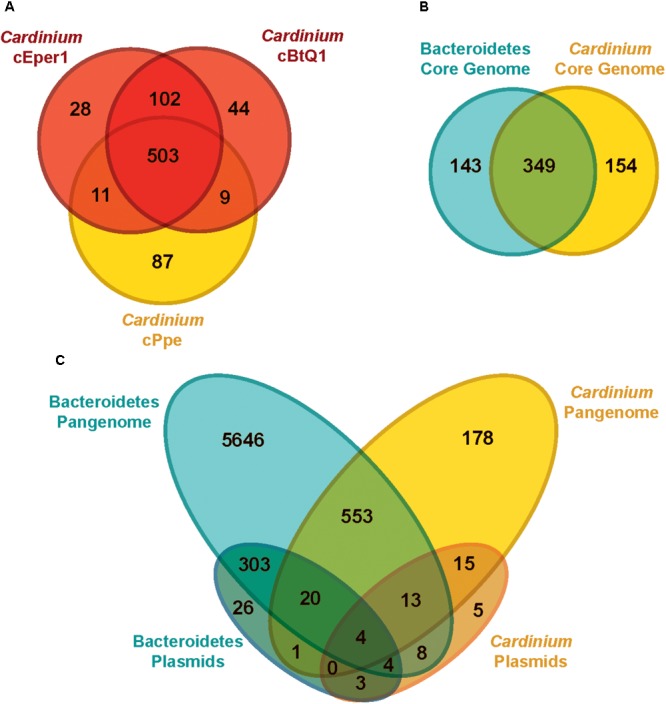
Genome content (ortholog) comparisons among *Cardinium* strains and outgroups. **(A)** Genes shared between the three available *Cardinium* strains, cEper1 from *Encarsia* wasps, cBtQ1 from *Bemisia tabaci* whiteflies, and cPpe from *P. penetrans* plant-parasitic nematodes (PPN) (gene list in **Supplementary Table 7**). **(B)** Gene content overlap for completely shared “core” genes among groups of strains for Bacteroidetes in **Supplementary Table 4** (gene list in **Supplementary Table 8**). **(C)** Genetic repertoire similarity for all genes (“pangenome”) between different groups, including plasmids (gene list in **Supplementary Table 9**).

Most genes from *Cardinium* cPpe with sequence similarity to proteins of known function appeared to encode genes for cellular structure, catabolic, and replicative processes. Among the three *Cardinium* genomes (**Figure 6A**), there was greater overlap between cEper1 and cBtQ1 (102 uniquely shared genes) than between either of these strains and cPpe, while cPpe had the largest number of unique genes, at 87. Notably, *Cardinium* strains shared a core set of 349 orthologs with other species in the Order Cytophagales (**Figure 6B**), while 154 core shared *Cardinium* genes were not universally shared with others in the group. From this set of 154 core shared *Cardinium* orthologs, 26% (40 genes) had no known function, 14.9% (23 genes) had only general functional prediction, and 4.5% (7 genes) were transposases. Pangenome comparisons for *Cardinium* and outgroup species in the Cytophagales (**Figure 6C**) showed 178 of 7,175 (or 2.5%) of the predicted gene clusters consisted of *Cardinium* orthologs with no homolog in the outgroup, as well as many singleton genes unique to each *Cardinium* strain.

*Cardinium* plasmids pCher and pCHV, from strains cEper1 and cBtQ1, respectively, showed few shared genes with outgroups or cPpe (**Figure 6C**). The majority of *Cardinium* plasmid genes (78.8%, or 41 of 52) were not found in the outgroups, while the majority of outgroup plasmid genes (96.9%, or 350 of 361) were not found in *Cardinium* plasmids pCher and pCHV. Few genes from cPpe showed homology with pCher and pCHV plasmids (12 of 52 genes) and these genes in cPpe occurred on different contigs or in distant locations along contigs, never in adjacent locations.

A search for homologs to toxin candidates in cPpe revealed no homologs for *Amoebophilus* insect-toxin-like genes (Aasi_1414 and Aasi_1417) or antimicrobial lasso peptides (Aasi_0899 and Aasi_1417) in any *Cardinium* strain. Nor were there homologs to the cBtQ1 plasmid parasitoid-toxin-like genes CHV_p018 or CHV_p021 in cPpe. *Cardinium* cPpe and coinfecting *Wolbachia* wPpe showed no loci homologous to any of the predicted toxin-antidote genes implicated in *Wolbachia* CI (WPA_0282 CidB, WPA_0283 CidA, WPA_0294 CinA, WPA_0295 CinB). However, cPpe had a full set of genes for lipid A synthesis (*lpxA, lpxC, lpxD, lpxB, lpxH, lpxK, lpxL*/*htrB*).

A search for ubiquitin-related domains [e.g., UBOX, FBOX, and ubiquitin-specific proteases (USP)] matching those reported previously in *Amoebophilus* revealed several matches in *Cardinium* strains. For example, from among the nine UBOX domain genes in *Amoebophilus*, one of these (Aasi_1435) shared homology with several ankyrin repeat containing genes from *Cardinium* strains cBtQ1, cEper1, and cPpe, with matches to outgroup bacteroidetes, and having homology to a putative beta-lactamase precursor *hcpC*. For the FBOX domain containing genes, among the 16 reported in *Amoebophilus*, 11 of these (including Aasi_0025) clustered together into a group of leucine rich repeat containing orthologs with homology to formamidopyrimidine/5-formyluracil/5-hydroxymethyluracil DNA glycosylase (MutM). This predicted protein also clustered with 18 predicted proteins in cPpe but did not match any other *Cardinium* strains or bacteroidetes outgroup taxa. In cPpe, the 18 gene locations were highly clustered, localized to one set of eight tandem genes, and four other sets of two genes, and only two homologs located singly. Blastn and blastp nucleotide and protein searches of these 18 homologs showed typically short (∼60 bp) high similarity (80%) matches, or low similarity (∼50% amino acid) matches across 70% of the protein. Matches included a broad range of distantly related eukaryotes including many fungi. For the USPs identified previously in *Amoebophilus*, there were no homologs in this data set, but two unrelated USPs, both ubiquitin carboxyl-terminal hydrolases, were identified exclusively in the *Cardinium* clade, having as much as 37% amino acid similarity over 74% of their length within this clade. Blastp showed the next closest matches as eukaryotes, with insects, algae, and nematodes as the highest hits (27% amino acid similarity across 79% of the length of these genes).

### Duplicated Genes and Expanded Gene Families

**FIGURE 7:**
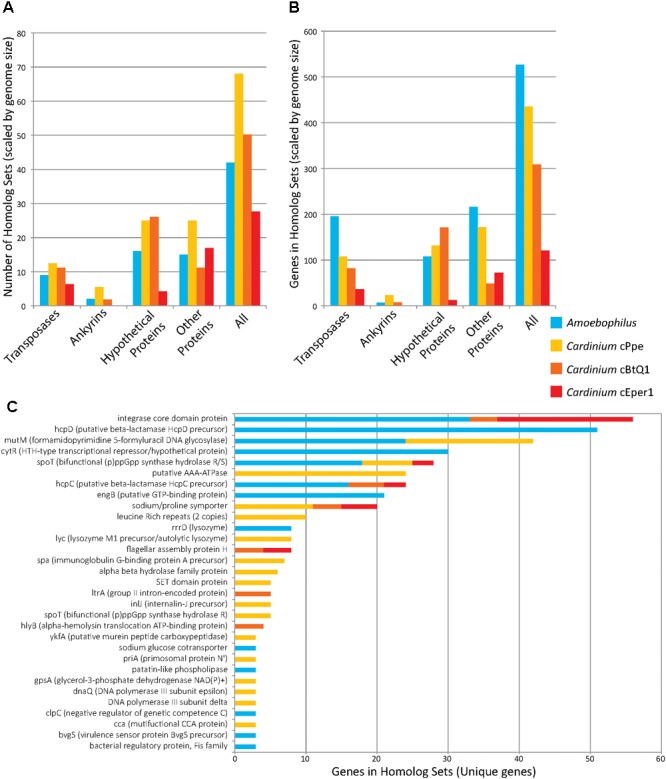
Gene copy number expansions as measured by orthoMCL orthologous gene sets for *Candidatus* Amoebophilus asiaticus and *Cardinium* strains from *P. penetrans* (cPpe), whitefly (cBtQ1), and *Encarsia* wasps (cEper1). **(A)** Number of homologous sets of genes by protein category, normalized for differences in genome size. **(B)** Gene number among all homologous sets, normalized for genome size, for sets with three or more homologs. **(C)** Genes names and numbers in homolog sets for predicted protein-coding genes of known function (“unique genes”). In all comparisons **(A–C)**, only sets with three or more homologs were used.

While the genome sizes of all *Cardinium* endosymbionts were smaller than those of the outgroups, several genes occurred within orthologous clusters with multiple copies per genome (**Figure 7** and **Supplementary Table 5**). Many homologous gene sets encoded transposase domain-containing proteins. For example, one such expansion consisted of 74 homologs in *Amoebophilus*, having a DDE-motif transposase with genes broadly distributed across the genome, with no homologs to other genes in the set of taxa analyzed. Another expanded DDE gene occurred in 36 copies in *Amoebophilus*, with 25 copies in cBtQ1 but only 2 and 1 copy in cPpe and cEper1, respectively. A further transposase expansion in cPpe consisted of 13 copies of an IS66 transposase located in two clusters of tandemly located copies (5 and 3) as well as single copies dispersed across the genome. This gene was homologous to a single-copy gene in *Amoebophilus*. The largest expansion in cPpe was the set of 24 homologs of a putative AAA-ATPase with only one homolog in each of the other *Cardinium* strains, and no other homologs in outgroup taxa. This gene displayed highest blast similarity to alphaproteobacteria, particularly *Wolbachia* and *Rickettsia*, as discussed below. The next most expanded gene in cPpe was *mutM* (formamidopyrimidine/5-formyluracil/5-hydroxymethyluracil DNA glycosylase) with 18 copies. This gene had 24 homologs in *Amoebophilus*, and also contained an FBOX domain as discussed earlier. There were 18 homologs of *spoT*, a bifunctional (p)ppGpp synthase hydrolase R/S/sodium glucose cotransporter, in *Amoebophilus*, while *Cardinium* strains cPpe, cEper1, and cBtQ1 had only seven, three, and two homologs, respectively. Conversely, notable non-expanded genes in *Cardinium* cPpe include the beta hydrolase precursors (*hpcC* and *hpcD*) and integrase core domain proteins. Other differences in gene numbers in this dataset were that cPpe had more genes in gene clusters overall, compared with other *Cardinium* strains when data were scaled for genome size differences (**Figure 7**). In other words, when accounting for genome size, it would appear cEper1 had fewer gene expansions than other taxa in this dataset. This pattern varied depending on the class of genes considered. While *Amoebophilus* had fewer homolog sets than *Cardinium* cPpe, it had more gene expansions within these clusters, suggesting recent expansions of a small subset of genes.

### Phage-Derived Protein Secretion System

**FIGURE 8:**
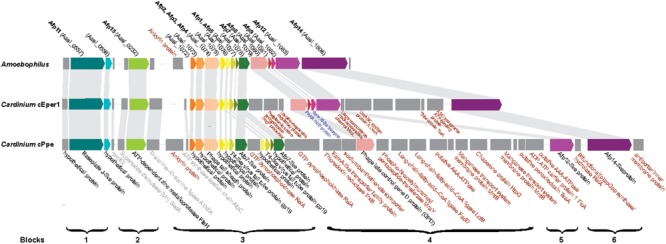
Comparison of antifeeding prophage-like protein secretion system synteny among Amoebophilaceae showing flanking genes for transport and other relevant functions of interest. Color block arrows show genes homologous to *Serratia entomophila* pADAP “Afp” antifeeding prophage and other universally conserved Amoebophilaceae genes, whereas dark gray blocks show flanking genes of interest. Synteny regions are shown with light gray connectors. Proteins in black font are conserved in this clade; proteins in gray font are conserved among only *Cardinium* strains; proteins in red font are uniquely located in specific *Cardinium* strains; proteins in blue font appear to be lost in *Cardinium* from *P. penetrans* (cPpe).

Previously, anti-feeding prophage tail-derived genes were described in *Amoebophilus* and *Cardinium* cEper1. Because of interest in these as the major secretion system in these endosymbionts and their association with the distinctive laminar fibrils in *Cardinium*, we compared these genes in detail. Sixteen of the genes in the cassette of phage-like protein translocation structures (PLTS) were conserved across *Cardinium* strains, with only two genes missing in cPpe (**Figure 8**). Comparison of synteny and flanking genes showed the original three blocks of this 16-gene unit were broken into up to six blocks in *Cardinium*, with the conserved retention of an 8-gene block displaying almost complete synteny with the exception of some duplicated copies in cPpe (**Figure 8** and **Supplementary Table 6**). Blocks 1 and 2 (cPpe genes 00191, 00192, and 00050) had synteny among immediately flanking genes for all three *Cardinium* strains. The third 13-gene block in *Amoebophilus* was broken into three or four blocks in *Cardinium*, with little synteny in adjacent genes. This block flanks another gene of interest a relA/spoT/bifunctional ppG(pp) synthase hydrolate gene (cPpe 01022) in cPpe that also has a number of homologs in this strain. Adjacent genes in these strains appear to be mostly transport-related. However, adjacent to the phage late control gene/baseplate assembly gene (cPpe 000783) is a series of genes potentially important in transport. Block 5 contains two putative AAA-ATPases and two manganese transport gene homologs. Block 6 comprises one final large conserved protein that also has no conserved synteny with flanking gene regions but is annotated with similarity to a leucine permease transcriptional regulator.

### Biosynthetic Capacity of *Cardinium* cPpe

Based on comparison with general predicted metabolic pathways, cPpe appears to be missing a majority of genes for metabolic pathways necessary for independent survival outside host cells (**Supplementary Figure 3**). However, many of these apparently missing functions are entirely or mostly absent from all members of the *Amoebophilus–Cardinium* clade, such as most amino acid biosynthesis pathways (e.g., arginine, histidine, isoleucine, leucine, valine, methionine, threonine, tryptophan, chorismate, glutamate, proline, and serine) and many other biosynthetic pathways (e.g., those for carotenoids, folate, heme, niacin, pantothenate, pyridoxine, riboflavin, sulfates, and thiamine) (**Supplementary Figure 3**). A few synthetic functions were fully or mostly retained universally in this clade, including synthesis pathways for alanine, glycine, PRPP, phospholipids, peptidoglycan, lipid A, and basic replication/transcription and recombination/repair functions. Of interest in this analysis are predicted functions that differ among members of this clade. For instance, biosynthetic capacity for lysine, asparagine, and aspartate synthesis appear mostly present in *Amoebophilus* only, whereas several functions appear partly or fully present in some or all *Cardinium* strains only. For example, some strains have genes for methionine, cysteine, biotin, and lipoate biosynthesis. While most genes for methionine synthesis are missing, the second gene (*metB*, cystathionine gamma-synthetase) appears to be universally conserved in *Cardinium*. In contrast, only *Cardinium* cEper1 and cBtQ1 have some or all of the biotin and lipoate genes. The latter appear to be entirely missing in cPpe (**Supplementary Figure 3**). No members of the clade had any evidence of plant cell wall degrading enzymes.

### Potential Horizontally Transferred Genes in *Wolbachia* wPpe and *Cardinium* cPpe

**FIGURE 9:**
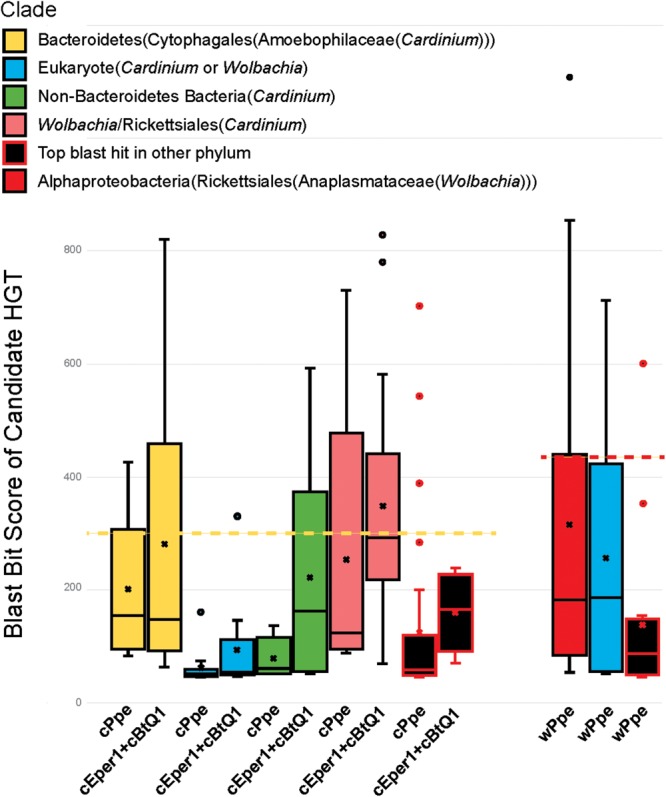
Relative ranges of blast bit score for candidate HGT genes, by phylogenetic clade of blast hits. Relative divergence (in blast bit score) of candidate HGTs compared with ordinary flanking genes in *Cardinium* cPpe from *P. penetrans, Cardinium* cBtQ1/cEper1 from insects (yellow boxes), and *Wolbachia* wPpe from *P. penetrans* (red boxes). Black boxes indicate top blast hits outside the clades *Cardinium* or *Wolbachia*, while other colors indicate the top hit matches *Cardinium* or *Wolbachia*, but all remaining hits match distant taxa (blue = eukaryotes, green = other bacterial phyla, pink = *Wolbachia*/Rickettsiales). Dotted lines serve as a visual guide to indicate the range in blast bit scores relative to the upper quartile of cPpe and wPpe genes for which the gene tree matches the species tree. (Box and whisker plot: box represents interquartile range, whisker represents remaining quartiles, with midline and ‘x’ denoting the median and mean, respectively, and outliers as dots beyond 1.5 times the interquartile range.)

**FIGURE 10:**
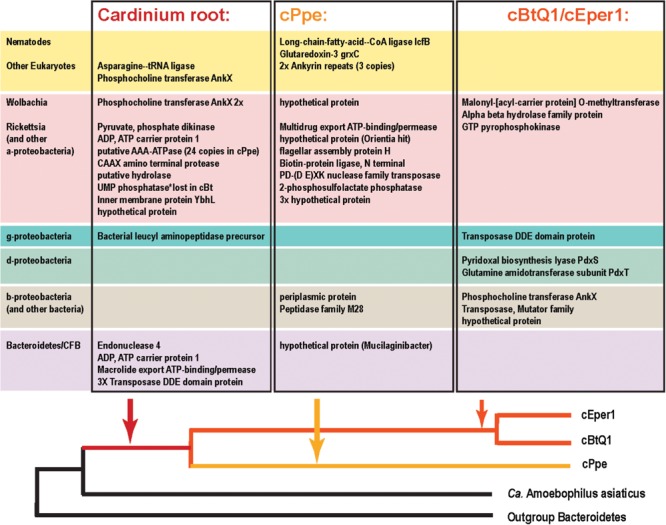
Candidate horizontally transferred genes (HGTs) in *Cardinium* strains from *P. penetrans* (cPpe), whitefly (cBtQ1), and *Encarsia* wasps (cEper1) based on absence of blast similarity to proteins from outgroup genomes and presence of high blast similarity to other organisms. This list emerged from a filtered subset of blastx hits comprising the outliers in hit length and similarity shown in **Supplementary Figure 3** and **Supplementary Table 10**, and thus is a fairly conservative subset of candidate HTGs. Source taxa for these genes are shown to the left and place of entry into the *Cardinium* lineage is shown at the bottom.

In addition to several candidate HTGs already listed in previous sections, additional blast and phylogenetic analyses were performed to examine genes that may be candidates for HGT from *Wolbachia* wPpe and *Cardinium* cPpe (**Figures 9, 10, Supplementary Figures 4–7** and **Supplementary Table 10**). Ortholog analysis recently published from wPpe revealed 202 predicted proteins having no ortholog across other *Wolbachia* strains (Brown et al., 2016). Of these, 10 proteins with functional prediction showed highest blastp similarity to proteins from non-*Wolbachia* clades. One of these (wPpe_00273, 283aa) had 70% amino acid similarity to endonuclease IV from *Cardinium* strains cEper1 and cBtQ1. Two further candidate HGTs in wPpe were annotated as lysine-metabolism proteins: aspartate-semialdehyde dehydrogenase (wPpe_00038, 328aa) matching the intra-mitochondrial alphaproteobacterial genus *Midichloria* with 60% amino acid similarity, and saccharopine dehydrogenase (wPpe_00280, 371aa) matching the amoeba-endosymbiotic gammaproteobacterial genus *Legionella*, at 56% amino acid similarity. Two other notable candidate HGTs to wPpe are for proteins involved in carotenoid synthesis: an apocarotenoid-15,15′-oxygenase (wPpe_00201, 524aa) matching gammaproteobacteria with 51% amino acid similarity.

From the list of 154 shared core *Cardinium* genes not shared with outgroups several had high blast similarity to other taxa and a phylogenetic pattern strongly suggesting of HGT origins (**Figures 9, 10** and **Supplementary Table 10**). These include a gene for a mitochondrial carrier protein (cPpe 01095) with 66–67% amino acid identity between *Cardinium* strains, but at most only 33% amino acid similarity to any other protein in blastp protein searches against the ref_seq database, with the highest similarity to *Neorickettsia* species over 90% of the protein length. All remaining similar genes were from a range of eukaryotes and matched the calcium-binding mitochondrial carrier protein SCaMC-1 described as catalyzing uptake of adenine nucleotides across the inner mitochondrial membrane and protecting against oxidative stress. Another candidate HGT from *Cardinium* strains was the AP site-preferring DNA repair gene endonuclease 4, *nfo* (cPpe 00958), which shared about 70% amino acid similarity among *Cardinium* strains and had highest blast similarity to *Wolbachia* (68% amino acid identity) with 58–61% similarity to a wide range of Parachlamydiales, including *Simkania negevensis*, a species with a wide host range, including amoeba. A further gene from among this shared core set of *Cardinium* genes was a ubiquitin carboxyl-terminal hydrolase (cPpe 00816), which encodes a large, 411 amino acid protease with distant similarity to a wide range of eukaryotes including the nematode CRE-USP-3 ubiquitin protease.

From the 178 predicted orthologs present only in the pangenome of *Cardinium* strains, but absent in outgroups, most genes (64%) had no predicted function or could only be annotated with a general functional prediction. Among the remaining genes, 15 (8.4%) contained ankyrin repeats and 9 (5%) encoded transposases. Coinfecting *Wolbachia* wPpe was similar with 148/202 (73%) of genes with no function prediction among unshared clusters, and 5% ankyrins. One notable gene in this list of candidate HGTs in *Cardinium* was a large toxin A gene (766 amino acids) unique to cPpe, with highest blast similarity to a glycosyl transferase in *Regiella insecticola*, a gammaproteobacterial symbiont shown to be parasitoid-protective in aphids and toxin A in *Pseudomonas* spp. and other gammaproteobacteria such as *Providencia burhodrogranariea* (associated with insect hemolymph) and the symbiont *Candidatus* Fukatsuia symbiotica.

Among the genes listed in **Figure 10** that were shared among *Cardinium* strains were ankyrins potentially from *Wolbachia* and some eukaryotes, and many other genes including an ADP, ATP carrier protein, and multiple copies of a putative AAA-ATPase and other genes from *Rickettsia*-like alphaproteobacteria. Candidate HGTs specific to cPpe included a multidrug export ATP-binding/permease and biotin-protein ligase from Rickettsias, and various other genes shown in **Figure 10**. There was evidence of two strong candidate genes in cPpe alone from nematodes: a long-chain-fatty-acid-CoA ligase *lcfB* (cPpe 00786) matching a mitochondrial gene involved in lipid storage in larval development, and a glutaredoxin-3 *grxC* (cPpe 01161) matching thioredoxin-like superfamily proteins in nematodes. Analysis of the long-chain-fatty-acid-CoA ligases located adjacent to the putative phage-derived protein secretion system (**Figure 8**) showed these two copies have different origins, one likely within the bacteroidetes and the other somewhere within the Nematoda (**Supplementary Figure 6**), but missing nematode intron regions (**Supplementary Figure 8**). Two other cPpe genes with high similarity in *Wolbachia*/*Rickettsia* were a transposase (01133) and a gene of unknown function (00500) (**Supplementary Figures 5, 7**). Several other candidate HGT genes were shared only among insect endosymbiont *Cardinium* strains. These include two pyridoxal-5′-phosphate (vitamin B_6_) biosynthesis genes, *pdxS* (pyridoxal biosynthesis lyase), and *pdxT* (glutamine amidotransferase subunit pdxT), which were present in cEper1 and cBtQ1 and adjacent on the chromosome, but appeared to have no homologs in cPpe or outgroups, and had highest blastp similarities (about 71 and 41%, respectively) over most of their lengths with other phyla of bacteria, such as Firmicutes and Deltaproteobacteria. This *Bacillus*-like biosynthetic pathway is in contrast to *Cardinium* outgroup and *Wolbachia*, appearing to have the *Escherichia coli*-like pathway for pyridoxal-5′-phosphate synthesis with genes *serC, pdxA, pdxJ*, and *pdxH*. Biotin (vitamin B_7_) biosynthesis genes, which were located in an operon in cEper1 and were mostly present in cBtQ1 (previously recognized as a case of HGT between *Cardinium* and *Wolbachia*) were not found in cPpe, despite extensive blast searches of assembled contigs and raw reads from *P. penetrans*.

## Discussion

Our study provides the first examination of coinfecting endosymbionts *Cardinium* and *Wolbachia* in a nematode. In addition, this study includes a comparative genomic analysis of a new *Cardinium* strain – one of few *Cardinium* genomes available to date. This dual infection in a PPN is of major interest both for its potential effects in modifying the biology of this significant crop pest, and for its phylogenomic place as an early branch in these widespread terrestrial invertebrate endosymbionts. Despite a large number of genes with unknown function in this endosymbiotic branch of Bacteroidetes (family Amoebophilaceae), results here illuminate gene repertoire changes potentially connected with changes in host and role, and possible ancient gene exchanges in the *Wolbachia*–*Cardinium* dual endosymbiosis.

Microscopic features and PCR confirmed the presence of a dual coinfection reported previously in a genome skimming survey (Denver et al., 2016), and confirmed key features previously reported for *Wolbachia* and *Cardinium* in PPNs. As shown previously, *Wolbachia* wPpe and wRad have dispersed tissue distribution, with somewhat concentrated densities in the ovaries and anterior intestine (Haegeman et al., 2009; Brown et al., 2015), consistent with vertical transmission and a possible role in host nutritional supplementation. For *Wolbachia*, PCR prevalence data showed that about half of the *P. penetrans* individuals analyzed were infected, indicating a non-obligate association and potentially imperfect vertical transmission in *P. penetrans*, contrasting with data previously reported for wRad in *R. similis* (Haegeman et al., 2009). The tissue distribution of *Cardinium* in *P. penetrans* matched that reported from cyst nematodes *G. rostochiensis, H. goettingiana, H. avenae*, and *H. glycines* (Shepherd et al., 1973; Endo, 1979; Walsh et al., 1983a,b; Noel and Atibalentja, 2006; Yang et al., 2017), with presence throughout the tissues of the nematode body including the ovary walls and oocytes, although it was unclear whether cPpe occupied the esophageal glands. In contrast to wPpe, PCR showed cPpe to be at nearly 100% prevalence in this *P. penetrans* population, consistent with data from *Heterodera* spp. (Walsh et al., 1983a,b). In theory, obligate mutualism could lead to 100% prevalence, but this is not known in *Cardinium*. Instead, high prevalence may be a sign of strong reproductive parasitism: CI and high *Cardinium* prevalence have been observed in spider mites (Gotoh et al., 2007; Kopecky et al., 2013; Zhao et al., 2013; Zhang et al., 2016a; Zhu et al., 2018) and *Encarsia* wasps (Hunter et al., 2003; Zchori-Fein et al., 2004; Zchori-Fein and Perlman, 2004; Perlman et al., 2008; White et al., 2011; Gebiola et al., 2017; Mann et al., 2017).

While ours is the first study of coinfection with *Wolbachia* and *Cardinium* in nematodes, coinfection has been studied in mites (Gotoh et al., 2007; Ros et al., 2012; Zhao et al., 2013; Zélé et al., 2018; Zhu et al., 2018), planthoppers (Nakamura et al., 2009, 2012), *Encarsia* wasps (White et al., 2009, 2011), *Culicoides* midges (Mee et al., 2015), and thrips (Nguyen et al., 2017). The effects of coinfection in insects appear to be mixed, with several studies showing no particular synergistic or competitive effect or interaction (Gotoh et al., 2007; White et al., 2009; Nguyen et al., 2016), while others suggest a possible facilitation or enhanced CI phenotypes in dual infection (Nakamura et al., 2009, 2012; Zhao et al., 2013; Zélé et al., 2018). Numerous studies have examined the tendency of co-occurring endosymbionts to evolve metabolic complementarity in hosts with nutrient depleted plant-based diets (Bennett and Moran, 2015; Luan et al., 2015; Sudakaran et al., 2017). For reasons that are unclear, to our knowledge, *Wolbachia* and *Cardinium* have not been implicated in metabolic complementation. It may be that while strains of *Wolbachia* can evolve mutualist roles (e.g., in filarial nematodes, bedbugs, and others) (Hosokawa et al., 2010; Landmann et al., 2014; Nikoh et al., 2014; Gerth and Bleidorn, 2016; Lefoulon et al., 2016), *Cardinium* has lost too much biosynthetic capacity. In *P. penetrans*, it appears *Wolbachia* wPpe has lost riboflavin (vitamin B_2_) synthesis (Brown et al., 2016) which was proposed as a probable side-benefit conferred by even the reproductive manipulator strains (Moriyama et al., 2015; Brown et al., 2016), and has no biotin (vitamin B_7_) or thiamine (vitamin B_1_) cassettes (Brown et al., 2016; Gerth and Bleidorn, 2016). Alternatively, the ability to manipulate host reproduction, especially by CI, may preclude the evolution of a strong mutualist role. CI is not known in *Wolbachia* wPpe (Brown et al., 2016). In *Cardinium* cPpe and no homologs to known CI genes were found, but the rarity of males in our coinfected *P. penetrans* population (Personal Comm.) raise the question of the endosymbionts’ role in sex ratio distortion.

Phylogenomic support for long, early branches in both *Cardinium* (this study) and *Wolbachia* (Brown et al., 2016) from PPNs suggests long histories of endosymbiosis and possibly also dual endosymbiosis. Strains of each of these endosymbionts confidently clustered together with strains from other PPN hosts. This result tends to reject the possibility of horizontal transmission of *Cardinium* or *Wolbachia* between PPNs and other taxa – a phenomenon of interest between groups of insect-inhabiting *Wolbachia* and *Cardinium* (Ros et al., 2012; Yang et al., 2012; Mee et al., 2015; Lefoulon et al., 2016; Zhang et al., 2016a), particularly by transmission through plant tissues (Gonella et al., 2015; Li et al., 2016). Thus, we suggest that the early predominance of plant hosts in the phylogenies of both *Wolbachia* and *Cardinium* support the idea that plant transmission may have led to ancient host switches from the below-ground (nematode) hosts, to above-ground (insect) hosts. While we argued previously, based on phylogenies and comparative genomics that early *Wolbachia* strains may have played a role in supporting a plant diet (Brown et al., 2016), perhaps through supplementation of iron/heme and later other nutrients absent in plant sap, current results for *Cardinium* can be interpreted in a similar light. While our phylogenies placed *Cardinium* strains from *Culicoides* (biting midges) as probable outgroups to PPN *Cardinium* strains, these midges’ diets are primarily nectar until females take a blood meal prior to laying eggs. Furthermore, while *Cardinium* is also widespread in spiders and mites, the majority of Acari hosts are plant-sap feeders, and the insect group with the highest prevalence, planthoppers (Fulgomorpha) (Nakamura et al., 2009, 2012), an early-branching sap-feeding clade with a mixture of nutritional symbionts including yeasts (Sudakaran et al., 2017).

Comparative genomics of cPpe and wPpe provide indicators of metabolic and ecological roles of these endosymbionts. This is of interest given past speculation that these symbionts may play a mutualist role (Endo, 1979). For example, on the basis of high prevalence, intimate contact with ribosomes and the host nuclei and presence in the esophageal glands, previous authors speculated that *Cardinium* in *H. glycines* may be mutualists (Endo, 1979). However, our genomic data (genome size, GC content, ortholog length, proportion coding, gene expansions) suggest cPpe is similar to other endosymbionts (*Amoebophilus*, cBtQ1, cEper1) that are not obligate mutualists, and does not follow the extreme reduction and AT-compositional bias seen in *Xiphinematobacter*, the endosymbiont of the PPN *X. americanum* (Brown et al., 2015), or the pattern typical of a wide range of obligate mutualist symbionts from insects (Bennett and Moran, 2015; Luan et al., 2015; Sudakaran et al., 2017). However, the forces leading to genome size reduction (bottleneck during vertical transmission, deletion bias, relaxed selection for genes redundant to living within a host cell, and replication advantage) may not be linear over time or constant among groups. Thus, specific metabolic enrichment is a far better indicator in comparative studies.

Gene repertoire analysis of *Wolbachia* wPpe suggested a role intermediate between mutualism and reproductive parasitism (Brown et al., 2016), whereas here gene repertoire analysis of coinfecting *Cardinium* cPpe suggests interaction with host mitochondria and a role in host-derived fatty acid metabolism. For example, cPpe had a candidate HGT long-chain-fatty-acid-CoA-ligase/synthase (*lcfB*) with ∼65% amino acid identity to *C. elegans* (*acs-1*), a mitochondria-related gene involved in lipid storage in embryo and larval development. However, the present function of this gene is unknown. The absence of introns in this gene suggest an early nematode-to-symbiont transfer that may be maintained in the symbiont due to an essential role either for the symbiont or the host. This gene is located directly adjacent to the phage-like putative protein translocation system (PLTS) along with numerous ATP-/transport-related genes, suggesting possible coordination of fatty-acid metabolic activities. This result is consistent with previous experiments implicating *Cardinium* in nematode lipid metabolism in the juvenile infective stage (Walsh et al., 1983b). Recent transcriptome analysis (Gardner et al., 2018) support ATP-binding and transport as important functions in this *Cardinium*. Connected with these functions we also uncovered an expanded family of 24 putative AAA-ATPases in cPpe appearing to have more homology with *Rickettsia* or *Wolbachia* than with outgroup Bacteroidetes. These genes likely encode proteins with either protein translocation (chaperone), proteolytic, or organelle biogenesis functions, and their abundance in cPpe as well as candidate HGT status, will make them a target for future analysis.

**FIGURE 11:**
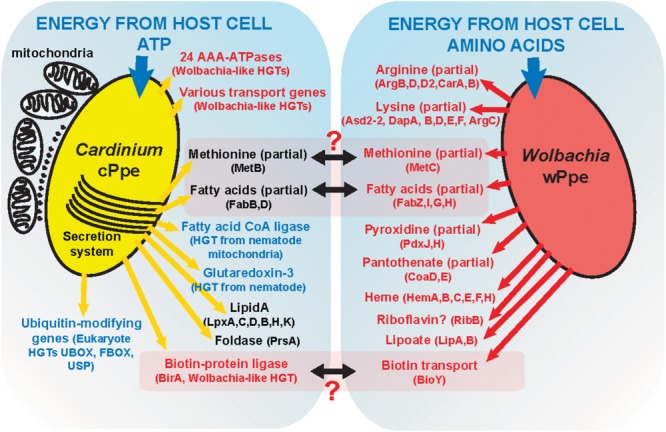
A model of possible interactions between *Cardinium* cPpe and *Wolbachia* wPpe in the PPN *P. penetrans*.

Metabolic biosynthetic pathways in *Cardinium* cPpe appear to be largely lacking, as has been shown for *Amoebophilus* (Schmitz-Esser et al., 2010) and insect *Cardinium* strains cBtQ1 (Santos-Garcia et al., 2014) and cEper1 (Penz et al., 2012). Furthermore, despite *Cardinium* cPpe having a larger genome than these insect strains, it appeared to have even more diminished biosynthetic capacity, with no evidence of biotin and thiamine operons, lipoate biosynthesis genes, or insect toxin genes. There was also no evidence of cPpe having a plasmid; a surprising finding giving the large size of the insect *Cardinium* plasmids, suggesting perhaps the latter arose later in this group. Notably, the whitefly *Cardinium* cBtQ1 plasmid hosts genes for gliding motility (Santos-Garcia et al., 2014) that may play a role in horizontal transmission through plant tissues (Gonella et al., 2015), but these genes are missing in cPpe, suggesting perhaps absence of this transmission route. Several partial pathways remain in cPpe and wPpe that might form partial complementarity (**Figure 11**).

Because both *Cardinium* and *Wolbachia* are thought to manipulate host cellular processes including reproduction using a set of eukaryote-derived protein domains, such as the ubiquitination-modulating proteins (Schmitz-Esser et al., 2010; Beckmann et al., 2017; Mann et al., 2017; White et al., 2017), we examined these in detail and found moderate abundance of these in cPpe, particularly FBOX domain genes expanded and unique USP homologous in cPpe, cBtQ1, and cEper1, with blast similarity to nematodes and algae. These domains are thought to modify ubiquitination, marking proteins for degradation or changing their interactions with other proteins. The FBOX is thought to regulate the SCF multi-protein E3 ubiquitin ligase complex targeting proteins for degradation and there has been significant attention on deubiquitylation as a critical component of not only successful endosymbiosis but also efficient CI (Beckmann et al., 2017; LePage et al., 2017).

Candidate HGTs were abundant in cPpe and wPpe and are consistent with the hypothesis of early endosymbiosis as a trading ground (Schmitz-Esser et al., 2010), emphasizing potential exchanges in multiple directions. Over 2% of cPpe and wPpe genes were not universally shared with other strains of *Cardinium* and *Wolbachia*, but instead represent candidate HGTs arising in the ancestry of these groups. For example, in wPpe there were two genes predicted to be involved in carotenoid synthesis with similarity to other bacteria (Brown et al., 2016). In cPpe there was a large toxin gene with high similarity to a glycosyl transferase in *R. insecticola*, a gammaproteobacterial symbiont shown to be protective in aphids (Vorburger et al., 2010). While candidate HGTs in cPpe also included potentially *Rickettsia*-derived multidrug export ATP-binding/permease and biotin-protein ligase, no biotin (vitamin B_7_) biosynthesis genes were found in either cPpe or wPpe. Acquisition of biotin and thiamine synthesis, and other B vitamins, is a common feature among symbionts of blood feeders and other specialists, such as cEper1, but the biotin operon appears partially lost in cBtQ1 on which *Encarsia* is parasitic (Penz et al., 2012; Santos-Garcia et al., 2014). Similarly, cEper1 and cBtQ1 have vitamin B_6_ biosynthesis genes, *pdxS* and *pdxT* and *pdxT* resulting perhaps from HGT from other bacteria, but these were absent in cPpe. However, cPpe appears to possess many ankyrin-repeat containing genes and ADP/ATP carrier proteins with similarity to those in *Wolbachia* and eukaryotes, and a mitochondrial carrier protein with similarity to *Neorickettsia*, and a calcium-binding mitochondrial carrier protein with similarity to those of eukaryotes. Similar HGTs have been implicated in the amoeba symbiont *Legionella* (Dolezal et al., 2012). In contrast, wPpe has candidate HGTs for amino acid metabolism, such as phenylalanine degradation, histidine and lysine biosynthesis, among others. This is consistent with data suggesting *Wolbachia* may use amino acids as a primary energy source (Ju et al., 2017). Together, these results support the idea that HGTs underlie success in these endosymbionts.

Gene expansions tend to be reduced in endosymbionts, but numerous gene expansions were noted in *Amoebophilus* (Schmitz-Esser et al., 2010) and are of interest especially in cases where they are associated with changes in gene function through insertion sequence (IS) element lateral transfer (Duron, 2013). This process is particularly important in coinfecting symbioses. Two sources of lateral transfer widely established in these endosymbionts are the WO prophage of *Wolbachia* and the large plasmid of *Cardinium* (similarly *Rickettsia* plasmids). However, in our study there was no evidence of either WO phage in wPpe nor plasmids in cPpe. This may be the reason for limited evidence of recent horizontal gene exchange between cPpe and wPpe. However, expansions in cPpe included transposases such as the transposase gene 01133, which had highest blast similarity to Rickettsia/*Wolbachia*, along with flanking genes 01130 and 01131. These and other low-similarity, presumably ancient, transfers will require further characterization, such as gene expression analyses, to assess their possible role. Among the expansions of most interest in such studies will be the 24-copy alphaproteobacteria-like AAA-ATPases that may be involved in protein modification or translocation, one of which is associated with the antifeeding phage tail-derived protein secretion (PTPS), and the 18-copy FBOX-containing *mutM*-like gene that may have a role in communication with the host. Interestingly, both of these gene families appeared to be uniquely expanded in cPpe compared with other *Cardinium* strains.

*Cardinium* strains lack typical protein secretion systems (Schmitz-Esser et al., 2010; Penz et al., 2012; Santos-Garcia et al., 2014), but instead rely on the prophage-derived PTPS system, also denoted T6SS^iv^ (for its distant relationship with Type VI secretion systems). These are thought to arise from an ancient horizontal gene transfer (Heymann et al., 2013; Sarris et al., 2014) and to be a part of the TEM-visible “tubule” or “fibril” network characteristic of this group (Penz et al., 2012). In our analysis it appears that cPpe retained most of the genes for this system in synteny, despite the long evolutionary distances, suggesting an essential role. We analyzed genes clustered near the PTPS and found most to be transport-related including two manganese transport gene homologs. This structure and possible secretory function will be an interesting focus for further study, particularly because of recent reports that T6SS systems may play a role in interbacterial competition or protection against fungi (Bernal et al., 2018).

Based on these data, we propose a model (**Figure 11**) of possible interactions of these endosymbionts in *P. penetrans*. Given that cPpe and wPpe appear to occupy the same tissues in the same host, we considered possible metabolic complementarity. As shown in **Figure 11**, only a few genes in methionine biosynthesis, fatty acid biosynthesis, and biotin uptake regulation appeared to potentially fit this pattern, but in all cases, the pathways remain incomplete. In future, further genomes will be needed to validate this model. Alternatively, these endosymbionts may have a fitness cost and may compete, as suggested by alternate abundance profiles between these two endosymbionts. Phylogenomic data suggest this co-occurrence may be ancient, placing greater interest still in how these endosymbionts might interact. In whiteflies, *Cardinium* cBtQ1 was suggested as a possible “hitchhiker,” benefiting metabolically from primary symbionts (Santos-Garcia et al., 2014), since their hosts have nutritional symbionts. Prevalence data and fatty acid synthesis in our system raise the question of whether *Wolbachia* could hitchhike on the symbiosis with *Cardinium*. Of note is the absence of plant cell-wall degrading enzymes in cPpe, suggesting absence of a role in promoting plant-parasitism.

Two unanswered questions that arise from this work are: (1) How widespread are these endosymbionts? and (2) what are their specific effects on host fitness? A priority in the near future will be to increase screening of PPNs for these endosymbionts. However, our data suggest these endosymbionts have large evolutionary distances and can occur as low-titer infections, which may reduce the efficacy of PCR-based surveys. Instead, a preferred approach may be high-throughput genome screening (Denver et al., 2016). Such an approach can also be used to screen plant tissues to explore the idea of horizontal transmission (Gonella et al., 2015). A greater challenge will be to effectively conduct manipulative experiments measuring fitness effects of antibiotic-cleared nematodes, which, for these migratory endoparasitic nematodes may present considerable challenges.

## Data Availability

The draft genome of *Cardinium* cPpe from *P. penetrans* is deposited in NCBI GenBank under project accession number PRJNA308318. Raw MiSeq data are available under NCBI SRA number SRR3097580.

## Author Contributions

AB, DD, and IZ designed the study. AB led the genome analyses and wrote the initial manuscript. Specimens were collected by IZ and AP. Genomic library preparation was performed by DH. PCR and FISH experiments were performed by AB and SW. The final manuscript was revised by all authors.

## Conflict of Interest Statement

The authors declare that the research was conducted in the absence of any commercial or financial relationships that could be construed as a potential conflict of interest.

## Footnotes

1 http://hannonlab.csht.edu/fastx_toolkit/
