# Supplementary material for: Comparative Genomics of Wolbachia–Cardinium Dual Endosymbiosis in a Plant-Parasitic Nematode
Source: Front Microbiol. 2018 Oct 16;9:2482. doi: 10.3389/fmicb.2018.02482 (PMC6232779; doi:10.3389/fmicb.2018.02482)
Supplement: Supplementary file 1 [file Data_Sheet_1.docx]

***Supplementary Material***

Comparative genomics of *Wolbachia*-*Cardinium* dual endosymbiosis in a plant-parasitic nematode

**Amanda M.V. Brown^*^, Sulochana K. Wasala, Dana K. Howe, Amy B. Peetz, Inga A. Zasada, Dee R. Denver**

*** Correspondence:** Amanda M.V. Brown: amanda.mv.brown@ttu.edu

**Supplementary Table 1.** PCR Primers and conditions for survey of *Cardinium* cPpe and *Wolbachia* in *P. penetrans*.

| Primer name | Sequence | Thermal cycle conditions | |
| --- | --- | --- | --- |
| Cardinium |  | One cycle:  36 cycles:  One cycle: | 95°C – 2 min  95°C – 30 sec  52°C – 20 sec  72°C – 2 min  72°C – 10 min |
| Car_281_F | 5’ GGT AGG GGT TCT TAG TGG AAG 3’ |  |  |
| Car_269_R | 5’ TGC TCC CCA CGC TTT CGT G 3’ |  |  |
| *Wolbachia** |  | PCR 1:  One cycle:  36 cycles:  One cycle:  PCR 2:  One cycle:  36 cycles:  One cycle: | 95°C – 2 min  95°C – 30 sec  52°C – 20 sec  72°C – 2 min  72°C – 10 min  95°C – 3 min  95°C – 30 sec  58°C – 20 sec  72°C – 1 min  72°C – 10 min |
| Wol_16S_F | 5’ GGT AGG GTA ATG GCT TAC CAA GG 3’ |  |  |
| Wol_16S_R | 5’ TTC ATC GTT TAC AGC GTG GA 3’ |  |  |
| Wol_281B_F | 5’ AAT GGC TTA CCA AGG YWA TGA TCT 3’ |  |  |
| Wol_692_R | 5’ TCC TCT TTC AAT CTC TAG ATT AG 3’ |  |  |
|  |  |  |  |

*This PCR was performed in two steps, the products from the first PCR reaction diluted 1:10 and used as a template for the second, more specific PCR.

**Supplementary Table 2.** Genes included in 37-gene phylogenomic analyses.

|  | Gene symbol | Product |
| --- | --- | --- |
|  | rpoB | DNA-directed RNA polymerase subunit beta |
|  | rpoC | DNA-directed RNA polymerase subunit beta' |
|  | fusA | Elongation factor G |
|  | infB | Translation initiation factor IF-2 |
|  | alaS | Alanine--tRNA ligase |
|  | argS | Arginine--tRNA ligase |
|  | cysS | Cysteine--tRNA ligase |
|  | glyS | Glycine--tRNA ligase subunit β |
|  | hisS | Histidine--tRNA ligase |
|  | ileS | Isoleucine--tRNA ligase |
|  | lysS | Lysine--tRNA ligase |
|  | metG | Methionine--tRNA ligase |
|  | pheT | Phenylalanine--tRNA ligase beta subunit |
|  | proS | Proline--tRNA ligase |
|  | serS | Serine--tRNA ligase |
|  | thrS | Threonine--tRNA ligase |
|  | valS | Valine--tRNA ligase |
|  | dnaK | Chaperone protein DnaK |
|  | ppiD | Periplasmic folding chaperone |
|  | cafA (rng) | RNase G |
|  | cca | Multifunctional CCA protein |
|  | rho | Transcription termination factor Rho |
|  | dnaG | DNA primase |
|  | gyrA | DNA gyrase subunit A |
|  | gyrB | DNA gyrase subunit B |
|  | topA | DNA topoisomerase 1 |
|  | gidA (mnmG) | tRNA uridine 5-carboxymethylaminomethyl mo |
|  | ligA | DNA ligase |
|  | mutL | DNA mismatch repair protein MutL |
|  | dnaE | DNA polymerase III subunit alpha |
|  | mutS | DNA mismatch repair protein MutS |
|  | polA | DNA polymerase I |
|  | engA (der) | 50S ribosomal subunit stability factor |
|  | miaB | Isopentenyl-adenosine A37 tRNA methylthiolase |
|  | nusA | Transcription termination/antitermination protein NusA |
|  | tilS | tRNA(Ile)-lysidine synthase |
|  | trmE (mnmE) | 5-carboxymethylaminomethyluridine-tRNA synthase GTPase subunit |

**Supplementary Table 3.** Assembly details for *Cardinium* cPpe.

|  | Feature |  |
| --- | --- | --- |
|  | # Reads (Raw) | 19,164,652 |
|  | Read length | 301 |
|  | # bp (lengthX#reads) | 5,768,560,252 |
|  | # Scaffolds (total assembly) | 807,072 |
|  | N50 (total assembly) | 5,531 |
|  | Max. scaffold length | 1,697,130 |
|  | Sum of scaffold lengths (total assembly) | 349,666,756 |
|  | # Scaffolds matching *Cardinium* | 27 |
|  | N50 (matching *Cardinium*) | 163,560 |
|  | Coverage (*Cardinium* scaffolds) | 15.01X |
|  | Max. scaffold length (matching *Cardinium*) | 241,819 |
|  | Sum of scaffold lengths (matching *Cardinium*) | 1,358,214 |

**Supplementary Table 4.** Species, habitat, and genome features, and accession numbers for *Cardinium* and related Bacteroidetes that were compared in this study. c = complete; wgs = whole genome shotgun sequencing.

|  | Species or strain | Habitat (and host) | Genome size | Predicted Proteins | %GC | rRNA | tRNA | Prop. coding | Orth. length | Accession | Data status |
| --- | --- | --- | --- | --- | --- | --- | --- | --- | --- | --- | --- |
|  | *Spirosoma linguale* | freshwater/soil | 8,078,757 | 6,524 | 50.2 | 11 | 46 | 0.89 | 80,461 | CP001769 | c |
|  | *Dyadobacter fermentans* | soil/roots | 6,967,790 | 5,697 | 51.5 | 12 | 38 | 0.91 | 78,692 | NC_013037 | c |
|  | *Runella slithyformis* | fresh water | 6,568,739 | 5,415 | 46.6 | 6 | 42 | 0.89 | 79,540 | NC_015703 | c |
|  | *Cyclobacterium marinum* | coelomic fluid of sand dollar | 6,221,273 | 4,942 | 38.1 | 9 | 39 | 0.86 | 79,354 | NC_015914.1 | c |
|  | *Flavobacterium johnsoniae* | freshwater/soil | 6,096,872 | 5,099 | 34.1 | 18 | 60 | 0.88 | 79,642 | NC_009441 | c |
|  | *Marivirga tractuosa* | beach sand | 4,511,574 | 3,721 | 35.3 | 6 | 39 | 0.89 | 78,547 | NC_014759 | c |
|  | *Cytophaga hutchinsonii* | free-living, cellulolytic | 4,433,218 | 3,662 | 38.8 | 9 | 38 | 0.90 | 83,176 | NC_008255 | c |
|  | *Leadbetterella byssophilia* | soil/compost | 4,059,653 | 3,469 | 40.4 | 9 | 41 | 0.92 | 77,434 | NC_014655 | c |
|  | *Ca. Amoebophilus asiaticus* | endosymbiont of *Acanthamoebae* sp. TUMSJ-321 | 1,884,364 | 1,394 | 35.0 | 3 | 35 | 0.77 | 76,321 | NC_010830 | c |
|  | *Ca. Cardinium* cPpe | endosymbiont of *Pratylenchus penetrans* | 1,358,214 | 1,131 | 35.8 | 3 | 35 | 0.79 | 72,925 | This Study | wgs |
|  | *Ca. Cardinium* cBtQ1 | endosymbiont of *Bemisia tabaci* | 1,012,588 | 869 | 36.1 | 3 | 35 | 0.73 | 74,350 | NZ_CBQZ0-00000000 | wgs |
|  | *Ca. Cardinium* cEper1 | endosymbiont of *Encarsia pergandiella* | 887,130 | 710 | 36.6 | 3 | 37 | 0.86 | 74,104 | NC_018605 | c |
|  | *S. linguale* plasmid 1 | freshwater/soil | 189,452 | 177 | 49.2 | - | - | 0.88 | - | CP00770.1 | c |
|  | *S. linguale* plasmid 2 | freshwater/soil | 146,936 | 142 | 51.3 | - | - | 0.89 | - | CP00771.1 | c |
|  | *R. slithyformis* plasmid 1 | fresh water | 106,999 | 78 | 46.3 | - | - | 0.87 | - | NC_015693 | c |
|  | *R. slithyformis* plasmid 2 | fresh water | 93,527 | 90 | 41.4 | - | - | 0.94 | - | NC_015704 | c |
|  | *R. slithyformis* plasmid 3 | fresh water | 66,926 | 62 | 40.8 | - | - | 0.87 | - | NC_015694 | c |
|  | *Cardinium* cEper1 pCher | endosymbiont of *E. pergandiella* | 57,800 | 51 | 31.5 | - | - | 0.81 | - | NC_018606 | c |
|  | *Cardinium* cBtQ1 pCHV | Endosymbiont of *B. tabaci* | 52,050 | 30 | 31.9 | - | - | 0.86 | - | HG422566 | c |
|  | *R. slithyformis* plasmid 4 | fresh water | 44,754 | 41 | 43.2 | - | - | 0.84 | - | NC_015705 | c |
|  | *R. slithyformis* plasmid 5 | fresh water | 38,784 | 32 | 44.2 | - | - | 0.88 | - | NC_015695 | c |
|  | *S. linguale* plasmid 3 | freshwater/soil | 36,434 | 40 | 44.3 | - | - | 0.71 | - | CP00772.1 | c |
|  | *S. linguale* plasmid 4 | freshwater/soil | 9,965 | 14 | 47.8 | - | - | 0.73 | - | CP00773.1 | c |
|  | *S. linguale* plasmid 5 | freshwater/soil | 8,651 | 12 | 44.2 | - | - | 0.60 | - | CP00774.1 | c |
|  | *S. linguale* plasmid 6 | freshwater/soil | 7,683 | 10 | 47.3 | - | - | 0.77 | - | CP00775.1 | c |
|  | *S. linguale* plasmid 7 | freshwater/soil | 7,308 | 11 | 47.6 | - | - | 0.75 | - | CP00776.1 | c |
|  | *S. linguale* plasmid 8 | freshwater/soil | 6,072 | 8 | 48.4 | - | - | 0.77 | - | CP00777.1 | c |
|  | *M. tractuosa* plasmid | beach sand | 4,916 | 8 | 39.7 | - | - | 0.77 | - | NC_014750 | c |

**Supplementary Table 5.** List of genes with multiple copies or paralogs in *Cardinium* strains and *Amoebophilus asiaticus*.

| **Amoebophilus** | **#** | **cEper1** | **#** | **cBtQ1** | **#** | | **cPpe** | **#** | |
| --- | --- | --- | --- | --- | --- | --- | --- | --- | --- |
| Transposase DDE domain protein | 74 | Transposase, Mutator family | 9 | Transposase DDE domain protein | 25 | | Transposase DDE domain protein | 46 | |
| hcpD | 51 | Integrase core domain protein | 8 | hypothetical protein | 14 | | putative AAA-ATPase | 24 | |
| Transposase DDE domain protein | 36 | Sodium/proline symporter | 5 | hypothetical protein | 14 | | mutM | 18 | |
| cytR | 30 | Integrase core domain protein | 5 | hypothetical protein | 13 | | hypothetical protein | 13 | |
| IS1 transposase | 26 | flagellar assembly protein H | 4 | hypothetical protein | 7 | | putative transposase | 12 | |
| Integrase core domain protein | 25 | Transposase DDE domain protein | 4 | hypothetical protein | 7 | | hypothetical protein | 12 | |
| mutM | 24 | Transposase, Mutator family | 4 | hypothetical protein | 7 | | Sodium/proline symporter | 11 | |
| engB | 21 | hcpC | 3 | hcpC | 5 | | Leucine Rich repeats (2 copies) | 10 | |
| spoT | 18 | spoT | 3 | Transposase DDE domain protein | 5 | | lyc | 8 | |
| hypothetical protein | 17 | Integrase core domain protein | 3 | ltrA | 5 | | hypothetical protein | 8 | |
| hcpC | 16 | hypothetical protein | 3 | hypothetical protein | 5 | | spoT | 7 | |
| hypothetical protein | 16 | hypothetical protein | 3 | hypothetical protein | 5 | | spa | 7 | |
| hypothetical protein | 16 | Integrase core domain protein | 3 | hlyB | 4 | | hypothetical protein | 7 | |
| Transposase DDE domain protein | 16 | lspA | 2 | Sodium/proline symporter | 4 | | Transposase DDE domain protein | 6 | |
| Transposase DDE domain protein | 12 | tadA | 2 | flagellar assembly protein H | 4 | | Transposase DDE domain protein | 6 | |
| Transposase IS116 IS110 IS902 family prote | 11 | PD-(D E)XK nuclease family transposase | 2 | Integrase core domain protein | 4 | | hypothetical protein | 6 | |
| hypothetical protein | 11 | Transposase DDE domain protein | 2 | ankX | 4 | | hypothetical protein | 6 | |
| hypothetical protein | 9 | troA | 2 | hypothetical protein | 4 | | inlJ | 5 | |
| rrrD | 8 | Transposase | 2 | Transposase DDE domain protein | 4 | | ankX | 5 | |
| Transposase IS200 like protein | 8 | tlcA | 2 | hypothetical protein | 4 | | Transposase DDE domain protein | 5 | |
| Transposase DDE domain protein | 7 | putative transporter | 2 | Transposase IS66 family protein | 4 | | Bifunctional (p)ppGpp synthase hydrolase R | 5 | |
| Transposase DDE domain protein | 6 | SMI1 KNR4 family protein | 2 | Transposase, Mutator family | 3 | | hypothetical protein | 5 | |
| Integrase core domain protein | 5 | hypothetical protein | 2 | hypothetical protein | 3 | | SET domain protein | 5 | |
| hypothetical protein | 5 | hypothetical protein | 2 | hypothetical protein | 3 | | hypothetical protein | 5 | |
| hypothetical protein | 4 |  |  | Transposase DDE domain protein | 3 | | Ankyrinrepeats (3 copies) | 4 | |
| hypothetical protein | 4 |  |  | hypothetical protein | 3 | | Ankyrinrepeats (3 copies) | 4 | |
| ankX | 4 |  |  | hypothetical protein | 3 | | hypothetical protein | 4 | |
| hypothetical protein | 4 |  |  | spoT | 2 | | Ankyrinrepeats (3 copies) | 4 | |
| hypothetical protein | 4 |  |  | Transposase, Mutator family | 2 | | hypothetical protein | 4 | |
| clpC | 3 |  |  | hypothetical protein | 2 | | hypothetical protein | 4 | |
| bvgS | 3 |  |  | lspA | 2 | | ykfA | 3 | |
| hypothetical protein | 3 |  |  | Putative 1,2-phenylacetyl-CoA epoxidase, s | 2 | | DNA polymerase III subunit delta | 3 | |
| Integrase core domain protein | 3 |  |  | ruvC | 2 | | cca | 3 | |
| Bacterial regulatory protein, Fis family | 3 |  |  | tadA | 2 | | priA | 3 | |
| Patatin-like phospholipase | 3 |  |  | recG | 2 | | gpsA | 3 | |
| hypothetical protein | 3 |  |  | glyQS | 2 | | dnaQ | 3 | |
| hypothetical protein | 3 |  |  | mreB | 2 | | PD-(D E)XK nuclease family transposase | 3 | |
| hypothetical protein | 3 |  |  | spoIIIE | 2 | | hypothetical protein | 3 | |
| Sodium glucose cotransporter | 3 |  |  | lpxH | 2 | | Alpha beta hydrolase family protein | 3 | |
| hypothetical protein | 3 |  |  | troA | 2 | | hypothetical protein | 3 | |
| hypothetical protein | 3 |  |  | tlcA | 2 | | hypothetical protein | 3 | |
| Ankyrin repeat protein | 3 |  |  | putative transporter | 2 | | hypothetical protein | 3 | |
| hypothetical protein | 2 |  |  | Ankyrin repeat protein | 2 | | Alpha beta hydrolase family protein | 3 | |
| tnpR | 2 |  |  | hypothetical protein | 2 | | hypothetical protein | 3 | |
| xerC | 2 |  |  | Sodium:solute symporter family protein | 2 | | hypothetical protein | 3 | |
| uvrC | 2 |  |  | hypothetical protein | 2 | | hypothetical protein | 3 | |
| hypothetical protein | 2 |  |  | Transposase DDE domain protein | 2 | | Transposase DDE domain protein | 2 | |
| Colicin V production protein | 2 |  |  | hypothetical protein | 2 | | hcpC | 2 | |
| Putative multidrug export ATP-binding permease | 2 |  |  | hypothetical protein | 2 | | Integrase core domain protein | 2 | |
| Helix-hairpin-helix motif protein | 2 |  |  | hypothetical protein | 2 | | Transposase, Mutator family | 2 | |
| oppD | 2 |  |  | Transposase IS66 family protein | 2 | | flagellar assembly protein H | 2 | |
| PD-(D E)XK nuclease superfamily protein | 2 |  |  | Outer membrane efflux protein | 2 | | lepB | 2 | |
| putative metallophosphoesterase | 2 |  |  | hlyD | 2 | | lspA | 2 | |
| yhhQ | 2 |  |  | hypothetical protein | 2 | | Putative 1,2-phenylacetyl-CoA epoxidase, s | 2 | |
| Di- tripeptide transporter | 2 |  |  | hypothetical protein | 2 | | uvrD | 2 | |
| WbqC-like protein family protein | 2 |  |  |  |  | | dnaE | 2 | |
| pyk | 2 |  |  |  |  | | htrB | 2 | |
| rnhB | 2 |  |  |  |  | | dnaB | 2 | |
| pld | 2 |  |  |  |  | | rpoN | 2 | |
| yoeB | 2 |  |  |  |  | | ruvC | 2 | |
| gsiC | 2 |  |  |  |  | | murA | 2 | |
| hbpA | 2 |  |  |  |  | | birA | 2 | |
| mutS | 2 |  |  |  |  | | Putative zinc metalloprotease | 2 | |
| dppB | 2 |  |  |  |  | | glgA | 2 | |
| puuB | 2 |  |  |  |  | | ftsW | 2 | |
| oppD | 2 |  |  |  |  | | hypothetical protein | 2 | |
| slrP | 2 |  |  |  |  | | trmD | 2 | |
| hypothetical protein | 2 |  |  |  |  | | putative permease YjgP YjgQ family protein | 2 | |
| hypothetical protein | 2 |  |  |  |  | | trpS | 2 | |
| Bifunctional (p)ppGpp synthase hydrolase r | 2 |  |  |  |  | | hypothetical protein | 2 | |
| hypothetical protein | 2 |  |  |  |  | | gltX | 2 | |
| hypothetical protein | 2 |  |  |  |  | | psd | 2 | |
| hcpC | 2 |  |  |  |  | | polA | 2 | |
| hypothetical protein | 2 |  |  |  |  | | bamA | 2 | |
| putative lyase | 2 |  |  |  |  | | queA | 2 | |
| hypothetical protein | 2 |  |  |  |  | | hypothetical protein | 2 | |
| hypothetical protein | 2 |  |  |  |  | | fus | 2 | |
| Ubiquitin carboxyl-terminal hydrolase | 2 |  |  |  |  | | rpsG | 2 | |
| hypothetical protein | 2 |  |  |  |  | | asnS | 2 | |
| ankX | 2 |  |  |  |  | | atpG | 2 | |
| hypothetical protein | 2 |  |  |  |  | | atpA | 2 | |
| hypothetical protein | 2 |  |  |  |  | | dnaG | 2 | |
| hypothetical protein | 2 |  |  |  |  | | leuS | 2 | |
| Ankyrinrepeats (3 copies) | 2 |  |  |  |  | | metN | 2 | |
| Stress-induced bacterial acidophilic repea | 2 |  |  |  |  | | mlaE | 2 | |
|  |  |  |  |  |  | | cdsA | 2 | |
|  |  |  |  |  |  | | norM | 2 | |
|  |  |  |  |  |  | | metal-dependent hydrolase | 2 | |
|  |  |  |  |  |  | | alkA | 2 | |
|  |  |  |  |  |  | | dut | 2 | |
|  |  |  |  |  |  | | mutS | 2 | |
|  |  |  |  |  |  | | T4-like virus tail tube protein gp19 | 2 | |
|  |  |  |  |  |  | | clpP | 2 | |
|  |  |  |  |  |  | | hypothetical protein | 2 | |
|  |  |  |  |  |  | | hypothetical protein | 2 | |
|  |  |  |  |  |  | | hypothetical protein | 2 | |
|  |  |  |  |  |  | | hypothetical protein | 2 | |
|  |  |  |  |  |  | | putative hydrolase | 2 | |
|  |  |  |  |  |  | | tlcA | 2 | |
|  |  |  |  |  |  | | Ubiquitin carboxyl-terminal hydrolase | 2 | |
|  |  |  |  |  |  | | hypothetical protein | 2 | |
|  |  |  |  |  |  | | Bacterial leucyl aminopeptidase precursor | 2 | |
|  |  |  |  |  |  | | nfo | 2 | |
|  |  |  |  |  |  | | spsI | 2 | |
|  |  |  |  |  |  | | UMP phosphatase | 2 | |
|  |  |  |  |  |  | | hypothetical protein | 2 | |
|  |  |  |  |  |  | | hypothetical protein | 2 | |
|  |  |  |  |  |  | | hypothetical protein | 2 | |
|  |  |  |  |  |  | | hypothetical protein | 2 | |
|  |  |  |  |  |  | | hypothetical protein | 2 | |
|  |  |  |  |  |  | | hypothetical protein | 2 | |
|  |  |  |  |  |  | | hypothetical protein | 2 | |
|  |  |  |  |  |  | | hypothetical protein | 2 | |
|  |  |  |  |  |  | | hypothetical protein | 2 | |
|  |  |  |  |  |  | | hypothetical protein | 2 | |
|  |  |  |  |  |  | | hypothetical protein | 2 | |
|  |  |  |  |  |  | | matE | 2 | |
|  |  |  |  |  |  | | PD-(D E)XK nuclease family transposase | 2 | |
|  |  |  |  |  |  | | OPT oligopeptide transporter protein | 2 | |
|  |  |  |  |  |  | | Ankyrinrepeats (3 copies) | 2 | |
|  |  |  |  |  |  | | hypothetical protein | 2 | |
|  |  |  |  |  |  | | hypothetical protein | 2 | |
|  |  |  |  |  |  | | hypothetical protein | 2 | |
|  |  |  |  |  |  | | hypothetical protein | 2 | |
|  |  |  |  |  |  | | mnaA | 2 | |
|  |  |  |  |  |  | | gltX2 | 2 | |
|  |  |  |  |  |  | | Ankyrinrepeats (3 copies) | 2 | |
|  |  |  |  |  |  | | hypothetical protein | 2 | |
|  |  |  |  |  |  | | hypothetical protein | 2 | |
|  |  |  |  |  |  | | hypothetical protein | 2 | |
|  |  |  |  |  |  | | Zinc finger, C3H C4 type (RING finger) | 2 | |
|  |  |  |  |  |  | | hypothetical protein | 2 | |
|  |  |  |  |  |  | | 2-phosphosulfolactate phosphatase | 2 | |
|  |  |  |  |  |  | | ankX | 2 | |
|  |  |  |  |  |  | | hypothetical protein | 2 | |
|  |  |  |  |  |  | | Phosphatidylcholine-sterol acyltransferase precursor | 2 | |
|  |  |  |  |  |  | | hypothetical protein | 2 | |
|  |  |  |  |  |  | | hypothetical protein | 2 | |
|  |  |  |  |  |  | | hypothetical protein | 2 | |
|  |  |  |  |  |  | | Leucine Rich repeats (2 copies) | 2 | |
|  |  |  |  |  |  | hypothetical protein | | | 2 |

**Supplementary Table 7.** Gene names for genes in ortholog groups from Figure 6A. CarP = *Cardinium* cPpe from *Pratylenchus penetrans*; CarB = *Cardinium* cBtQ1; CarE = *Cardinium* cEper1.

| Venn Grouping | Number | Group Name | Gene Name |
| --- | --- | --- | --- |
| CarB CarE CarP | 503 | CarP4776: | asnS |
|  |  | CarP1329: | hypothetical protein |
|  |  | CarP1139: | trpS |
|  |  | CarP1476: | murB |
|  |  | CarP1492: | nusG |
|  |  | CarP3628: | hypothetical protein |
|  |  | CarP2616: | plsB |
|  |  | CarP1541: | atpE |
|  |  | CarP1451: | tig |
|  |  | CarP1464: | lysS |
|  |  | CarP1364: | mltD |
|  |  | CarP1310: | ybeY |
|  |  | CarP1232: | tsf |
|  |  | CarP1194: | cdsA |
|  |  | CarP1171: | nifA |
|  |  | CarP1839: | rnhA |
|  |  | CarP4779: | tmk |
|  |  | CarP1252: | tsaB |
|  |  | CarP1412: | prsA |
|  |  | CarP3631: | nfo |
|  |  | CarP4768: | Alpha/beta hydrolase family protein |
|  |  | CarP4761: | ybhL |
|  |  | CarP1282: | rplN |
|  |  | CarP1449: | clpA |
|  |  | CarP3119: | hypothetical protein |
|  |  | CarP2027: | greA |
|  |  | CarP1254: | nusA |
|  |  | CarP1484: | prkC |
|  |  | CarP1249: | rplU |
|  |  | CarP1271: | infA |
|  |  | CarP1447: | sufB |
|  |  | CarP1248: | Cell division protein ZapA |
|  |  | CarP1421: | epsM |
|  |  | CarP1243: | CDP-alcohol phosphatidyltransferase |
|  |  | CarP1375: | murE |
|  |  | CarP1299: | rbfA |
|  |  | CarP1438: | murG |
|  |  | CarP1253: | rpsJ |
|  |  | CarP3127: | hypothetical protein |
|  |  | CarP1096: | Putative 1,2-phenylacetyl-CoA epoxidase, s |
|  |  | CarP1026: | clpC |
|  |  | CarP3626: | Ubiquitin carboxyl-terminal hydrolase |
|  |  | CarP1134: | ftsW |
|  |  | CarP1145: | queA |
|  |  | CarP1234: | rpsI |
|  |  | CarP1583: | ppa |
|  |  | CarP1135: | hypothetical protein |
|  |  | CarP1220: | rpsO |
|  |  | CarP1397: | Helix-hairpin-helix motif protein |
|  |  | CarP1298: | sufD |
|  |  | CarP1455: | lptD |
|  |  | CarP1037: | Sodium/proline symporter |
|  |  | CarP1452: | relA |
|  |  | CarP1100: | dnaE |
|  |  | CarP1130: | murA |
|  |  | CarP1103: | htrB |
|  |  | CarP2987: | hypothetical protein |
|  |  | CarP1372: | tyrS |
|  |  | CarP1353: | tetratricopeptide repeat protein |
|  |  | CarP1042: | fabG |
|  |  | CarP3625: | tlcA |
|  |  | CarP1286: | rpsC |
|  |  | CarP1221: | pnp |
|  |  | CarP1474: | recA |
|  |  | CarP1457: | mnmA |
|  |  | CarP4777: | Mitochondrial carrier protein |
|  |  | CarP1291: | rplD |
|  |  | CarP1287: | rplV |
|  |  | CarP1399: | Rhomboid family protein |
|  |  | CarP4762: | anaerobic C4-dicarboxylate transporter |
|  |  | CarP1308: | ptrA |
|  |  | CarP1235: | rplM |
|  |  | CarP4745: | mutS |
|  |  | CarP1263: | yfhQ |
|  |  | CarP1377: | ftsL |
|  |  | CarP1477: | hypothetical protein |
|  |  | CarP2623: | mntB |
|  |  | CarP3142: | macB |
|  |  | CarP1470: | metG |
|  |  | CarP1219: | dnaA |
|  |  | CarP2973: | oppD |
|  |  | CarP1238: | atpB |
|  |  | CarP3797: | hypothetical protein |
|  |  | CarP4766: | hypothetical protein |
|  |  | CarP1398: | rluA |
|  |  | CarP3833: | spoIVFB |
|  |  | CarP1092: | lspA |
|  |  | CarP1340: | smc |
|  |  | CarP1446: | der |
|  |  | CarP1158: | periplasmic chaperone |
|  |  | CarP1188: | PD-(D E)XK nuclease superfamily protein |
|  |  | CarP1564: | mce related protein |
|  |  | CarP1439: | gyrB |
|  |  | CarP1418: | gyrA |
|  |  | CarP2627: | Polymer-forming cytoskeletal |
|  |  | CarP1151: | atpA |
|  |  | CarP1559: | rpmB |
|  |  | CarP1165: | truA |
|  |  | CarP1352: | rplY |
|  |  | CarP1227: | queuine tRNA-ribosyltransferase |
|  |  | CarP1560: | hypothetical protein |
|  |  | CarP1333: | rpsR |
|  |  | CarP1351: | dnaK |
|  |  | CarP1354: | acoA |
|  |  | CarP1335: | YtxH-like protein |
|  |  | CarP1441: | acpS |
|  |  | CarP1193: | mlaE |
|  |  | CarP1362: | rpmI |
|  |  | CarP1264: | ssb |
|  |  | CarP2640: | hypothetical protein |
|  |  | CarP1146: | hypothetical protein |
|  |  | CarP1321: | groS5 |
|  |  | CarP1330: | ychF |
|  |  | CarP1392: | miaA |
|  |  | CarP1542: | bcp |
|  |  | CarP1393: | sppA |
|  |  | CarP2984: | hypothetical protein |
|  |  | CarP1491: | rplK |
|  |  | CarP1070: | lepB |
|  |  | CarP1324: | maeB |
|  |  | CarP1496: | recJ |
|  |  | CarP1236: | exbD |
|  |  | CarP1367: | ftsY |
|  |  | CarP1143: | DNA polymerase I |
|  |  | CarP1694: | dut |
|  |  | CarP1312: | lpxA |
|  |  | CarP3820: | hypothetical protein |
|  |  | CarP2630: | hypothetical protein |
|  |  | CarP1664: | Acyltransferase |
|  |  | CarP1322: | accA |
|  |  | CarP1381: | hypothetical protein |
|  |  | CarP1228: | putative permease YjgP YjgQ family protein |
|  |  | CarP1225: | prmB |
|  |  | CarP1305: | mfd |
|  |  | CarP1131: | birA |
|  |  | CarP2981: | clpP |
|  |  | CarP1919: | gsiC |
|  |  | CarP1021: | putative AAA-ATPase |
|  |  | CarP1405: | HIT-like protein |
|  |  | CarP4780: | Outer membrane efflux protein |
|  |  | CarP1378: | argS |
|  |  | CarP4763: | hypothetical protein |
|  |  | CarP4759: | CAAX amino terminal protease self- immunity |
|  |  | CarP3788: | dcuA |
|  |  | CarP1101: | priA |
|  |  | CarP1365: | rpmG |
|  |  | CarP3842: | macA |
|  |  | CarP1059: | flagellar assembly protein H |
|  |  | CarP1360: | thrS |
|  |  | CarP2641: | Leucine permease transcriptional regulator helical domain protein |
|  |  | CarP1400: | prfA |
|  |  | CarP1448: | mutL |
|  |  | CarP1270: | rpsM |
|  |  | CarP1132: | Putative zinc metalloprotease |
|  |  | CarP1317: | dhfrIII |
|  |  | CarP1567: | disA |
|  |  | CarP1444: | hypothetical protein |
|  |  | CarP2029: | pepA |
|  |  | CarP1085: | lyc |
|  |  | CarP1386: | gatA |
|  |  | CarP1172: | hypothetical protein |
|  |  | CarP1120: | macB |
|  |  | CarP1497: | lipoprotein chaperone |
|  |  | CarP1357: | Peptidase M16 inactive domain protein |
|  |  | CarP1246: | rpsT |
|  |  | CarP1294: | tadA |
|  |  | CarP1266: | rplQ |
|  |  | CarP1336: | N utilization substance protein B homolog |
|  |  | CarP1390: | hypothetical protein |
|  |  | CarP1304: | cgtA |
|  |  | CarP4754: | hypothetical protein |
|  |  | CarP4749: | tlcA |
|  |  | CarP1350: | lptC |
|  |  | CarP1584: | ribonuclease P |
|  |  | CarP1093: | dmpP |
|  |  | CarP1323: | dnaX |
|  |  | CarP1481: | frr |
|  |  | CarP1262: | hup |
|  |  | CarP1602: | alkA |
|  |  | CarP1128: | mreB |
|  |  | CarP1224: | iscS |
|  |  | CarP1255: | infB |
|  |  | CarP4767: | ppdK |
|  |  | CarP1389: | rpsA |
|  |  | CarP1396: | lrp |
|  |  | CarP1157: | efp |
|  |  | CarP1931: | T4-like virus tail tube protein gp19 |
|  |  | CarP4781: | macB |
|  |  | CarP1431: | putative ABC transporter ATP-binding protein |
|  |  | CarP4784: | Bacterial regulatory protein, Fis family |
|  |  | CarP4769: | hypothetical protein |
|  |  | CarP1068: | bacA |
|  |  | CarP1557: | PAP2 superfamily protein |
|  |  | CarP1461: | murF |
|  |  | CarP1426: | grpE |
|  |  | CarP1226: | ppx |
|  |  | CarP1500: | hypothetical protein |
|  |  | CarP1283: | rpsQ |
|  |  | CarP1116: | fadB |
|  |  | CarP3832: | Putative reductase/y4119/YP 4011 |
|  |  | CarP1186: | oppD |
|  |  | CarP1586: | ksgA |
|  |  | CarP1373: | comM |
|  |  | CarP1024: | trxA |
|  |  | CarP1222: | sigA |
|  |  | CarP1118: | ideR |
|  |  | CarP1119: | tadA |
|  |  | CarP2019: | Phage tail sheath protein |
|  |  | CarP1478: | hypothetical protein |
|  |  | CarP1543: | rpsL |
|  |  | CarP4765: | hypothetical protein |
|  |  | CarP3819: | hypothetical protein |
|  |  | CarP3622: | putative hydrolase |
|  |  | CarP3620: | hypothetical protein |
|  |  | CarP1267: | rpoA |
|  |  | CarP1295: | ftsH |
|  |  | CarP4774: | Major Facilitator Superfamily protein |
|  |  | CarP1341: | dksA |
|  |  | CarP1331: | tRNA pseudouridine synthase B |
|  |  | CarP1111: | fbp |
|  |  | CarP1277: | rplR |
|  |  | CarP1414: | secA |
|  |  | CarP1275: | rpmD |
|  |  | CarP1348: | ndk |
|  |  | CarP1265: | smpB |
|  |  | CarP1167: | sodA |
|  |  | CarP1326: | lolC |
|  |  | CarP1343: | ileS |
|  |  | CarP3800: | hypothetical protein |
|  |  | CarP1473: | rnr |
|  |  | CarP1498: | accA |
|  |  | CarP1129: | spoIIIE |
|  |  | CarP1229: | hslV |
|  |  | CarP1030: | putative transposase |
|  |  | CarP1385: | cysS |
|  |  | CarP1429: | iscS |
|  |  | CarP4772: | hypothetical protein |
|  |  | CarP1460: | acpP |
|  |  | CarP1359: | nrdA |
|  |  | CarP1576: | ATP-dependent helicase nuclease subunit A |
|  |  | CarP1475: | yqgF |
|  |  | CarP1409: | rpmE2 |
|  |  | CarP1347: | clpY |
|  |  | CarP3095: | hypothetical protein |
|  |  | CarP1233: | rpsB |
|  |  | CarP1044: | soj |
|  |  | CarP1382: | Non-canonical purine NTP pyrophosphatase |
|  |  | CarP1293: | fabD |
|  |  | CarP1487: | rpoB |
|  |  | CarP1268: | rpsD |
|  |  | CarP1074: | fabB |
|  |  | CarP1380: | secG |
|  |  | CarP1147: | fus |
|  |  | CarP1296: | yidC |
|  |  | CarP1358: | nrdB |
|  |  | CarP1416: | dus |
|  |  | CarP1342: | rluA |
|  |  | CarP1159: | fabB |
|  |  | CarP1797: | arnT |
|  |  | CarP1258: | Septum formation initiator |
|  |  | CarP2002: | Baseplate J-like protein |
|  |  | CarP1292: | rplC |
|  |  | CarP1570: | Peptidase family M3 |
|  |  | CarP1247: | pheT |
|  |  | CarP3809: | ypwA |
|  |  | CarP1156: | hypothetical protein |
|  |  | CarP4756: | hypothetical protein |
|  |  | CarP1284: | rpmC |
|  |  | CarP1922: | mutS |
|  |  | CarP1125: | Colicin V production protein |
|  |  | CarP1087: | hypothetical protein |
|  |  | CarP1285: | rplP |
|  |  | CarP1313: | lpxC |
|  |  | CarP1406: | greA |
|  |  | CarP1104: | dnaB |
|  |  | CarP1098: | cca |
|  |  | CarP1432: | atpC |
|  |  | CarP2517: | dppB |
|  |  | CarP1930: | Transposase DDE domain protein |
|  |  | CarP1580: | trmB |
|  |  | CarP1493: | elongation factor Tu |
|  |  | CarP1281: | rplX |
|  |  | CarP1334: | ung |
|  |  | CarP1302: | rho |
|  |  | CarP1376: | spoVD |
|  |  | CarP1430: | sufE |
|  |  | CarP1486: | rpoC |
|  |  | CarP1149: | asnS |
|  |  | CarP2985: | hypothetical protein |
|  |  | CarP1410: | lolD |
|  |  | CarP3789: | hypothetical protein |
|  |  | CarP1300: | mraY |
|  |  | CarP1251: | rpsP |
|  |  | CarP1404: | typA |
|  |  | CarP1142: | phosphatidylserine decarboxylase |
|  |  | CarP1366: | hypothetical protein |
|  |  | CarP1483: | yjjV |
|  |  | CarP1306: | gidA |
|  |  | CarP1445: | Putative TrmH family tRNA rRNA methyltransferase |
|  |  | CarP4782: | CAAX amino terminal protease self- immunity |
|  |  | CarP1346: | lon2 |
|  |  | CarP1489: | rplJ |
|  |  | CarP1178: | leuS |
|  |  | CarP1289: | rplB |
|  |  | CarP1290: | rplW |
|  |  | CarP1443: | Undecaprenyl-phosphate mannosyltransferase |
|  |  | CarP1407: | pth |
|  |  | CarP2552: | hypothetical protein |
|  |  | CarP1175: | ispU |
|  |  | CarP1337: | hypothetical protein |
|  |  | CarP1408: | lpxB |
|  |  | CarP1316: | uvrB |
|  |  | CarP1503: | hypothetical protein |
|  |  | CarP3825: | ribN |
|  |  | CarP4760: | hypothetical protein |
|  |  | CarP1279: | rpsH |
|  |  | CarP4770: | hypothetical protein |
|  |  | CarP1458: | mnmE |
|  |  | CarP1344: | ComEC family competence protein |
|  |  | CarP1415: | Sporulation related domain protein |
|  |  | CarP1437: | Nucleoid-associated protein |
|  |  | CarP1391: | gyrB |
|  |  | CarP3843: | lnt |
|  |  | CarP1328: | fdx |
|  |  | CarP1601: | Transposase DDE domain protein |
|  |  | CarP1471: | recQ |
|  |  | CarP1126: | recG |
|  |  | CarP1383: | engB |
|  |  | CarP1276: | rpsE |
|  |  | CarP1133: | glgA |
|  |  | CarP1309: | proS |
|  |  | CarP1105: | rpoN |
|  |  | CarP1424: | rplS |
|  |  | CarP1419: | minD |
|  |  | CarP4748: | hypothetical protein |
|  |  | CarP1261: | rng |
|  |  | CarP3805: | Putative multidrug export ATP-binding permease |
|  |  | CarP1138: | dnaQ |
|  |  | CarP1173: | lgt |
|  |  | CarP1368: | Aspartyl/glutamyl-tRNA(Asn/Gln) amidotransferase subunit B |
|  |  | CarP3818: | hypothetical protein |
|  |  | CarP1166: | xerD |
|  |  | CarP1499: | hypothetical protein |
|  |  | CarP1137: | putative permease YjgP YjgQ family protein |
|  |  | CarP1136: | trmD |
|  |  | CarP1278: | rplF |
|  |  | CarP1920: | hbpA |
|  |  | CarP1921: | Bacterial regulatory protein, Fis family |
|  |  | CarP1423: | lepA |
|  |  | CarP1325: | tsaB |
|  |  | CarP1546: | rpsN2 |
|  |  | CarP1433: | prfB |
|  |  | CarP2629: | hypothetical protein |
|  |  | CarP1071: | ykfA |
|  |  | CarP1244: | secD |
|  |  | CarP2637: | mntB |
|  |  | CarP1369: | alaS |
|  |  | CarP1338: | lpxK |
|  |  | CarP1099: | uvrD |
|  |  | CarP1411: | mutS |
|  |  | CarP1231: | PPIC-type PPIASE domain protein |
|  |  | CarP1256: | rpsU |
|  |  | CarP1394: | aviRb |
|  |  | CarP1485: | ligA |
|  |  | CarP1585: | metB |
|  |  | CarP4758: | hypothetical protein |
|  |  | CarP1401: | accB |
|  |  | CarP1425: | htpG |
|  |  | CarP2005: | nhaP2 |
|  |  | CarP2522: | Alpha beta hydrolase family protein |
|  |  | CarP1257: | alr |
|  |  | CarP1384: | degP |
|  |  | CarP1440: | era |
|  |  | CarP1060: | Transposase DDE domain protein |
|  |  | CarP1314: | lpxD |
|  |  | CarP1355: | mgtE |
|  |  | CarP1427: | dnaJ |
|  |  | CarP1490: | rplA |
|  |  | CarP2639: | Phage late control gene D protein (GPD) |
|  |  | CarP3837: | kgtP |
|  |  | CarP1239: | atpF |
|  |  | CarP1097: | DNA polymerase III subunit delta |
|  |  | CarP1356: | Receptor family ligand binding region |
|  |  | CarP1413: | Polysaccharide biosynthesis protein |
|  |  | CarP1482: | phosphodiesterase |
|  |  | CarP4747: | hypothetical protein |
|  |  | CarP1571: | fadD |
|  |  | CarP1168: | adenylate kinase |
|  |  | CarP1162: | ruvA |
|  |  | CarP1250: | rpmA |
|  |  | CarP1468: | valS |
|  |  | CarP2520: | tlcA |
|  |  | CarP2650: | citrate transporter |
|  |  | CarP1260: | DNA polymerase III subunit beta |
|  |  | CarP1272: | map |
|  |  | CarP1318: | hypothetical protein |
|  |  | CarP1169: | Putative multidrug export ATP-binding permease |
|  |  | CarP1395: | bamA |
|  |  | CarP1495: | lptB |
|  |  | CarP1402: | accC |
|  |  | CarP1332: | rplI |
|  |  | CarP2652: | rmuC |
|  |  | CarP1472: | gntX |
|  |  | CarP1688: | pld |
|  |  | CarP3629: | Bacterial leucyl aminopeptidase precursor |
|  |  | CarP1192: | metN |
|  |  | CarP4752: | hypothetical protein |
|  |  | CarP3798: | hypothetical protein |
|  |  | CarP1459: | rnc |
|  |  | CarP1327: | hypothetical protein |
|  |  | CarP1339: | ybgI |
|  |  | CarP1029: | uvrA |
|  |  | CarP1301: | hisS |
|  |  | CarP3138: | hypothetical protein |
|  |  | CarP1345: | ksgA |
|  |  | CarP1556: | murI |
|  |  | CarP1315: | ddlA |
|  |  | CarP1010: | hcpC |
|  |  | CarP3799: | hypothetical protein |
|  |  | CarP1545: | hypothetical protein |
|  |  | CarP1140: | hypothetical protein |
|  |  | CarP3829: | ddlB |
|  |  | CarP1307: | glyA |
|  |  | CarP1011: | Bifunctional (p)ppGpp synthase hydrolase R |
|  |  | CarP1456: | tilS |
|  |  | CarP1269: | rpsK |
|  |  | CarP1127: | glyQS |
|  |  | CarP3096: | tolB |
|  |  | CarP1573: | murD |
|  |  | CarP1363: | rplT |
|  |  | CarP1466: | ftsA |
|  |  | CarP1374: | rimM |
|  |  | CarP4764: | hypothetical protein |
|  |  | CarP1191: | lpxH |
|  |  | CarP1835: | troA |
|  |  | CarP1223: | trxB |
|  |  | CarP1578: | znuC |
|  |  | CarP1319: | parB |
|  |  | CarP1501: | ruvA |
|  |  | CarP1303: | serS |
|  |  | CarP1320: | groEL |
|  |  | CarP1388: | lptD |
|  |  | CarP1237: | tolQ |
|  |  | CarP3120: | hypothetical protein |
|  |  | CarP4750: | tmk |
|  |  | CarP4714: | putative hydrolase |
|  |  | CarP1435: | putative aminodeoxychorismate lyase |
|  |  | CarP1469: | hypothetical protein |
|  |  | CarP2622: | hypothetical protein |
|  |  | CarP1480: | gatA |
|  |  | CarP1577: | putative rhodanese-related sulfurtransferase |
|  |  | CarP1454: | bamA |
|  |  | CarP3831: | wapA |
|  |  | CarP1241: | lolC |
|  |  | CarP4771: | ankX |
|  |  | CarP1102: | gpsA |
|  |  | CarP1422: | atpD |
|  |  | CarP1428: | ffh |
|  |  | CarP1450: | clpP |
|  |  | CarP2024: | ZIP Zinc transporter |
|  |  | CarP1274: | rplO |
|  |  | CarP3121: | tetratricopeptide repeat protein |
|  |  | CarP1479: | dbpA |
|  |  | CarP1297: | sufC |
|  |  | CarP1002: | Transposase DDE domain protein |
|  |  | CarP1148: | rpsG |
|  |  | CarP1370: | zntR |
|  |  | CarP1144: | bamA |
|  |  | CarP1150: | atpG |
|  |  | CarP1123: | uvrC |
|  |  | CarP1240: | atpH |
|  |  | CarP1195: | PD-(D E)XK nuclease family transposase |
|  |  | CarP1494: | thyA |
|  |  | CarP1280: | rplE |
|  |  | CarP1361: | infC |
|  |  | CarP1124: | hypothetical protein |
|  |  | CarP1420: | ksgA |
|  |  | CarP1488: | 50S ribosomal protein L7/L12 |
|  |  | CarP1053: | Transposase, Mutator family |
|  |  | CarP1379: | nrnA |
|  |  | CarP1462: | hflX |
|  |  | CarP1442: | topA |
|  |  | CarP1047: | mrcA |
|  |  | CarP2626: | mepM |
|  |  | CarP1170: | mepM |
|  |  | CarP1176: | dnaG |
|  |  | CarP1245: | S1 P1 Nuclease |
|  |  | CarP1141: | gltX |
|  |  | CarP4753: | Ankyrinrepeats (3 copies) |
|  |  | CarP2008: | hypothetical protein |
|  |  | CarP3132: | mutS2 |
|  |  | CarP1273: | secY |
|  |  | CarP1465: | hypothetical protein |
|  |  | CarP1502: | bamA |
|  |  | CarP1453: | rpb6 |
|  |  | CarP1387: | holB |
|  |  | CarP1417: | gyrA |
|  |  | CarP1230: | hypothetical protein |
|  |  | CarP1436: | wbbL |
|  |  | CarP1288: | rpsS |
|  |  | CarP1467: | ftsZ |
|  |  | CarP1349: | pheS |
|  |  | CarP1106: | ruvC |
| CarB CarE | 102 | CarP6183: | hypothetical protein |
|  |  | CarP1563: | narL |
|  |  | CarP1547: | mtaB |
|  |  | CarP4723: | hypothetical protein |
|  |  | CarP1568: | rpmF |
|  |  | CarP1555: | Dihydrolipoyl dehydrogenase |
|  |  | CarP6186: | hypothetical protein |
|  |  | CarP6206: | hypothetical protein |
|  |  | CarP3143: | bioA |
|  |  | CarP2662: | bioF |
|  |  | CarP1069: | Integrase core domain protein |
|  |  | CarP2653: | SigmaW regulon antibacterial |
|  |  | CarP6194: | WH2 motif protein |
|  |  | CarP6229: | Bacterial regulatory protein, Fis family |
|  |  | CarP6198: | ankX |
|  |  | CarP1659: | lipA |
|  |  | CarP2519: | hypothetical protein |
|  |  | CarP6215: | hypothetical protein |
|  |  | CarP6218: | hypothetical protein |
|  |  | CarP1671: | lipB |
|  |  | CarP6223: | hypothetical protein |
|  |  | CarP6208: | hypothetical protein |
|  |  | CarP1554: | rpsF |
|  |  | CarP6203: | hypothetical protein |
|  |  | CarP6193: | relA |
|  |  | CarP6197: | hypothetical protein |
|  |  | CarP6137: | hypothetical protein |
|  |  | CarP6216: | hypothetical protein |
|  |  | CarP2020: | PAAR motif protein |
|  |  | CarP1540: | aceF |
|  |  | CarP1536: | acs |
|  |  | CarP1660: | tsaC |
|  |  | CarP1544: | tsaD |
|  |  | CarP6182: | hypothetical protein |
|  |  | CarP1549: | pepP |
|  |  | CarP6211: | Ankyrin repeat protein |
|  |  | CarP6217: | hypothetical protein |
|  |  | CarP1691: | hypothetical protein |
|  |  | CarP1463: | xerD |
|  |  | CarP1562: | nth |
|  |  | CarP3106: | ldh |
|  |  | CarP3128: | Gene 25-like lysozyme |
|  |  | CarP1177: | rfbC |
|  |  | CarP1757: | Glycosyl hydrolase family 1 |
|  |  | CarP6173: | ankX |
|  |  | CarP4751: | Beta-hexosaminidase |
|  |  | CarP6188: | hypothetical protein |
|  |  | CarP6227: | pdxT |
|  |  | CarP1561: | miaB |
|  |  | CarP6189: | hypothetical protein |
|  |  | CarP6204: | hypothetical protein |
|  |  | CarP1665: | murC |
|  |  | CarP1581: | gpx1 |
|  |  | CarP6228: | pdxS |
|  |  | CarP1217: | rfbB |
|  |  | CarP1569: | pgk |
|  |  | CarP1661: | phr |
|  |  | CarP6202: | hypothetical protein |
|  |  | CarP4713: | hhoA |
|  |  | CarP4726: | Lipid A export ATP-binding permease protei |
|  |  | CarP1926: | Transposase, Mutator family |
|  |  | CarP1434: | gap |
|  |  | CarP1511: | Integrase core domain protein |
|  |  | CarP1566: | bfmBAB |
|  |  | CarP6192: | hypothetical protein |
|  |  | CarP3617: | hypothetical protein |
|  |  | CarP1550: | gpmI |
|  |  | CarP1572: | dapF |
|  |  | CarP4491: | Ankyrin repeat protein |
|  |  | CarP2036: | rtcB |
|  |  | CarP3792: | hypothetical protein |
|  |  | CarP1552: | bfmBAB |
|  |  | CarP6185: | Nicotinamide mononucleotide transporter |
|  |  | CarP1928: | Transposase |
|  |  | CarP1064: | cspG |
|  |  | CarP6209: | hypothetical protein |
|  |  | CarP6220: | Alpha/beta hydrolase family protein |
|  |  | CarP6187: | hypothetical protein |
|  |  | CarP1669: | putative inorganic polyphosphate/ATP-NAD kinase |
|  |  | CarP6200: | hypothetical protein |
|  |  | CarP1548: | tpiA |
|  |  | CarP6219: | hypothetical protein |
|  |  | CarP1005: | Integrase core domain protein |
|  |  | CarP3613: | putative transporter |
|  |  | CarP6221: | hypothetical protein |
|  |  | CarP6199: | hypothetical protein |
|  |  | CarP1553: | hemN |
|  |  | CarP6225: | hypothetical protein |
|  |  | CarP3144: | bioD |
|  |  | CarP1259: | eno |
|  |  | CarP1582: | mscL |
|  |  | CarP6222: | ankX |
|  |  | CarP4710: | nudG |
|  |  | CarP6207: | hypothetical protein |
|  |  | CarP1565: | aceF |
|  |  | CarP6224: | bioC |
|  |  | CarP6190: | Ankyrinrepeats (3 copies) |
|  |  | CarP6212: | hypothetical protein |
|  |  | CarP6205: | Ankyrinrepeats (3 copies) |
|  |  | CarP6191: | Sodium/proline symporter |
|  |  | CarP2624: | ynaI |
|  |  | CarP1558: | rlmN |
| CarE CarP | 11 | CarP1008: | Transposase IS116 IS110 IS902 family prote |
|  |  | CarP4498: | UMP phosphatase |
|  |  | CarP1014: | Integrase core domain protein |
|  |  | CarP1673: | rpmH |
|  |  | CarP3806: | hypothetical protein |
|  |  | CarP1185: | Helix-hairpin-helix motif protein |
|  |  | CarP1174: | Acid shock protein |
|  |  | CarP2521: | hypothetical protein |
|  |  | CarP2040: | secE |
|  |  | CarP3147: | PQ loop repeat protein |
|  |  | CarP6232: | hypothetical protein |
| CarB CarP | 9 | CarP6214: | hypothetical protein |
|  |  | CarP1083: | hypothetical protein |
|  |  | CarP1190: | ankX |
|  |  | CarP1045: | hypothetical protein |
|  |  | CarP6195: | hypothetical protein |
|  |  | CarP4500: | hypothetical protein |
|  |  | CarP1513: | norM |
|  |  | CarP1687: | Transposase DDE domain protein |
|  |  | CarP1996: | ykgO |
| CarE | 28 | CarP1526: | hypothetical protein |
|  |  | CarP1738: | zraS |
|  |  | CarP1614: | Transposase DDE domain protein |
|  |  | CarP1692: | dnaG |
|  |  | CarP2039: | rbn |
|  |  | CarP3618: | Integrase core domain protein |
|  |  | CarP2038: | bmrU |
|  |  | CarP1863: | hypothetical protein |
|  |  | CarP4735: | rhtC |
|  |  | CarP5703: | hypothetical protein |
|  |  | CarP3807: | amiD |
|  |  | CarP1862: | pyrG |
|  |  | CarP1189: | hypothetical protein |
|  |  | CarP1650: | yidD |
|  |  | CarP1052: | tnpR |
|  |  | CarP6139: | lon |
|  |  | CarP1923: | Patatin-like phospholipase |
|  |  | CarP5702: | hypothetical protein |
|  |  | CarP6181: | hypothetical protein |
|  |  | CarP1864: | nudJ |
|  |  | CarP3787: | xerC |
|  |  | CarP4722: | bin3 |
|  |  | CarP1054: | kinE |
|  |  | CarP6233: | Tn3 transposase DDE domain protein |
|  |  | CarP6180: | Transposase, Mutator family |
|  |  | CarP2042: | NUDIX domain protein |
|  |  | CarP4495: | SMI1 KNR4 family protein |
|  |  | CarP3149: | bioB |
| CarB | 44 | CarP1025: | hlyB |
|  |  | CarP6177: | hypothetical protein |
|  |  | CarP5695: | hypothetical protein |
|  |  | CarP1927: | hypothetical protein |
|  |  | CarP5697: | Transposase IS66 family protein |
|  |  | CarP3616: | Transposase IS66 family protein |
|  |  | CarP5698: | Outer membrane efflux protein |
|  |  | CarP1925: | hypothetical protein |
|  |  | CarP6178: | hypothetical protein |
|  |  | CarP1859: | metC |
|  |  | CarP6172: | hypothetical protein |
|  |  | CarP1061: | ltrA |
|  |  | CarP1036: | Transposase DDE domain protein |
|  |  | CarP5691: | Sodium:solute symporter family protein |
|  |  | CarP5699: | hlyD |
|  |  | CarP4746: | yidK |
|  |  | CarP6175: | hypothetical protein |
|  |  | CarP1670: | Starch-binding associating with outer membrane |
|  |  | CarP4755: | hypothetical protein |
|  |  | CarP4494: | hypothetical protein |
|  |  | CarP6213: | hypothetical protein |
|  |  | CarP5700: | hypothetical protein |
|  |  | CarP1666: | radA |
|  |  | CarP5693: | Transposase DDE domain protein |
|  |  | CarP3615: | hypothetical protein |
|  |  | CarP3614: | Transposase DDE domain protein |
|  |  | CarP5694: | hypothetical protein |
|  |  | CarP5690: | hypothetical protein |
|  |  | CarP2977: | hypothetical protein |
|  |  | CarP1081: | hypothetical protein |
|  |  | CarP1082: | hypothetical protein |
|  |  | CarP5696: | hypothetical protein |
|  |  | CarP4492: | Transposase DDE domain protein |
|  |  | CarP3836: | DinB superfamily protein |
|  |  | CarP5692: | hypothetical protein |
|  |  | CarP6176: | hypothetical protein |
|  |  | CarP2976: | hypothetical protein |
|  |  | CarP6210: | hypothetical protein |
|  |  | CarP5701: | hypothetical protein |
|  |  | CarP2032: | sugE |
|  |  | CarP4493: | hypothetical protein |
|  |  | CarP1924: | hypothetical protein |
|  |  | CarP4490: | Ankyrinrepeats (3 copies) |
|  |  | CarP4729: | hypothetical protein |
| CarP | 87 | CarP1084: | hypothetical protein |
|  |  | CarP3630: | hypothetical protein |
|  |  | CarP2044: | pimB |
|  |  | CarP1603: | hypothetical protein |
|  |  | CarP3627: | hypothetical protein |
|  |  | CarP5729: | ankX |
|  |  | CarP4497: | spsI |
|  |  | CarP5732: | hypothetical protein |
|  |  | CarP3131: | pcm |
|  |  | CarP5722: | Ankyrinrepeats (3 copies) |
|  |  | CarP5710: | hypothetical protein |
|  |  | CarP4505: | hypothetical protein |
|  |  | CarP4503: | hypothetical protein |
|  |  | CarP5718: | hypothetical protein |
|  |  | CarP1658: | ftnA |
|  |  | CarP2046: | ddpX |
|  |  | CarP5712: | matE |
|  |  | CarP5709: | hypothetical protein |
|  |  | CarP2050: | smc |
|  |  | CarP3153: | yajL |
|  |  | CarP5705: | hypothetical protein |
|  |  | CarP1046: | inlJ |
|  |  | CarP2524: | hypothetical protein |
|  |  | CarP3624: | Ankyrinrepeats (3 copies) |
|  |  | CarP2978: | Bifunctional (p)ppGpp synthase hydrolase R |
|  |  | CarP5711: | hypothetical protein |
|  |  | CarP4501: | hypothetical protein |
|  |  | CarP2980: | SET domain protein |
|  |  | CarP2983: | hypothetical protein |
|  |  | CarP4502: | hypothetical protein |
|  |  | CarP5730: | hypothetical protein |
|  |  | CarP6153: | Alpha beta hydrolase family protein |
|  |  | CarP5704: | hypothetical protein |
|  |  | CarP2986: | hypothetical protein |
|  |  | CarP6130: | hypothetical protein |
|  |  | CarP5725: | hypothetical protein |
|  |  | CarP3849: | hflK |
|  |  | CarP1009: | formamidopyrimidine 5-formyluracil 5-hydr |
|  |  | CarP5721: | gltX2 |
|  |  | CarP3623: | hypothetical protein |
|  |  | CarP5724: | hypothetical protein |
|  |  | CarP3151: | Ankyrinrepeats (3 copies) |
|  |  | CarP2979: | hypothetical protein |
|  |  | CarP5715: | Ankyrinrepeats (3 copies) |
|  |  | CarP3621: | Ankyrinrepeats (3 copies) |
|  |  | CarP5719: | hypothetical protein |
|  |  | CarP5727: | hypothetical protein |
|  |  | CarP1200: | tuaD |
|  |  | CarP6235: | glpT |
|  |  | CarP5716: | hypothetical protein |
|  |  | CarP6237: | yvdP |
|  |  | CarP1693: | spa |
|  |  | CarP4504: | hypothetical protein |
|  |  | CarP5717: | hypothetical protein |
|  |  | CarP1929: | hypothetical protein |
|  |  | CarP3846: | btrR |
|  |  | CarP5713: | PD-(D E)XK nuclease family transposase |
|  |  | CarP5708: | hypothetical protein |
|  |  | CarP1651: | putative ABC transporter ATP-binding protein |
|  |  | CarP5723: | hypothetical protein |
|  |  | CarP5731: | Phosphatidylcholine-sterol acyltransferase precursor |
|  |  | CarP5714: | OPT oligopeptide transporter protein |
|  |  | CarP2045: | PKD domain protein |
|  |  | CarP2049: | dacB |
|  |  | CarP5706: | hypothetical protein |
|  |  | CarP3155: | prpE |
|  |  | CarP2523: | hypothetical protein |
|  |  | CarP2646: | Proline betaine transporter |
|  |  | CarP1007: | Transposase DDE domain protein |
|  |  | CarP1514: | metal-dependent hydrolase |
|  |  | CarP5734: | hypothetical protein |
|  |  | CarP5707: | hypothetical protein |
|  |  | CarP3619: | Ankyrinrepeats (3 copies) |
|  |  | CarP2043: | mnaA |
|  |  | CarP4499: | Alpha beta hydrolase family protein |
|  |  | CarP5720: | mnaA |
|  |  | CarP5733: | hypothetical protein |
|  |  | CarP5726: | Zinc finger, C3H C4 type (RING finger) |
|  |  | CarP5735: | Leucine Rich repeats (2 copies) |
|  |  | CarP3156: | hypothetical protein |
|  |  | CarP3817: | galE |
|  |  | CarP1512: | Leucine Rich repeats (2 copies) |
|  |  | CarP6148: | ribN |
|  |  | CarP4496: | hypothetical protein |
|  |  | CarP2982: | hypothetical protein |
|  |  | CarP3647: | hypothetical protein |
|  |  | CarP5728: | 2-phosphosulfolactate phosphatase |

**Supplementary Table 8.** Gene names for genes in ortholog groups from Figure 6B. CoreCar = *Cardinium* cPpe from *Pratylenchus penetrans,* and *Cardinium* strains cEper1 and cBtQ1; CoreOut = outgroup Bacteroidetes from taxa listed in Supplementary Table 4.

| Venn Grouping | Number | Group Name | Gene Name |
| --- | --- | --- | --- |
| CoreCar CoreOut | 349 | CarP1139: | trpS |
|  |  | CarP1329: | hypothetical protein |
|  |  | CarP1476: | murB |
|  |  | CarP1492: | nusG |
|  |  | CarP1451: | tig |
|  |  | CarP1464: | lysS |
|  |  | CarP1364: | mltD |
|  |  | CarP1310: | ybeY |
|  |  | CarP1232: | tsf |
|  |  | CarP1171: | nifA |
|  |  | CarP1252: | tsaB |
|  |  | CarP1412: | prsA |
|  |  | CarP1282: | rplN |
|  |  | CarP1449: | clpA |
|  |  | CarP1254: | nusA |
|  |  | CarP1249: | rplU |
|  |  | CarP1484: | prkC |
|  |  | CarP1271: | infA |
|  |  | CarP1447: | sufB |
|  |  | CarP1248: | Cell division protein ZapA |
|  |  | CarP1421: | epsM |
|  |  | CarP1243: | CDP-alcohol phosphatidyltransferase |
|  |  | CarP1375: | murE |
|  |  | CarP1299: | rbfA |
|  |  | CarP1253: | rpsJ |
|  |  | CarP1438: | murG |
|  |  | CarP1096: | Putative 1,2-phenylacetyl-CoA epoxidase, s |
|  |  | CarP1026: | clpC |
|  |  | CarP1134: | ftsW |
|  |  | CarP1145: | queA |
|  |  | CarP1234: | rpsI |
|  |  | CarP1135: | hypothetical protein |
|  |  | CarP1220: | rpsO |
|  |  | CarP1397: | Helix-hairpin-helix motif protein |
|  |  | CarP1298: | sufD |
|  |  | CarP1455: | lptD |
|  |  | CarP1452: | relA |
|  |  | CarP1100: | dnaE |
|  |  | CarP1130: | murA |
|  |  | CarP1103: | htrB |
|  |  | CarP1353: | tetratricopeptide repeat protein |
|  |  | CarP1372: | tyrS |
|  |  | CarP1042: | fabG |
|  |  | CarP1286: | rpsC |
|  |  | CarP1221: | pnp |
|  |  | CarP1474: | recA |
|  |  | CarP1457: | mnmA |
|  |  | CarP1291: | rplD |
|  |  | CarP1287: | rplV |
|  |  | CarP1399: | Rhomboid family protein |
|  |  | CarP1308: | ptrA |
|  |  | CarP1235: | rplM |
|  |  | CarP1263: | yfhQ |
|  |  | CarP1377: | ftsL |
|  |  | CarP1477: | hypothetical protein |
|  |  | CarP1219: | dnaA |
|  |  | CarP1470: | metG |
|  |  | CarP1238: | atpB |
|  |  | CarP1398: | rluA |
|  |  | CarP1092: | lspA |
|  |  | CarP1340: | smc |
|  |  | CarP1446: | der |
|  |  | CarP1158: | periplasmic chaperone |
|  |  | CarP1439: | gyrB |
|  |  | CarP1418: | gyrA |
|  |  | CarP1151: | atpA |
|  |  | CarP1165: | truA |
|  |  | CarP1352: | rplY |
|  |  | CarP1227: | queuine tRNA-ribosyltransferase |
|  |  | CarP1333: | rpsR |
|  |  | CarP1351: | dnaK |
|  |  | CarP1354: | acoA |
|  |  | CarP1335: | YtxH-like protein |
|  |  | CarP1441: | acpS |
|  |  | CarP1362: | rpmI |
|  |  | CarP1264: | ssb |
|  |  | CarP1146: | hypothetical protein |
|  |  | CarP1321: | groS5 |
|  |  | CarP1330: | ychF |
|  |  | CarP1392: | miaA |
|  |  | CarP1393: | sppA |
|  |  | CarP1070: | lepB |
|  |  | CarP1491: | rplK |
|  |  | CarP1324: | maeB |
|  |  | CarP1496: | recJ |
|  |  | CarP1236: | exbD |
|  |  | CarP1367: | ftsY |
|  |  | CarP1143: | DNA polymerase I |
|  |  | CarP1312: | lpxA |
|  |  | CarP1322: | accA |
|  |  | CarP1381: | hypothetical protein |
|  |  | CarP1228: | putative permease YjgP YjgQ family protein |
|  |  | CarP1225: | prmB |
|  |  | CarP1305: | mfd |
|  |  | CarP1131: | birA |
|  |  | CarP1405: | HIT-like protein |
|  |  | CarP1378: | argS |
|  |  | CarP1101: | priA |
|  |  | CarP1365: | rpmG |
|  |  | CarP1360: | thrS |
|  |  | CarP1400: | prfA |
|  |  | CarP1270: | rpsM |
|  |  | CarP1448: | mutL |
|  |  | CarP1132: | Putative zinc metalloprotease |
|  |  | CarP1317: | dhfrIII |
|  |  | CarP1444: | hypothetical protein |
|  |  | CarP1386: | gatA |
|  |  | CarP1172: | hypothetical protein |
|  |  | CarP1497: | lipoprotein chaperone |
|  |  | CarP1357: | Peptidase M16 inactive domain protein |
|  |  | CarP1246: | rpsT |
|  |  | CarP1294: | tadA |
|  |  | CarP1266: | rplQ |
|  |  | CarP1336: | N utilization substance protein B homolog |
|  |  | CarP1390: | hypothetical protein |
|  |  | CarP1304: | cgtA |
|  |  | CarP1350: | lptC |
|  |  | CarP1323: | dnaX |
|  |  | CarP1481: | frr |
|  |  | CarP1262: | hup |
|  |  | CarP1128: | mreB |
|  |  | CarP1224: | iscS |
|  |  | CarP1255: | infB |
|  |  | CarP1389: | rpsA |
|  |  | CarP1157: | efp |
|  |  | CarP1396: | lrp |
|  |  | CarP1431: | putative ABC transporter ATP-binding protein |
|  |  | CarP1068: | bacA |
|  |  | CarP1461: | murF |
|  |  | CarP1426: | grpE |
|  |  | CarP1226: | ppx |
|  |  | CarP1283: | rpsQ |
|  |  | CarP1500: | hypothetical protein |
|  |  | CarP1116: | fadB |
|  |  | CarP1373: | comM |
|  |  | CarP1024: | trxA |
|  |  | CarP1222: | sigA |
|  |  | CarP1118: | ideR |
|  |  | CarP1119: | tadA |
|  |  | CarP1478: | hypothetical protein |
|  |  | CarP1267: | rpoA |
|  |  | CarP1295: | ftsH |
|  |  | CarP1341: | dksA |
|  |  | CarP1331: | tRNA pseudouridine synthase B |
|  |  | CarP1111: | fbp |
|  |  | CarP1277: | rplR |
|  |  | CarP1414: | secA |
|  |  | CarP1275: | rpmD |
|  |  | CarP1348: | ndk |
|  |  | CarP1265: | smpB |
|  |  | CarP1167: | sodA |
|  |  | CarP1326: | lolC |
|  |  | CarP1343: | ileS |
|  |  | CarP1473: | rnr |
|  |  | CarP1129: | spoIIIE |
|  |  | CarP1498: | accA |
|  |  | CarP1229: | hslV |
|  |  | CarP1385: | cysS |
|  |  | CarP1429: | iscS |
|  |  | CarP1359: | nrdA |
|  |  | CarP1460: | acpP |
|  |  | CarP1475: | yqgF |
|  |  | CarP1409: | rpmE2 |
|  |  | CarP1347: | clpY |
|  |  | CarP1233: | rpsB |
|  |  | CarP1044: | soj |
|  |  | CarP1293: | fabD |
|  |  | CarP1382: | Non-canonical purine NTP pyrophosphatase |
|  |  | CarP1487: | rpoB |
|  |  | CarP1268: | rpsD |
|  |  | CarP1074: | fabB |
|  |  | CarP1380: | secG |
|  |  | CarP1147: | fus |
|  |  | CarP1296: | yidC |
|  |  | CarP1416: | dus |
|  |  | CarP1358: | nrdB |
|  |  | CarP1342: | rluA |
|  |  | CarP1159: | fabB |
|  |  | CarP1258: | Septum formation initiator |
|  |  | CarP1292: | rplC |
|  |  | CarP1247: | pheT |
|  |  | CarP1156: | hypothetical protein |
|  |  | CarP1284: | rpmC |
|  |  | CarP1125: | Colicin V production protein |
|  |  | CarP1087: | hypothetical protein |
|  |  | CarP1285: | rplP |
|  |  | CarP1313: | lpxC |
|  |  | CarP1406: | greA |
|  |  | CarP1104: | dnaB |
|  |  | CarP1432: | atpC |
|  |  | CarP1098: | cca |
|  |  | CarP1493: | elongation factor Tu |
|  |  | CarP1281: | rplX |
|  |  | CarP1334: | ung |
|  |  | CarP1302: | rho |
|  |  | CarP1376: | spoVD |
|  |  | CarP1486: | rpoC |
|  |  | CarP1430: | sufE |
|  |  | CarP1149: | asnS |
|  |  | CarP1410: | lolD |
|  |  | CarP1300: | mraY |
|  |  | CarP1404: | typA |
|  |  | CarP1251: | rpsP |
|  |  | CarP1142: | phosphatidylserine decarboxylase |
|  |  | CarP1366: | hypothetical protein |
|  |  | CarP1483: | yjjV |
|  |  | CarP1445: | Putative TrmH family tRNA rRNA methyltransferase |
|  |  | CarP1306: | gidA |
|  |  | CarP1489: | rplJ |
|  |  | CarP1346: | lon2 |
|  |  | CarP1178: | leuS |
|  |  | CarP1289: | rplB |
|  |  | CarP1290: | rplW |
|  |  | CarP1443: | Undecaprenyl-phosphate mannosyltransferase |
|  |  | CarP1407: | pth |
|  |  | CarP1408: | lpxB |
|  |  | CarP1337: | hypothetical protein |
|  |  | CarP1175: | ispU |
|  |  | CarP1503: | hypothetical protein |
|  |  | CarP1316: | uvrB |
|  |  | CarP1279: | rpsH |
|  |  | CarP1458: | mnmE |
|  |  | CarP1344: | ComEC family competence protein |
|  |  | CarP1437: | Nucleoid-associated protein |
|  |  | CarP1415: | Sporulation related domain protein |
|  |  | CarP1391: | gyrB |
|  |  | CarP1328: | fdx |
|  |  | CarP1471: | recQ |
|  |  | CarP1126: | recG |
|  |  | CarP1383: | engB |
|  |  | CarP1276: | rpsE |
|  |  | CarP1309: | proS |
|  |  | CarP1133: | glgA |
|  |  | CarP1424: | rplS |
|  |  | CarP1105: | rpoN |
|  |  | CarP1419: | minD |
|  |  | CarP1261: | rng |
|  |  | CarP1173: | lgt |
|  |  | CarP1368: | Aspartyl/glutamyl-tRNA(Asn/Gln) amidotransferase subunit B |
|  |  | CarP1166: | xerD |
|  |  | CarP1499: | hypothetical protein |
|  |  | CarP1137: | putative permease YjgP YjgQ family protein |
|  |  | CarP1136: | trmD |
|  |  | CarP1278: | rplF |
|  |  | CarP1423: | lepA |
|  |  | CarP1325: | tsaB |
|  |  | CarP1433: | prfB |
|  |  | CarP1071: | ykfA |
|  |  | CarP1244: | secD |
|  |  | CarP1369: | alaS |
|  |  | CarP1338: | lpxK |
|  |  | CarP1411: | mutS |
|  |  | CarP1099: | uvrD |
|  |  | CarP1231: | PPIC-type PPIASE domain protein |
|  |  | CarP1256: | rpsU |
|  |  | CarP1485: | ligA |
|  |  | CarP1394: | aviRb |
|  |  | CarP1401: | accB |
|  |  | CarP1425: | htpG |
|  |  | CarP1257: | alr |
|  |  | CarP1384: | degP |
|  |  | CarP1440: | era |
|  |  | CarP1427: | dnaJ |
|  |  | CarP1355: | mgtE |
|  |  | CarP1314: | lpxD |
|  |  | CarP1490: | rplA |
|  |  | CarP1239: | atpF |
|  |  | CarP1356: | Receptor family ligand binding region |
|  |  | CarP1097: | DNA polymerase III subunit delta |
|  |  | CarP1413: | Polysaccharide biosynthesis protein |
|  |  | CarP1482: | phosphodiesterase |
|  |  | CarP1168: | adenylate kinase |
|  |  | CarP1162: | ruvA |
|  |  | CarP1250: | rpmA |
|  |  | CarP1468: | valS |
|  |  | CarP1260: | DNA polymerase III subunit beta |
|  |  | CarP1272: | map |
|  |  | CarP1318: | hypothetical protein |
|  |  | CarP1169: | Putative multidrug export ATP-binding permease |
|  |  | CarP1395: | bamA |
|  |  | CarP1495: | lptB |
|  |  | CarP1402: | accC |
|  |  | CarP1332: | rplI |
|  |  | CarP1472: | gntX |
|  |  | CarP1459: | rnc |
|  |  | CarP1327: | hypothetical protein |
|  |  | CarP1339: | ybgI |
|  |  | CarP1029: | uvrA |
|  |  | CarP1301: | hisS |
|  |  | CarP1345: | ksgA |
|  |  | CarP1315: | ddlA |
|  |  | CarP1140: | hypothetical protein |
|  |  | CarP1456: | tilS |
|  |  | CarP1307: | glyA |
|  |  | CarP1269: | rpsK |
|  |  | CarP1127: | glyQS |
|  |  | CarP1363: | rplT |
|  |  | CarP1466: | ftsA |
|  |  | CarP1374: | rimM |
|  |  | CarP1223: | trxB |
|  |  | CarP1319: | parB |
|  |  | CarP1501: | ruvA |
|  |  | CarP1303: | serS |
|  |  | CarP1320: | groEL |
|  |  | CarP1388: | lptD |
|  |  | CarP1237: | tolQ |
|  |  | CarP1435: | putative aminodeoxychorismate lyase |
|  |  | CarP1469: | hypothetical protein |
|  |  | CarP1480: | gatA |
|  |  | CarP1454: | bamA |
|  |  | CarP1241: | lolC |
|  |  | CarP1102: | gpsA |
|  |  | CarP1422: | atpD |
|  |  | CarP1428: | ffh |
|  |  | CarP1450: | clpP |
|  |  | CarP1274: | rplO |
|  |  | CarP1479: | dbpA |
|  |  | CarP1297: | sufC |
|  |  | CarP1148: | rpsG |
|  |  | CarP1370: | zntR |
|  |  | CarP1144: | bamA |
|  |  | CarP1150: | atpG |
|  |  | CarP1240: | atpH |
|  |  | CarP1123: | uvrC |
|  |  | CarP1494: | thyA |
|  |  | CarP1280: | rplE |
|  |  | CarP1361: | infC |
|  |  | CarP1124: | hypothetical protein |
|  |  | CarP1420: | ksgA |
|  |  | CarP1488: | 50S ribosomal protein L7/L12 |
|  |  | CarP1379: | nrnA |
|  |  | CarP1462: | hflX |
|  |  | CarP1442: | topA |
|  |  | CarP1047: | mrcA |
|  |  | CarP1245: | S1 P1 Nuclease |
|  |  | CarP1176: | dnaG |
|  |  | CarP1141: | gltX |
|  |  | CarP1273: | secY |
|  |  | CarP1465: | hypothetical protein |
|  |  | CarP1502: | bamA |
|  |  | CarP1453: | rpb6 |
|  |  | CarP1387: | holB |
|  |  | CarP1417: | gyrA |
|  |  | CarP1436: | wbbL |
|  |  | CarP1230: | hypothetical protein |
|  |  | CarP1467: | ftsZ |
|  |  | CarP1288: | rpsS |
|  |  | CarP1349: | pheS |
|  |  | CarP1106: | ruvC |
| CoreOut | 143 | CarP1025: | hlyB |
|  |  | CarP1662: | rnhB |
|  |  | CarP1796: | polX |
|  |  | CarP1563: | narL |
|  |  | CarP1547: | mtaB |
|  |  | CarP1789: | dapE |
|  |  | CarP1606: | CAAX amino terminal protease self- immunity |
|  |  | CarP1770: | algA |
|  |  | CarP1568: | rpmF |
|  |  | CarP1654: | hypothetical protein |
|  |  | CarP1802: | Putative O-methyltransferase MSMEI 4947 |
|  |  | CarP1778: | hypothetical protein |
|  |  | CarP1108: | ybbD |
|  |  | CarP1858: | aspC |
|  |  | CarP1555: | Dihydrolipoyl dehydrogenase |
|  |  | CarP1787: | hypothetical protein |
|  |  | CarP1668: | pepN |
|  |  | CarP1599: | WbqC-like protein family protein |
|  |  | CarP1844: | hypothetical protein |
|  |  | CarP1829: | gycine oxidase |
|  |  | CarP1791: | hypothetical protein |
|  |  | CarP1851: | def |
|  |  | CarP1655: | pkn1 |
|  |  | CarP1793: | yajC |
|  |  | CarP1595: | putative metallophosphoesterase |
|  |  | CarP1645: | glmS |
|  |  | CarP1817: | hypothetical protein |
|  |  | CarP1766: | hypothetical protein |
|  |  | CarP1828: | ydiI |
|  |  | CarP1788: | ftsX |
|  |  | CarP1821: | hypothetical protein |
|  |  | CarP1652: | hypothetical protein |
|  |  | CarP1842: | hypothetical protein |
|  |  | CarP1857: | dapA |
|  |  | CarP1658: | ftnA |
|  |  | CarP1780: | ogt |
|  |  | CarP1814: | recO |
|  |  | CarP1818: | trmH |
|  |  | CarP1772: | hypothetical protein |
|  |  | CarP1626: | hypothetical protein |
|  |  | CarP1653: | hypothetical protein |
|  |  | CarP1554: | rpsF |
|  |  | CarP1657: | hypothetical protein |
|  |  | CarP1792: | hypothetical protein |
|  |  | CarP1813: | hypothetical protein |
|  |  | CarP1520: | hypothetical protein |
|  |  | CarP1762: | Phosphomannomutase phosphoglucomutase |
|  |  | CarP1807: | putative metallo-hydrolase |
|  |  | CarP1783: | prs |
|  |  | CarP1853: | fmt |
|  |  | CarP1540: | aceF |
|  |  | CarP1781: | dapB |
|  |  | CarP1799: | yheI |
|  |  | CarP1544: | tsaD |
|  |  | CarP1549: | pepP |
|  |  | CarP1812: | hypothetical protein |
|  |  | CarP1463: | xerD |
|  |  | CarP1790: | thiL |
|  |  | CarP1855: | mazG |
|  |  | CarP1185: | Helix-hairpin-helix motif protein |
|  |  | CarP1856: | Putative neutral zinc metallopeptidase |
|  |  | CarP1562: | nth |
|  |  | CarP1761: | araC |
|  |  | CarP1598: | Di- tripeptide transporter |
|  |  | CarP1177: | rfbC |
|  |  | CarP1815: | hypothetical protein |
|  |  | CarP1765: | fabI |
|  |  | CarP1174: | Acid shock protein |
|  |  | CarP1834: | putative CtpA-like serine protease |
|  |  | CarP1804: | ksgA |
|  |  | CarP1852: | GH3 auxin-responsive promoter |
|  |  | CarP1838: | yxeP |
|  |  | CarP1561: | miaB |
|  |  | CarP1771: | aspS |
|  |  | CarP1189: | hypothetical protein |
|  |  | CarP1600: | pyk |
|  |  | CarP1776: | mrdB |
|  |  | CarP1774: | ABC-2 family transporter protein |
|  |  | CarP1666: | radA |
|  |  | CarP1217: | rfbB |
|  |  | CarP1569: | pgk |
|  |  | CarP1650: | yidD |
|  |  | CarP1824: | pkn1 |
|  |  | CarP1831: | hypothetical protein |
|  |  | CarP1651: | putative ABC transporter ATP-binding protein |
|  |  | CarP1827: | folD |
|  |  | CarP1779: | mreC |
|  |  | CarP1763: | Putative multidrug export ATP-binding permease |
|  |  | CarP1840: | divIVA |
|  |  | CarP1841: | ndx1 |
|  |  | CarP1311: | Bifunctional transcriptional activator DNA |
|  |  | CarP1522: | hypothetical protein |
|  |  | CarP1596: | yhhQ |
|  |  | CarP1843: | recQ |
|  |  | CarP1786: | ysdC |
|  |  | CarP1809: | bifunctional N-acetylglucosamine-1-phosphate uridyltransferase |
|  |  | CarP1434: | gap |
|  |  | CarP1811: | hypothetical protein |
|  |  | CarP1566: | bfmBAB |
|  |  | CarP1777: | spoVD |
|  |  | CarP1769: | ilvB |
|  |  | CarP1803: | hypothetical protein |
|  |  | CarP1550: | gpmI |
|  |  | CarP1551: | pncC |
|  |  | CarP1850: | oprF |
|  |  | CarP1773: | ABC-type uncharacterized transport system |
|  |  | CarP1572: | dapF |
|  |  | CarP1830: | BadF BadG BcrA BcrD ATPase family protein |
|  |  | CarP1816: | tsaC |
|  |  | CarP1845: | recX |
|  |  | CarP1768: | hypothetical protein |
|  |  | CarP1785: | fbp |
|  |  | CarP1579: | UvrB uvrC motif protein |
|  |  | CarP1764: | tonB |
|  |  | CarP1552: | bfmBAB |
|  |  | CarP1775: | rsfS |
|  |  | CarP1836: | dapD |
|  |  | CarP1794: | asd |
|  |  | CarP1805: | hypothetical protein |
|  |  | CarP1846: | gltP |
|  |  | CarP1808: | aarA |
|  |  | CarP1767: | envC |
|  |  | CarP1760: | hypothetical protein |
|  |  | CarP1820: | ispB |
|  |  | CarP1094: | xerC |
|  |  | CarP1810: | amiA |
|  |  | CarP1548: | tpiA |
|  |  | CarP1832: | bcp |
|  |  | CarP1833: | menA |
|  |  | CarP1553: | hemN |
|  |  | CarP1798: | recF |
|  |  | CarP1819: | ywaD |
|  |  | CarP1849: | hypothetical protein |
|  |  | CarP1801: | ybeZ |
|  |  | CarP1259: | eno |
|  |  | CarP1825: | acdS |
|  |  | CarP1854: | ftsE |
|  |  | CarP1806: | recR |
|  |  | CarP1565: | aceF |
|  |  | CarP1782: | rarA |
|  |  | CarP1759: | rlmCD |
|  |  | CarP1837: | hypothetical protein |
|  |  | CarP1558: | rlmN |
| CoreCar | 154 | CarP4776: | asnS |
|  |  | CarP3628: | hypothetical protein |
|  |  | CarP2616: | plsB |
|  |  | CarP1541: | atpE |
|  |  | CarP1194: | cdsA |
|  |  | CarP1839: | rnhA |
|  |  | CarP4779: | tmk |
|  |  | CarP3631: | nfo |
|  |  | CarP4761: | ybhL |
|  |  | CarP4768: | Alpha/beta hydrolase family protein |
|  |  | CarP2027: | greA |
|  |  | CarP3119: | hypothetical protein |
|  |  | CarP3127: | hypothetical protein |
|  |  | CarP3626: | Ubiquitin carboxyl-terminal hydrolase |
|  |  | CarP1583: | ppa |
|  |  | CarP1037: | Sodium/proline symporter |
|  |  | CarP2987: | hypothetical protein |
|  |  | CarP3625: | tlcA |
|  |  | CarP4777: | Mitochondrial carrier protein |
|  |  | CarP4762: | anaerobic C4-dicarboxylate transporter |
|  |  | CarP4745: | mutS |
|  |  | CarP2623: | mntB |
|  |  | CarP3142: | macB |
|  |  | CarP2973: | oppD |
|  |  | CarP3797: | hypothetical protein |
|  |  | CarP4766: | hypothetical protein |
|  |  | CarP3833: | spoIVFB |
|  |  | CarP1188: | PD-(D E)XK nuclease superfamily protein |
|  |  | CarP1564: | mce related protein |
|  |  | CarP2627: | Polymer-forming cytoskeletal |
|  |  | CarP1559: | rpmB |
|  |  | CarP1560: | hypothetical protein |
|  |  | CarP1193: | mlaE |
|  |  | CarP2640: | hypothetical protein |
|  |  | CarP1542: | bcp |
|  |  | CarP2984: | hypothetical protein |
|  |  | CarP1694: | dut |
|  |  | CarP2630: | hypothetical protein |
|  |  | CarP3820: | hypothetical protein |
|  |  | CarP1664: | Acyltransferase |
|  |  | CarP1919: | gsiC |
|  |  | CarP2981: | clpP |
|  |  | CarP1021: | putative AAA-ATPase |
|  |  | CarP4780: | Outer membrane efflux protein |
|  |  | CarP3788: | dcuA |
|  |  | CarP4759: | CAAX amino terminal protease self- immunity |
|  |  | CarP4763: | hypothetical protein |
|  |  | CarP1059: | flagellar assembly protein H |
|  |  | CarP3842: | macA |
|  |  | CarP2641: | Leucine permease transcriptional regulator helical domain protein |
|  |  | CarP1567: | disA |
|  |  | CarP1085: | lyc |
|  |  | CarP2029: | pepA |
|  |  | CarP1120: | macB |
|  |  | CarP4749: | tlcA |
|  |  | CarP4754: | hypothetical protein |
|  |  | CarP1093: | dmpP |
|  |  | CarP1584: | ribonuclease P |
|  |  | CarP1602: | alkA |
|  |  | CarP4767: | ppdK |
|  |  | CarP1931: | T4-like virus tail tube protein gp19 |
|  |  | CarP4781: | macB |
|  |  | CarP4784: | Bacterial regulatory protein, Fis family |
|  |  | CarP4769: | hypothetical protein |
|  |  | CarP1557: | PAP2 superfamily protein |
|  |  | CarP3832: | Putative reductase/y4119/YP 4011 |
|  |  | CarP1186: | oppD |
|  |  | CarP1586: | ksgA |
|  |  | CarP2019: | Phage tail sheath protein |
|  |  | CarP1543: | rpsL |
|  |  | CarP4765: | hypothetical protein |
|  |  | CarP3819: | hypothetical protein |
|  |  | CarP3622: | putative hydrolase |
|  |  | CarP3620: | hypothetical protein |
|  |  | CarP4774: | Major Facilitator Superfamily protein |
|  |  | CarP3800: | hypothetical protein |
|  |  | CarP1030: | putative transposase |
|  |  | CarP4772: | hypothetical protein |
|  |  | CarP1576: | ATP-dependent helicase nuclease subunit A |
|  |  | CarP3095: | hypothetical protein |
|  |  | CarP1797: | arnT |
|  |  | CarP2002: | Baseplate J-like protein |
|  |  | CarP1570: | Peptidase family M3 |
|  |  | CarP3809: | ypwA |
|  |  | CarP4756: | hypothetical protein |
|  |  | CarP1922: | mutS |
|  |  | CarP2517: | dppB |
|  |  | CarP1930: | Transposase DDE domain protein |
|  |  | CarP1580: | trmB |
|  |  | CarP2985: | hypothetical protein |
|  |  | CarP3789: | hypothetical protein |
|  |  | CarP4782: | CAAX amino terminal protease self- immunity |
|  |  | CarP2552: | hypothetical protein |
|  |  | CarP3825: | ribN |
|  |  | CarP4760: | hypothetical protein |
|  |  | CarP4770: | hypothetical protein |
|  |  | CarP3843: | lnt |
|  |  | CarP1601: | Transposase DDE domain protein |
|  |  | CarP4748: | hypothetical protein |
|  |  | CarP3805: | Putative multidrug export ATP-binding permease |
|  |  | CarP1138: | dnaQ |
|  |  | CarP3818: | hypothetical protein |
|  |  | CarP1920: | hbpA |
|  |  | CarP1921: | Bacterial regulatory protein, Fis family |
|  |  | CarP1546: | rpsN2 |
|  |  | CarP2629: | hypothetical protein |
|  |  | CarP2637: | mntB |
|  |  | CarP1585: | metB |
|  |  | CarP4758: | hypothetical protein |
|  |  | CarP2005: | nhaP2 |
|  |  | CarP2522: | Alpha beta hydrolase family protein |
|  |  | CarP1060: | Transposase DDE domain protein |
|  |  | CarP2639: | Phage late control gene D protein (GPD) |
|  |  | CarP3837: | kgtP |
|  |  | CarP1571: | fadD |
|  |  | CarP4747: | hypothetical protein |
|  |  | CarP2520: | tlcA |
|  |  | CarP2650: | citrate transporter |
|  |  | CarP2652: | rmuC |
|  |  | CarP1688: | pld |
|  |  | CarP3629: | Bacterial leucyl aminopeptidase precursor |
|  |  | CarP1192: | metN |
|  |  | CarP4752: | hypothetical protein |
|  |  | CarP3798: | hypothetical protein |
|  |  | CarP3138: | hypothetical protein |
|  |  | CarP1556: | murI |
|  |  | CarP1010: | hcpC |
|  |  | CarP3799: | hypothetical protein |
|  |  | CarP1545: | hypothetical protein |
|  |  | CarP3829: | ddlB |
|  |  | CarP1011: | Bifunctional (p)ppGpp synthase hydrolase R |
|  |  | CarP3096: | tolB |
|  |  | CarP1573: | murD |
|  |  | CarP4764: | hypothetical protein |
|  |  | CarP1191: | lpxH |
|  |  | CarP1835: | troA |
|  |  | CarP1578: | znuC |
|  |  | CarP3120: | hypothetical protein |
|  |  | CarP4750: | tmk |
|  |  | CarP4714: | putative hydrolase |
|  |  | CarP2622: | hypothetical protein |
|  |  | CarP1577: | putative rhodanese-related sulfurtransferase |
|  |  | CarP3831: | wapA |
|  |  | CarP4771: | ankX |
|  |  | CarP2024: | ZIP Zinc transporter |
|  |  | CarP3121: | tetratricopeptide repeat protein |
|  |  | CarP1002: | Transposase DDE domain protein |
|  |  | CarP1195: | PD-(D E)XK nuclease family transposase |
|  |  | CarP1053: | Transposase, Mutator family |
|  |  | CarP2626: | mepM |
|  |  | CarP1170: | mepM |
|  |  | CarP4753: | Ankyrinrepeats (3 copies) |
|  |  | CarP2008: | hypothetical protein |
|  |  | CarP3132: | mutS2 |

**Supplementary Table 9.** Gene names for genes in ortholog groups from Figure 6C. CardiniumPangenome = all genes occurring in at least one main chromosome of one strain of Cardinium; cardPLAS = all genes occurring in at least one plasmid from one strain of Cardinium; outgroupPLAS = all genes occurring in at least one plasmid from one strain of outgroup Bacteroidetes species listed in Supplementary Table 4; outgroupPangenome = all genes occurring in at least one main chromosome of one Bacteroidetes species listed in Supplementary Table 4.

| Venn Group | Number | Group Name | Gene Name |
| --- | --- | --- | --- |
| CardiniumPangenome cardPLAS outgroupPLAS outgroupPangenome | 4 | CarP1044: | soj |
|  |  | CarP1005: | Integrase core domain protein |
|  |  | CarP1008: | Transposase IS116 IS110 IS902 family protein |
|  |  | CarP1052: | tnpR |
| CardiniumPangenome outgroupPLAS outgroupPangenome | 20 | CarP1923: | Patatin-like phospholipase |
|  |  | CarP3787: | xerC |
|  |  | CarP1100: | dnaE |
|  |  | CarP1061: | ltrA |
|  |  | CarP1093: | dmpP |
|  |  | CarP1024: | trxA |
|  |  | CarP1099: | uvrD |
|  |  | CarP1168: | adenylate kinase |
|  |  | CarP1259: | eno |
|  |  | CarP1042: | fabG |
|  |  | CarP1068: | bacA |
|  |  | CarP1175: | ispU |
|  |  | CarP3156: | hypothetical protein |
|  |  | CarP1172: | hypothetical protein |
|  |  | CarP1463: | xerD |
|  |  | CarP1757: | Glycosyl hydrolase family 1 |
|  |  | CarP1036: | Transposase DDE domain protein |
|  |  | CarP1170: | mepM |
|  |  | CarP1557: | PAP2 superfamily protein |
|  |  | CarP1171: | nifA |
| cardPLAS outgroupPLAS outgroupPangenome | 4 | CarP1403: | Plasmid recombination enzyme |
|  |  | CarP1575: | virB4 |
|  |  | CarP3810: | CobQ CobB MinD ParA nucleotide binding domain protein |
|  |  | CarP1663: | traD |
| CardiniumPangenome cardPLAS outgroupPangenome | 13 | CarP1030: | putative transposase |
|  |  | CarP1069: | Integrase core domain protein |
|  |  | CarP1195: | PD-(D E)XK nuclease family transposase |
|  |  | CarP1045: | hypothetical protein |
|  |  | CarP1511: | Integrase core domain protein |
|  |  | CarP1002: | Transposase DDE domain protein |
|  |  | CarP1053: | Transposase, Mutator family |
|  |  | CarP4729: | hypothetical protein |
|  |  | CarP1928: | Transposase |
|  |  | CarP1059: | flagellar assembly protein H |
|  |  | CarP1010: | hcpC |
|  |  | CarP1692: | dnaG |
|  |  | CarP1166: | xerD |
| outgroupPLAS outgroupPangenome | 303 | CarP7499: | hypothetical protein |
|  |  | CarP3174: | cirA |
|  |  | CarP6377: | hypothetical protein |
|  |  | CarP2734: | Outer membrane efflux protein |
|  |  | CarP7501: | Serine threonine-protein kinase HipA |
|  |  | CarP7763: | hypothetical protein |
|  |  | CarP1213: | hypothetical protein |
|  |  | CarP1117: | btr |
|  |  | CarP3530: | hypothetical protein |
|  |  | CarP1885: | fecA |
|  |  | CarP5509: | hypothetical protein |
|  |  | CarP2601: | pgl |
|  |  | CarP7485: | hypothetical protein |
|  |  | CarP1208: | mdtC |
|  |  | CarP1733: | Endo-1,4-beta-xylanase feruloyl esterase protein |
|  |  | CarP7770: | hypothetical protein |
|  |  | CarP5118: | ureG |
|  |  | CarP5604: | Cytochrome D1 heme domain protein |
|  |  | CarP1643: | Bestrophin, RFP-TM, chloride channel |
|  |  | CarP2515: | hypothetical protein |
|  |  | CarP1066: | hypothetical protein |
|  |  | CarP1977: | uxaA |
|  |  | CarP4254: | DsrE DsrF-like family protein |
|  |  | CarP4476: | hpaIIM |
|  |  | CarP4035: | multifunctional acyl-CoA thioesterase I an |
|  |  | CarP3648: | CobQ CobB MinD ParA nucleotide binding dom |
|  |  | CarP7767: | hypothetical protein |
|  |  | CarP7773: | hypothetical protein |
|  |  | CarP7480: | hypothetical protein |
|  |  | CarP3047: | hypothetical protein |
|  |  | CarP3474: | hypothetical protein |
|  |  | CarP1684: | putative Mg(2+) transport ATPase |
|  |  | CarP5124: | hypothetical protein |
|  |  | CarP2179: | Alpha-ketoglutaric semialdehyde dehydrogen |
|  |  | CarP4838: | zupT |
|  |  | CarP4342: | hypothetical protein |
|  |  | CarP1946: | hypothetical protein |
|  |  | CarP3043: | hypothetical protein |
|  |  | CarP1180: | CAI-1 autoinducer sensor kinase phosphatase |
|  |  | CarP1183: | hypothetical protein |
|  |  | CarP4475: | hypothetical protein |
|  |  | CarP5661: | NHL repeat protein |
|  |  | CarP7340: | hypothetical protein |
|  |  | CarP1040: | transposase IS protein |
|  |  | CarP1823: | pksN |
|  |  | CarP5572: | hypothetical protein |
|  |  | CarP1703: | cusA |
|  |  | CarP7339: | hypothetical protein |
|  |  | CarP1915: | ccoP |
|  |  | CarP1164: | Archaeal ATPase |
|  |  | CarP1592: | yedV |
|  |  | CarP3572: | hypothetical protein |
|  |  | CarP4477: | hypothetical protein |
|  |  | CarP1656: | hypothetical protein |
|  |  | CarP2247: | hypothetical protein |
|  |  | CarP1755: | hypothetical protein |
|  |  | CarP2590: | lexA |
|  |  | CarP3513: | hypothetical protein |
|  |  | CarP1723: | hypothetical protein |
|  |  | CarP1032: | rcp1 |
|  |  | CarP1886: | higB-1 |
|  |  | CarP1538: | anaerobic benzoate catabolism transcriptional regulator |
|  |  | CarP3740: | tuaC |
|  |  | CarP3016: | hypothetical protein |
|  |  | CarP3855: | rhaS |
|  |  | CarP2747: | fpaP |
|  |  | CarP1518: | dnaG |
|  |  | CarP7768: | hypothetical protein |
|  |  | CarP6873: | crtF |
|  |  | CarP4656: | Serine threonine-protein kinase HipA |
|  |  | CarP7765: | Transposase |
|  |  | CarP5660: | hypothetical protein |
|  |  | CarP4215: | btr |
|  |  | CarP1715: | EcoKI restriction-modification system protein |
|  |  | CarP1911: | hypothetical protein |
|  |  | CarP1636: | melR |
|  |  | CarP6852: | hypothetical protein |
|  |  | CarP2671: | melR |
|  |  | CarP4978: | fes |
|  |  | CarP3602: | hypothetical protein |
|  |  | CarP7771: | hypothetical protein |
|  |  | CarP6630: | hypothetical protein |
|  |  | CarP3050: | Transposase |
|  |  | CarP4189: | cueO |
|  |  | CarP2885: | aseR |
|  |  | CarP4480: | hypothetical protein |
|  |  | CarP7769: | hypothetical protein |
|  |  | CarP2472: | xylI |
|  |  | CarP1903: | hypothetical protein |
|  |  | CarP6663: | cadA |
|  |  | CarP7772: | hypothetical protein |
|  |  | CarP2668: | Cytochrome c |
|  |  | CarP7483: | Periplasmic [NiFeSe] hydrogenase large sub |
|  |  | CarP1090: | TonB-dependent Receptor Plug Domain protein |
|  |  | CarP5016: | hypothetical protein |
|  |  | CarP6629: | MotA TolQ ExbB proton channel family prote |
|  |  | CarP7484: | hydA |
|  |  | CarP2582: | hypothetical protein |
|  |  | CarP4343: | fumD |
|  |  | CarP7505: | hypothetical protein |
|  |  | CarP7482: | hybD |
|  |  | CarP7500: | pleD |
|  |  | CarP1163: | Helix-turn-helix domain protein |
|  |  | CarP1998: | sspH1 |
|  |  | CarP3453: | copA |
|  |  | CarP1905: | ntcA |
|  |  | CarP4050: | Trehalose utilisation |
|  |  | CarP4578: | hypothetical protein |
|  |  | CarP7230: | hypothetical protein |
|  |  | CarP2022: | Radical SAM superfamily protein |
|  |  | CarP3340: | hypothetical protein |
|  |  | CarP2896: | hypothetical protein |
|  |  | CarP2445: | cbgA |
|  |  | CarP2826: | putative type I restriction enzymeP M prot |
|  |  | CarP7206: | selD |
|  |  | CarP1574: | birA |
|  |  | CarP1015: | btuB |
|  |  | CarP7491: | RHS Repeat protein |
|  |  | CarP1826: | hypothetical protein |
|  |  | CarP3232: | gdhB |
|  |  | CarP1048: | xylI |
|  |  | CarP4727: | EamA-like transporter family protein |
|  |  | CarP2766: | hypothetical protein |
|  |  | CarP4481: | hypothetical protein |
|  |  | CarP4977: | proA |
|  |  | CarP3895: | hypothetical protein |
|  |  | CarP1077: | Transposase |
|  |  | CarP6466: | glpF |
|  |  | CarP1897: | fucP |
|  |  | CarP2591: | DNA polymerase V subunit UmuC |
|  |  | CarP4438: | hypothetical protein |
|  |  | CarP1153: | pacS |
|  |  | CarP3431: | higA |
|  |  | CarP3201: | hypothetical protein |
|  |  | CarP1214: | hypothetical protein |
|  |  | CarP3175: | LytTr DNA-binding domain protein |
|  |  | CarP6557: | Hydrogenase expression formation protein H |
|  |  | CarP3294: | mntH |
|  |  | CarP6958: | hypothetical protein |
|  |  | CarP1648: | nhaX |
|  |  | CarP7415: | YcfA-like protein |
|  |  | CarP1618: | ziaA |
|  |  | CarP3988: | Nickel uptake substrate-specific transmemb |
|  |  | CarP4129: | hypothetical protein |
|  |  | CarP7504: | hypothetical protein |
|  |  | CarP2928: | gmhA |
|  |  | CarP4319: | yfkN |
|  |  | CarP2411: | hypothetical protein |
|  |  | CarP5150: | hypothetical protein |
|  |  | CarP1705: | copB |
|  |  | CarP1902: | hypothetical protein |
|  |  | CarP7362: | Cytochrome D1 heme domain protein |
|  |  | CarP4150: | yrbG |
|  |  | CarP2737: | rhaB |
|  |  | CarP3083: | hypothetical protein |
|  |  | CarP2461: | hypothetical protein |
|  |  | CarP7489: | hypF |
|  |  | CarP1206: | hypothetical protein |
|  |  | CarP2929: | lipid A 1-phosphatase |
|  |  | CarP3302: | hypothetical protein |
|  |  | CarP5324: | Polysaccharide biosynthesis export protein |
|  |  | CarP1686: | tauE |
|  |  | CarP1530: | hypothetical protein |
|  |  | CarP1012: | czcA |
|  |  | CarP3447: | Fumarylacetoacetate (FAA) hydrolase family |
|  |  | CarP1633: | cocE |
|  |  | CarP3065: | Helix-turn-helix domain protein |
|  |  | CarP4188: | hypothetical protein |
|  |  | CarP6142: | baeB |
|  |  | CarP3094: | ycgJ |
|  |  | CarP1028: | Paired box' domain protein |
|  |  | CarP7774: | hypothetical protein |
|  |  | CarP7764: | hypothetical protein |
|  |  | CarP1649: | vapC |
|  |  | CarP3356: | dinB |
|  |  | CarP7490: | hydrogenase nickel incorporation protein |
|  |  | CarP7502: | Serine threonine-protein kinase HipA |
|  |  | CarP6982: | hypothetical protein |
|  |  | CarP1078: | hypothetical protein |
|  |  | CarP4991: | Fatty acid hydroxylase superfamily protein |
|  |  | CarP6949: | kdgR |
|  |  | CarP7487: | Hydrogenase expression formation protein H |
|  |  | CarP5634: | Divergent AAA domain protein |
|  |  | CarP5553: | hypothetical protein |
|  |  | CarP4891: | hypothetical protein |
|  |  | CarP4095: | hypothetical protein |
|  |  | CarP2761: | ade |
|  |  | CarP1907: | hypothetical protein |
|  |  | CarP2012: | hypothetical protein |
|  |  | CarP2971: | btr |
|  |  | CarP4479: | hypothetical protein |
|  |  | CarP1079: | lytR |
|  |  | CarP4329: | Xylosidase arabinosidase |
|  |  | CarP3275: | NnrU protein |
|  |  | CarP2948: | hypothetical protein |
|  |  | CarP4853: | xynZ |
|  |  | CarP7488: | hypC |
|  |  | CarP2883: | Cadmium, cobalt and zinc H(+)-K(+) antipor |
|  |  | CarP7486: | hypothetical protein |
|  |  | CarP4253: | N-6 DNA Methylase |
|  |  | CarP4107: | iolE |
|  |  | CarP7777: | hypothetical protein |
|  |  | CarP3190: | TMAO DMSO reductase |
|  |  | CarP6724: | hypothetical protein |
|  |  | CarP4216: | hypothetical protein |
|  |  | CarP1508: | cusR |
|  |  | CarP1973: | hypothetical protein |
|  |  | CarP1155: | hypothetical protein |
|  |  | CarP1869: | nadE |
|  |  | CarP1212: | Transposase |
|  |  | CarP3295: | gnl |
|  |  | CarP1055: | yliI |
|  |  | CarP3656: | hypothetical protein |
|  |  | CarP3339: | bepC |
|  |  | CarP1537: | coxN |
|  |  | CarP4657: | Serine threonine-protein kinase HipA |
|  |  | CarP1940: | terB |
|  |  | CarP5230: | celK |
|  |  | CarP5408: | hypothetical protein |
|  |  | CarP1551: | pncC |
|  |  | CarP1579: | UvrB uvrC motif protein |
|  |  | CarP1043: | ypdB |
|  |  | CarP1075: | yehU |
|  |  | CarP2028: | ampC |
|  |  | CarP3297: | H(+) Cl(-) exchange transporter ClcA |
|  |  | CarP3191: | hypothetical protein |
|  |  | CarP5155: | xylI |
|  |  | CarP7416: | hypothetical protein |
|  |  | CarP1182: | Relaxase Mobilisation nuclease domain protein |
|  |  | CarP1073: | cnrB |
|  |  | CarP3500: | limB |
|  |  | CarP1519: | Ribbon-helix-helix protein, copG family |
|  |  | CarP1683: | merP |
|  |  | CarP5667: | hypothetical protein |
|  |  | CarP6944: | hsdR |
|  |  | CarP4788: | rutR |
|  |  | CarP5670: | exonuclease VIII |
|  |  | CarP7241: | hypothetical protein |
|  |  | CarP7493: | Myo-inositol-1-phosphate synthase |
|  |  | CarP3868: | soxA |
|  |  | CarP7494: | Carbohydrate acetyl esterase feruloyl este |
|  |  | CarP3148: | hypothetical protein |
|  |  | CarP5226: | hypothetical protein |
|  |  | CarP3982: | cysL |
|  |  | CarP3141: | rcp1 |
|  |  | CarP5616: | xynB |
|  |  | CarP1611: | cusB |
|  |  | CarP1067: | yycG |
|  |  | CarP4451: | hypothetical protein |
|  |  | CarP1035: | Integrase core domain protein |
|  |  | CarP1615: | anr |
|  |  | CarP1890: | atsA |
|  |  | CarP5646: | ycgF |
|  |  | CarP1894: | sigH |
|  |  | CarP7475: | outer membrane porin, OprD family |
|  |  | CarP7481: | hypothetical protein |
|  |  | CarP7433: | hypothetical protein |
|  |  | CarP1624: | hypothetical protein |
|  |  | CarP1242: | higA-1 |
|  |  | CarP1000: | btuB |
|  |  | CarP7300: | cirA |
|  |  | CarP7002: | xynZ |
|  |  | CarP3722: | putative methyltransferase |
|  |  | CarP1179: | bepF |
|  |  | CarP5119: | cobN |
|  |  | CarP1161: | blh |
|  |  | CarP3571: | hypothetical protein |
|  |  | CarP4478: | hypothetical protein |
|  |  | CarP2827: | Helix-turn-helix |
|  |  | CarP1629: | higB |
|  |  | CarP4088: | hypothetical protein |
|  |  | CarP3529: | cusB |
|  |  | CarP7766: | low affinity iron permease |
|  |  | CarP4631: | hypothetical protein |
|  |  | CarP1784: | ydaF |
|  |  | CarP1089: | hypothetical protein |
|  |  | CarP1908: | idhA |
|  |  | CarP3418: | hypothetical protein |
|  |  | CarP7393: | yicJ |
|  |  | CarP4282: | hypothetical protein |
|  |  | CarP2884: | hypothetical protein |
|  |  | CarP3438: | outer membrane channel protein |
|  |  | CarP1739: | Fibronectin type III domain protein |
|  |  | CarP1019: | ypdA |
|  |  | CarP4030: | sauU |
|  |  | CarP1704: | pbpE |
|  |  | CarP6327: | putative endoribonuclease L-PSP |
|  |  | CarP7152: | cmoA |
|  |  | CarP4981: | hypothetical protein |
|  |  | CarP1122: | SusD family protein |
|  |  | CarP1080: | tesA |
|  |  | CarP2793: | Xylose isomerase-like TIM barrel |
|  |  | CarP5201: | Metallo-beta-lactamase superfamily protein |
|  |  | CarP5125: | hypothetical protein |
|  |  | CarP3435: | gudD |
|  |  | CarP5443: | hypothetical protein |
|  |  | CarP2825: | traC |
|  |  | CarP1714: | actIII |
|  |  | CarP3909: | blc |
|  |  | CarP6367: | hypothetical protein |
|  |  | CarP3133: | hypothetical protein |
|  |  | CarP5030: | hypothetical protein |
|  |  | CarP6909: | selU |
| CardiniumPangenome outgroupPLAS | 1 | CarP6233: | Tn3 transposase DDE domain protein |
| cardPLAS outgroupPLAS | 3 | CarP4744: | hypothetical protein |
|  |  | CarP3102: | antiporter inner membrane protein |
|  |  | CarP6179: | hypothetical protein |
| CardiniumPangenome outgroupPangenome | 553 | CarP2616: | plsB |
|  |  | CarP1464: | lysS |
|  |  | CarP1194: | cdsA |
|  |  | CarP1252: | tsaB |
|  |  | CarP1421: | epsM |
|  |  | CarP1253: | rpsJ |
|  |  | CarP1145: | queA |
|  |  | CarP1583: | ppa |
|  |  | CarP1738: | zraS |
|  |  | CarP1452: | relA |
|  |  | CarP4505: | hypothetical protein |
|  |  | CarP1188: | PD-(D E)XK nuclease superfamily protein |
|  |  | CarP1158: | periplasmic chaperone |
|  |  | CarP1564: | mce related protein |
|  |  | CarP2038: | bmrU |
|  |  | CarP1333: | rpsR |
|  |  | CarP1354: | acoA |
|  |  | CarP1193: | mlaE |
|  |  | CarP1491: | rplK |
|  |  | CarP1496: | recJ |
|  |  | CarP1367: | ftsY |
|  |  | CarP1694: | dut |
|  |  | CarP1664: | Acyltransferase |
|  |  | CarP2981: | clpP |
|  |  | CarP2641: | Leucine permease transcriptional regulator helical domain protein |
|  |  | CarP1444: | hypothetical protein |
|  |  | CarP2029: | pepA |
|  |  | CarP1085: | lyc |
|  |  | CarP1584: | ribonuclease P |
|  |  | CarP1540: | aceF |
|  |  | CarP1185: | Helix-hairpin-helix motif protein |
|  |  | CarP4751: | Beta-hexosaminidase |
|  |  | CarP6213: | hypothetical protein |
|  |  | CarP1268: | rpsD |
|  |  | CarP1342: | rluA |
|  |  | CarP1581: | gpx1 |
|  |  | CarP1570: | Peptidase family M3 |
|  |  | CarP1247: | pheT |
|  |  | CarP1666: | radA |
|  |  | CarP1087: | hypothetical protein |
|  |  | CarP2517: | dppB |
|  |  | CarP1281: | rplX |
|  |  | CarP1430: | sufE |
|  |  | CarP1306: | gidA |
|  |  | CarP1503: | hypothetical protein |
|  |  | CarP1309: | proS |
|  |  | CarP2646: | Proline betaine transporter |
|  |  | CarP1920: | hbpA |
|  |  | CarP1585: | metB |
|  |  | CarP6139: | lon |
|  |  | CarP1384: | degP |
|  |  | CarP1427: | dnaJ |
|  |  | CarP3837: | kgtP |
|  |  | CarP1318: | hypothetical protein |
|  |  | CarP2043: | mnaA |
|  |  | CarP1339: | ybgI |
|  |  | CarP1029: | uvrA |
|  |  | CarP1064: | cspG |
|  |  | CarP6210: | hypothetical protein |
|  |  | CarP3121: | tetratricopeptide repeat protein |
|  |  | CarP2042: | NUDIX domain protein |
|  |  | CarP1240: | atpH |
|  |  | CarP1488: | 50S ribosomal protein L7/L12 |
|  |  | CarP1462: | hflX |
|  |  | CarP1047: | mrcA |
|  |  | CarP3149: | bioB |
|  |  | CarP1417: | gyrA |
|  |  | CarP1558: | rlmN |
|  |  | CarP1476: | murB |
|  |  | CarP4723: | hypothetical protein |
|  |  | CarP2027: | greA |
|  |  | CarP1254: | nusA |
|  |  | CarP1484: | prkC |
|  |  | CarP1243: | CDP-alcohol phosphatidyltransferase |
|  |  | CarP2662: | bioF |
|  |  | CarP1340: | smc |
|  |  | CarP2627: | Polymer-forming cytoskeletal |
|  |  | CarP1560: | hypothetical protein |
|  |  | CarP1859: | metC |
|  |  | CarP1146: | hypothetical protein |
|  |  | CarP1330: | ychF |
|  |  | CarP1542: | bcp |
|  |  | CarP2630: | hypothetical protein |
|  |  | CarP1305: | mfd |
|  |  | CarP1405: | HIT-like protein |
|  |  | CarP1101: | priA |
|  |  | CarP1567: | disA |
|  |  | CarP1336: | N utilization substance protein B homolog |
|  |  | CarP1350: | lptC |
|  |  | CarP1262: | hup |
|  |  | CarP1863: | hypothetical protein |
|  |  | CarP1128: | mreB |
|  |  | CarP4755: | hypothetical protein |
|  |  | CarP3849: | hflK |
|  |  | CarP1389: | rpsA |
|  |  | CarP3151: | Ankyrinrepeats (3 copies) |
|  |  | CarP1426: | grpE |
|  |  | CarP1373: | comM |
|  |  | CarP1222: | sigA |
|  |  | CarP1118: | ideR |
|  |  | CarP1341: | dksA |
|  |  | CarP1348: | ndk |
|  |  | CarP1326: | lolC |
|  |  | CarP3800: | hypothetical protein |
|  |  | CarP1347: | clpY |
|  |  | CarP3095: | hypothetical protein |
|  |  | CarP4504: | hypothetical protein |
|  |  | CarP1074: | fabB |
|  |  | CarP1665: | murC |
|  |  | CarP1285: | rplP |
|  |  | CarP1406: | greA |
|  |  | CarP1104: | dnaB |
|  |  | CarP1661: | phr |
|  |  | CarP1302: | rho |
|  |  | CarP1410: | lolD |
|  |  | CarP1251: | rpsP |
|  |  | CarP1366: | hypothetical protein |
|  |  | CarP1489: | rplJ |
|  |  | CarP2552: | hypothetical protein |
|  |  | CarP1276: | rpsE |
|  |  | CarP2045: | PKD domain protein |
|  |  | CarP3805: | Putative multidrug export ATP-binding permease |
|  |  | CarP1368: | Aspartyl/glutamyl-tRNA(Asn/Gln) amidotransferase subunit B |
|  |  | CarP2637: | mntB |
|  |  | CarP2522: | Alpha beta hydrolase family protein |
|  |  | CarP1257: | alr |
|  |  | CarP1490: | rplA |
|  |  | CarP1468: | valS |
|  |  | CarP2036: | rtcB |
|  |  | CarP1495: | lptB |
|  |  | CarP3798: | hypothetical protein |
|  |  | CarP1301: | hisS |
|  |  | CarP3799: | hypothetical protein |
|  |  | CarP1545: | hypothetical protein |
|  |  | CarP2024: | ZIP Zinc transporter |
|  |  | CarP1479: | dbpA |
|  |  | CarP4710: | nudG |
|  |  | CarP2624: | ynaI |
|  |  | CarP1492: | nusG |
|  |  | CarP1412: | prsA |
|  |  | CarP1563: | narL |
|  |  | CarP2044: | pimB |
|  |  | CarP1248: | Cell division protein ZapA |
|  |  | CarP1375: | murE |
|  |  | CarP1526: | hypothetical protein |
|  |  | CarP1234: | rpsI |
|  |  | CarP4497: | spsI |
|  |  | CarP1130: | murA |
|  |  | CarP1399: | Rhomboid family protein |
|  |  | CarP1235: | rplM |
|  |  | CarP1659: | lipA |
|  |  | CarP1165: | truA |
|  |  | CarP2640: | hypothetical protein |
|  |  | CarP1324: | maeB |
|  |  | CarP1236: | exbD |
|  |  | CarP1360: | thrS |
|  |  | CarP1270: | rpsM |
|  |  | CarP6153: | Alpha beta hydrolase family protein |
|  |  | CarP1246: | rpsT |
|  |  | CarP1536: | acs |
|  |  | CarP1157: | efp |
|  |  | CarP1660: | tsaC |
|  |  | CarP1500: | hypothetical protein |
|  |  | CarP1478: | hypothetical protein |
|  |  | CarP3106: | ldh |
|  |  | CarP1129: | spoIIIE |
|  |  | CarP1174: | Acid shock protein |
|  |  | CarP1293: | fabD |
|  |  | CarP1258: | Septum formation initiator |
|  |  | CarP1125: | Colicin V production protein |
|  |  | CarP1486: | rpoC |
|  |  | CarP1300: | mraY |
|  |  | CarP1404: | typA |
|  |  | CarP1142: | phosphatidylserine decarboxylase |
|  |  | CarP3825: | ribN |
|  |  | CarP1458: | mnmE |
|  |  | CarP1344: | ComEC family competence protein |
|  |  | CarP1434: | gap |
|  |  | CarP3818: | hypothetical protein |
|  |  | CarP1921: | Bacterial regulatory protein, Fis family |
|  |  | CarP1411: | mutS |
|  |  | CarP1440: | era |
|  |  | CarP1395: | bamA |
|  |  | CarP3792: | hypothetical protein |
|  |  | CarP1192: | metN |
|  |  | CarP1269: | rpsK |
|  |  | CarP1363: | rplT |
|  |  | CarP1388: | lptD |
|  |  | CarP1480: | gatA |
|  |  | CarP1577: | putative rhodanese-related sulfurtransferase |
|  |  | CarP1422: | atpD |
|  |  | CarP1148: | rpsG |
|  |  | CarP1123: | uvrC |
|  |  | CarP1687: | Transposase DDE domain protein |
|  |  | CarP1361: | infC |
|  |  | CarP1420: | ksgA |
|  |  | CarP1565: | aceF |
|  |  | CarP1273: | secY |
|  |  | CarP1387: | holB |
|  |  | CarP1349: | pheS |
|  |  | CarP3119: | hypothetical protein |
|  |  | CarP3127: | hypothetical protein |
|  |  | CarP1474: | recA |
|  |  | CarP3131: | pcm |
|  |  | CarP2653: | SigmaW regulon antibacterial |
|  |  | CarP1470: | metG |
|  |  | CarP2973: | oppD |
|  |  | CarP1092: | lspA |
|  |  | CarP1446: | der |
|  |  | CarP1418: | gyrA |
|  |  | CarP2046: | ddpX |
|  |  | CarP1335: | YtxH-like protein |
|  |  | CarP1321: | groS5 |
|  |  | CarP2050: | smc |
|  |  | CarP1392: | miaA |
|  |  | CarP1312: | lpxA |
|  |  | CarP3820: | hypothetical protein |
|  |  | CarP1381: | hypothetical protein |
|  |  | CarP1131: | birA |
|  |  | CarP1919: | gsiC |
|  |  | CarP1365: | rpmG |
|  |  | CarP3842: | macA |
|  |  | CarP1400: | prfA |
|  |  | CarP1317: | dhfrIII |
|  |  | CarP3806: | hypothetical protein |
|  |  | CarP4746: | yidK |
|  |  | CarP1304: | cgtA |
|  |  | CarP1323: | dnaX |
|  |  | CarP1670: | Starch-binding associating with outer membrane |
|  |  | CarP2020: | PAAR motif protein |
|  |  | CarP1224: | iscS |
|  |  | CarP1255: | infB |
|  |  | CarP1431: | putative ABC transporter ATP-binding protein |
|  |  | CarP1186: | oppD |
|  |  | CarP4735: | rhtC |
|  |  | CarP1267: | rpoA |
|  |  | CarP3128: | Gene 25-like lysozyme |
|  |  | CarP1167: | sodA |
|  |  | CarP1473: | rnr |
|  |  | CarP1229: | hslV |
|  |  | CarP1460: | acpP |
|  |  | CarP1409: | rpmE2 |
|  |  | CarP1693: | spa |
|  |  | CarP1382: | Non-canonical purine NTP pyrophosphatase |
|  |  | CarP1487: | rpoB |
|  |  | CarP1380: | secG |
|  |  | CarP1189: | hypothetical protein |
|  |  | CarP1296: | yidC |
|  |  | CarP2002: | Baseplate J-like protein |
|  |  | CarP1156: | hypothetical protein |
|  |  | CarP1513: | norM |
|  |  | CarP1580: | trmB |
|  |  | CarP1376: | spoVD |
|  |  | CarP3789: | hypothetical protein |
|  |  | CarP1346: | lon2 |
|  |  | CarP1289: | rplB |
|  |  | CarP1408: | lpxB |
|  |  | CarP1337: | hypothetical protein |
|  |  | CarP1316: | uvrB |
|  |  | CarP1279: | rpsH |
|  |  | CarP1415: | Sporulation related domain protein |
|  |  | CarP1328: | fdx |
|  |  | CarP1126: | recG |
|  |  | CarP1173: | lgt |
|  |  | CarP1137: | putative permease YjgP YjgQ family protein |
|  |  | CarP1433: | prfB |
|  |  | CarP1244: | secD |
|  |  | CarP1338: | lpxK |
|  |  | CarP1231: | PPIC-type PPIASE domain protein |
|  |  | CarP1250: | rpmA |
|  |  | CarP2520: | tlcA |
|  |  | CarP1260: | DNA polymerase III subunit beta |
|  |  | CarP1402: | accC |
|  |  | CarP1332: | rplI |
|  |  | CarP1327: | hypothetical protein |
|  |  | CarP1864: | nudJ |
|  |  | CarP3829: | ddlB |
|  |  | CarP1307: | glyA |
|  |  | CarP1127: | glyQS |
|  |  | CarP1191: | lpxH |
|  |  | CarP1223: | trxB |
|  |  | CarP1501: | ruvA |
|  |  | CarP1303: | serS |
|  |  | CarP1582: | mscL |
|  |  | CarP1245: | S1 P1 Nuclease |
|  |  | CarP3132: | mutS2 |
|  |  | CarP1453: | rpb6 |
|  |  | CarP1025: | hlyB |
|  |  | CarP1232: | tsf |
|  |  | CarP1447: | sufB |
|  |  | CarP1096: | Putative 1,2-phenylacetyl-CoA epoxidase, s |
|  |  | CarP1026: | clpC |
|  |  | CarP1220: | rpsO |
|  |  | CarP1103: | htrB |
|  |  | CarP1238: | atpB |
|  |  | CarP2039: | rbn |
|  |  | CarP3797: | hypothetical protein |
|  |  | CarP1671: | lipB |
|  |  | CarP1441: | acpS |
|  |  | CarP1264: | ssb |
|  |  | CarP1070: | lepB |
|  |  | CarP1046: | inlJ |
|  |  | CarP1554: | rpsF |
|  |  | CarP1225: | prmB |
|  |  | CarP1120: | macB |
|  |  | CarP1266: | rplQ |
|  |  | CarP1602: | alkA |
|  |  | CarP1549: | pepP |
|  |  | CarP1543: | rpsL |
|  |  | CarP1111: | fbp |
|  |  | CarP3807: | amiD |
|  |  | CarP1385: | cysS |
|  |  | CarP6237: | yvdP |
|  |  | CarP1359: | nrdA |
|  |  | CarP1561: | miaB |
|  |  | CarP1358: | nrdB |
|  |  | CarP1416: | dus |
|  |  | CarP1098: | cca |
|  |  | CarP1432: | atpC |
|  |  | CarP1483: | yjjV |
|  |  | CarP1178: | leuS |
|  |  | CarP2040: | secE |
|  |  | CarP1391: | gyrB |
|  |  | CarP1105: | rpoN |
|  |  | CarP2049: | dacB |
|  |  | CarP1138: | dnaQ |
|  |  | CarP1566: | bfmBAB |
|  |  | CarP1423: | lepA |
|  |  | CarP1546: | rpsN2 |
|  |  | CarP2005: | nhaP2 |
|  |  | CarP1355: | mgtE |
|  |  | CarP1097: | DNA polymerase III subunit delta |
|  |  | CarP1572: | dapF |
|  |  | CarP1413: | Polysaccharide biosynthesis protein |
|  |  | CarP2652: | rmuC |
|  |  | CarP1688: | pld |
|  |  | CarP1466: | ftsA |
|  |  | CarP1320: | groEL |
|  |  | CarP1435: | putative aminodeoxychorismate lyase |
|  |  | CarP1054: | kinE |
|  |  | CarP1454: | bamA |
|  |  | CarP1241: | lolC |
|  |  | CarP1428: | ffh |
|  |  | CarP1297: | sufC |
|  |  | CarP1494: | thyA |
|  |  | CarP1467: | ftsZ |
|  |  | CarP1084: | hypothetical protein |
|  |  | CarP1364: | mltD |
|  |  | CarP1282: | rplN |
|  |  | CarP1449: | clpA |
|  |  | CarP1271: | infA |
|  |  | CarP1603: | hypothetical protein |
|  |  | CarP1135: | hypothetical protein |
|  |  | CarP1455: | lptD |
|  |  | CarP1372: | tyrS |
|  |  | CarP2987: | hypothetical protein |
|  |  | CarP1291: | rplD |
|  |  | CarP1287: | rplV |
|  |  | CarP1377: | ftsL |
|  |  | CarP3833: | spoIVFB |
|  |  | CarP1351: | dnaK |
|  |  | CarP3153: | yajL |
|  |  | CarP1143: | DNA polymerase I |
|  |  | CarP1014: | Integrase core domain protein |
|  |  | CarP1228: | putative permease YjgP YjgQ family protein |
|  |  | CarP2983: | hypothetical protein |
|  |  | CarP1497: | lipoprotein chaperone |
|  |  | CarP1481: | frr |
|  |  | CarP1396: | lrp |
|  |  | CarP1931: | T4-like virus tail tube protein gp19 |
|  |  | CarP1226: | ppx |
|  |  | CarP1116: | fadB |
|  |  | CarP1119: | tadA |
|  |  | CarP2019: | Phage tail sheath protein |
|  |  | CarP1331: | tRNA pseudouridine synthase B |
|  |  | CarP1275: | rpmD |
|  |  | CarP1576: | ATP-dependent helicase nuclease subunit A |
|  |  | CarP1233: | rpsB |
|  |  | CarP1862: | pyrG |
|  |  | CarP3846: | btrR |
|  |  | CarP1159: | fabB |
|  |  | CarP1292: | rplC |
|  |  | CarP1284: | rpmC |
|  |  | CarP1217: | rfbB |
|  |  | CarP1493: | elongation factor Tu |
|  |  | CarP1290: | rplW |
|  |  | CarP1443: | Undecaprenyl-phosphate mannosyltransferase |
|  |  | CarP1437: | Nucleoid-associated protein |
|  |  | CarP3843: | lnt |
|  |  | CarP1261: | rng |
|  |  | CarP1499: | hypothetical protein |
|  |  | CarP3155: | prpE |
|  |  | CarP1278: | rplF |
|  |  | CarP1071: | ykfA |
|  |  | CarP1369: | alaS |
|  |  | CarP1514: | metal-dependent hydrolase |
|  |  | CarP1485: | ligA |
|  |  | CarP1394: | aviRb |
|  |  | CarP1239: | atpF |
|  |  | CarP1571: | fadD |
|  |  | CarP1272: | map |
|  |  | CarP1169: | Putative multidrug export ATP-binding permease |
|  |  | CarP1556: | murI |
|  |  | CarP1456: | tilS |
|  |  | CarP1548: | tpiA |
|  |  | CarP3120: | hypothetical protein |
|  |  | CarP1102: | gpsA |
|  |  | CarP1553: | hemN |
|  |  | CarP1144: | bamA |
|  |  | CarP1150: | atpG |
|  |  | CarP1124: | hypothetical protein |
|  |  | CarP1379: | nrnA |
|  |  | CarP2008: | hypothetical protein |
|  |  | CarP1465: | hypothetical protein |
|  |  | CarP1502: | bamA |
|  |  | CarP1451: | tig |
|  |  | CarP1310: | ybeY |
|  |  | CarP1839: | rnhA |
|  |  | CarP1547: | mtaB |
|  |  | CarP1568: | rpmF |
|  |  | CarP1299: | rbfA |
|  |  | CarP1438: | murG |
|  |  | CarP3143: | bioA |
|  |  | CarP1614: | Transposase DDE domain protein |
|  |  | CarP1221: | pnp |
|  |  | CarP1457: | mnmA |
|  |  | CarP1308: | ptrA |
|  |  | CarP1263: | yfhQ |
|  |  | CarP2623: | mntB |
|  |  | CarP3142: | macB |
|  |  | CarP1219: | dnaA |
|  |  | CarP1151: | atpA |
|  |  | CarP1352: | rplY |
|  |  | CarP1362: | rpmI |
|  |  | CarP1673: | rpmH |
|  |  | CarP1378: | argS |
|  |  | CarP3788: | dcuA |
|  |  | CarP1448: | mutL |
|  |  | CarP1132: | Putative zinc metalloprotease |
|  |  | CarP1190: | ankX |
|  |  | CarP1357: | Peptidase M16 inactive domain protein |
|  |  | CarP1283: | rpsQ |
|  |  | CarP3832: | Putative reductase/y4119/YP 4011 |
|  |  | CarP1586: | ksgA |
|  |  | CarP1295: | ftsH |
|  |  | CarP1177: | rfbC |
|  |  | CarP1277: | rplR |
|  |  | CarP1414: | secA |
|  |  | CarP1200: | tuaD |
|  |  | CarP1429: | iscS |
|  |  | CarP1475: | yqgF |
|  |  | CarP1797: | arnT |
|  |  | CarP3809: | ypwA |
|  |  | CarP1922: | mutS |
|  |  | CarP1650: | yidD |
|  |  | CarP1651: | putative ABC transporter ATP-binding protein |
|  |  | CarP1334: | ung |
|  |  | CarP1149: | asnS |
|  |  | CarP4713: | hhoA |
|  |  | CarP1407: | pth |
|  |  | CarP1471: | recQ |
|  |  | CarP4726: | Lipid A export ATP-binding permease protei |
|  |  | CarP1133: | glgA |
|  |  | CarP1424: | rplS |
|  |  | CarP1136: | trmD |
|  |  | CarP1007: | Transposase DDE domain protein |
|  |  | CarP1401: | accB |
|  |  | CarP1550: | gpmI |
|  |  | CarP1314: | lpxD |
|  |  | CarP2639: | Phage late control gene D protein (GPD) |
|  |  | CarP1162: | ruvA |
|  |  | CarP2650: | citrate transporter |
|  |  | CarP1459: | rnc |
|  |  | CarP1345: | ksgA |
|  |  | CarP1315: | ddlA |
|  |  | CarP3096: | tolB |
|  |  | CarP1573: | murD |
|  |  | CarP1669: | putative inorganic polyphosphate/ATP-NAD kinase |
|  |  | CarP1469: | hypothetical protein |
|  |  | CarP1450: | clpP |
|  |  | CarP2032: | sugE |
|  |  | CarP1280: | rplE |
|  |  | CarP1442: | topA |
|  |  | CarP2626: | mepM |
|  |  | CarP1176: | dnaG |
|  |  | CarP1141: | gltX |
|  |  | CarP1436: | wbbL |
|  |  | CarP1288: | rpsS |
|  |  | CarP1106: | ruvC |
|  |  | CarP1139: | trpS |
|  |  | CarP1329: | hypothetical protein |
|  |  | CarP1541: | atpE |
|  |  | CarP1249: | rplU |
|  |  | CarP1555: | Dihydrolipoyl dehydrogenase |
|  |  | CarP1134: | ftsW |
|  |  | CarP1397: | Helix-hairpin-helix motif protein |
|  |  | CarP1298: | sufD |
|  |  | CarP1353: | tetratricopeptide repeat protein |
|  |  | CarP1286: | rpsC |
|  |  | CarP1477: | hypothetical protein |
|  |  | CarP1398: | rluA |
|  |  | CarP1439: | gyrB |
|  |  | CarP1559: | rpmB |
|  |  | CarP1658: | ftnA |
|  |  | CarP1227: | queuine tRNA-ribosyltransferase |
|  |  | CarP1393: | sppA |
|  |  | CarP1322: | accA |
|  |  | CarP1386: | gatA |
|  |  | CarP1294: | tadA |
|  |  | CarP1390: | hypothetical protein |
|  |  | CarP2986: | hypothetical protein |
|  |  | CarP1009: | formamidopyrimidine 5-formyluracil 5-hydr |
|  |  | CarP1544: | tsaD |
|  |  | CarP1461: | murF |
|  |  | CarP1562: | nth |
|  |  | CarP3819: | hypothetical protein |
|  |  | CarP1265: | smpB |
|  |  | CarP6235: | glpT |
|  |  | CarP1343: | ileS |
|  |  | CarP1498: | accA |
|  |  | CarP1147: | fus |
|  |  | CarP1569: | pgk |
|  |  | CarP1313: | lpxC |
|  |  | CarP1445: | Putative TrmH family tRNA rRNA methyltransferase |
|  |  | CarP1383: | engB |
|  |  | CarP1419: | minD |
|  |  | CarP1325: | tsaB |
|  |  | CarP2629: | hypothetical protein |
|  |  | CarP1256: | rpsU |
|  |  | CarP3147: | PQ loop repeat protein |
|  |  | CarP1425: | htpG |
|  |  | CarP1356: | Receptor family ligand binding region |
|  |  | CarP3836: | DinB superfamily protein |
|  |  | CarP1482: | phosphodiesterase |
|  |  | CarP1472: | gntX |
|  |  | CarP1552: | bfmBAB |
|  |  | CarP3138: | hypothetical protein |
|  |  | CarP1140: | hypothetical protein |
|  |  | CarP1011: | Bifunctional (p)ppGpp synthase hydrolase R |
|  |  | CarP1374: | rimM |
|  |  | CarP1835: | troA |
|  |  | CarP1578: | znuC |
|  |  | CarP1319: | parB |
|  |  | CarP1237: | tolQ |
|  |  | CarP2622: | hypothetical protein |
|  |  | CarP3817: | galE |
|  |  | CarP6148: | ribN |
|  |  | CarP1274: | rplO |
|  |  | CarP1370: | zntR |
|  |  | CarP3144: | bioD |
|  |  | CarP3647: | hypothetical protein |
|  |  | CarP1996: | ykgO |
|  |  | CarP1230: | hypothetical protein |
| "cardPLAS outgroupPangenome | " | 8 | CarP1655: |
|  |  | CarP1520: | hypothetical protein |
|  |  | CarP1751: | Transposase |
|  |  | CarP1076: | Transposase IS116 IS110 IS902 family protein |
|  |  | CarP6149: | hypothetical protein |
|  |  | CarP1654: | hypothetical protein |
|  |  | CarP1653: | hypothetical protein |
|  |  | CarP6150: | hypothetical protein |
| CardiniumPangenome cardPLAS | 15 | CarP6172: | hypothetical protein |
|  |  | CarP6175: | hypothetical protein |
|  |  | CarP1060: | Transposase DDE domain protein |
|  |  | CarP3831: | wapA |
|  |  | CarP6180: | Transposase, Mutator family |
|  |  | CarP6178: | hypothetical protein |
|  |  | CarP2977: | hypothetical protein |
|  |  | CarP4722: | bin3 |
|  |  | CarP3618: | Integrase core domain protein |
|  |  | CarP1691: | hypothetical protein |
|  |  | CarP6181: | hypothetical protein |
|  |  | CarP6177: | hypothetical protein |
|  |  | CarP4495: | SMI1 KNR4 family protein |
|  |  | CarP6176: | hypothetical protein |
|  |  | CarP4490: | Ankyrinrepeats (3 copies) |
| outgroupPLAS | 26 | CarP7496: | hypothetical protein |
|  |  | CarP7775: | hypothetical protein |
|  |  | CarP5672: | hypothetical protein |
|  |  | CarP7498: | traO |
|  |  | CarP7761: | hypothetical protein |
|  |  | CarP7762: | hypothetical protein |
|  |  | CarP5668: | hypothetical protein |
|  |  | CarP1181: | Initiator Replication protein |
|  |  | CarP7759: | class III cytochrome C family protein |
|  |  | CarP6043: | molybdate ABC transporter periplasmic moly |
|  |  | CarP6042: | hypothetical protein |
|  |  | CarP7130: | hypothetical protein |
|  |  | CarP4709: | xerC |
|  |  | CarP7776: | hypothetical protein |
|  |  | CarP7758: | hypothetical protein |
|  |  | CarP5994: | Metallo-beta-lactamase superfamily protein |
|  |  | CarP7195: | tam |
|  |  | CarP5671: | hypothetical protein |
|  |  | CarP7385: | hypothetical protein |
|  |  | CarP5344: | hypothetical protein |
|  |  | CarP7506: | hypothetical protein |
|  |  | CarP7497: | hypothetical protein |
|  |  | CarP7778: | hypothetical protein |
|  |  | CarP7760: | hypothetical protein |
|  |  | CarP4482: | hypothetical protein |
|  |  | CarP5669: | hypothetical protein |
| outgroupPangenome | 5646 | CarP5294: | Outer membrane efflux protein |
|  |  | CarP6554: | hypothetical protein |
|  |  | CarP5792: | hypothetical protein |
|  |  | CarP3233: | Cytochrome c oxidase subunit III |
|  |  | CarP5561: | 18 kDa heat shock protein |
|  |  | CarP3359: | pglC |
|  |  | CarP7320: | novN |
|  |  | CarP2340: | prephenate dehydrogenase |
|  |  | CarP2067: | iaaA |
|  |  | CarP4558: | hypothetical protein |
|  |  | CarP3918: | sbcD |
|  |  | CarP4354: | DinB family protein |
|  |  | CarP5783: | Calcineurin-like phosphoesterase |
|  |  | CarP7435: | cry2 |
|  |  | CarP3158: | FtsX-like permease family protein |
|  |  | CarP6985: | Macrolide export ATP-binding permease prot |
|  |  | CarP1796: | polX |
|  |  | CarP7127: | putative ABC transporter ATP-binding protein |
|  |  | CarP7007: | trxB |
|  |  | CarP2158: | rocA1 |
|  |  | CarP1881: | fecR |
|  |  | CarP5784: | hypothetical protein |
|  |  | CarP3166: | dinB |
|  |  | CarP3769: | Thermophilic serine proteinase precursor |
|  |  | CarP1610: | cycA |
|  |  | CarP5358: | Caspase domain protein |
|  |  | CarP4617: | hypothetical protein |
|  |  | CarP6064: | arlS |
|  |  | CarP5231: | hypothetical protein |
|  |  | CarP1789: | dapE |
|  |  | CarP5804: | atsA |
|  |  | CarP1606: | CAAX amino terminal protease self- immunity |
|  |  | CarP3373: | hypothetical protein |
|  |  | CarP3666: | hypothetical protein |
|  |  | CarP2229: | pdxJ |
|  |  | CarP2542: | soluble pyridine nucleotide transhydrogena |
|  |  | CarP2436: | hypothetical protein |
|  |  | CarP3713: | bglX |
|  |  | CarP2690: | exuT |
|  |  | CarP2352: | von Willebrand factor type A domain protei |
|  |  | CarP5916: | Alpha beta hydrolase family protein |
|  |  | CarP7263: | hypothetical protein |
|  |  | CarP5062: | atsA |
|  |  | CarP3444: | AAA-like domain protein |
|  |  | CarP2299: | leuB |
|  |  | CarP6702: | yrrB |
|  |  | CarP2663: | Putative multidrug export ATP-binding perm |
|  |  | CarP7428: | Sulfotransferase domain protein |
|  |  | CarP5512: | hypothetical protein |
|  |  | CarP1108: | ybbD |
|  |  | CarP4348: | NUDIX domain protein |
|  |  | CarP3428: | yteT |
|  |  | CarP1912: | GH3 auxin-responsive promoter |
|  |  | CarP2548: | hypothetical protein |
|  |  | CarP5925: | fecR |
|  |  | CarP1613: | Protein of unknown function (Porph ging) |
|  |  | CarP5922: | hypothetical protein |
|  |  | CarP3472: | bfce |
|  |  | CarP4185: | mprA |
|  |  | CarP2756: | Amidohydrolase |
|  |  | CarP1202: | NADH dehydrogenase-like protein |
|  |  | CarP5459: | sodA |
|  |  | CarP4943: | Endoribonuclease L-PSP |
|  |  | CarP2841: | lpxH |
|  |  | CarP1622: | pupA |
|  |  | CarP6617: | fhuA |
|  |  | CarP2176: | tetratricopeptide repeat protein |
|  |  | CarP7037: | hypothetical protein |
|  |  | CarP2108: | hemE |
|  |  | CarP7575: | hypothetical protein |
|  |  | CarP5872: | hypothetical protein |
|  |  | CarP6881: | metal-dependent hydrolase |
|  |  | CarP6422: | Trehalose utilisation |
|  |  | CarP1791: | hypothetical protein |
|  |  | CarP6638: | dinB |
|  |  | CarP2065: | etfB |
|  |  | CarP4951: | Bacterial alpha-L-rhamnosidase |
|  |  | CarP5490: | hypothetical protein |
|  |  | CarP4982: | hypothetical protein |
|  |  | CarP4526: | Polysaccharide biosynthesis export protein |
|  |  | CarP6560: | Sulfotransferase domain protein |
|  |  | CarP4847: | Flavin reductase like domain protein |
|  |  | CarP2823: | icaA |
|  |  | CarP4370: | hypothetical protein |
|  |  | CarP1877: | ramA |
|  |  | CarP7467: | hypothetical protein |
|  |  | CarP2330: | nadD |
|  |  | CarP4568: | HEPN domain protein |
|  |  | CarP7510: | hepA |
|  |  | CarP5903: | fecR |
|  |  | CarP3507: | kanE |
|  |  | CarP4309: | cfr |
|  |  | CarP5776: | hypothetical protein |
|  |  | CarP6856: | yiaD |
|  |  | CarP3951: | SusD family protein |
|  |  | CarP4036: | ltaS2 |
|  |  | CarP6065: | Transcriptional regulator PadR-like family |
|  |  | CarP5821: | anaerobic benzoate catabolism transcriptional regulator |
|  |  | CarP2183: | Ycf48-like protein precursor |
|  |  | CarP5170: | betA |
|  |  | CarP3776: | hypothetical protein |
|  |  | CarP7685: | hypothetical protein |
|  |  | CarP5219: | hypothetical protein |
|  |  | CarP7696: | DNA-binding ATP-dependent protease |
|  |  | CarP7693: | Glycosyl hydrolases family 43 |
|  |  | CarP3686: | hypothetical protein |
|  |  | CarP3265: | hyi |
|  |  | CarP1766: | hypothetical protein |
|  |  | CarP3946: | afr |
|  |  | CarP2455: | maf |
|  |  | CarP4923: | pdxH |
|  |  | CarP5441: | hisC2 |
|  |  | CarP4000: | hypothetical protein |
|  |  | CarP2602: | divJ |
|  |  | CarP7302: | hypothetical protein |
|  |  | CarP3929: | Glycosyl hydrolases family 43 |
|  |  | CarP3558: | paaF |
|  |  | CarP5128: | hypothetical protein |
|  |  | CarP2374: | ybgC |
|  |  | CarP6018: | hypothetical protein |
|  |  | CarP6493: | hypothetical protein |
|  |  | CarP6885: | hypothetical protein |
|  |  | CarP3670: | hypothetical protein |
|  |  | CarP6878: | poxB |
|  |  | CarP4139: | hypothetical protein |
|  |  | CarP1695: | hypothetical protein |
|  |  | CarP5912: | hypothetical protein |
|  |  | CarP7022: | chbR |
|  |  | CarP6415: | rffA |
|  |  | CarP3271: | ksgA |
|  |  | CarP6023: | evgA |
|  |  | CarP5264: | hypothetical protein |
|  |  | CarP5094: | clcB |
|  |  | CarP1652: | hypothetical protein |
|  |  | CarP6672: | hypothetical protein |
|  |  | CarP5625: | hypothetical protein |
|  |  | CarP7106: | hypothetical protein |
|  |  | CarP6631: | Phosphotransferase enzyme family protein |
|  |  | CarP4822: | ampC |
|  |  | CarP4076: | ramA |
|  |  | CarP3252: | NHL repeat protein |
|  |  | CarP2154: | cysG |
|  |  | CarP7379: | tnaA1 |
|  |  | CarP5897: | AAA-like domain protein |
|  |  | CarP3758: | Auracyanin-A precursor |
|  |  | CarP1935: | TonB dependent receptor |
|  |  | CarP5126: | hypothetical protein |
|  |  | CarP2333: | carA |
|  |  | CarP6076: | hypothetical protein |
|  |  | CarP7149: | proA |
|  |  | CarP2209: | dxs |
|  |  | CarP1857: | dapA |
|  |  | CarP5771: | Amidohydrolase |
|  |  | CarP4466: | hypothetical protein |
|  |  | CarP3140: | Sodium glucose cotransporter |
|  |  | CarP5486: | FIST N domain protein |
|  |  | CarP4140: | hypothetical protein |
|  |  | CarP4369: | WbqC-like protein family protein |
|  |  | CarP3721: | hypothetical protein |
|  |  | CarP2219: | gltB |
|  |  | CarP3237: | Type I phosphodiesterase nucleotide pyro |
|  |  | CarP6513: | metH |
|  |  | CarP2168: | ksgA |
|  |  | CarP5519: | hypothetical protein |
|  |  | CarP6340: | fixL |
|  |  | CarP5556: | hypothetical protein |
|  |  | CarP6515: | UDP-D-galactose:(glucosyl)lipopolysacchari |
|  |  | CarP4605: | hypothetical protein |
|  |  | CarP6362: | betI |
|  |  | CarP4484: | hypothetical protein |
|  |  | CarP1187: | hypothetical protein |
|  |  | CarP4975: | btuB |
|  |  | CarP4175: | dgoA |
|  |  | CarP2180: | hypothetical protein |
|  |  | CarP6674: | hypothetical protein |
|  |  | CarP1750: | sigM |
|  |  | CarP2148: | allB |
|  |  | CarP7342: | ycnE |
|  |  | CarP7118: | fabB |
|  |  | CarP5791: | atsA |
|  |  | CarP3314: | gtf1 |
|  |  | CarP2811: | vioA |
|  |  | CarP6464: | hypothetical protein |
|  |  | CarP1626: | hypothetical protein |
|  |  | CarP7219: | hypothetical protein |
|  |  | CarP4611: | hypothetical protein |
|  |  | CarP3921: | besA |
|  |  | CarP3035: | hypothetical protein |
|  |  | CarP7746: | hypothetical protein |
|  |  | CarP7226: | hypothetical protein |
|  |  | CarP4375: | yteR |
|  |  | CarP6869: | Fatty acid desaturase |
|  |  | CarP6866: | psaB |
|  |  | CarP2566: | btuB |
|  |  | CarP1847: | dfrA |
|  |  | CarP4529: | hypothetical protein |
|  |  | CarP5839: | hypothetical protein |
|  |  | CarP4933: | hypothetical protein |
|  |  | CarP5476: | nfi |
|  |  | CarP1884: | btuB |
|  |  | CarP7276: | hypothetical protein |
|  |  | CarP3854: | hypothetical protein |
|  |  | CarP2462: | nagK |
|  |  | CarP7515: | hypothetical protein |
|  |  | CarP2617: | nuoN |
|  |  | CarP4716: | anmK |
|  |  | CarP2278: | ctaA |
|  |  | CarP6602: | hypothetical protein |
|  |  | CarP5840: | Acyltransferase family protein |
|  |  | CarP6826: | hypothetical protein |
|  |  | CarP2375: | galE |
|  |  | CarP4404: | Barstar (barnase inhibitor) |
|  |  | CarP2083: | VanZ like family protein |
|  |  | CarP4082: | Autoinducer 2 sensor kinase phosphatase Lu |
|  |  | CarP3690: | sdpR |
|  |  | CarP7701: | hypothetical protein |
|  |  | CarP5827: | hypothetical protein |
|  |  | CarP3483: | hypothetical protein |
|  |  | CarP5774: | yiaM |
|  |  | CarP4905: | hypothetical protein |
|  |  | CarP4304: | hypothetical protein |
|  |  | CarP7603: | Putative aminoglycoside phosphotransferase |
|  |  | CarP2473: | asnC |
|  |  | CarP5167: | fosA |
|  |  | CarP1807: | putative metallo-hydrolase |
|  |  | CarP4211: | glpQ1 |
|  |  | CarP3346: | NADH-quinone oxidoreductase subunit 12 |
|  |  | CarP2485: | hypothetical protein |
|  |  | CarP3599: | hypothetical protein |
|  |  | CarP3038: | hypothetical protein |
|  |  | CarP1746: | rmlA1 |
|  |  | CarP3309: | rhaM |
|  |  | CarP4959: | MORN repeat variant |
|  |  | CarP5227: | hypothetical protein |
|  |  | CarP3392: | fepA |
|  |  | CarP7434: | alkB2 |
|  |  | CarP6391: | hypothetical protein |
|  |  | CarP4067: | nucleoside diphosphate kinase regulator |
|  |  | CarP3145: | kbl |
|  |  | CarP1590: | heat-inducible protein |
|  |  | CarP3026: | Transglutaminase-like superfamily protein |
|  |  | CarP1685: | hypothetical protein |
|  |  | CarP7651: | hypothetical protein |
|  |  | CarP2740: | Serine threonine-protein kinase pkn1 |
|  |  | CarP4411: | hypothetical protein |
|  |  | CarP3743: | tagA |
|  |  | CarP5351: | arlS |
|  |  | CarP1527: | rimI |
|  |  | CarP6141: | hypothetical protein |
|  |  | CarP4049: | Bacterial leucyl aminopeptidase precursor |
|  |  | CarP6544: | hypothetical protein |
|  |  | CarP4956: | xerD |
|  |  | CarP4422: | hypothetical protein |
|  |  | CarP3976: | tsaC1 |
|  |  | CarP4950: | Phosphate-selective porin O and P |
|  |  | CarP3200: | Ribonuclease |
|  |  | CarP6718: | hypothetical protein |
|  |  | CarP3985: | hypothetical protein |
|  |  | CarP5214: | qdoI |
|  |  | CarP3533: | crcB |
|  |  | CarP7309: | Phage integrase family protein |
|  |  | CarP2560: | hypothetical protein |
|  |  | CarP3112: | cspC |
|  |  | CarP7307: | todS |
|  |  | CarP5197: | hypothetical protein |
|  |  | CarP4264: | kdpB |
|  |  | CarP7744: | yiaD |
|  |  | CarP1812: | hypothetical protein |
|  |  | CarP2142: | iap |
|  |  | CarP1979: | pepO |
|  |  | CarP7519: | hypothetical protein |
|  |  | CarP5881: | Ferric uptake regulator family protein |
|  |  | CarP1961: | spoIIE |
|  |  | CarP7665: | btrR |
|  |  | CarP1865: | fdtB |
|  |  | CarP6129: | Stress-induced bacterial acidophilic repea |
|  |  | CarP5472: | ygiC |
|  |  | CarP3067: | hypothetical protein |
|  |  | CarP6247: | HI0933-like protein |
|  |  | CarP1712: | yesX |
|  |  | CarP7331: | 3D-(3,5 4)-trihydroxycyclohexane-1,2-dione |
|  |  | CarP6609: | Fatty acid hydroxylase superfamily protein |
|  |  | CarP4223: | wbbL |
|  |  | CarP3750: | btr |
|  |  | CarP7243: | hypothetical protein |
|  |  | CarP1963: | galE |
|  |  | CarP7017: | yehU |
|  |  | CarP6500: | hypothetical protein |
|  |  | CarP6256: | ptlH |
|  |  | CarP3445: | Helix-turn-helix domain protein |
|  |  | CarP7633: | yiaD |
|  |  | CarP7278: | hypothetical protein |
|  |  | CarP6950: | ATP10 protein |
|  |  | CarP5260: | xynB |
|  |  | CarP2069: | thrC |
|  |  | CarP3171: | 2OG-Fe(II) oxygenase superfamily protein |
|  |  | CarP4662: | hypothetical protein |
|  |  | CarP2997: | atsA |
|  |  | CarP2252: | hypothetical protein |
|  |  | CarP7333: | mmsA |
|  |  | CarP2139: | ilvE |
|  |  | CarP7304: | hypothetical protein |
|  |  | CarP6389: | Non-canonical purine NTP phosphatase |
|  |  | CarP4015: | cmpR |
|  |  | CarP1765: | fabI |
|  |  | CarP2281: | qoxC |
|  |  | CarP6447: | hypothetical protein |
|  |  | CarP4396: | sigW |
|  |  | CarP3279: | UbiA prenyltransferase family protein |
|  |  | CarP3685: | ysnE |
|  |  | CarP4797: | Auracyanin-B precursor |
|  |  | CarP3269: | dhaA |
|  |  | CarP2216: | serC |
|  |  | CarP3008: | hypothetical protein |
|  |  | CarP5025: | hypothetical protein |
|  |  | CarP6792: | ybaL |
|  |  | CarP4181: | nanM |
|  |  | CarP3214: | Putative lumazine-binding protein |
|  |  | CarP2288: | FKBP-type peptidyl-prolyl cis-trans isomerase |
|  |  | CarP4120: | formate dehydrogenase accessory protein |
|  |  | CarP7205: | putative glycosyl transferase |
|  |  | CarP2703: | ilvA |
|  |  | CarP6347: | hypothetical protein |
|  |  | CarP6031: | hypothetical protein |
|  |  | CarP7089: | btuB |
|  |  | CarP5942: | hypothetical protein |
|  |  | CarP6388: | trxA |
|  |  | CarP6051: | hypothetical protein |
|  |  | CarP4109: | fadM |
|  |  | CarP6302: | kfoC |
|  |  | CarP3479: | kynA |
|  |  | CarP5738: | hypothetical protein |
|  |  | CarP5885: | ABC-2 family transporter protein |
|  |  | CarP3354: | apxIB |
|  |  | CarP5866: | hypothetical protein |
|  |  | CarP4730: | Transposase IS200 like protein |
|  |  | CarP4635: | hypothetical protein |
|  |  | CarP2140: | btuR |
|  |  | CarP4539: | CHAT domain protein |
|  |  | CarP6348: | merA |
|  |  | CarP5798: | hypothetical protein |
|  |  | CarP5299: | zraR |
|  |  | CarP7547: | sdrD |
|  |  | CarP5376: | Starch-binding associating with outer memb |
|  |  | CarP4594: | hypothetical protein |
|  |  | CarP7388: | Cupin domain protein |
|  |  | CarP4104: | vapC |
|  |  | CarP3576: | hypothetical protein |
|  |  | CarP6848: | Methyltransferase domain protein |
|  |  | CarP3907: | BlaR1 peptidase M56 |
|  |  | CarP3015: | hypothetical protein |
|  |  | CarP2952: | acn |
|  |  | CarP4259: | ftrB |
|  |  | CarP6029: | Helix-turn-helix |
|  |  | CarP7170: | Xylose isomerase-like TIM barrel |
|  |  | CarP6373: | ydhP |
|  |  | CarP3073: | cirA |
|  |  | CarP6930: | hypothetical protein |
|  |  | CarP5953: | ISXO2-like transposase domain protein |
|  |  | CarP5331: | malL |
|  |  | CarP7021: | abn-ts |
|  |  | CarP6831: | arsC |
|  |  | CarP3661: | amt |
|  |  | CarP3795: | azo1 |
|  |  | CarP3697: | Peptidase family S41 |
|  |  | CarP5229: | hypothetical protein |
|  |  | CarP3926: | tlyC |
|  |  | CarP5008: | Fatty acid hydroxylase superfamily protein |
|  |  | CarP7520: | ptpA |
|  |  | CarP3972: | exonuclease V subunit alpha |
|  |  | CarP5499: | nirM |
|  |  | CarP1774: | ABC-2 family transporter protein |
|  |  | CarP4220: | icaA |
|  |  | CarP7635: | hypothetical protein |
|  |  | CarP3552: | hypothetical protein |
|  |  | CarP3323: | cpnA |
|  |  | CarP5555: | hypothetical protein |
|  |  | CarP7275: | hypothetical protein |
|  |  | CarP5576: | hypothetical protein |
|  |  | CarP2776: | glpK |
|  |  | CarP7559: | cirA |
|  |  | CarP4587: | hypothetical protein |
|  |  | CarP6059: | wbbL |
|  |  | CarP4941: | Zinc carboxypeptidase |
|  |  | CarP3741: | rcoM1 |
|  |  | CarP2470: | hisB |
|  |  | CarP2101: | hypothetical protein |
|  |  | CarP6369: | atoE |
|  |  | CarP4673: | hypothetical protein |
|  |  | CarP1702: | atsA |
|  |  | CarP1678: | degU |
|  |  | CarP3655: | Outer membrane efflux protein |
|  |  | CarP2356: | hisF |
|  |  | CarP4936: | ispH |
|  |  | CarP6979: | hypothetical protein |
|  |  | CarP6528: | Abi-like protein |
|  |  | CarP2370: | queE |
|  |  | CarP6671: | hypothetical protein |
|  |  | CarP4168: | araC |
|  |  | CarP5633: | hypothetical protein |
|  |  | CarP7638: | hypothetical protein |
|  |  | CarP2578: | hsaD |
|  |  | CarP5361: | hypothetical protein |
|  |  | CarP3752: | nagA |
|  |  | CarP5338: | Stress responsive A B Barrel Domain protei |
|  |  | CarP2529: | qorA |
|  |  | CarP6643: | parE1 |
|  |  | CarP4471: | ABC-2 family transporter protein |
|  |  | CarP5584: | xynC |
|  |  | CarP3857: | CRISPR associated protein Cas6 |
|  |  | CarP5393: | pepP |
|  |  | CarP3668: | hrpB |
|  |  | CarP6976: | hypothetical protein |
|  |  | CarP2834: | hypothetical protein |
|  |  | CarP6534: | NAD dependent epimerase dehydratase family |
|  |  | CarP3780: | recombination protein F |
|  |  | CarP6935: | bfmBAB |
|  |  | CarP6699: | ilvE |
|  |  | CarP5077: | soluble pyridine nucleotide transhydrogena |
|  |  | CarP6918: | hypothetical protein |
|  |  | CarP3932: | hypothetical protein |
|  |  | CarP4094: | hypothetical protein |
|  |  | CarP1879: | cymR |
|  |  | CarP6588: | alsT |
|  |  | CarP3007: | hypothetical protein |
|  |  | CarP6816: | yliI |
|  |  | CarP6093: | hypothetical protein |
|  |  | CarP4199: | btuB |
|  |  | CarP3367: | yumC |
|  |  | CarP6393: | PRC-barrel domain protein |
|  |  | CarP5878: | hypothetical protein |
|  |  | CarP3730: | per1 |
|  |  | CarP3589: | Glyoxalase Bleomycin resistance protein Di |
|  |  | CarP7663: | hypothetical protein |
|  |  | CarP3400: | Beta-monoglucosyldiacylglycerol synthase |
|  |  | CarP4666: | putative two-component response-regulatory |
|  |  | CarP4291: | Fructosamine kinase |
|  |  | CarP3604: | Zinc carboxypeptidase |
|  |  | CarP7269: | hypothetical protein |
|  |  | CarP3884: | Glyoxalase-like domain protein |
|  |  | CarP4514: | hepA |
|  |  | CarP5622: | yteR |
|  |  | CarP4559: | hypothetical protein |
|  |  | CarP5685: | ankX |
|  |  | CarP5151: | uxuA |
|  |  | CarP2428: | hypothetical protein |
|  |  | CarP7076: | Glycosyl hydrolases family 43 |
|  |  | CarP2628: | nuoK |
|  |  | CarP4688: | cirA |
|  |  | CarP3657: | hypothetical protein |
|  |  | CarP6277: | ykuT |
|  |  | CarP5494: | hypothetical protein |
|  |  | CarP1843: | recQ |
|  |  | CarP2126: | hypothetical protein |
|  |  | CarP6505: | SusD family protein |
|  |  | CarP7579: | galD |
|  |  | CarP5362: | Xylose isomerase-like TIM barrel |
|  |  | CarP6321: | trpB |
|  |  | CarP4550: | hypothetical protein |
|  |  | CarP2329: | gmk |
|  |  | CarP6657: | fldA |
|  |  | CarP3443: | Biopolymer transport protein ExbD TolR |
|  |  | CarP3839: | ppsA |
|  |  | CarP3202: | pglA |
|  |  | CarP7629: | lytR |
|  |  | CarP3858: | DinB family protein |
|  |  | CarP2475: | ybaQ |
|  |  | CarP2824: | ansA |
|  |  | CarP6775: | hypothetical protein |
|  |  | CarP5750: | hypothetical protein |
|  |  | CarP5070: | nasB |
|  |  | CarP4825: | hypothetical protein |
|  |  | CarP4706: | RNase III inhibitor |
|  |  | CarP6440: | hypothetical protein |
|  |  | CarP2167: | suhB |
|  |  | CarP1113: | hypothetical protein |
|  |  | CarP2880: | hypothetical protein |
|  |  | CarP5452: | Acyltransferase family protein |
|  |  | CarP3078: | MOSC domain protein |
|  |  | CarP2903: | hypothetical protein |
|  |  | CarP5838: | hypothetical protein |
|  |  | CarP6713: | putative 3-hydroxyphenylpropionic transpor |
|  |  | CarP2011: | erpA |
|  |  | CarP6971: | hypothetical protein |
|  |  | CarP2837: | pepN |
|  |  | CarP4642: | hypothetical protein |
|  |  | CarP4600: | hypothetical protein |
|  |  | CarP3235: | putative adenylyltransferase sulfurtransfe |
|  |  | CarP7417: | hypothetical protein |
|  |  | CarP2862: | CHRD domain protein |
|  |  | CarP2802: | ppc |
|  |  | CarP2544: | hypothetical protein |
|  |  | CarP2296: | ilvC |
|  |  | CarP3199: | gumD |
|  |  | CarP1769: | ilvB |
|  |  | CarP5656: | Metallopeptidase family M24 |
|  |  | CarP7159: | uidA |
|  |  | CarP1803: | hypothetical protein |
|  |  | CarP3716: | bsn |
|  |  | CarP7203: | nrdZ |
|  |  | CarP4705: | hypothetical protein |
|  |  | CarP4258: | hypothetical protein |
|  |  | CarP7223: | Enamine imine deaminase |
|  |  | CarP4548: | hypothetical protein |
|  |  | CarP1966: | hypothetical protein |
|  |  | CarP7281: | tkt |
|  |  | CarP6022: | hypothetical protein |
|  |  | CarP2197: | aroE |
|  |  | CarP4218: | Glucose Sorbosone dehydrogenase |
|  |  | CarP1003: | yknZ |
|  |  | CarP3368: | LVIVD repeat protein |
|  |  | CarP2965: | ybgF |
|  |  | CarP3054: | hypothetical protein |
|  |  | CarP1944: | SusD family protein |
|  |  | CarP3601: | nadB |
|  |  | CarP5289: | Peptidase S46 |
|  |  | CarP4241: | hypothetical protein |
|  |  | CarP4707: | glmU |
|  |  | CarP7512: | BNR Asp-box repeat protein |
|  |  | CarP2917: | tuaC |
|  |  | CarP5320: | hypothetical protein |
|  |  | CarP7172: | hypothetical protein |
|  |  | CarP6319: | hypothetical protein |
|  |  | CarP4802: | dhaA |
|  |  | CarP1845: | recX |
|  |  | CarP6364: | resA |
|  |  | CarP5967: | abfB |
|  |  | CarP4394: | arnC |
|  |  | CarP4414: | tetratricopeptide repeat protein |
|  |  | CarP7509: | Lipid A export ATP-binding permease protei |
|  |  | CarP7231: | hypothetical protein |
|  |  | CarP6332: | hypothetical protein |
|  |  | CarP4643: | hypothetical protein |
|  |  | CarP2918: | uxaB |
|  |  | CarP5939: | rcp1 |
|  |  | CarP2454: | Putative pyridoxal phosphate-dependent acy |
|  |  | CarP2635: | ndhI |
|  |  | CarP3111: | hypothetical protein |
|  |  | CarP2972: | hypothetical protein |
|  |  | CarP5741: | hypothetical protein |
|  |  | CarP1630: | ycjS |
|  |  | CarP3712: | hypothetical protein |
|  |  | CarP5423: | hypothetical protein |
|  |  | CarP2327: | hypothetical protein |
|  |  | CarP2137: | queD |
|  |  | CarP4222: | O-Antigen ligase |
|  |  | CarP5019: | Fatty acid hydroxylase superfamily protein |
|  |  | CarP2774: | hypothetical protein |
|  |  | CarP7698: | Eco57I restriction-modification methylase |
|  |  | CarP5168: | Glyoxalase-like domain protein |
|  |  | CarP2098: | hemF |
|  |  | CarP4338: | Carboxynorspermidine carboxyspermidine dec |
|  |  | CarP6892: | hypothetical protein |
|  |  | CarP4711: | apbE |
|  |  | CarP3029: | FAD dependent oxidoreductase |
|  |  | CarP3640: | hepC |
|  |  | CarP1805: | hypothetical protein |
|  |  | CarP4186: | topB |
|  |  | CarP5143: | Restriction endonuclease |
|  |  | CarP4931: | hypothetical protein |
|  |  | CarP6583: | sasA |
|  |  | CarP3636: | PKD domain protein |
|  |  | CarP6684: | hypothetical protein |
|  |  | CarP6654: | gno |
|  |  | CarP3501: | hypothetical protein |
|  |  | CarP5246: | hypothetical protein |
|  |  | CarP3766: | hypothetical protein |
|  |  | CarP2085: | hypothetical protein |
|  |  | CarP6915: | novP |
|  |  | CarP5181: | cycA |
|  |  | CarP2994: | Cytochrome c |
|  |  | CarP5195: | hypothetical protein |
|  |  | CarP2814: | TVP38 TMEM64 family inner membrane protein |
|  |  | CarP2704: | purU |
|  |  | CarP6851: | RHS Repeat protein |
|  |  | CarP7668: | hypothetical protein |
|  |  | CarP6545: | doxX |
|  |  | CarP7749: | hypothetical protein |
|  |  | CarP2408: | btuB |
|  |  | CarP6000: | Transposase |
|  |  | CarP3197: | Chain length determinant protein |
|  |  | CarP6862: | Sodium proton antiporter ChaA |
|  |  | CarP4432: | SCP-2 sterol transfer family protein |
|  |  | CarP3416: | Amidohydrolase |
|  |  | CarP4518: | betC |
|  |  | CarP7222: | hypothetical protein |
|  |  | CarP3108: | hypothetical protein |
|  |  | CarP6608: | Calcineurin-like phosphoesterase superfami |
|  |  | CarP3134: | vapC |
|  |  | CarP4212: | dnaE2 |
|  |  | CarP2303: | mdh |
|  |  | CarP5467: | hypothetical protein |
|  |  | CarP5282: | chrA |
|  |  | CarP3782: | abfB |
|  |  | CarP3000: | axe1 |
|  |  | CarP7628: | hypothetical protein |
|  |  | CarP6386: | hypothetical protein |
|  |  | CarP4461: | NADP-dependent 7-alpha-hydroxysteroid dehy |
|  |  | CarP5662: | fabG |
|  |  | CarP7522: | hypothetical protein |
|  |  | CarP6394: | hypothetical protein |
|  |  | CarP3747: | TonB-dependent Receptor Plug Domain protei |
|  |  | CarP1721: | Sodium Bile acid symporter family protein |
|  |  | CarP5140: | cnrB |
|  |  | CarP4048: | DinB family protein |
|  |  | CarP5644: | hypothetical protein |
|  |  | CarP1197: | rcp1 |
|  |  | CarP7061: | hypothetical protein |
|  |  | CarP2224: | ohrR |
|  |  | CarP2695: | prfC |
|  |  | CarP7211: | hypothetical protein |
|  |  | CarP5470: | hypothetical protein |
|  |  | CarP6140: | putative kinase inhibitor protein |
|  |  | CarP6154: | vraS |
|  |  | CarP2088: | yycF |
|  |  | CarP4044: | SnoaL-like domain protein |
|  |  | CarP7123: | hypothetical protein |
|  |  | CarP6017: | hypothetical protein |
|  |  | CarP4958: | hypothetical protein |
|  |  | CarP3729: | thiol-disulfide oxidoreductase |
|  |  | CarP3816: | hypothetical protein |
|  |  | CarP7306: | hypothetical protein |
|  |  | CarP5597: | hypothetical protein |
|  |  | CarP4403: | exsA |
|  |  | CarP3559: | hypothetical protein |
|  |  | CarP6116: | TonB-dependent Receptor Plug Domain protei |
|  |  | CarP4097: | gdhB |
|  |  | CarP2945: | amn |
|  |  | CarP2863: | DinB superfamily protein |
|  |  | CarP2797: | hypothetical protein |
|  |  | CarP2909: | nboR |
|  |  | CarP1889: | mutS2 |
|  |  | CarP4861: | hypothetical protein |
|  |  | CarP4676: | hypothetical protein |
|  |  | CarP4572: | hypothetical protein |
|  |  | CarP3672: | hypothetical protein |
|  |  | CarP5528: | hypothetical protein |
|  |  | CarP2564: | RNA polymerase sigma factor |
|  |  | CarP1801: | ybeZ |
|  |  | CarP2465: | dxs |
|  |  | CarP5975: | hypothetical protein |
|  |  | CarP6133: | hypothetical protein |
|  |  | CarP4512: | SusD family protein |
|  |  | CarP7158: | hypothetical protein |
|  |  | CarP7131: | Polysaccharide pyruvyl transferase |
|  |  | CarP3913: | iolC |
|  |  | CarP2344: | hypothetical protein |
|  |  | CarP6436: | hypothetical protein |
|  |  | CarP3266: | btr |
|  |  | CarP5413: | hypothetical protein |
|  |  | CarP2768: | Neutral alkaline non-lysosomal ceramidase |
|  |  | CarP4187: | garB |
|  |  | CarP3761: | hypothetical protein |
|  |  | CarP1992: | EamA-like transporter family protein |
|  |  | CarP6925: | CHASE2 domain protein |
|  |  | CarP7311: | hypothetical protein |
|  |  | CarP6685: | hypothetical protein |
|  |  | CarP1806: | recR |
|  |  | CarP7690: | hypothetical protein |
|  |  | CarP7044: | Metal binding domain of Ada |
|  |  | CarP4995: | hypothetical protein |
|  |  | CarP4091: | czcS |
|  |  | CarP5582: | hypothetical protein |
|  |  | CarP6829: | hypothetical protein |
|  |  | CarP6504: | dhaA |
|  |  | CarP6947: | hypothetical protein |
|  |  | CarP4292: | hypothetical protein |
|  |  | CarP5889: | lytR |
|  |  | CarP6511: | uspE |
|  |  | CarP2573: | dsbD |
|  |  | CarP3454: | plsC |
|  |  | CarP6598: | Cyclic nucleotide-binding domain protein |
|  |  | CarP5520: | hypothetical protein |
|  |  | CarP7154: | hypothetical protein |
|  |  | CarP2969: | emrB |
|  |  | CarP6162: | hypothetical protein |
|  |  | CarP4014: | hpaIIM |
|  |  | CarP2680: | hypothetical protein |
|  |  | CarP1994: | putative 3'-5' exonuclease related to the exonuclease domain of PolB |
|  |  | CarP6822: | hypothetical protein |
|  |  | CarP1759: | rlmCD |
|  |  | CarP7218: | hypothetical protein |
|  |  | CarP4924: | trpG |
|  |  | CarP2276: | hypothetical protein |
|  |  | CarP5966: | hypothetical protein |
|  |  | CarP5749: | atsA |
|  |  | CarP2232: | hypothetical protein |
|  |  | CarP5165: | 3-demethylubiquinone-9 3-methyltransferase |
|  |  | CarP5360: | Caspase domain protein |
|  |  | CarP3184: | hypothetical protein |
|  |  | CarP7425: | dan |
|  |  | CarP5615: | Peptidase family S41 |
|  |  | CarP2504: | menE |
|  |  | CarP4854: | arsR |
|  |  | CarP2589: | hsdS |
|  |  | CarP6561: | hypothetical protein |
|  |  | CarP4301: | Methyltransferase domain protein |
|  |  | CarP6855: | wecA |
|  |  | CarP4251: | hypothetical protein |
|  |  | CarP3490: | cysC |
|  |  | CarP7013: | ccp |
|  |  | CarP6008: | hypothetical protein |
|  |  | CarP3763: | hypothetical protein |
|  |  | CarP4939: | Bacterial leucyl aminopeptidase precursor |
|  |  | CarP7244: | baeB |
|  |  | CarP3698: | hypothetical protein |
|  |  | CarP2077: | yfhO |
|  |  | CarP6834: | hypothetical protein |
|  |  | CarP5328: | cysE |
|  |  | CarP6687: | hypothetical protein |
|  |  | CarP1735: | hypothetical protein |
|  |  | CarP2764: | hypothetical protein |
|  |  | CarP4171: | exodeoxyribonuclease VII small subunit |
|  |  | CarP6357: | mepM |
|  |  | CarP1778: | hypothetical protein |
|  |  | CarP7694: | ceramidase |
|  |  | CarP6720: | SusD family protein |
|  |  | CarP1871: | tqsA |
|  |  | CarP5996: | hypothetical protein |
|  |  | CarP2988: | IS66 Orf2 like protein |
|  |  | CarP2033: | Bacterial leucyl aminopeptidase precursor |
|  |  | CarP4524: | Putative addiction module component |
|  |  | CarP5628: | hypothetical protein |
|  |  | CarP3919: | sbcC |
|  |  | CarP7122: | hypothetical protein |
|  |  | CarP3493: | hypothetical protein |
|  |  | CarP1844: | hypothetical protein |
|  |  | CarP3718: | hypothetical protein |
|  |  | CarP6491: | hypothetical protein |
|  |  | CarP4640: | hypothetical protein |
|  |  | CarP6416: | nqrA |
|  |  | CarP4976: | merC |
|  |  | CarP4908: | hypothetical protein |
|  |  | CarP3756: | mrsA |
|  |  | CarP2953: | uxuB |
|  |  | CarP2201: | hypothetical protein |
|  |  | CarP6995: | hypothetical protein |
|  |  | CarP5258: | terB |
|  |  | CarP3049: | Caspase domain protein |
|  |  | CarP3652: | hypothetical protein |
|  |  | CarP6454: | hypothetical protein |
|  |  | CarP5763: | hypothetical protein |
|  |  | CarP6253: | fadB2 |
|  |  | CarP5997: | hypothetical protein |
|  |  | CarP5279: | ruvB |
|  |  | CarP4182: | cusB |
|  |  | CarP6833: | zitB |
|  |  | CarP1645: | glmS |
|  |  | CarP5444: | iolE |
|  |  | CarP4620: | GIY-YIG nuclease superfamily protein |
|  |  | CarP6323: | hypothetical protein |
|  |  | CarP3663: | celZ |
|  |  | CarP2533: | hypothetical protein |
|  |  | CarP4132: | dan |
|  |  | CarP5907: | hypothetical protein |
|  |  | CarP5753: | hypothetical protein |
|  |  | CarP7392: | putative transporter |
|  |  | CarP3077: | Prolyl oligopeptidase family protein |
|  |  | CarP1800: | sdpR |
|  |  | CarP2702: | hypothetical protein |
|  |  | CarP5068: | hypothetical protein |
|  |  | CarP5800: | hypothetical protein |
|  |  | CarP3498: | hypothetical protein |
|  |  | CarP4339: | D-alanyl-D-alanine-carboxypeptidase endope |
|  |  | CarP3522: | nqo1 |
|  |  | CarP6738: | VIT family protein |
|  |  | CarP2021: | Ubiquinone menaquinone biosynthesis C-meth |
|  |  | CarP3396: | trxB |
|  |  | CarP6572: | cph1 |
|  |  | CarP3573: | NmrA-like family protein |
|  |  | CarP5186: | gfo |
|  |  | CarP6874: | hypothetical protein |
|  |  | CarP4001: | galK |
|  |  | CarP4530: | cslA |
|  |  | CarP4111: | FG-GAP repeat protein |
|  |  | CarP2535: | selA |
|  |  | CarP7571: | hypothetical protein |
|  |  | CarP5023: | O-Antigen ligase |
|  |  | CarP5860: | hypothetical protein |
|  |  | CarP1672: | Outer membrane efflux protein |
|  |  | CarP6981: | hypothetical protein |
|  |  | CarP3420: | short chain dehydrogenase |
|  |  | CarP2681: | rfbT |
|  |  | CarP5473: | hypothetical protein |
|  |  | CarP6288: | putative acetyltransferase |
|  |  | CarP2320: | pimB |
|  |  | CarP3434: | ThiS family protein |
|  |  | CarP6463: | Helix-turn-helix domain protein |
|  |  | CarP6014: | hlyD |
|  |  | CarP4063: | hypothetical protein |
|  |  | CarP2899: | can |
|  |  | CarP5980: | hypothetical protein |
|  |  | CarP2346: | dtd |
|  |  | CarP2305: | glpD |
|  |  | CarP2063: | ppiB |
|  |  | CarP5995: | hypothetical protein |
|  |  | CarP3066: | 50S ribosomal protein L21 unknown domain f |
|  |  | CarP4449: | ytrB |
|  |  | CarP4579: | ccpA |
|  |  | CarP3130: | yfiH |
|  |  | CarP6085: | alpha beta hydrolase fold protein |
|  |  | CarP5981: | hypothetical protein |
|  |  | CarP3516: | nuoN |
|  |  | CarP7492: | proA |
|  |  | CarP4152: | GDSL-like Lipase Acylhydrolase |
|  |  | CarP3357: | PGL p-HBAD biosynthesis glycosyltransferas |
|  |  | CarP1039: | hypothetical protein |
|  |  | CarP2222: | hypothetical protein |
|  |  | CarP4162: | hypothetical protein |
|  |  | CarP7444: | hypothetical protein |
|  |  | CarP2915: | rpoE |
|  |  | CarP3691: | SusD family protein |
|  |  | CarP2514: | Metallo-beta-lactamase L1 precursor |
|  |  | CarP6099: | hypothetical protein |
|  |  | CarP5874: | hypothetical protein |
|  |  | CarP3181: | hypothetical protein |
|  |  | CarP3204: | hypothetical protein |
|  |  | CarP4472: | Serine carboxypeptidase |
|  |  | CarP5412: | ptpA |
|  |  | CarP3550: | fdtA |
|  |  | CarP2922: | mecI |
|  |  | CarP2694: | GDSL-like Lipase Acylhydrolase |
|  |  | CarP6498: | htpX |
|  |  | CarP6350: | btuB |
|  |  | CarP6462: | Alpha-L-fucosidase |
|  |  | CarP6342: | phosphodiesterase |
|  |  | CarP3329: | putative epimerase dehydratase |
|  |  | CarP6434: | tetratricopeptide repeat protein |
|  |  | CarP3815: | ktrA |
|  |  | CarP6658: | HicB family protein |
|  |  | CarP6335: | yraA |
|  |  | CarP5824: | hypothetical protein |
|  |  | CarP4699: | hypothetical protein |
|  |  | CarP2453: | dsbH |
|  |  | CarP6410: | hypothetical protein |
|  |  | CarP4400: | Peptidase family S58 |
|  |  | CarP6371: | tcrA |
|  |  | CarP2335: | cysM |
|  |  | CarP5483: | Apolipoprotein A1 A4 E domain protein |
|  |  | CarP4318: | enterobactin ferric enterobactin esterase |
|  |  | CarP6481: | YciI-like protein |
|  |  | CarP6107: | ssuD |
|  |  | CarP6516: | tagO |
|  |  | CarP7430: | hypothetical protein |
|  |  | CarP6732: | hypothetical protein |
|  |  | CarP7543: | pcaK |
|  |  | CarP5265: | fabZ |
|  |  | CarP1892: | hmuU |
|  |  | CarP5745: | hypothetical protein |
|  |  | CarP5056: | Hydrogenase expression formation protein H |
|  |  | CarP4465: | pspA |
|  |  | CarP5158: | SusD family protein |
|  |  | CarP3185: | Four helix bundle sensory module for signa |
|  |  | CarP2927: | qdoI |
|  |  | CarP4317: | sigE |
|  |  | CarP7312: | ssb |
|  |  | CarP6441: | Modification methylase AplI |
|  |  | CarP5679: | hypothetical protein |
|  |  | CarP5552: | tenA |
|  |  | CarP7001: | hypothetical protein |
|  |  | CarP1205: | ybaQ |
|  |  | CarP2234: | yfiC |
|  |  | CarP3260: | axeA1 |
|  |  | CarP4571: | hypothetical protein |
|  |  | CarP2240: | hypothetical protein |
|  |  | CarP5596: | hypothetical protein |
|  |  | CarP3754: | hypothetical protein |
|  |  | CarP5629: | hypothetical protein |
|  |  | CarP4652: | Archaeal ATPase |
|  |  | CarP5919: | hypothetical protein |
|  |  | CarP5268: | fabB |
|  |  | CarP5252: | hypothetical protein |
|  |  | CarP3975: | DinB family protein |
|  |  | CarP1726: | pmbA |
|  |  | CarP7009: | hypothetical protein |
|  |  | CarP7634: | hypothetical protein |
|  |  | CarP6278: | hypothetical protein |
|  |  | CarP7344: | hypothetical protein |
|  |  | CarP6640: | hypothetical protein |
|  |  | CarP1034: | btuB |
|  |  | CarP2115: | ispD |
|  |  | CarP5006: | Na(+) H(+) antiporter subunit C |
|  |  | CarP6427: | hypothetical protein |
|  |  | CarP5022: | fbiB |
|  |  | CarP3682: | hypothetical protein |
|  |  | CarP3506: | hypothetical protein |
|  |  | CarP3519: | nqo9 |
|  |  | CarP3871: | PAP2 superfamily protein |
|  |  | CarP3556: | Polysaccharide biosynthesis export protein |
|  |  | CarP2613: | hypothetical protein |
|  |  | CarP2510: | cheR |
|  |  | CarP2559: | rhlG |
|  |  | CarP2930: | prc |
|  |  | CarP1783: | prs |
|  |  | CarP4949: | sigX |
|  |  | CarP6032: | hypothetical protein |
|  |  | CarP7590: | SusD family protein |
|  |  | CarP5503: | ptlH |
|  |  | CarP2585: | legI |
|  |  | CarP6595: | GIY-YIG nuclease superfamily protein |
|  |  | CarP6507: | hypothetical protein |
|  |  | CarP6106: | hypothetical protein |
|  |  | CarP5879: | hypothetical protein |
|  |  | CarP6309: | hypothetical protein |
|  |  | CarP4622: | hypothetical protein |
|  |  | CarP3904: | mdtC |
|  |  | CarP7251: | nagA |
|  |  | CarP5949: | Integrase core domain protein |
|  |  | CarP4415: | hypothetical protein |
|  |  | CarP6082: | Cupin domain protein |
|  |  | CarP3107: | arnC |
|  |  | CarP7438: | Nucleotidyltransferase domain protein |
|  |  | CarP3925: | oprF |
|  |  | CarP6970: | hypothetical protein |
|  |  | CarP6030: | hypothetical protein |
|  |  | CarP5298: | META domain protein |
|  |  | CarP6635: | hypothetical protein |
|  |  | CarP6385: | ywaD |
|  |  | CarP5748: | hypothetical protein |
|  |  | CarP5081: | bvgS |
|  |  | CarP3064: | HTH-type transcriptional regulator AppY |
|  |  | CarP5028: | rsbU |
|  |  | CarP4003: | egtB |
|  |  | CarP5910: | acpP |
|  |  | CarP3227: | fabG |
|  |  | CarP3466: | bglA |
|  |  | CarP2906: | Acetylornithine acetyl-lysine aminotransfe |
|  |  | CarP7521: | abgB |
|  |  | CarP2686: | lineage-specific thermal regulator protein |
|  |  | CarP3662: | hypothetical protein |
|  |  | CarP7035: | hypothetical protein |
|  |  | CarP3025: | yncA |
|  |  | CarP5173: | Cytochrome c |
|  |  | CarP1799: | yheI |
|  |  | CarP1781: | dapB |
|  |  | CarP2843: | hypothetical protein |
|  |  | CarP6730: | fabZ |
|  |  | CarP5301: | hypothetical protein |
|  |  | CarP6251: | hypothetical protein |
|  |  | CarP2820: | sarZ |
|  |  | CarP7724: | pnbA |
|  |  | CarP2749: | hypothetical protein |
|  |  | CarP2501: | hypothetical protein |
|  |  | CarP7622: | SusD family protein |
|  |  | CarP5947: | bglA |
|  |  | CarP4100: | fieF |
|  |  | CarP1855: | mazG |
|  |  | CarP3924: | sacC |
|  |  | CarP5524: | hldE |
|  |  | CarP3701: | arbA |
|  |  | CarP3080: | Macrolide export ATP-binding permease prot |
|  |  | CarP4238: | Cytochrome c |
|  |  | CarP2161: | lptB |
|  |  | CarP7611: | hypothetical protein |
|  |  | CarP6056: | hypothetical protein |
|  |  | CarP3579: | hypothetical protein |
|  |  | CarP2959: | hypothetical protein |
|  |  | CarP7229: | relG |
|  |  | CarP6620: | Bacterial regulatory proteins, tetR family |
|  |  | CarP5198: | SusD family protein |
|  |  | CarP7538: | hypothetical protein |
|  |  | CarP7529: | hypothetical protein |
|  |  | CarP6069: | ntrC |
|  |  | CarP5403: | ybaQ |
|  |  | CarP3219: | xynZ |
|  |  | CarP7272: | hypothetical protein |
|  |  | CarP7182: | yoaB |
|  |  | CarP4516: | atsA |
|  |  | CarP5174: | hypothetical protein |
|  |  | CarP4393: | Mg-protoporphyrin IX methyl transferase |
|  |  | CarP4007: | hypothetical protein |
|  |  | CarP4081: | hypothetical protein |
|  |  | CarP4371: | hypothetical protein |
|  |  | CarP6686: | ftrA |
|  |  | CarP6509: | bvgS |
|  |  | CarP5898: | ybaQ |
|  |  | CarP1598: | Di- tripeptide transporter |
|  |  | CarP4828: | hypothetical protein |
|  |  | CarP3436: | Macrolide export ATP-binding permease prot |
|  |  | CarP2076: | hypothetical protein |
|  |  | CarP4886: | hypothetical protein |
|  |  | CarP5968: | pal |
|  |  | CarP4998: | cbs |
|  |  | CarP2736: | hypothetical protein |
|  |  | CarP2872: | moaA |
|  |  | CarP4698: | hypothetical protein |
|  |  | CarP1727: | tldD |
|  |  | CarP3058: | pspC |
|  |  | CarP3725: | hypothetical protein |
|  |  | CarP4248: | Phenolphthiocerol synthesis polyketide syn |
|  |  | CarP6490: | macA |
|  |  | CarP6115: | tetA |
|  |  | CarP7707: | mog |
|  |  | CarP2755: | Xylose isomerase-like TIM barrel |
|  |  | CarP3135: | hypothetical protein |
|  |  | CarP4836: | Polysaccharide biosynthesis protein |
|  |  | CarP6555: | hypothetical protein |
|  |  | CarP1604: | hypothetical protein |
|  |  | CarP6963: | hypothetical protein |
|  |  | CarP4731: | hypothetical protein |
|  |  | CarP7016: | NUDIX domain protein |
|  |  | CarP6590: | pntB |
|  |  | CarP2655: | pnuC |
|  |  | CarP5801: | hypothetical protein |
|  |  | CarP3610: | ankX |
|  |  | CarP3272: | kynU |
|  |  | CarP1525: | xerD |
|  |  | CarP6998: | ydbC |
|  |  | CarP2795: | copper silver efflux system outer membrane |
|  |  | CarP4242: | OsmC-like protein |
|  |  | CarP7049: | hypothetical protein |
|  |  | CarP4638: | Lactonase, 7-bladed beta-propeller |
|  |  | CarP1938: | FG-GAP repeat protein |
|  |  | CarP5110: | glxR |
|  |  | CarP7680: | Nuclease A inhibitor-like protein |
|  |  | CarP1771: | aspS |
|  |  | CarP5875: | epsH |
|  |  | CarP6861: | hypothetical protein |
|  |  | CarP4927: | TonB-dependent Receptor Plug Domain protei |
|  |  | CarP4070: | ccpA |
|  |  | CarP2459: | NAD NADP-dependent betaine aldehyde dehydr |
|  |  | CarP7384: | hutI |
|  |  | CarP6161: | zinc cadmium mercury lead-transporting ATP |
|  |  | CarP2839: | hypothetical protein |
|  |  | CarP6079: | pbpE |
|  |  | CarP4274: | Undecaprenyl-phosphate mannosyltransferase |
|  |  | CarP5018: | hypothetical protein |
|  |  | CarP3248: | hypothetical protein |
|  |  | CarP5849: | mtfA |
|  |  | CarP7523: | gatA |
|  |  | CarP4300: | hypothetical protein |
|  |  | CarP4195: | ycjS |
|  |  | CarP4646: | hypothetical protein |
|  |  | CarP3347: | hypothetical protein |
|  |  | CarP1539: | ftrA |
|  |  | CarP7232: | aminoalkylphosphonic acid N-acetyltransferase |
|  |  | CarP4350: | hypothetical protein |
|  |  | CarP3759: | hypothetical protein |
|  |  | CarP5092: | oprM |
|  |  | CarP3646: | hypothetical protein |
|  |  | CarP2342: | bamA |
|  |  | CarP2611: | hypothetical protein |
|  |  | CarP1870: | hypothetical protein |
|  |  | CarP6503: | hypothetical protein |
|  |  | CarP4812: | Haemolysin-III related |
|  |  | CarP4883: | opuD |
|  |  | CarP3091: | FG-GAP repeat protein |
|  |  | CarP5278: | hypothetical protein |
|  |  | CarP4988: | Zinc-type alcohol dehydrogenase-like prote |
|  |  | CarP5422: | lipopolysaccharide core biosynthesis prote |
|  |  | CarP5921: | hypothetical protein |
|  |  | CarP3874: | hypothetical protein |
|  |  | CarP3901: | hypothetical protein |
|  |  | CarP3609: | hypothetical protein |
|  |  | CarP4887: | qdoI |
|  |  | CarP1827: | folD |
|  |  | CarP7041: | SusD family protein |
|  |  | CarP7296: | ccp |
|  |  | CarP3308: | cirA |
|  |  | CarP3351: | hypothetical protein |
|  |  | CarP1112: | YceI-like domain protein |
|  |  | CarP6274: | mepA |
|  |  | CarP6301: | gfo |
|  |  | CarP6596: | D12 class N6 adenine-specific DNA methyltr |
|  |  | CarP7612: | Heme NO binding protein |
|  |  | CarP4113: | idhA |
|  |  | CarP7647: | hypothetical protein |
|  |  | CarP7503: | hypothetical protein |
|  |  | CarP6667: | pdp |
|  |  | CarP6004: | hypothetical protein |
|  |  | CarP6989: | icaA |
|  |  | CarP2386: | pyrC |
|  |  | CarP2066: | hypothetical protein |
|  |  | CarP5029: | hsdM |
|  |  | CarP2165: | rpiB |
|  |  | CarP6456: | hypothetical protein |
|  |  | CarP4293: | PGL p-HBAD biosynthesis glycosyltransferas |
|  |  | CarP5924: | degU |
|  |  | CarP2477: | metX |
|  |  | CarP6057: | hypothetical protein |
|  |  | CarP3738: | Starch-binding associating with outer memb |
|  |  | CarP3528: | hldD |
|  |  | CarP3409: | tolB |
|  |  | CarP1596: | yhhQ |
|  |  | CarP3481: | Eukaryotic DNA topoisomerase I, catalytic |
|  |  | CarP6246: | bphF |
|  |  | CarP4791: | yueD |
|  |  | CarP5637: | scpA |
|  |  | CarP2949: | spmA |
|  |  | CarP6075: | LytTr DNA-binding domain protein |
|  |  | CarP5238: | hypothetical protein |
|  |  | CarP7409: | udk |
|  |  | CarP5242: | bioC |
|  |  | CarP5851: | SET domain protein |
|  |  | CarP2233: | ompH |
|  |  | CarP3048: | hypothetical protein |
|  |  | CarP5993: | hypothetical protein |
|  |  | CarP2727: | Cyclic nucleotide-gated potassium channel |
|  |  | CarP2198: | ribD |
|  |  | CarP3997: | Peptidase M16 inactive domain protein |
|  |  | CarP4312: | PAP2 superfamily protein |
|  |  | CarP3607: | hsdR |
|  |  | CarP4671: | btr |
|  |  | CarP6259: | hypothetical protein |
|  |  | CarP3020: | phoA |
|  |  | CarP1747: | ptpA |
|  |  | CarP5742: | hypothetical protein |
|  |  | CarP3105: | aceF |
|  |  | CarP4820: | hypothetical protein |
|  |  | CarP3812: | hypothetical protein |
|  |  | CarP7601: | Blue-light-activated histidine kinase 2 |
|  |  | CarP3899: | iolI |
|  |  | CarP2791: | clcD |
|  |  | CarP4135: | ccpA |
|  |  | CarP6484: | 2-acyl-glycerophospho-ethanolamine acyltra |
|  |  | CarP2279: | ctaB |
|  |  | CarP4105: | LigB family dioxygenase |
|  |  | CarP6840: | Acyltransferase family protein |
|  |  | CarP5655: | cirA |
|  |  | CarP2612: | treA |
|  |  | CarP4042: | yhdN |
|  |  | CarP3830: | hypothetical protein |
|  |  | CarP7262: | hypothetical protein |
|  |  | CarP5175: | hypothetical protein |
|  |  | CarP4575: | Calcineurin-like phosphoesterase |
|  |  | CarP3113: | csp |
|  |  | CarP7406: | hypothetical protein |
|  |  | CarP5113: | hypothetical protein |
|  |  | CarP4178: | Serine threonine exchanger SteT |
|  |  | CarP6104: | hypothetical protein |
|  |  | CarP7143: | hypothetical protein |
|  |  | CarP2348: | Putative anti-sigma factor antagonist |
|  |  | CarP2331: | pyrH |
|  |  | CarP6838: | hypothetical protein |
|  |  | CarP5933: | hypothetical protein |
|  |  | CarP4878: | tehB |
|  |  | CarP3775: | Enamine imine deaminase |
|  |  | CarP6526: | fabG |
|  |  | CarP4062: | hypothetical protein |
|  |  | CarP3255: | hypothetical protein |
|  |  | CarP7081: | kfoC |
|  |  | CarP6676: | YHS domain protein |
|  |  | CarP4066: | arnC |
|  |  | CarP6312: | yoaD |
|  |  | CarP2401: | Pyridoxine pyridoxamine 5'-phosphate oxida |
|  |  | CarP5911: | hypothetical protein |
|  |  | CarP4997: | hypothetical protein |
|  |  | CarP7609: | ypdA |
|  |  | CarP3996: | hypothetical protein |
|  |  | CarP3902: | SPFH domain Band 7 family protein |
|  |  | CarP6810: | cph1 |
|  |  | CarP2608: | ywrD |
|  |  | CarP4272: | vancomycin high temperature exclusion prot |
|  |  | CarP4619: | senX3 |
|  |  | CarP2347: | hemB |
|  |  | CarP5955: | putative transposase |
|  |  | CarP5487: | pabB |
|  |  | CarP5458: | hypothetical protein |
|  |  | CarP5052: | hypothetical protein |
|  |  | CarP6114: | hypothetical protein |
|  |  | CarP5105: | kdgK |
|  |  | CarP6996: | hypothetical protein |
|  |  | CarP3937: | hypothetical protein |
|  |  | CarP6901: | putative nicotinate phosphoribosyltransfer |
|  |  | CarP6633: | CAAX amino terminal protease self- immunity |
|  |  | CarP6706: | T4-like virus tail tube protein gp19 |
|  |  | CarP3313: | Putative acetyltransferase |
|  |  | CarP2111: | Putative bifunctional phosphatase peptidyl |
|  |  | CarP6785: | hypothetical protein |
|  |  | CarP5806: | xanthine-guanine phosphoribosyltransferase |
|  |  | CarP4639: | hypothetical protein |
|  |  | CarP4593: | hypothetical protein |
|  |  | CarP4286: | hypothetical protein |
|  |  | CarP3355: | Outer membrane efflux protein |
|  |  | CarP5931: | hypothetical protein |
|  |  | CarP4287: | hypothetical protein |
|  |  | CarP3864: | mecI |
|  |  | CarP2403: | hypothetical protein |
|  |  | CarP2911: | axe7A |
|  |  | CarP4573: | hypothetical protein |
|  |  | CarP2762: | Fatty acid hydroxylase superfamily protein |
|  |  | CarP1736: | mscS |
|  |  | CarP4724: | hypothetical protein |
|  |  | CarP3330: | KWG Leptospira |
|  |  | CarP3126: | pckA |
|  |  | CarP7197: | hypothetical protein |
|  |  | CarP4595: | htrB |
|  |  | CarP4467: | hypothetical protein |
|  |  | CarP5166: | Thermophilic serine proteinase precursor |
|  |  | CarP2954: | YCII-related domain protein |
|  |  | CarP7534: | Alpha beta hydrolase family protein |
|  |  | CarP5041: | hypothetical protein |
|  |  | CarP4778: | lieA |
|  |  | CarP2371: | 6-pyruvoyl tetrahydropterin synthase |
|  |  | CarP2310: | ccmF |
|  |  | CarP1984: | linC |
|  |  | CarP6254: | hemL |
|  |  | CarP5024: | hypothetical protein |
|  |  | CarP5309: | hypothetical protein |
|  |  | CarP7531: | Ureidoglycolate lyase |
|  |  | CarP7613: | Xylose isomerase-like TIM barrel |
|  |  | CarP6655: | hypothetical protein |
|  |  | CarP7441: | hypothetical protein |
|  |  | CarP2511: | hypothetical protein |
|  |  | CarP6252: | hypothetical protein |
|  |  | CarP5272: | fabZ |
|  |  | CarP3009: | hypothetical protein |
|  |  | CarP6801: | hypothetical protein |
|  |  | CarP2941: | hypothetical protein |
|  |  | CarP7227: | oxidative stress defense protein |
|  |  | CarP1785: | fbp |
|  |  | CarP4412: | hypothetical protein |
|  |  | CarP4742: | FG-GAP repeat protein |
|  |  | CarP4227: | hypothetical protein |
|  |  | CarP4208: | dat |
|  |  | CarP2458: | CO2+ MG2+ efflux protein ApaG |
|  |  | CarP1371: | hypothetical protein |
|  |  | CarP4442: | hypothetical protein |
|  |  | CarP4148: | hypothetical protein |
|  |  | CarP2102: | hypothetical protein |
|  |  | CarP6165: | hypothetical protein |
|  |  | CarP5449: | hypothetical protein |
|  |  | CarP2763: | hypothetical protein |
|  |  | CarP4016: | nuc |
|  |  | CarP6126: | dnaK |
|  |  | CarP7213: | hypothetical protein |
|  |  | CarP7297: | hypothetical protein |
|  |  | CarP3086: | iscS |
|  |  | CarP1724: | mdtC |
|  |  | CarP7111: | rebG |
|  |  | CarP3790: | DNA alkylation repair enzyme |
|  |  | CarP6537: | hypothetical protein |
|  |  | CarP3281: | bifunctional 3-demethylubiquinone-9 3-methyltransferase/ 2-octaprenyl-6-hydroxy phenol methylase |
|  |  | CarP5621: | hypothetical protein |
|  |  | CarP5855: | hypothetical protein |
|  |  | CarP5415: | cycA |
|  |  | CarP5141: | perR |
|  |  | CarP5789: | atsA |
|  |  | CarP4674: | btuB |
|  |  | CarP4803: | hypothetical protein |
|  |  | CarP2213: | hemH |
|  |  | CarP5182: | hypothetical protein |
|  |  | CarP4546: | Helix-turn-helix domain protein |
|  |  | CarP4018: | azoB |
|  |  | CarP7378: | hypothetical protein |
|  |  | CarP4196: | ccpA |
|  |  | CarP3862: | SusD family protein |
|  |  | CarP4214: | ugl |
|  |  | CarP6488: | hypothetical protein |
|  |  | CarP5554: | hypothetical protein |
|  |  | CarP3660: | Regulator of chromosome condensation (RCC1 |
|  |  | CarP6314: | hypothetical protein |
|  |  | CarP4888: | hypothetical protein |
|  |  | CarP6070: | hypothetical protein |
|  |  | CarP6271: | Acyltransferase |
|  |  | CarP4720: | aseR |
|  |  | CarP7587: | hypothetical protein |
|  |  | CarP5037: | putative cation efflux system protein MT20 |
|  |  | CarP4717: | Undecaprenyl-phosphate mannosyltransferase |
|  |  | CarP5560: | hypothetical protein |
|  |  | CarP7204: | Putative Ig domain protein |
|  |  | CarP5606: | phosphoribosylformylglycinamidine synthase II |
|  |  | CarP2097: | Proteasome subunit |
|  |  | CarP4247: | oatA |
|  |  | CarP7273: | hypothetical protein |
|  |  | CarP3510: | pbpF |
|  |  | CarP3737: | coxS |
|  |  | CarP3880: | ywnH |
|  |  | CarP1681: | hypothetical protein |
|  |  | CarP5132: | kipI |
|  |  | CarP5976: | hypothetical protein |
|  |  | CarP6599: | tlyC |
|  |  | CarP7301: | cpdA |
|  |  | CarP6483: | atsA |
|  |  | CarP3742: | hypothetical protein |
|  |  | CarP5269: | fabB |
|  |  | CarP5318: | PKD domain protein |
|  |  | CarP5095: | fabB |
|  |  | CarP2064: | etfA |
|  |  | CarP6322: | FG-GAP repeat protein |
|  |  | CarP2913: | bepF |
|  |  | CarP2580: | bm3R1 |
|  |  | CarP7715: | hypothetical protein |
|  |  | CarP7361: | Phosphoesterase family protein |
|  |  | CarP3365: | glxK |
|  |  | CarP7245: | hypothetical protein |
|  |  | CarP3442: | aprE |
|  |  | CarP2742: | ypdA |
|  |  | CarP5785: | lysA |
|  |  | CarP3241: | TatD related DNase |
|  |  | CarP4270: | hypothetical protein |
|  |  | CarP5737: | blaI |
|  |  | CarP4184: | lytR |
|  |  | CarP3439: | nylB |
|  |  | CarP1728: | Helix-turn-helix |
|  |  | CarP1993: | hypothetical protein |
|  |  | CarP3728: | hypothetical protein |
|  |  | CarP3303: | hypothetical protein |
|  |  | CarP6969: | putative DNA-binding transcriptional regul |
|  |  | CarP6846: | cysE |
|  |  | CarP6828: | hypothetical protein |
|  |  | CarP6707: | hypothetical protein |
|  |  | CarP7615: | LytTr DNA-binding domain protein |
|  |  | CarP5927: | hypothetical protein |
|  |  | CarP1819: | ywaD |
|  |  | CarP3259: | araC |
|  |  | CarP3574: | rhaR |
|  |  | CarP5871: | hypothetical protein |
|  |  | CarP6975: | hypothetical protein |
|  |  | CarP5974: | ampH |
|  |  | CarP5093: | dauA |
|  |  | CarP4985: | atsA |
|  |  | CarP4311: | hypothetical protein |
|  |  | CarP5156: | SusD family protein |
|  |  | CarP4686: | putative oxidoreductase |
|  |  | CarP6285: | Glyoxalase-like domain protein |
|  |  | CarP5823: | hypothetical protein |
|  |  | CarP4801: | hypothetical protein |
|  |  | CarP6914: | pseG |
|  |  | CarP4916: | putative acyl-CoA thioester hydrolase |
|  |  | CarP3388: | dbpA |
|  |  | CarP5938: | hypothetical protein |
|  |  | CarP5397: | tmoS |
|  |  | CarP5664: | rluA |
|  |  | CarP5573: | hypothetical protein |
|  |  | CarP2759: | hypothetical protein |
|  |  | CarP7756: | hypothetical protein |
|  |  | CarP6512: | hypothetical protein |
|  |  | CarP4667: | fabG |
|  |  | CarP7271: | Late embryogenesis abundant protein |
|  |  | CarP6343: | hypothetical protein |
|  |  | CarP5535: | hypothetical protein |
|  |  | CarP4736: | Na(+) H(+) antiporter subunit A |
|  |  | CarP3521: | nqo3 |
|  |  | CarP7048: | polC |
|  |  | CarP6659: | hypothetical protein |
|  |  | CarP6496: | hypothetical protein |
|  |  | CarP4058: | betC |
|  |  | CarP5636: | pcaF |
|  |  | CarP3696: | sigL |
|  |  | CarP5069: | deoxyhypusine synthase-like protein |
|  |  | CarP2074: | yfiT |
|  |  | CarP5021: | Nitroreductase family protein |
|  |  | CarP7126: | WD40-like Beta Propeller Repeat protein |
|  |  | CarP4487: | hypothetical protein |
|  |  | CarP7236: | ssb |
|  |  | CarP7246: | dhfrIII |
|  |  | CarP5803: | 2,6-dihydropseudooxynicotine hydrolase |
|  |  | CarP7171: | Trans-acting enoyl reductase |
|  |  | CarP4121: | fdhF |
|  |  | CarP2190: | hypothetical protein |
|  |  | CarP6788: | putative acyl-CoA thioester hydrolase |
|  |  | CarP3544: | cmdF |
|  |  | CarP1524: | hypothetical protein |
|  |  | CarP4257: | gdh |
|  |  | CarP5797: | Relaxase Mobilisation nuclease domain prot |
|  |  | CarP7591: | acrC |
|  |  | CarP7351: | cpt |
|  |  | CarP6486: | ppgK |
|  |  | CarP6446: | hypothetical protein |
|  |  | CarP6457: | hypothetical protein |
|  |  | CarP1837: | hypothetical protein |
|  |  | CarP3956: | rluA |
|  |  | CarP5234: | hypothetical protein |
|  |  | CarP2245: | hypothetical protein |
|  |  | CarP7689: | hypothetical protein |
|  |  | CarP6880: | yqiK |
|  |  | CarP1662: | rnhB |
|  |  | CarP1891: | Epimerase family protein |
|  |  | CarP5513: | ydhF |
|  |  | CarP6574: | atsA |
|  |  | CarP5300: | hsdR |
|  |  | CarP7443: | Hydroxymethylglutaryl-coenzyme A reductase |
|  |  | CarP5479: | rbsB |
|  |  | CarP1914: | yliI |
|  |  | CarP3677: | xerD |
|  |  | CarP2013: | asnB |
|  |  | CarP6467: | mca |
|  |  | CarP7436: | cfiB |
|  |  | CarP6331: | alr |
|  |  | CarP2274: | hypothetical protein |
|  |  | CarP4084: | hypothetical protein |
|  |  | CarP4458: | Macrolide export ATP-binding permease prot |
|  |  | CarP3051: | hypothetical protein |
|  |  | CarP3551: | hypothetical protein |
|  |  | CarP6417: | nqrA |
|  |  | CarP1533: | hypothetical protein |
|  |  | CarP5248: | hypothetical protein |
|  |  | CarP7031: | hypothetical protein |
|  |  | CarP3180: | hypothetical protein |
|  |  | CarP2999: | hypothetical protein |
|  |  | CarP2769: | hypothetical protein |
|  |  | CarP2323: | hemA |
|  |  | CarP6066: | hypothetical protein |
|  |  | CarP3785: | pncB2 |
|  |  | CarP7421: | hypothetical protein |
|  |  | CarP4535: | cph1 |
|  |  | CarP4835: | hypothetical protein |
|  |  | CarP4879: | TonB-dependent Receptor Plug Domain protei |
|  |  | CarP4032: | leuA |
|  |  | CarP3477: | bifunctional 3-demethylubiquinone-9 3-methyltransferase/ 2-octaprenyl-6-hydroxy phenol methylase |
|  |  | CarP1802: | Putative O-methyltransferase MSMEI 4947 |
|  |  | CarP2241: | ydgJ |
|  |  | CarP4372: | novN |
|  |  | CarP5240: | aminoalkylphosphonic acid N-acetyltransferase |
|  |  | CarP6736: | iolI |
|  |  | CarP1858: | aspC |
|  |  | CarP1591: | hypothetical protein |
|  |  | CarP4906: | nbaC |
|  |  | CarP1114: | hypothetical protein |
|  |  | CarP2767: | hypothetical protein |
|  |  | CarP5920: | hypothetical protein |
|  |  | CarP1599: | WbqC-like protein family protein |
|  |  | CarP5107: | hypothetical protein |
|  |  | CarP3452: | malP |
|  |  | CarP2345: | hypothetical protein |
|  |  | CarP7147: | Calcineurin-like phosphoesterase |
|  |  | CarP6353: | Fatty acid desaturase |
|  |  | CarP4077: | scpC |
|  |  | CarP7540: | pcaB |
|  |  | CarP3079: | treZ |
|  |  | CarP5015: | cocE |
|  |  | CarP1851: | def |
|  |  | CarP5347: | Epi-isozizaene 5-monooxygenase (E)-beta-fa |
|  |  | CarP7431: | treZ |
|  |  | CarP4898: | ybeZ |
|  |  | CarP2961: | hypothetical protein |
|  |  | CarP6751: | hypothetical protein |
|  |  | CarP6778: | Alpha beta hydrolase family protein |
|  |  | CarP6835: | hypothetical protein |
|  |  | CarP3124: | mlaE |
|  |  | CarP7189: | pglJ |
|  |  | CarP4632: | Glutaryl-7-aminocephalosporanic-acid acyla |
|  |  | CarP7334: | iolB |
|  |  | CarP5179: | pglA |
|  |  | CarP3395: | mepA |
|  |  | CarP7708: | nlpI |
|  |  | CarP6929: | hypothetical protein |
|  |  | CarP1887: | deoC1 |
|  |  | CarP5074: | SusD family protein |
|  |  | CarP4562: | glgX |
|  |  | CarP6986: | hypothetical protein |
|  |  | CarP7059: | ypdA |
|  |  | CarP3455: | can |
|  |  | CarP2856: | mapB |
|  |  | CarP6002: | hypothetical protein |
|  |  | CarP4355: | GtrA-like protein |
|  |  | CarP4288: | yehU |
|  |  | CarP2001: | hypothetical protein |
|  |  | CarP6812: | Serine threonine-protein kinase RsbT |
|  |  | CarP4197: | ptpA |
|  |  | CarP7104: | linC |
|  |  | CarP5277: | hypothetical protein |
|  |  | CarP6733: | Helix-turn-helix |
|  |  | CarP7322: | gmhB |
|  |  | CarP6384: | tlpA |
|  |  | CarP5972: | hypothetical protein |
|  |  | CarP6997: | gno |
|  |  | CarP4085: | Blue-light-activated histidine kinase 1 |
|  |  | CarP6680: | hypothetical protein |
|  |  | CarP6341: | putative TonB-dependent receptor precursor |
|  |  | CarP3375: | luxA |
|  |  | CarP7402: | resA |
|  |  | CarP3984: | phnX |
|  |  | CarP2780: | Dihydrolipoyl dehydrogenase |
|  |  | CarP7446: | hypothetical protein |
|  |  | CarP3256: | yvdD |
|  |  | CarP7136: | hypothetical protein |
|  |  | CarP6038: | Glycosyl hydrolases family 39 |
|  |  | CarP5848: | celI |
|  |  | CarP1667: | hypothetical protein |
|  |  | CarP6510: | cat1 |
|  |  | CarP1942: | hypothetical protein |
|  |  | CarP7565: | hypothetical protein |
|  |  | CarP7194: | mshB |
|  |  | CarP6767: | KWG Leptospira |
|  |  | CarP2144: | putative reductase |
|  |  | CarP3520: | nqo8 |
|  |  | CarP7582: | hypothetical protein |
|  |  | CarP1062: | Transposase IS66 family protein |
|  |  | CarP6155: | Transposase IS200 like protein |
|  |  | CarP2541: | PKD domain protein |
|  |  | CarP1535: | Polysaccharide biosynthesis export protein |
|  |  | CarP2845: | hypothetical protein |
|  |  | CarP5688: | hypothetical protein |
|  |  | CarP3085: | hypothetical protein |
|  |  | CarP6936: | hypothetical protein |
|  |  | CarP3794: | Undecaprenyl-phosphate mannosyltransferase |
|  |  | CarP3039: | Fibronectin type III domain protein |
|  |  | CarP5746: | FG-GAP repeat protein |
|  |  | CarP2051: | coaE |
|  |  | CarP6132: | hypothetical protein |
|  |  | CarP6037: | hypothetical protein |
|  |  | CarP3157: | hypothetical protein |
|  |  | CarP2770: | HNH endonuclease |
|  |  | CarP3244: | mecI |
|  |  | CarP7474: | aacA4 |
|  |  | CarP2701: | upp |
|  |  | CarP4455: | hypothetical protein |
|  |  | CarP3056: | hypothetical protein |
|  |  | CarP2897: | bfce |
|  |  | CarP5244: | nlhH |
|  |  | CarP4445: | hypothetical protein |
|  |  | CarP5507: | hypothetical protein |
|  |  | CarP2659: | macA |
|  |  | CarP4462: | hypothetical protein |
|  |  | CarP4641: | hypothetical protein |
|  |  | CarP5755: | hypothetical protein |
|  |  | CarP6902: | Helix-turn-helix |
|  |  | CarP4072: | fecR |
|  |  | CarP2817: | ltaA |
|  |  | CarP2505: | menB |
|  |  | CarP1696: | hypothetical protein |
|  |  | CarP2660: | Macrolide export ATP-binding permease protein |
|  |  | CarP7413: | dcsG |
|  |  | CarP5257: | qseF |
|  |  | CarP2444: | aguA |
|  |  | CarP4071: | sigX |
|  |  | CarP5548: | noeI |
|  |  | CarP5326: | iorA |
|  |  | CarP2634: | NADH:ubiquinone oxidoreductase subunit J |
|  |  | CarP2227: | hypothetical protein |
|  |  | CarP2433: | ompH |
|  |  | CarP4668: | XisH protein |
|  |  | CarP7677: | hypothetical protein |
|  |  | CarP6551: | Glutaryl-7-aminocephalosporanic-acid acyla |
|  |  | CarP2647: | nuoL |
|  |  | CarP7356: | M61 glycyl aminopeptidase |
|  |  | CarP2248: | cmk |
|  |  | CarP3063: | hypothetical protein |
|  |  | CarP7097: | arnE |
|  |  | CarP5271: | ybgC |
|  |  | CarP5952: | Recombinase |
|  |  | CarP4061: | Radical SAM superfamily protein |
|  |  | CarP2493: | queA |
|  |  | CarP7630: | hypothetical protein |
|  |  | CarP7176: | hypothetical protein |
|  |  | CarP4281: | hypothetical protein |
|  |  | CarP7357: | hypothetical protein |
|  |  | CarP6763: | hypothetical protein |
|  |  | CarP6882: | Chromatin associated protein KTI12 |
|  |  | CarP6769: | fabB |
|  |  | CarP5251: | hypothetical protein |
|  |  | CarP3353: | cefD |
|  |  | CarP4613: | nanM |
|  |  | CarP7396: | hypothetical protein |
|  |  | CarP7720: | oxidoreductase |
|  |  | CarP4979: | hypothetical protein |
|  |  | CarP4848: | degP1 |
|  |  | CarP4795: | araD |
|  |  | CarP5281: | L-Ala-D L-Glu epimerase |
|  |  | CarP7248: | hypothetical protein |
|  |  | CarP1050: | hcf136 |
|  |  | CarP2117: | frdB |
|  |  | CarP5557: | hypothetical protein |
|  |  | CarP1638: | mltD |
|  |  | CarP5336: | hypothetical protein |
|  |  | CarP6956: | hypothetical protein |
|  |  | CarP4256: | hypothetical protein |
|  |  | CarP6482: | atsA |
|  |  | CarP6313: | hypothetical protein |
|  |  | CarP4384: | hypothetical protein |
|  |  | CarP4794: | Siderophore-interacting protein |
|  |  | CarP4612: | hypothetical protein |
|  |  | CarP7160: | hypothetical protein |
|  |  | CarP6673: | glnB |
|  |  | CarP2427: | pepN |
|  |  | CarP7355: | mdoC |
|  |  | CarP7157: | oprF |
|  |  | CarP2047: | Sulfate thiosulfate import ATP-binding pro |
|  |  | CarP3089: | fbp |
|  |  | CarP2175: | ribE |
|  |  | CarP7298: | hypothetical protein |
|  |  | CarP5163: | yvaA |
|  |  | CarP3859: | nirM |
|  |  | CarP7717: | hypothetical protein |
|  |  | CarP7186: | hypothetical protein |
|  |  | CarP5267: | pgdA |
|  |  | CarP7088: | yvoA |
|  |  | CarP3019: | hepA |
|  |  | CarP2512: | sigX |
|  |  | CarP2673: | chrA |
|  |  | CarP7548: | hypothetical protein |
|  |  | CarP4655: | saeR |
|  |  | CarP3030: | ttgF |
|  |  | CarP6814: | rsbRA |
|  |  | CarP6752: | Competence protein CoiA-like family protei |
|  |  | CarP5652: | hypothetical protein |
|  |  | CarP4942: | hypothetical protein |
|  |  | CarP7055: | lspA |
|  |  | CarP1709: | walK |
|  |  | CarP1970: | sigK |
|  |  | CarP3995: | qorA |
|  |  | CarP5923: | hypothetical protein |
|  |  | CarP2337: | aroB |
|  |  | CarP2226: | fadA |
|  |  | CarP5564: | arnC |
|  |  | CarP2829: | feoB |
|  |  | CarP2881: | Glyoxylate hydroxypyruvate reductase A |
|  |  | CarP7451: | hypothetical protein |
|  |  | CarP6418: | nqrA |
|  |  | CarP3727: | liaR |
|  |  | CarP2078: | Polysaccharide biosynthesis protein |
|  |  | CarP5581: | hypothetical protein |
|  |  | CarP4079: | gdhA |
|  |  | CarP5970: | hypothetical protein |
|  |  | CarP3577: | ptpA |
|  |  | CarP5313: | hypothetical protein |
|  |  | CarP3641: | Polymer-forming cytoskeletal |
|  |  | CarP2003: | hppA1 |
|  |  | CarP4654: | mprA |
|  |  | CarP6951: | DNA-dependent helicase II |
|  |  | CarP7572: | ypdB |
|  |  | CarP5627: | phosphoribosylformylglycinamidine synthase II |
|  |  | CarP5589: | iolE |
|  |  | CarP4206: | gfo |
|  |  | CarP3372: | yjjN |
|  |  | CarP6275: | acn |
|  |  | CarP1901: | Pentapeptide repeats (8 copies) |
|  |  | CarP1088: | fecR |
|  |  | CarP2658: | macA |
|  |  | CarP5250: | rebM |
|  |  | CarP2151: | fumC |
|  |  | CarP4900: | hypothetical protein |
|  |  | CarP4733: | AP-4-A phosphorylase |
|  |  | CarP3508: | RNA polymerase sigma factor |
|  |  | CarP4999: | SusD family protein |
|  |  | CarP7508: | btuB |
|  |  | CarP6957: | hypothetical protein |
|  |  | CarP4299: | albA |
|  |  | CarP2073: | pgi |
|  |  | CarP5160: | Trehalose utilisation |
|  |  | CarP3161: | putative S-adenosylmethionine-dependent me |
|  |  | CarP5445: | arfA |
|  |  | CarP3360: | epsM |
|  |  | CarP3301: | axeA1 |
|  |  | CarP2275: | Cytochrome c |
|  |  | CarP6553: | putative oxidoreductase |
|  |  | CarP5317: | LysE type translocator |
|  |  | CarP4267: | kinB |
|  |  | CarP1041: | Integrase core domain protein |
|  |  | CarP5485: | Glycosyl hydrolases family 43 |
|  |  | CarP2605: | cph1 |
|  |  | CarP3307: | ybaQ |
|  |  | CarP2920: | hypothetical protein |
|  |  | CarP2782: | ald |
|  |  | CarP3638: | hypothetical protein |
|  |  | CarP6305: | MORN repeat variant |
|  |  | CarP4957: | hypothetical protein |
|  |  | CarP3940: | Major Facilitator Superfamily protein |
|  |  | CarP7455: | hypothetical protein |
|  |  | CarP6501: | hypothetical protein |
|  |  | CarP3804: | hypothetical protein |
|  |  | CarP3772: | gatA |
|  |  | CarP7747: | NIF3 (NGG1p interacting factor 3) |
|  |  | CarP6755: | hypothetical protein |
|  |  | CarP6700: | hemY |
|  |  | CarP6333: | tetratricopeptide repeat protein |
|  |  | CarP2931: | accD5 |
|  |  | CarP5438: | hypothetical protein |
|  |  | CarP7343: | hypothetical protein |
|  |  | CarP2421: | Peptidase family M50 |
|  |  | CarP2389: | wbpA |
|  |  | CarP6980: | LVIVD repeat protein |
|  |  | CarP4869: | xylI |
|  |  | CarP5417: | yiaD |
|  |  | CarP5453: | hypothetical protein |
|  |  | CarP2536: | GIY-YIG nuclease superfamily protein |
|  |  | CarP2373: | purA |
|  |  | CarP3399: | hypothetical protein |
|  |  | CarP4116: | UDP-D-galactose:(glucosyl)lipopolysacchari |
|  |  | CarP2478: | hypothetical protein |
|  |  | CarP2135: | kdsC |
|  |  | CarP1107: | Putative tyrosine-protein kinase in cps re |
|  |  | CarP6134: | hypothetical protein |
|  |  | CarP2924: | resA |
|  |  | CarP3733: | hypothetical protein |
|  |  | CarP3027: | NmrA-like family protein |
|  |  | CarP2498: | Cytochrome oxidase maturation protein cbb3 |
|  |  | CarP5816: | atsA |
|  |  | CarP4739: | topology modulation protein |
|  |  | CarP1895: | aroF |
|  |  | CarP5888: | ypdA |
|  |  | CarP1507: | cysG |
|  |  | CarP7736: | mcrB |
|  |  | CarP3664: | celD |
|  |  | CarP2200: | pbuE |
|  |  | CarP5411: | hypothetical protein |
|  |  | CarP2081: | hypothetical protein |
|  |  | CarP4450: | Vitamin K epoxide reductase family protein |
|  |  | CarP3770: | treY |
|  |  | CarP1677: | mfpsA |
|  |  | CarP6091: | hypothetical protein |
|  |  | CarP2181: | hypothetical protein |
|  |  | CarP7318: | hypothetical protein |
|  |  | CarP4918: | hypothetical protein |
|  |  | CarP4544: | hypothetical protein |
|  |  | CarP2846: | prc |
|  |  | CarP1717: | glgB |
|  |  | CarP2726: | hypothetical protein |
|  |  | CarP3957: | hypothetical protein |
|  |  | CarP6864: | NmrA-like family protein |
|  |  | CarP6648: | hypothetical protein |
|  |  | CarP7287: | hypothetical protein |
|  |  | CarP1834: | putative CtpA-like serine protease |
|  |  | CarP2358: | gcvT |
|  |  | CarP7209: | hypothetical protein |
|  |  | CarP4715: | anhydro-N-acetylmuramic acid kinase |
|  |  | CarP7716: | hypothetical protein |
|  |  | CarP5237: | Cyclic nucleotide-binding domain protein |
|  |  | CarP7407: | hypothetical protein |
|  |  | CarP5082: | GDSL-like Lipase Acylhydrolase |
|  |  | CarP5076: | hypothetical protein |
|  |  | CarP5736: | hypothetical protein |
|  |  | CarP5854: | hypothetical protein |
|  |  | CarP6867: | tetratricopeptide repeat protein |
|  |  | CarP6568: | hypothetical protein |
|  |  | CarP3651: | RNA polymerase sigma factor |
|  |  | CarP7284: | glgA |
|  |  | CarP6283: | hypothetical protein |
|  |  | CarP7619: | Lanthionine synthetase C-like protein |
|  |  | CarP6431: | hypothetical protein |
|  |  | CarP6086: | hypothetical protein |
|  |  | CarP3371: | Biopolymer transport protein ExbD TolR |
|  |  | CarP6668: | Ribonuclease |
|  |  | CarP7277: | hypothetical protein |
|  |  | CarP7210: | hypothetical protein |
|  |  | CarP5120: | dnaJ |
|  |  | CarP4038: | Glyoxalase-like domain protein |
|  |  | CarP6006: | hypothetical protein |
|  |  | CarP3765: | epsH |
|  |  | CarP5935: | hypothetical protein |
|  |  | CarP7621: | thiol-disulfide oxidoreductase |
|  |  | CarP2719: | carA2 |
|  |  | CarP5530: | fucP |
|  |  | CarP6298: | hypothetical protein |
|  |  | CarP6307: | hypothetical protein |
|  |  | CarP4201: | hypothetical protein |
|  |  | CarP3933: | acuR |
|  |  | CarP4658: | lytR |
|  |  | CarP4391: | hypothetical protein |
|  |  | CarP6941: | hypothetical protein |
|  |  | CarP6750: | hypothetical protein |
|  |  | CarP4826: | hypothetical protein |
|  |  | CarP2182: | hypothetical protein |
|  |  | CarP2423: | nnr |
|  |  | CarP5802: | Trehalose utilisation |
|  |  | CarP4334: | Outer membrane efflux protein |
|  |  | CarP3827: | putative transposase |
|  |  | CarP3384: | hypothetical protein |
|  |  | CarP2406: | gpmA |
|  |  | CarP2471: | sucC |
|  |  | CarP4832: | crtA |
|  |  | CarP4360: | ecsA |
|  |  | CarP4101: | yfgC |
|  |  | CarP2079: | tetratricopeptide repeat protein |
|  |  | CarP2557: | Autoinducer 2 sensor kinase phosphatase Lu |
|  |  | CarP5159: | PhoD-like phosphatase |
|  |  | CarP5233: | hypothetical protein |
|  |  | CarP2651: | hypothetical protein |
|  |  | CarP7664: | smc |
|  |  | CarP2815: | miaE |
|  |  | CarP7606: | madL |
|  |  | CarP7386: | hypothetical protein |
|  |  | CarP4418: | hypothetical protein |
|  |  | CarP2724: | DSBA-like thioredoxin domain protein |
|  |  | CarP4909: | iolE |
|  |  | CarP7464: | Alpha beta hydrolase family protein |
|  |  | CarP4532: | hypothetical protein |
|  |  | CarP4046: | zraR |
|  |  | CarP5329: | epsJ |
|  |  | CarP6618: | hypothetical protein |
|  |  | CarP4362: | Starch-binding associating with outer memb |
|  |  | CarP2145: | guaA |
|  |  | CarP5377: | hypothetical protein |
|  |  | CarP4971: | Arylesterase precursor |
|  |  | CarP1720: | Helix-turn-helix domain protein |
|  |  | CarP5307: | hypothetical protein |
|  |  | CarP3553: | nicR |
|  |  | CarP4169: | zitB |
|  |  | CarP2391: | pglF |
|  |  | CarP5414: | hypothetical protein |
|  |  | CarP7071: | hypothetical protein |
|  |  | CarP4805: | putative ABC transporter ATP-binding protein |
|  |  | CarP4665: | hypothetical protein |
|  |  | CarP6891: | Alpha beta hydrolase family protein |
|  |  | CarP2700: | hpt |
|  |  | CarP6559: | hypothetical protein |
|  |  | CarP2311: | ccmE |
|  |  | CarP2072: | rimO |
|  |  | CarP7533: | pnbA |
|  |  | CarP5760: | atsA |
|  |  | CarP1754: | csgB |
|  |  | CarP5316: | Phage protein Gp37 Gp68 |
|  |  | CarP4158: | uxuA |
|  |  | CarP3943: | galE |
|  |  | CarP5381: | Ppx GppA phosphatase family protein |
|  |  | CarP5121: | Glycosyl transferases group 1 |
|  |  | CarP5374: | recF |
|  |  | CarP3292: | galK |
|  |  | CarP7215: | hypothetical protein |
|  |  | CarP4103: | Heparinase II III-like protein |
|  |  | CarP2422: | yihX |
|  |  | CarP5859: | cmpD |
|  |  | CarP7224: | hypothetical protein |
|  |  | CarP6112: | hypothetical protein |
|  |  | CarP5534: | gdhIV |
|  |  | CarP5349: | divK |
|  |  | CarP3927: | recQ |
|  |  | CarP7004: | TonB-dependent Receptor Plug Domain protei |
|  |  | CarP1719: | hypothetical protein |
|  |  | CarP3210: | Serpin (serine protease inhibitor) |
|  |  | CarP6803: | hypothetical protein |
|  |  | CarP2159: | dctB |
|  |  | CarP6502: | hypothetical protein |
|  |  | CarP7042: | hypothetical protein |
|  |  | CarP4964: | cotSA |
|  |  | CarP2892: | tetA |
|  |  | CarP1809: | bifunctional N-acetylglucosamine-1-phosphate uridyltransferase/glucosamine-1-phosphate acetyltransferase |
|  |  | CarP5545: | yvaA |
|  |  | CarP4972: | gdhI |
|  |  | CarP3720: | membrane-bound lytic transglycosylase F |
|  |  | CarP6124: | hypothetical protein |
|  |  | CarP4131: | hypothetical protein |
|  |  | CarP7011: | hypothetical protein |
|  |  | CarP3981: | iolE |
|  |  | CarP6409: | CAAX amino terminal protease self- immunity |
|  |  | CarP6604: | miaE |
|  |  | CarP4448: | hypothetical protein |
|  |  | CarP1811: | hypothetical protein |
|  |  | CarP2380: | hypothetical protein |
|  |  | CarP7174: | nanA |
|  |  | CarP2712: | Bestrophin, RFP-TM, chloride channel |
|  |  | CarP6781: | Imelysin |
|  |  | CarP3327: | Fatty acid hydroxylase superfamily protein |
|  |  | CarP5946: | hypothetical protein |
|  |  | CarP5451: | fdtB |
|  |  | CarP7167: | hypothetical protein |
|  |  | CarP2866: | gnl |
|  |  | CarP2685: | fabB |
|  |  | CarP2594: | hypothetical protein |
|  |  | CarP7688: | hypothetical protein |
|  |  | CarP7235: | hypothetical protein |
|  |  | CarP5943: | uhpB |
|  |  | CarP6072: | hypothetical protein |
|  |  | CarP2317: | odhB |
|  |  | CarP2610: | hypothetical protein |
|  |  | CarP7426: | hypothetical protein |
|  |  | CarP6870: | xthA |
|  |  | CarP2631: | bphD |
|  |  | CarP5135: | hypothetical protein |
|  |  | CarP6045: | hypothetical protein |
|  |  | CarP6877: | hypothetical protein |
|  |  | CarP5371: | panE |
|  |  | CarP5764: | hypothetical protein |
|  |  | CarP3888: | tolB |
|  |  | CarP4596: | hypothetical protein |
|  |  | CarP3475: | fixQ |
|  |  | CarP7650: | hypothetical protein |
|  |  | CarP6613: | hypothetical protein |
|  |  | CarP1006: | hcpD |
|  |  | CarP7598: | acpP |
|  |  | CarP2357: | hisE |
|  |  | CarP3612: | hypothetical protein |
|  |  | CarP2859: | SusD family protein |
|  |  | CarP6709: | alr |
|  |  | CarP4649: | lagD |
|  |  | CarP3509: | cry |
|  |  | CarP3987: | hypothetical protein |
|  |  | CarP4054: | FG-GAP repeat protein |
|  |  | CarP3745: | YtxH-like protein |
|  |  | CarP7377: | ornithine cyclodeaminase |
|  |  | CarP3708: | ptpA |
|  |  | CarP3461: | nlpI |
|  |  | CarP1605: | Planctomycete cytochrome C |
|  |  | CarP7108: | hypothetical protein |
|  |  | CarP7659: | hypothetical protein |
|  |  | CarP3592: | Putative lumazine-binding protein |
|  |  | CarP3897: | epsH |
|  |  | CarP2565: | comR |
|  |  | CarP5847: | hypothetical protein |
|  |  | CarP2532: | cph1 |
|  |  | CarP6888: | catD |
|  |  | CarP5014: | Starch-binding associating with outer memb |
|  |  | CarP2118: | hypothetical protein |
|  |  | CarP7267: | Glycosyl hydrolase family 92 |
|  |  | CarP7069: | sigL |
|  |  | CarP6879: | pspA |
|  |  | CarP4947: | hypothetical protein |
|  |  | CarP5613: | hypothetical protein |
|  |  | CarP5290: | 2-succinyl-6-hydroxy-2,4-cyclohexadiene-1- |
|  |  | CarP4506: | pelA |
|  |  | CarP3208: | Glyoxylate hydroxypyruvate reductase B |
|  |  | CarP6398: | S1 P1 Nuclease |
|  |  | CarP6156: | bifunctional phosphoglucose phosphomannose |
|  |  | CarP5223: | Polyketide cyclase dehydrase and lipid t |
|  |  | CarP5752: | hypothetical protein |
|  |  | CarP1109: | hypothetical protein |
|  |  | CarP2682: | yigZ |
|  |  | CarP3231: | rpoE |
|  |  | CarP7604: | scpA |
|  |  | CarP7332: | fba |
|  |  | CarP6884: | hypothetical protein |
|  |  | CarP6571: | Polysaccharide deacetylase |
|  |  | CarP5965: | hypothetical protein |
|  |  | CarP3495: | nudK |
|  |  | CarP1860: | Aminopeptidase YwaD precursor |
|  |  | CarP2902: | arfB |
|  |  | CarP1063: | hypothetical protein |
|  |  | CarP5177: | FAD dependent oxidoreductase |
|  |  | CarP7452: | Carboxymuconolactone decarboxylase family |
|  |  | CarP5224: | hypothetical protein |
|  |  | CarP2964: | hypothetical protein |
|  |  | CarP4859: | Membrane transport protein |
|  |  | CarP3345: | nuoM |
|  |  | CarP6552: | Glutaryl-7-aminocephalosporanic-acid acyla |
|  |  | CarP5370: | Enamine imine deaminase |
|  |  | CarP3911: | rspA |
|  |  | CarP1775: | rsfS |
|  |  | CarP5790: | hypothetical protein |
|  |  | CarP1516: | gumD |
|  |  | CarP6580: | Zinc carboxypeptidase |
|  |  | CarP5587: | afr |
|  |  | CarP5541: | hypothetical protein |
|  |  | CarP3268: | hypothetical protein |
|  |  | CarP3643: | axe1 |
|  |  | CarP3465: | moaA |
|  |  | CarP3287: | hypothetical protein |
|  |  | CarP5876: | hypothetical protein |
|  |  | CarP6764: | Flagellin N-methylase |
|  |  | CarP7656: | sdrD |
|  |  | CarP6110: | hypothetical protein |
|  |  | CarP3709: | Transposase |
|  |  | CarP5292: | mdtN |
|  |  | CarP5187: | hypothetical protein |
|  |  | CarP3514: | putative GTPase MT1543 |
|  |  | CarP5236: | Glucose mannose transporter GlcP |
|  |  | CarP3363: | rbsK |
|  |  | CarP3585: | glnS |
|  |  | CarP2816: | rskA |
|  |  | CarP3224: | gnl |
|  |  | CarP5778: | mntH |
|  |  | CarP5017: | hypothetical protein |
|  |  | CarP3251: | comP |
|  |  | CarP4946: | hypothetical protein |
|  |  | CarP3511: | MG2 domain protein |
|  |  | CarP2942: | hypothetical protein |
|  |  | CarP7028: | SnoaL-like domain protein |
|  |  | CarP2407: | resA |
|  |  | CarP1767: | envC |
|  |  | CarP7221: | RES domain protein |
|  |  | CarP5870: | hypothetical protein |
|  |  | CarP4833: | Polysaccharide pyruvyl transferase |
|  |  | CarP2939: | yngG |
|  |  | CarP6740: | hypothetical protein |
|  |  | CarP7748: | Glyoxalase-like domain protein |
|  |  | CarP3288: | yegT |
|  |  | CarP3482: | yedI |
|  |  | CarP1057: | hypothetical protein |
|  |  | CarP7145: | hypothetical protein |
|  |  | CarP1091: | Macrolide export ATP-binding permease protein |
|  |  | CarP4284: | Acetyltransferase (GNAT) family protein |
|  |  | CarP7177: | beta-D-glucuronidase |
|  |  | CarP7616: | devR |
|  |  | CarP1707: | ybaT |
|  |  | CarP5157: | SusD family protein |
|  |  | CarP2070: | thrB |
|  |  | CarP6569: | hypothetical protein |
|  |  | CarP7395: | hypothetical protein |
|  |  | CarP4142: | Alpha beta hydrolase family protein |
|  |  | CarP5388: | hypothetical protein |
|  |  | CarP5941: | hypothetical protein |
|  |  | CarP1758: | IPT TIG domain protein |
|  |  | CarP4545: | yhaH |
|  |  | CarP2596: | hypothetical protein |
|  |  | CarP5426: | hypothetical protein |
|  |  | CarP7472: | ptpA |
|  |  | CarP4460: | kinA |
|  |  | CarP3242: | hypothetical protein |
|  |  | CarP2873: | hypothetical protein |
|  |  | CarP1833: | menA |
|  |  | CarP3183: | N(4)-(Beta-N-acetylglucosaminyl)-L-asparag |
|  |  | CarP6737: | hypothetical protein |
|  |  | CarP4792: | Enamine imine deaminase |
|  |  | CarP3040: | hypothetical protein |
|  |  | CarP2957: | dgkA |
|  |  | CarP6715: | hypothetical protein |
|  |  | CarP4857: | N-acetyldiaminopimelate deacetylase |
|  |  | CarP6330: | hypothetical protein |
|  |  | CarP5206: | thiS |
|  |  | CarP7734: | Inositol 2-dehydrogenase |
|  |  | CarP7098: | hypothetical protein |
|  |  | CarP3011: | hypothetical protein |
|  |  | CarP4591: | Cupin domain protein |
|  |  | CarP7618: | iolE |
|  |  | CarP2534: | rcsC |
|  |  | CarP7614: | Bacterial lipid A biosynthesis acyltransfe |
|  |  | CarP4893: | mepB |
|  |  | CarP7740: | embR |
|  |  | CarP7238: | mntH |
|  |  | CarP5540: | hypothetical protein |
|  |  | CarP5551: | thiM |
|  |  | CarP4626: | hypothetical protein |
|  |  | CarP5992: | Cna protein B-type domain protein |
|  |  | CarP2748: | hypothetical protein |
|  |  | CarP3953: | pbpX |
|  |  | CarP4692: | zraR |
|  |  | CarP5612: | hypothetical protein |
|  |  | CarP3905: | hypothetical protein |
|  |  | CarP2055: | hypothetical protein |
|  |  | CarP6263: | Uracil DNA glycosylase superfamily protein |
|  |  | CarP2395: | trpC |
|  |  | CarP2410: | icaA |
|  |  | CarP6577: | hypothetical protein |
|  |  | CarP3912: | iolC |
|  |  | CarP1209: | hypothetical protein |
|  |  | CarP4419: | 4-hydroxyproline epimerase |
|  |  | CarP2921: | Fatty acid desaturase |
|  |  | CarP4239: | MORN repeat protein |
|  |  | CarP7554: | ptpA |
|  |  | CarP6366: | mscS |
|  |  | CarP1825: | acdS |
|  |  | CarP6567: | hypothetical protein |
|  |  | CarP2480: | argC |
|  |  | CarP4425: | hypothetical protein |
|  |  | CarP6360: | Nucleotidyltransferase domain protein |
|  |  | CarP3883: | nagA |
|  |  | CarP2561: | hyi |
|  |  | CarP2687: | Ribonuclease |
|  |  | CarP2730: | TonB-dependent Receptor Plug Domain protei |
|  |  | CarP3150: | hypothetical protein |
|  |  | CarP7337: | SusD family protein |
|  |  | CarP6096: | arbA |
|  |  | CarP4089: | hypothetical protein |
|  |  | CarP7518: | hypothetical protein |
|  |  | CarP6708: | deoB |
|  |  | CarP6035: | hypothetical protein |
|  |  | CarP2269: | TonB-dependent Receptor Plug Domain protei |
|  |  | CarP7328: | hypothetical protein |
|  |  | CarP5196: | hypothetical protein |
|  |  | CarP3848: | YtxH-like protein |
|  |  | CarP4202: | Bacterial leucyl aminopeptidase precursor |
|  |  | CarP3645: | hypothetical protein |
|  |  | CarP5462: | hypothetical protein |
|  |  | CarP4804: | hypothetical protein |
|  |  | CarP2206: | narL |
|  |  | CarP2319: | hypothetical protein |
|  |  | CarP3234: | Endoribonuclease L-PSP |
|  |  | CarP7713: | fpaP |
|  |  | CarP1917: | lrp |
|  |  | CarP7424: | hypothetical protein |
|  |  | CarP7134: | RNA polymerase sigma factor |
|  |  | CarP2778: | hypothetical protein |
|  |  | CarP5896: | hypothetical protein |
|  |  | CarP4624: | hypothetical protein |
|  |  | CarP2173: | hypothetical protein |
|  |  | CarP1782: | rarA |
|  |  | CarP6711: | argH |
|  |  | CarP3422: | hypothetical protein |
|  |  | CarP6771: | hypothetical protein |
|  |  | CarP4583: | SusD family protein |
|  |  | CarP7148: | RNA polymerase sigma factor |
|  |  | CarP3928: | tqsA |
|  |  | CarP7156: | MobA-like NTP transferase domain protein |
|  |  | CarP6749: | hypothetical protein |
|  |  | CarP1619: | Mannosyl oligosaccharide glucosidase |
|  |  | CarP2272: | ttrB |
|  |  | CarP7074: | hypothetical protein |
|  |  | CarP4069: | ccpA |
|  |  | CarP6016: | hypothetical protein |
|  |  | CarP7220: | Ubiquinone menaquinone biosynthesis C-meth |
|  |  | CarP1708: | btuB |
|  |  | CarP7012: | Patatin-like phospholipase |
|  |  | CarP6938: | Outer membrane efflux protein |
|  |  | CarP3998: | Peptidase M16 inactive domain protein |
|  |  | CarP3415: | hypothetical protein |
|  |  | CarP2914: | ttgF |
|  |  | CarP4468: | ptpA |
|  |  | CarP4463: | mltD |
|  |  | CarP4122: | ykoT |
|  |  | CarP7242: | hypothetical protein |
|  |  | CarP7366: | hypothetical protein |
|  |  | CarP6303: | icaA |
|  |  | CarP7135: | hypothetical protein |
|  |  | CarP2857: | uxuA |
|  |  | CarP3777: | hypothetical protein |
|  |  | CarP7018: | putative sensor-like histidine kinase |
|  |  | CarP5066: | FecR protein |
|  |  | CarP3082: | ytpA |
|  |  | CarP1990: | ald |
|  |  | CarP2402: | hypothetical protein |
|  |  | CarP2790: | hypothetical protein |
|  |  | CarP7050: | hypothetical protein |
|  |  | CarP6653: | hypothetical protein |
|  |  | CarP5111: | hypothetical protein |
|  |  | CarP5843: | hypothetical protein |
|  |  | CarP5826: | hypothetical protein |
|  |  | CarP2666: | hypothetical protein |
|  |  | CarP6376: | short chain dehydrogenase |
|  |  | CarP5853: | hypothetical protein |
|  |  | CarP5773: | siaT |
|  |  | CarP6978: | hypothetical protein |
|  |  | CarP6352: | fur |
|  |  | CarP1770: | algA |
|  |  | CarP3853: | hypothetical protein |
|  |  | CarP4347: | tadA |
|  |  | CarP3978: | ksgA |
|  |  | CarP4867: | ykoV |
|  |  | CarP6710: | hypothetical protein |
|  |  | CarP6592: | pntAA |
|  |  | CarP3059: | afr |
|  |  | CarP2474: | gltA2 |
|  |  | CarP3821: | Phage-related baseplate assembly protein |
|  |  | CarP5221: | hypothetical protein |
|  |  | CarP5149: | tsaC1 |
|  |  | CarP2417: | ydgH |
|  |  | CarP2958: | hypothetical protein |
|  |  | CarP4842: | hypothetical protein |
|  |  | CarP6999: | bphC |
|  |  | CarP5046: | ybgC |
|  |  | CarP3732: | hypothetical protein |
|  |  | CarP1848: | hypothetical protein |
|  |  | CarP3385: | Thermostable monoacylglycerol lipase |
|  |  | CarP6001: | hypothetical protein |
|  |  | CarP1706: | nhaA |
|  |  | CarP7564: | hypothetical protein |
|  |  | CarP6522: | blaZ |
|  |  | CarP4519: | hypothetical protein |
|  |  | CarP2147: | RlpA-like protein precursor |
|  |  | CarP5428: | hypothetical protein |
|  |  | CarP3321: | lolC |
|  |  | CarP5759: | hypothetical protein |
|  |  | CarP5779: | hypothetical protein |
|  |  | CarP4108: | pyrC |
|  |  | CarP4508: | hypothetical protein |
|  |  | CarP7155: | hypothetical protein |
|  |  | CarP6143: | hypothetical protein |
|  |  | CarP5012: | yteT |
|  |  | CarP1729: | hypothetical protein |
|  |  | CarP7317: | actIII |
|  |  | CarP7546: | hypothetical protein |
|  |  | CarP5308: | hypothetical protein |
|  |  | CarP3236: | hypothetical protein |
|  |  | CarP2294: | ispE |
|  |  | CarP6009: | hypothetical protein |
|  |  | CarP5926: | hypothetical protein |
|  |  | CarP1939: | hypothetical protein |
|  |  | CarP3306: | hypothetical protein |
|  |  | CarP5786: | hypothetical protein |
|  |  | CarP7528: | thcD |
|  |  | CarP6007: | hypothetical protein |
|  |  | CarP6523: | sseA |
|  |  | CarP5675: | Bifunctional (p)ppGpp synthase hydrolase r |
|  |  | CarP4326: | Putative esterase |
|  |  | CarP1621: | hypothetical protein |
|  |  | CarP2543: | HEAT repeat protein |
|  |  | CarP3642: | hypothetical protein |
|  |  | CarP4055: | Xylose isomerase-like TIM barrel |
|  |  | CarP7368: | tcdA |
|  |  | CarP7673: | hypothetical protein |
|  |  | CarP3162: | hypothetical protein |
|  |  | CarP5550: | xylB |
|  |  | CarP4910: | cofC |
|  |  | CarP6265: | hypothetical protein |
|  |  | CarP5475: | hypothetical protein |
|  |  | CarP4606: | Transposase |
|  |  | CarP6921: | hypothetical protein |
|  |  | CarP6311: | Sodium:neurotransmitter symporter family p |
|  |  | CarP6243: | rhlG |
|  |  | CarP6094: | hypothetical protein |
|  |  | CarP6623: | yknZ |
|  |  | CarP6379: | pbpE |
|  |  | CarP3757: | albF |
|  |  | CarP1817: | hypothetical protein |
|  |  | CarP1646: | virF |
|  |  | CarP7208: | hypothetical protein |
|  |  | CarP4388: | hypothetical protein |
|  |  | CarP7479: | hypothetical protein |
|  |  | CarP2124: | mutS2 |
|  |  | CarP6383: | sigR |
|  |  | CarP5537: | mevalonate kinase |
|  |  | CarP4831: | tqsA |
|  |  | CarP7082: | Transcriptional regulator PadR-like family |
|  |  | CarP2009: | bioH |
|  |  | CarP7477: | hypothetical protein |
|  |  | CarP7437: | hypothetical protein |
|  |  | CarP5461: | hypothetical protein |
|  |  | CarP2187: | acdA |
|  |  | CarP4027: | cirA |
|  |  | CarP1989: | Transposase IS200 like protein |
|  |  | CarP2350: | putative acetyl-CoA acyltransferase |
|  |  | CarP7420: | hypothetical protein |
|  |  | CarP4078: | Bacterial regulatory proteins, tetR family |
|  |  | CarP3322: | yagE |
|  |  | CarP4511: | atsA |
|  |  | CarP3129: | rpoE |
|  |  | CarP5869: | hypothetical protein |
|  |  | CarP5982: | divJ |
|  |  | CarP3658: | NHL repeat protein |
|  |  | CarP7349: | Alpha beta hydrolase family protein |
|  |  | CarP6506: | hypothetical protein |
|  |  | CarP2106: | hypothetical protein |
|  |  | CarP2753: | ydfG |
|  |  | CarP4877: | hypothetical protein |
|  |  | CarP7358: | pcaH |
|  |  | CarP4144: | cfr |
|  |  | CarP4285: | hypothetical protein |
|  |  | CarP2336: | bifunctional proline dehydrogenase pyrroli |
|  |  | CarP7128: | yheI |
|  |  | CarP4560: | hypothetical protein |
|  |  | CarP5255: | DinB superfamily protein |
|  |  | CarP2482: | argB |
|  |  | CarP1821: | hypothetical protein |
|  |  | CarP6003: | hypothetical protein |
|  |  | CarP4398: | hypothetical protein |
|  |  | CarP3423: | Cyclic nucleotide-binding domain protein |
|  |  | CarP1868: | tatA |
|  |  | CarP7181: | hypothetical protein |
|  |  | CarP1710: | tuaC |
|  |  | CarP6238: | hflK |
|  |  | CarP4068: | ccpA |
|  |  | CarP4250: | nreC |
|  |  | CarP2502: | ribulose-1,5-biphosphate synthetase |
|  |  | CarP4897: | rluA |
|  |  | CarP5739: | hypothetical protein |
|  |  | CarP3081: | icd |
|  |  | CarP1152: | fepA |
|  |  | CarP6249: | korA |
|  |  | CarP5684: | hypothetical protein |
|  |  | CarP4225: | gumD |
|  |  | CarP7741: | von Willebrand factor type A domain protein |
|  |  | CarP2525: | hypothetical protein |
|  |  | CarP6451: | hypothetical protein |
|  |  | CarP4439: | hypothetical protein |
|  |  | CarP7414: | thiol:disulfide interchange protein precur |
|  |  | CarP6773: | hypothetical protein |
|  |  | CarP6791: | hypothetical protein |
|  |  | CarP5608: | hypothetical protein |
|  |  | CarP6269: | NPCBM-associated, NEW3 domain of alpha-gal |
|  |  | CarP2361: | purD |
|  |  | CarP3546: | hypothetical protein |
|  |  | CarP6807: | hypothetical protein |
|  |  | CarP3632: | Amidohydrolase |
|  |  | CarP1780: | ogt |
|  |  | CarP2318: | sucA |
|  |  | CarP5327: | iorB |
|  |  | CarP2804: | Glycosyl hydrolase family 92 |
|  |  | CarP7594: | hypothetical protein |
|  |  | CarP4278: | celI |
|  |  | CarP5610: | mcrB |
|  |  | CarP5254: | hypothetical protein |
|  |  | CarP6514: | tetratricopeptide repeat protein |
|  |  | CarP4872: | yfmJ |
|  |  | CarP7095: | Starch-binding associating with outer memb |
|  |  | CarP4974: | RNA polymerase sigma factor |
|  |  | CarP3334: | hypothetical protein |
|  |  | CarP3072: | hypothetical protein |
|  |  | CarP1818: | trmH |
|  |  | CarP2282: | hypothetical protein |
|  |  | CarP3671: | DNA alkylation repair enzyme |
|  |  | CarP7595: | yknZ |
|  |  | CarP5390: | metal-dependent hydrolase |
|  |  | CarP7445: | bphC |
|  |  | CarP2861: | araC |
|  |  | CarP7669: | yjjL |
|  |  | CarP7008: | Zn-finger in ubiquitin-hydrolases and othe |
|  |  | CarP5756: | hypothetical protein |
|  |  | CarP7671: | hypothetical protein |
|  |  | CarP4323: | guaA |
|  |  | CarP2104: | ybjJ |
|  |  | CarP4793: | yhdN |
|  |  | CarP4378: | hypothetical protein |
|  |  | CarP1971: | dcp |
|  |  | CarP3527: | ndhC |
|  |  | CarP7539: | hypothetical protein |
|  |  | CarP6316: | hypothetical protein |
|  |  | CarP7093: | hypothetical protein |
|  |  | CarP5034: | hypothetical protein |
|  |  | CarP6448: | xecA1 |
|  |  | CarP6612: | Putative esterase |
|  |  | CarP2538: | Glycosyl hydrolase family 109 protein 1 pr |
|  |  | CarP2808: | putative NUDIX hydrolase |
|  |  | CarP5322: | bglA |
|  |  | CarP2188: | pcrB |
|  |  | CarP7662: | hypothetical protein |
|  |  | CarP6858: | hypothetical protein |
|  |  | CarP4647: | hypothetical protein |
|  |  | CarP7346: | mshA |
|  |  | CarP6959: | guaA |
|  |  | CarP2642: | ndhJ |
|  |  | CarP4948: | sigH |
|  |  | CarP6946: | hypothetical protein |
|  |  | CarP6455: | dsbD |
|  |  | CarP4603: | SusD family protein |
|  |  | CarP3115: | Glyoxalase-like domain protein |
|  |  | CarP5001: | hypothetical protein |
|  |  | CarP4374: | kduI |
|  |  | CarP2935: | hypothetical protein |
|  |  | CarP5112: | dgoD |
|  |  | CarP4659: | Helix-turn-helix |
|  |  | CarP3100: | apbE |
|  |  | CarP2943: | hypothetical protein |
|  |  | CarP4307: | hypothetical protein |
|  |  | CarP1752: | cseB |
|  |  | CarP6636: | ycf3 |
|  |  | CarP3684: | hypothetical protein |
|  |  | CarP6041: | CAAX amino terminal protease self- immunity |
|  |  | CarP4785: | Long-chain-fatty-acid--CoA ligase FadD15 |
|  |  | CarP3305: | hypothetical protein |
|  |  | CarP5676: | hypothetical protein |
|  |  | CarP5424: | hypothetical protein |
|  |  | CarP6876: | hypothetical protein |
|  |  | CarP2844: | Thioesterase superfamily protein |
|  |  | CarP4485: | hypothetical protein |
|  |  | CarP3543: | cheB |
|  |  | CarP5583: | hypothetical protein |
|  |  | CarP4075: | chiA1 |
|  |  | CarP3125: | pspC |
|  |  | CarP6372: | hypothetical protein |
|  |  | CarP4233: | hypothetical protein |
|  |  | CarP4191: | hypothetical protein |
|  |  | CarP2855: | fabG |
|  |  | CarP4209: | gno |
|  |  | CarP3689: | virF |
|  |  | CarP4166: | hypothetical protein |
|  |  | CarP2831: | ttgR |
|  |  | CarP7544: | nylB |
|  |  | CarP6306: | hypothetical protein |
|  |  | CarP3374: | srpA |
|  |  | CarP6480: | ybiV |
|  |  | CarP4704: | hypothetical protein |
|  |  | CarP2725: | hypothetical protein |
|  |  | CarP5203: | putative ABC transporter ATP-binding protein |
|  |  | CarP2037: | hisF |
|  |  | CarP3291: | hypothetical protein |
|  |  | CarP1051: | hypothetical protein |
|  |  | CarP3218: | hypothetical protein |
|  |  | CarP7526: | dapE |
|  |  | CarP2157: | yfnB |
|  |  | CarP2508: | cysD |
|  |  | CarP7427: | CDP-alcohol phosphatidyltransferase |
|  |  | CarP6836: | rfaQ |
|  |  | CarP4934: | Plasmid stabilisation system protein |
|  |  | CarP3228: | Major NAD(P)H-flavin oxidoreductase |
|  |  | CarP5033: | mntH |
|  |  | CarP3324: | Ureidoglycolate lyase |
|  |  | CarP5497: | hypothetical protein |
|  |  | CarP3348: | rhlE |
|  |  | CarP3771: | hypothetical protein |
|  |  | CarP4682: | LytTr DNA-binding domain protein |
|  |  | CarP4517: | hypothetical protein |
|  |  | CarP6849: | hypothetical protein |
|  |  | CarP4457: | macA |
|  |  | CarP3457: | B12 binding domain protein |
|  |  | CarP4013: | hypothetical protein |
|  |  | CarP3941: | hypothetical protein |
|  |  | CarP5568: | lineage-specific thermal regulator protein |
|  |  | CarP6262: | hypothetical protein |
|  |  | CarP4153: | kdgK |
|  |  | CarP2298: | leuD |
|  |  | CarP7190: | Calcineurin-like phosphoesterase |
|  |  | CarP6928: | eutB |
|  |  | CarP5526: | rfbE |
|  |  | CarP7561: | Susd and RagB outer membrane lipoprotein |
|  |  | CarP3634: | Neutral alkaline non-lysosomal ceramidase |
|  |  | CarP3523: | nqo2 |
|  |  | CarP5508: | hypothetical protein |
|  |  | CarP5836: | celE |
|  |  | CarP7542: | araC |
|  |  | CarP7162: | hypothetical protein |
|  |  | CarP4219: | ribulose-1,5-biphosphate synthetase |
|  |  | CarP3707: | Helix-turn-helix domain protein |
|  |  | CarP4176: | dhaT |
|  |  | CarP1532: | Transposase DDE domain protein |
|  |  | CarP5178: | atsA |
|  |  | CarP2309: | nadX |
|  |  | CarP6474: | acnB |
|  |  | CarP5492: | Secreted repeat of unknown function |
|  |  | CarP5987: | ABC transporter ATP-binding permease prote |
|  |  | CarP2451: | mca |
|  |  | CarP5051: | gpr |
|  |  | CarP7192: | hypothetical protein |
|  |  | CarP4174: | iorA |
|  |  | CarP3319: | xerD |
|  |  | CarP5799: | hypothetical protein |
|  |  | CarP4204: | argH |
|  |  | CarP6468: | SusD family protein |
|  |  | CarP5053: | idhA |
|  |  | CarP3542: | exbB |
|  |  | CarP3583: | hpd |
|  |  | CarP3222: | psdht |
|  |  | CarP2367: | zraR |
|  |  | CarP7188: | D-tagatose 3-epimerase |
|  |  | CarP6854: | xlnD |
|  |  | CarP6325: | hypothetical protein |
|  |  | CarP3714: | paeR7IM |
|  |  | CarP6800: | hypothetical protein |
|  |  | CarP4921: | rsbW |
|  |  | CarP5651: | iscS |
|  |  | CarP6945: | hypothetical protein |
|  |  | CarP4993: | phyH |
|  |  | CarP2230: | Yqey-like protein |
|  |  | CarP5332: | fpg |
|  |  | CarP2886: | hypothetical protein |
|  |  | CarP6698: | hypothetical protein |
|  |  | CarP6719: | hypothetical protein |
|  |  | CarP3456: | hypothetical protein |
|  |  | CarP7592: | hypothetical protein |
|  |  | CarP5917: | Common central domain of tyrosinase |
|  |  | CarP1515: | hypothetical protein |
|  |  | CarP4023: | hypothetical protein |
|  |  | CarP4004: | Sodium Bile acid symporter family protein |
|  |  | CarP2537: | hypothetical protein |
|  |  | CarP6747: | hypothetical protein |
|  |  | CarP1815: | hypothetical protein |
|  |  | CarP2052: | sucC |
|  |  | CarP4645: | nlpI |
|  |  | CarP7180: | dgdA |
|  |  | CarP3344: | hypothetical protein |
|  |  | CarP6536: | dlgD |
|  |  | CarP6387: | hypothetical protein |
|  |  | CarP2822: | adhB |
|  |  | CarP7283: | acrA |
|  |  | CarP5822: | hypothetical protein |
|  |  | CarP2186: | panD |
|  |  | CarP6508: | chiA1 |
|  |  | CarP6712: | hypothetical protein |
|  |  | CarP4141: | slmA |
|  |  | CarP4680: | Diacylglycerol acyltransferase mycolyltran |
|  |  | CarP7137: | Autoinducer 2 sensor kinase phosphatase Lu |
|  |  | CarP7129: | SusD family protein |
|  |  | CarP2894: | putative TonB-dependent receptor precursor |
|  |  | CarP5035: | amiA |
|  |  | CarP2321: | folP |
|  |  | CarP7080: | ccp |
|  |  | CarP4074: | Stress responsive A B Barrel Domain protei |
|  |  | CarP7367: | hypothetical protein |
|  |  | CarP2398: | trpA |
|  |  | CarP2366: | pyrB |
|  |  | CarP2569: | hypothetical protein |
|  |  | CarP2476: | rhlE |
|  |  | CarP3245: | hypothetical protein |
|  |  | CarP6827: | dnaJ |
|  |  | CarP1804: | ksgA |
|  |  | CarP6582: | Cytochrome c |
|  |  | CarP3934: | ttgR |
|  |  | CarP3258: | TonB protein |
|  |  | CarP4693: | fpaP |
|  |  | CarP4628: | hypothetical protein |
|  |  | CarP7315: | fabG |
|  |  | CarP5312: | phyH |
|  |  | CarP7166: | nlpI |
|  |  | CarP3101: | apbE |
|  |  | CarP2152: | Enamine imine deaminase |
|  |  | CarP3412: | moeB |
|  |  | CarP5044: | crnA |
|  |  | CarP6458: | mshA |
|  |  | CarP6904: | hypothetical protein |
|  |  | CarP4255: | sigW |
|  |  | CarP3041: | hypothetical protein |
|  |  | CarP6241: | Neutral alkaline non-lysosomal ceramidase |
|  |  | CarP6445: | hpf |
|  |  | CarP4554: | hypothetical protein |
|  |  | CarP2438: | SusD family protein |
|  |  | CarP3283: | mrsA |
|  |  | CarP3562: | hypothetical protein |
|  |  | CarP1722: | hypothetical protein |
|  |  | CarP7140: | hypothetical protein |
|  |  | CarP4119: | Cytochrome c |
|  |  | CarP7341: | yhdN |
|  |  | CarP6276: | hrp1 |
|  |  | CarP3784: | hypothetical protein |
|  |  | CarP2379: | serA |
|  |  | CarP2576: | rutD |
|  |  | CarP7597: | hypothetical protein |
|  |  | CarP7536: | paaF |
|  |  | CarP3178: | fadH |
|  |  | CarP7178: | hypothetical protein |
|  |  | CarP2324: | mhqR |
|  |  | CarP5880: | hypothetical protein |
|  |  | CarP3290: | Sodium hydrogen exchanger family protein |
|  |  | CarP1978: | fucP |
|  |  | CarP2054: | hypothetical protein |
|  |  | CarP4073: | Starch-binding associating with outer memb |
|  |  | CarP7109: | hypothetical protein |
|  |  | CarP5425: | NUDIX domain protein |
|  |  | CarP7239: | yfgC |
|  |  | CarP5164: | kefC |
|  |  | CarP5455: | hypothetical protein |
|  |  | CarP4691: | SnoaL-like domain protein |
|  |  | CarP2758: | iolE |
|  |  | CarP5805: | hypothetical protein |
|  |  | CarP5343: | afr |
|  |  | CarP3464: | mobA |
|  |  | CarP2339: | pheA |
|  |  | CarP3882: | sigW |
|  |  | CarP1505: | dps |
|  |  | CarP3706: | hypothetical protein |
|  |  | CarP3688: | hypothetical protein |
|  |  | CarP2164: | hypothetical protein |
|  |  | CarP5456: | nagB |
|  |  | CarP2955: | hypothetical protein |
|  |  | CarP7730: | hypothetical protein |
|  |  | CarP6628: | cotH |
|  |  | CarP2858: | putative lyase |
|  |  | CarP2723: | Divergent AAA domain protein |
|  |  | CarP3289: | hypothetical protein |
|  |  | CarP3532: | plsX |
|  |  | CarP5650: | hypothetical protein |
|  |  | CarP3539: | paaI |
|  |  | CarP3989: | Lycopene cyclase protein |
|  |  | CarP7292: | ahpD |
|  |  | CarP5280: | hypothetical protein |
|  |  | CarP2392: | trpE |
|  |  | CarP7090: | SusD family protein |
|  |  | CarP5957: | Helix-turn-helix domain protein |
|  |  | CarP5780: | hypothetical protein |
|  |  | CarP5906: | hypothetical protein |
|  |  | CarP3349: | apr |
|  |  | CarP7463: | hypothetical protein |
|  |  | CarP2783: | zraR |
|  |  | CarP7161: | hypothetical protein |
|  |  | CarP3381: | hypothetical protein |
|  |  | CarP2741: | zur |
|  |  | CarP4590: | hypothetical protein |
|  |  | CarP2696: | TraB family protein |
|  |  | CarP6102: | hypothetical protein |
|  |  | CarP6954: | hypothetical protein |
|  |  | CarP6136: | hypothetical protein |
|  |  | CarP4849: | hypothetical protein |
|  |  | CarP6857: | hypothetical protein |
|  |  | CarP7063: | ttuB |
|  |  | CarP7439: | hypothetical protein |
|  |  | CarP3531: | gmhB |
|  |  | CarP5523: | D-tagatose 3-epimerase |
|  |  | CarP5401: | coxS |
|  |  | CarP6900: | xynZ |
|  |  | CarP6028: | hypothetical protein |
|  |  | CarP3195: | yrrB |
|  |  | CarP3710: | hypothetical protein |
|  |  | CarP6564: | Glucose Sorbosone dehydrogenase |
|  |  | CarP3755: | xynB |
|  |  | CarP5431: | hypothetical protein |
|  |  | CarP6621: | hypothetical protein |
|  |  | CarP3581: | hypothetical protein |
|  |  | CarP3448: | hypothetical protein |
|  |  | CarP6967: | hypothetical protein |
|  |  | CarP4989: | ywqK |
|  |  | CarP1951: | puo |
|  |  | CarP6152: | hypothetical protein |
|  |  | CarP5142: | Divergent AAA domain protein |
|  |  | CarP7710: | gno |
|  |  | CarP6603: | phyH |
|  |  | CarP4437: | hypothetical protein |
|  |  | CarP1632: | hypothetical protein |
|  |  | CarP7165: | hypothetical protein |
|  |  | CarP7700: | hypothetical protein |
|  |  | CarP7683: | hypothetical protein |
|  |  | CarP7562: | TonB-dependent Receptor Plug Domain protei |
|  |  | CarP6894: | hypothetical protein |
|  |  | CarP2819: | proB |
|  |  | CarP5590: | hypothetical protein |
|  |  | CarP3496: | tetA |
|  |  | CarP6245: | yhdN |
|  |  | CarP5339: | hypothetical protein |
|  |  | CarP4721: | hapE |
|  |  | CarP7321: | mshA |
|  |  | CarP2460: | yhdG |
|  |  | CarP2683: | hypothetical protein |
|  |  | CarP7056: | hypothetical protein |
|  |  | CarP5477: | hypothetical protein |
|  |  | CarP7636: | htrA |
|  |  | CarP6419: | nqrA |
|  |  | CarP2665: | ycgE |
|  |  | CarP4670: | hcaR |
|  |  | CarP3526: | nqo6 |
|  |  | CarP7383: | hutU |
|  |  | CarP2242: | M61 glycyl aminopeptidase |
|  |  | CarP2193: | qdoI |
|  |  | CarP4798: | azu |
|  |  | CarP6924: | oprF |
|  |  | CarP6345: | Carbohydrate acetyl esterase feruloyl este |
|  |  | CarP5396: | lytR |
|  |  | CarP3955: | Surface antigen |
|  |  | CarP3278: | hypothetical protein |
|  |  | CarP3922: | DinB superfamily protein |
|  |  | CarP3031: | hypothetical protein |
|  |  | CarP3950: | hypothetical protein |
|  |  | CarP3584: | Fumarylacetoacetate (FAA) hydrolase family |
|  |  | CarP7711: | hypothetical protein |
|  |  | CarP7516: | hypothetical protein |
|  |  | CarP5216: | Glycine sarcosine N-methyltransferase |
|  |  | CarP5751: | Putative nucleotidyltransferase substrate |
|  |  | CarP5639: | lsrG |
|  |  | CarP3046: | phoR |
|  |  | CarP2087: | spsA |
|  |  | CarP1510: | Fatty acid hydroxylase superfamily protein |
|  |  | CarP2466: | sui1 |
|  |  | CarP3487: | 2OG-Fe(II) oxygenase superfamily protein |
|  |  | CarP3954: | tetA |
|  |  | CarP2316: | ramA |
|  |  | CarP3369: | putative type I restriction enzymeP M prot |
|  |  | CarP1777: | spoVD |
|  |  | CarP2556: | START domain protein |
|  |  | CarP6644: | hypothetical protein |
|  |  | CarP4043: | yhdN |
|  |  | CarP4607: | hypothetical protein |
|  |  | CarP5893: | hypothetical protein |
|  |  | CarP5527: | vapC |
|  |  | CarP4743: | fbaB |
|  |  | CarP6591: | pntA |
|  |  | CarP6226: | Sodium glucose cotransporter |
|  |  | CarP4324: | ybdK |
|  |  | CarP3667: | catD |
|  |  | CarP3075: | Cupin domain protein |
|  |  | CarP4944: | BNR Asp-box repeat protein |
|  |  | CarP4026: | ykfC |
|  |  | CarP1016: | FG-GAP repeat protein |
|  |  | CarP5593: | hypothetical protein |
|  |  | CarP7066: | SusD family protein |
|  |  | CarP4621: | WD domain, G-beta repeat |
|  |  | CarP2746: | hypothetical protein |
|  |  | CarP6872: | O-methyltransferase |
|  |  | CarP5936: | hypothetical protein |
|  |  | CarP7737: | Glyoxalase-like domain protein |
|  |  | CarP7138: | hypothetical protein |
|  |  | CarP5457: | Cupin domain protein |
|  |  | CarP6845: | iscS |
|  |  | CarP5998: | hypothetical protein |
|  |  | CarP6669: | gltP |
|  |  | CarP7476: | hypothetical protein |
|  |  | CarP5638: | ATP-dependent protease La (LON) domain pro |
|  |  | CarP3247: | hypothetical protein |
|  |  | CarP1523: | czcA |
|  |  | CarP5478: | murein L,D-transpeptidase |
|  |  | CarP7026: | yesS |
|  |  | CarP5435: | ybaQ |
|  |  | CarP3223: | lytH |
|  |  | CarP5958: | hypothetical protein |
|  |  | CarP1768: | hypothetical protein |
|  |  | CarP6147: | Helix-turn-helix domain protein |
|  |  | CarP5274: | fabG |
|  |  | CarP5235: | hypothetical protein |
|  |  | CarP3603: | NUMOD4 motif protein |
|  |  | CarP3587: | accD5 |
|  |  | CarP3494: | fyuA |
|  |  | CarP2184: | prmA |
|  |  | CarP6586: | yitF |
|  |  | CarP2171: | anmK |
|  |  | CarP4349: | LysM domain BON superfamily protein |
|  |  | CarP6541: | Glyoxalase-like domain protein |
|  |  | CarP2441: | hypothetical protein |
|  |  | CarP6818: | mrcA |
|  |  | CarP5474: | hypothetical protein |
|  |  | CarP7268: | dadA |
|  |  | CarP3856: | hypothetical protein |
|  |  | CarP5635: | AAA domain (dynein-related subfamily) |
|  |  | CarP2284: | hypothetical protein |
|  |  | CarP2483: | argE |
|  |  | CarP7686: | hypothetical protein |
|  |  | CarP5284: | gpmA |
|  |  | CarP5940: | hypothetical protein |
|  |  | CarP7364: | hypothetical protein |
|  |  | CarP2026: | mpl |
|  |  | CarP2100: | putative acyl-CoA thioester hydrolase |
|  |  | CarP5447: | dpiA |
|  |  | CarP3575: | hypothetical protein |
|  |  | CarP2967: | Integral membrane protein TerC family protein |
|  |  | CarP5190: | hypothetical protein |
|  |  | CarP2806: | mapP |
|  |  | CarP2956: | hypothetical protein |
|  |  | CarP2254: | pyrF |
|  |  | CarP7064: | apbE |
|  |  | CarP6080: | kaiB |
|  |  | CarP1700: | hypothetical protein |
|  |  | CarP6258: | sigW |
|  |  | CarP5948: | hypothetical protein |
|  |  | CarP3012: | hypothetical protein |
|  |  | CarP7105: | hypothetical protein |
|  |  | CarP6465: | crp |
|  |  | CarP5099: | hypothetical protein |
|  |  | CarP6423: | Acetyltransferase (GNAT) family protein |
|  |  | CarP1960: | gmd |
|  |  | CarP2828: | FeoA domain protein |
|  |  | CarP2847: | hypothetical protein |
|  |  | CarP6292: | carbamoyl phosphate synthase-like protein |
|  |  | CarP2754: | yfkJ |
|  |  | CarP3333: | 26 kDa periplasmic immunogenic protein pre |
|  |  | CarP3808: | hypothetical protein |
|  |  | CarP4328: | cphB |
|  |  | CarP6741: | rsbQ |
|  |  | CarP7667: | hemY |
|  |  | CarP3504: | hypothetical protein |
|  |  | CarP3468: | rihC |
|  |  | CarP6411: | hypothetical protein |
|  |  | CarP7280: | hypothetical protein |
|  |  | CarP6478: | hypothetical protein |
|  |  | CarP4346: | hcrB |
|  |  | CarP7120: | hypothetical protein |
|  |  | CarP5611: | TonB-dependent Receptor Plug Domain protei |
|  |  | CarP2170: | coaX |
|  |  | CarP2528: | hilA |
|  |  | CarP4157: | ygbN |
|  |  | CarP3930: | D-tagatose 3-epimerase |
|  |  | CarP3503: | hypothetical protein |
|  |  | CarP6550: | emrA |
|  |  | CarP2801: | pgl |
|  |  | CarP5382: | hypothetical protein |
|  |  | CarP2089: | phoR |
|  |  | CarP3217: | Flavin reductase like domain protein |
|  |  | CarP7404: | hypothetical protein |
|  |  | CarP3939: | sigH |
|  |  | CarP2283: | hypothetical protein |
|  |  | CarP6304: | Sulfotransferase domain protein |
|  |  | CarP2194: | ywqE |
|  |  | CarP3068: | Transposase |
|  |  | CarP4846: | hypothetical protein |
|  |  | CarP7100: | hypothetical protein |
|  |  | CarP4555: | hypothetical protein |
|  |  | CarP3414: | Bifunctional transcriptional activator DNA |
|  |  | CarP4443: | hypothetical protein |
|  |  | CarP5908: | ntrC |
|  |  | CarP7646: | hypothetical protein |
|  |  | CarP4008: | fucP |
|  |  | CarP3159: | ytrE |
|  |  | CarP5405: | PAP2 superfamily protein |
|  |  | CarP4337: | hypothetical protein |
|  |  | CarP5440: | hypothetical protein |
|  |  | CarP4056: | SusD family protein |
|  |  | CarP7470: | ntcA |
|  |  | CarP5213: | Vitamin K-dependent gamma-carboxylase |
|  |  | CarP1899: | Helix-turn-helix domain protein |
|  |  | CarP3491: | EVE domain protein |
|  |  | CarP5379: | hypothetical protein |
|  |  | CarP3973: | rluA |
|  |  | CarP5232: | hyaD |
|  |  | CarP2257: | hypothetical protein |
|  |  | CarP2484: | argH |
|  |  | CarP3110: | yedA |
|  |  | CarP6647: | choD |
|  |  | CarP2377: | hypothetical protein |
|  |  | CarP4310: | ygjH |
|  |  | CarP1798: | recF |
|  |  | CarP5665: | hypothetical protein |
|  |  | CarP3534: | mip |
|  |  | CarP5270: | fabB |
|  |  | CarP2130: | recD2 |
|  |  | CarP7354: | yehU |
|  |  | CarP4134: | nedA |
|  |  | CarP3459: | norG |
|  |  | CarP7323: | rfaQ |
|  |  | CarP6248: | gltB |
|  |  | CarP5055: | thiE |
|  |  | CarP3404: | narB |
|  |  | CarP1918: | SPFH domain Band 7 family protein |
|  |  | CarP3499: | acpP |
|  |  | CarP2322: | aroK |
|  |  | CarP2876: | ywaD |
|  |  | CarP2539: | mshA |
|  |  | CarP5432: | hypothetical protein |
|  |  | CarP6363: | hypothetical protein |
|  |  | CarP4907: | kmo |
|  |  | CarP5098: | doxX |
|  |  | CarP4087: | Fatty acid elongase 3-ketoacyl-CoA synthas |
|  |  | CarP6589: | bepF |
|  |  | CarP1607: | hypothetical protein |
|  |  | CarP1849: | hypothetical protein |
|  |  | CarP2306: | purH |
|  |  | CarP3959: | hypothetical protein |
|  |  | CarP3473: | hypothetical protein |
|  |  | CarP6081: | pnbA |
|  |  | CarP4965: | dgoT |
|  |  | CarP4380: | hypothetical protein |
|  |  | CarP7553: | namA |
|  |  | CarP4416: | hypothetical protein |
|  |  | CarP3362: | hypothetical protein |
|  |  | CarP5399: | exsA |
|  |  | CarP5302: | mshD |
|  |  | CarP3644: | hypothetical protein |
|  |  | CarP7068: | Transcriptional regulator PadR-like family |
|  |  | CarP3596: | hypothetical protein |
|  |  | CarP3835: | lytH |
|  |  | CarP3261: | tsaC1 |
|  |  | CarP3285: | lutB |
|  |  | CarP2963: | acdA |
|  |  | CarP6821: | ykgB |
|  |  | CarP2385: | metF |
|  |  | CarP7527: | hypothetical protein |
|  |  | CarP4773: | putative hydrolase |
|  |  | CarP1854: | ftsE |
|  |  | CarP7449: | czcA |
|  |  | CarP2495: | fixH |
|  |  | CarP1506: | resA |
|  |  | CarP1898: | Glycosyl transferase family 2 |
|  |  | CarP7670: | arlS |
|  |  | CarP2853: | acuC |
|  |  | CarP7173: | RES domain protein |
|  |  | CarP3230: | hypothetical protein |
|  |  | CarP1744: | yiaD |
|  |  | CarP5865: | RDD family protein |
|  |  | CarP6796: | hypothetical protein |
|  |  | CarP5321: | hypothetical protein |
|  |  | CarP6395: | hypothetical protein |
|  |  | CarP7185: | FtsX-like permease family protein |
|  |  | CarP2018: | rmlD |
|  |  | CarP4616: | hypothetical protein |
|  |  | CarP6426: | BNR Asp-box repeat protein |
|  |  | CarP3786: | Planctomycete cytochrome C |
|  |  | CarP5959: | hypothetical protein |
|  |  | CarP4986: | Dienelactone hydrolase family protein |
|  |  | CarP1968: | pchR |
|  |  | CarP6820: | nreC |
|  |  | CarP4190: | hypothetical protein |
|  |  | CarP5963: | hypothetical protein |
|  |  | CarP2968: | hypothetical protein |
|  |  | CarP3116: | Na(+) H(+) antiporter NhaP |
|  |  | CarP2228: | hypothetical protein |
|  |  | CarP2400: | galB |
|  |  | CarP6413: | allB |
|  |  | CarP7117: | acpP |
|  |  | CarP6437: | hypothetical protein |
|  |  | CarP5047: | phyH |
|  |  | CarP4683: | evgS |
|  |  | CarP4563: | hypothetical protein |
|  |  | CarP7666: | Putative bifunctional phosphatase peptidyl |
|  |  | CarP4336: | cyaB |
|  |  | CarP2711: | Membrane dipeptidase (Peptidase family M19 |
|  |  | CarP4566: | hypBA2 |
|  |  | CarP4474: | hypothetical protein |
|  |  | CarP3731: | hypothetical protein |
|  |  | CarP3331: | lolD |
|  |  | CarP3692: | fecR |
|  |  | CarP5781: | hypothetical protein |
|  |  | CarP5304: | hypothetical protein |
|  |  | CarP7198: | hypothetical protein |
|  |  | CarP2549: | hypothetical protein |
|  |  | CarP4446: | moeB |
|  |  | CarP3896: | Trehalase |
|  |  | CarP7382: | nreC |
|  |  | CarP6088: | hypothetical protein |
|  |  | CarP2256: | hypothetical protein |
|  |  | CarP2420: | nfuA |
|  |  | CarP3676: | hypothetical protein |
|  |  | CarP3605: | hypothetical protein |
|  |  | CarP4353: | yjaB |
|  |  | CarP4589: | hypothetical protein |
|  |  | CarP5348: | hypothetical protein |
|  |  | CarP4313: | hypothetical protein |
|  |  | CarP2071: | clpS |
|  |  | CarP7087: | hypothetical protein |
|  |  | CarP2633: | yrrB |
|  |  | CarP2062: | tal |
|  |  | CarP3748: | hypothetical protein |
|  |  | CarP2053: | nudF |
|  |  | CarP2620: | yhbE |
|  |  | CarP6044: | Peroxyureidoacrylate ureidoacrylate amidoh |
|  |  | CarP5867: | fkpA |
|  |  | CarP3206: | rimK |
|  |  | CarP6725: | nicotinamidase pyrazinamidase |
|  |  | CarP2506: | fbaA |
|  |  | CarP3565: | ydcZ |
|  |  | CarP3540: | tetratricopeptide repeat protein |
|  |  | CarP5468: | hypothetical protein |
|  |  | CarP7125: | hypothetical protein |
|  |  | CarP6558: | hypothetical protein |
|  |  | CarP2940: | glgA |
|  |  | CarP4028: | ytrA |
|  |  | CarP2832: | czcA |
|  |  | CarP5747: | hypothetical protein |
|  |  | CarP3205: | hypothetical protein |
|  |  | CarP4279: | lcp |
|  |  | CarP3243: | Cytochrome c |
|  |  | CarP6546: | hypothetical protein |
|  |  | CarP2169: | hypothetical protein |
|  |  | CarP2835: | DinB superfamily protein |
|  |  | CarP2149: | rhlE |
|  |  | CarP4663: | hcrB |
|  |  | CarP4183: | yehU |
|  |  | CarP4837: | hypothetical protein |
|  |  | CarP5961: | hypothetical protein |
|  |  | CarP4045: | hypothetical protein |
|  |  | CarP7249: | lrp |
|  |  | CarP6105: | hypothetical protein |
|  |  | CarP4903: | NADPH-dependent FMN reductase |
|  |  | CarP3267: | coaX |
|  |  | CarP4155: | pucA |
|  |  | CarP1829: | gycine oxidase |
|  |  | CarP5184: | glpQ1 |
|  |  | CarP7468: | bbsF |
|  |  | CarP6859: | nrtA |
|  |  | CarP6120: | Curli production assembly transport compon |
|  |  | CarP1793: | yajC |
|  |  | CarP3992: | Phospholipase Carboxylesterase |
|  |  | CarP7259: | rfbG |
|  |  | CarP7216: | hypothetical protein |
|  |  | CarP3220: | phoR |
|  |  | CarP2376: | hpf |
|  |  | CarP7387: | hypothetical protein |
|  |  | CarP6053: | hypothetical protein |
|  |  | CarP3167: | galM |
|  |  | CarP7460: | hypothetical protein |
|  |  | CarP6476: | gpr |
|  |  | CarP3910: | parD1 |
|  |  | CarP4200: | hemY |
|  |  | CarP4930: | hypothetical protein |
|  |  | CarP5964: | hypothetical protein |
|  |  | CarP2300: | recN |
|  |  | CarP3844: | bepF |
|  |  | CarP4194: | Serine threonine-protein kinase pkn1 |
|  |  | CarP4547: | hypothetical protein |
|  |  | CarP1718: | Sodium, potassium, lithium and rubidium H( |
|  |  | CarP2491: | kdsB |
|  |  | CarP2901: | hypothetical protein |
|  |  | CarP7652: | hypothetical protein |
|  |  | CarP6103: | hypothetical protein |
|  |  | CarP6625: | hypothetical protein |
|  |  | CarP6282: | hypothetical protein |
|  |  | CarP4911: | Bacterial transcription activator, effecto |
|  |  | CarP7255: | hypothetical protein |
|  |  | CarP3502: | hypothetical protein |
|  |  | CarP4381: | hypothetical protein |
|  |  | CarP4928: | cdd |
|  |  | CarP5945: | hypothetical protein |
|  |  | CarP6639: | hypothetical protein |
|  |  | CarP3440: | pbpE |
|  |  | CarP7119: | Outer membrane efflux protein |
|  |  | CarP7040: | cytR |
|  |  | CarP2343: | folB |
|  |  | CarP4029: | 2',5' RNA ligase family |
|  |  | CarP3463: | cfr |
|  |  | CarP6931: | deaD |
|  |  | CarP7400: | ypdA |
|  |  | CarP7649: | hypothetical protein |
|  |  | CarP5538: | bglA |
|  |  | CarP4143: | Putative neutral zinc metallopeptidase |
|  |  | CarP3014: | hypothetical protein |
|  |  | CarP6678: | hypothetical protein |
|  |  | CarP3945: | hypothetical protein |
|  |  | CarP1828: | ydiI |
|  |  | CarP2208: | hypothetical protein |
|  |  | CarP2060: | sigW |
|  |  | CarP5844: | RDD family protein |
|  |  | CarP6652: | hypothetical protein |
|  |  | CarP3903: | afr |
|  |  | CarP7755: | sauU |
|  |  | CarP5846: | hypothetical protein |
|  |  | CarP4556: | lytR |
|  |  | CarP5303: | hypothetical protein |
|  |  | CarP5350: | cph1 |
|  |  | CarP2721: | gntP |
|  |  | CarP2267: | hypothetical protein |
|  |  | CarP2889: | ggt |
|  |  | CarP5989: | hypothetical protein |
|  |  | CarP3169: | hypothetical protein |
|  |  | CarP6742: | rsbQ |
|  |  | CarP2365: | pyrR |
|  |  | CarP7314: | Membrane transport protein |
|  |  | CarP6396: | hypothetical protein |
|  |  | CarP1883: | ttgA |
|  |  | CarP3892: | bicA |
|  |  | CarP7057: | hypothetical protein |
|  |  | CarP4435: | hypothetical protein |
|  |  | CarP3092: | rfbE |
|  |  | CarP2220: | purE |
|  |  | CarP3451: | yhdG |
|  |  | CarP4320: | mhqO |
|  |  | CarP4433: | pitA |
|  |  | CarP7324: | Major Facilitator Superfamily protein |
|  |  | CarP3536: | glnII |
|  |  | CarP6092: | hypothetical protein |
|  |  | CarP4440: | BlaR1 peptidase M56 |
|  |  | CarP5148: | bfce |
|  |  | CarP1893: | yvdD |
|  |  | CarP1913: | entS |
|  |  | CarP2096: | bamA |
|  |  | CarP6584: | cydB |
|  |  | CarP3964: | gfo |
|  |  | CarP7462: | SusD family protein |
|  |  | CarP6691: | hypothetical protein |
|  |  | CarP2670: | yxeP |
|  |  | CarP3408: | Oxidoreductase family, NAD-binding Rossman |
|  |  | CarP2467: | hisG |
|  |  | CarP6442: | hypothetical protein |
|  |  | CarP5929: | dnaE2 |
|  |  | CarP5262: | hypothetical protein |
|  |  | CarP1772: | hypothetical protein |
|  |  | CarP6166: | hypothetical protein |
|  |  | CarP6489: | hypothetical protein |
|  |  | CarP4889: | ydjA |
|  |  | CarP5787: | hypothetical protein |
|  |  | CarP6084: | hypothetical protein |
|  |  | CarP4271: | NmrA-like family protein |
|  |  | CarP6570: | hypothetical protein |
|  |  | CarP7514: | L-Ala-D L-Glu epimerase |
|  |  | CarP6696: | PhoD-like phosphatase |
|  |  | CarP7374: | hypothetical protein |
|  |  | CarP6642: | parD4 |
|  |  | CarP7471: | nuoL |
|  |  | CarP5820: | phoD |
|  |  | CarP1627: | phoV |
|  |  | CarP6689: | hypothetical protein |
|  |  | CarP5828: | hypothetical protein |
|  |  | CarP6645: | lutP |
|  |  | CarP6420: | nqrA |
|  |  | CarP2794: | Serine threonine-protein kinase AfsK |
|  |  | CarP2540: | SusD family protein |
|  |  | CarP2669: | nlhH |
|  |  | CarP7566: | hypothetical protein |
|  |  | CarP7265: | hypothetical protein |
|  |  | CarP2349: | purC |
|  |  | CarP7718: | hypothetical protein |
|  |  | CarP5437: | SusD family protein |
|  |  | CarP4818: | hypothetical protein |
|  |  | CarP6477: | nhaX |
|  |  | CarP7200: | hypothetical protein |
|  |  | CarP5950: | hypothetical protein |
|  |  | CarP1680: | hypothetical protein |
|  |  | CarP6942: | hypothetical protein |
|  |  | CarP2163: | hepA |
|  |  | CarP2830: | nemA |
|  |  | CarP7092: | pdtaS |
|  |  | CarP5161: | cph1 |
|  |  | CarP5493: | yycF |
|  |  | CarP3611: | hypothetical protein |
|  |  | CarP4800: | hypothetical protein |
|  |  | CarP4173: | hypothetical protein |
|  |  | CarP7233: | Glycosyl transferases group 1 |
|  |  | CarP5392: | hypothetical protein |
|  |  | CarP4808: | guaB |
|  |  | CarP6953: | LemA family protein |
|  |  | CarP3823: | N(1)-aminopropylagmatine ureohydrolase |
|  |  | CarP4226: | hypothetical protein |
|  |  | CarP5862: | hypothetical protein |
|  |  | CarP2217: | TPR repeat-containing protein precursor |
|  |  | CarP2807: | hypothetical protein |
|  |  | CarP2419: | ribBA |
|  |  | CarP3560: | hypothetical protein |
|  |  | CarP2390: | vat |
|  |  | CarP6556: | hutG |
|  |  | CarP7286: | pgl |
|  |  | CarP6438: | Alpha beta hydrolase family protein |
|  |  | CarP4561: | hypothetical protein |
|  |  | CarP4966: | Glycosyl hydrolases family 2, sugar bindin |
|  |  | CarP1950: | hipB |
|  |  | CarP3900: | hypothetical protein |
|  |  | CarP6731: | dsdX |
|  |  | CarP5546: | putative two-component response-regulatory |
|  |  | CarP4159: | proA |
|  |  | CarP6533: | hypothetical protein |
|  |  | CarP4681: | hypothetical protein |
|  |  | CarP5185: | Glyoxalase-like domain protein |
|  |  | CarP5788: | hypothetical protein |
|  |  | CarP6326: | hypothetical protein |
|  |  | CarP7703: | hypothetical protein |
|  |  | CarP4894: | Type I phosphodiesterase nucleotide pyro |
|  |  | CarP4582: | hypothetical protein |
|  |  | CarP7124: | Putative endoglucanase |
|  |  | CarP4915: | AhpC TSA family protein |
|  |  | CarP6495: | hypothetical protein |
|  |  | CarP1049: | cotH |
|  |  | CarP6972: | petJ |
|  |  | CarP6990: | Glycosyl transferase family 11 |
|  |  | CarP5249: | ptpA |
|  |  | CarP1853: | fmt |
|  |  | CarP3915: | Planctomycete cytochrome C |
|  |  | CarP4261: | ATP-binding region |
|  |  | CarP6460: | glnB |
|  |  | CarP4712: | Xylose isomerase-like TIM barrel |
|  |  | CarP7525: | hypothetical protein |
|  |  | CarP5366: | nosD |
|  |  | CarP4994: | htpX |
|  |  | CarP7637: | colA |
|  |  | CarP5818: | hypothetical protein |
|  |  | CarP2644: | ndhC |
|  |  | CarP6910: | hypothetical protein |
|  |  | CarP5496: | Gene 25-like lysozyme |
|  |  | CarP3744: | hypothetical protein |
|  |  | CarP4623: | Plasmid stabilisation system protein |
|  |  | CarP1639: | xdhA |
|  |  | CarP3887: | TMAO DMSO reductase |
|  |  | CarP6637: | hypothetical protein |
|  |  | CarP2016: | yrrB |
|  |  | CarP7574: | hypothetical protein |
|  |  | CarP7541: | pcaG |
|  |  | CarP7535: | car |
|  |  | CarP4960: | rsbW |
|  |  | CarP5225: | hypothetical protein |
|  |  | CarP3036: | ybhS |
|  |  | CarP2895: | Beta alpha-amylase precursor |
|  |  | CarP5607: | liaS |
|  |  | CarP3394: | hypothetical protein |
|  |  | CarP5086: | Sodium Bile acid symporter family protein |
|  |  | CarP3813: | putative ABC transporter ATP-binding protein |
|  |  | CarP4922: | hypothetical protein |
|  |  | CarP4156: | hpcH |
|  |  | CarP6049: | hypothetical protein |
|  |  | CarP5039: | hypothetical protein |
|  |  | CarP5222: | hypothetical protein |
|  |  | CarP3405: | narT |
|  |  | CarP4787: | bioF |
|  |  | CarP3773: | hypothetical protein |
|  |  | CarP2429: | cobB |
|  |  | CarP7560: | cotH |
|  |  | CarP3736: | cph1 |
|  |  | CarP4325: | hypothetical protein |
|  |  | CarP3337: | punA |
|  |  | CarP2926: | Sulfite exporter TauE SafE |
|  |  | CarP4968: | hypothetical protein |
|  |  | CarP2757: | sdaA |
|  |  | CarP4862: | GDSL-like Lipase Acylhydrolase |
|  |  | CarP5909: | IPT TIG domain protein |
|  |  | CarP4276: | arginyl-tRNA-protein transferase |
|  |  | CarP7699: | hypothetical protein |
|  |  | CarP4231: | YceI-like domain protein |
|  |  | CarP5960: | hypothetical protein |
|  |  | CarP6294: | metE |
|  |  | CarP1957: | cheR |
|  |  | CarP4815: | DsrE DsrF-like family protein |
|  |  | CarP4351: | hypothetical protein |
|  |  | CarP1986: | hypothetical protein |
|  |  | CarP6758: | hypothetical protein |
|  |  | CarP2625: | nqo4 |
|  |  | CarP4331: | hypothetical protein |
|  |  | CarP4738: | Bacterial transcription activator, effector |
|  |  | CarP5904: | sigL |
|  |  | CarP3429: | KHG KDPG aldolase |
|  |  | CarP3693: | hypothetical protein |
|  |  | CarP5146: | Ion channel |
|  |  | CarP5796: | IstB-like ATP binding protein |
|  |  | CarP6459: | nhaX |
|  |  | CarP2679: | yagE |
|  |  | CarP4786: | hypothetical protein |
|  |  | CarP4863: | bamA |
|  |  | CarP7558: | hypothetical protein |
|  |  | CarP6607: | hypothetical protein |
|  |  | CarP6943: | hypothetical protein |
|  |  | CarP2489: | hypothetical protein |
|  |  | CarP4161: | truC |
|  |  | CarP5217: | tehB |
|  |  | CarP2007: | hypothetical protein |
|  |  | CarP5378: | hypothetical protein |
|  |  | CarP2698: | recombination and DNA strand exchange inhi |
|  |  | CarP3037: | DinB superfamily protein |
|  |  | CarP5663: | N-glycosyltransferase |
|  |  | CarP1033: | engB |
|  |  | CarP6587: | hypothetical protein |
|  |  | CarP7010: | oatA |
|  |  | CarP2263: | succinate dehydrogenase fumarate reductase |
|  |  | CarP2463: | mepM |
|  |  | CarP7642: | lieA |
|  |  | CarP6272: | aspartoacylase |
|  |  | CarP3023: | Auracyanin-A precursor |
|  |  | CarP7725: | hypothetical protein |
|  |  | CarP7423: | hypothetical protein |
|  |  | CarP5570: | hypothetical protein |
|  |  | CarP3253: | deoxyguanosinetriphosphate triphosphohydrolase-like protein |
|  |  | CarP6543: | hypothetical protein |
|  |  | CarP4092: | alsT |
|  |  | CarP5050: | hypothetical protein |
|  |  | CarP6131: | hypothetical protein |
|  |  | CarP5544: | hypothetical protein |
|  |  | CarP6163: | splB |
|  |  | CarP5073: | Xylose isomerase-like TIM barrel |
|  |  | CarP6540: | hypothetical protein |
|  |  | CarP7750: | hypothetical protein |
|  |  | CarP7062: | hypothetical protein |
|  |  | CarP5744: | hypothetical protein |
|  |  | CarP6766: | hypothetical protein |
|  |  | CarP5100: | galM |
|  |  | CarP4602: | RteC protein |
|  |  | CarP6917: | hypothetical protein |
|  |  | CarP4426: | Leucyl phenylalanyl-tRNA--protein transfer |
|  |  | CarP3055: | hypothetical protein |
|  |  | CarP6101: | Virulence-associated protein E |
|  |  | CarP5359: | Papain family cysteine protease |
|  |  | CarP2354: | PAP2 superfamily protein |
|  |  | CarP7617: | ptpA |
|  |  | CarP6939: | Acyl CoA binding protein |
|  |  | CarP6753: | hypothetical protein |
|  |  | CarP5152: | Sodium Bile acid symporter family protein |
|  |  | CarP1697: | frdA |
|  |  | CarP1776: | mrdB |
|  |  | CarP6670: | hypothetical protein |
|  |  | CarP4357: | rhlE |
|  |  | CarP7577: | CAAX amino terminal protease self- immunity |
|  |  | CarP6432: | hypothetical protein |
|  |  | CarP3332: | Macrolide export ATP-binding permease prot |
|  |  | CarP6145: | hypothetical protein |
|  |  | CarP6601: | araB |
|  |  | CarP4969: | hypothetical protein |
|  |  | CarP4525: | nanA |
|  |  | CarP6046: | DinB family protein |
|  |  | CarP2107: | purB |
|  |  | CarP7360: | hypothetical protein |
|  |  | CarP6062: | hypothetical protein |
|  |  | CarP2674: | ctb |
|  |  | CarP7352: | Zinc carboxypeptidase |
|  |  | CarP2715: | hypothetical protein |
|  |  | CarP2587: | chb |
|  |  | CarP2796: | AIG2-like family protein |
|  |  | CarP4870: | hypothetical protein |
|  |  | CarP6485: | hypothetical protein |
|  |  | CarP4207: | idhA |
|  |  | CarP7039: | ypdA |
|  |  | CarP5575: | purP |
|  |  | CarP2277: | coxM |
|  |  | CarP5188: | hypothetical protein |
|  |  | CarP7184: | xylB |
|  |  | CarP6656: | hypothetical protein |
|  |  | CarP7370: | TonB-dependent Receptor Plug Domain protei |
|  |  | CarP3681: | SusD family protein |
|  |  | CarP4198: | afr |
|  |  | CarP5833: | hypothetical protein |
|  |  | CarP5586: | hypothetical protein |
|  |  | CarP5115: | hypothetical protein |
|  |  | CarP1988: | Starch-binding associating with outer memb |
|  |  | CarP3389: | hypothetical protein |
|  |  | CarP5169: | rpoE |
|  |  | CarP6893: | hypothetical protein |
|  |  | CarP4983: | Putative phosphatase |
|  |  | CarP4322: | hypothetical protein |
|  |  | CarP6993: | Divergent AAA domain protein |
|  |  | CarP2598: | Integrase core domain protein |
|  |  | CarP1779: | mreC |
|  |  | CarP6809: | hypothetical protein |
|  |  | CarP5883: | hypothetical protein |
|  |  | CarP2205: | yehT |
|  |  | CarP5061: | hypothetical protein |
|  |  | CarP7102: | hypothetical protein |
|  |  | CarP5389: | putative inner membrane protein |
|  |  | CarP7658: | hypothetical protein |
|  |  | CarP4405: | hypothetical protein |
|  |  | CarP6805: | Fatty acid desaturase |
|  |  | CarP6412: | O-acetylhomoserine aminocarboxypropyltrans |
|  |  | CarP5386: | hypothetical protein |
|  |  | CarP4263: | kdpA |
|  |  | CarP6310: | hypothetical protein |
|  |  | CarP5775: | siaP |
|  |  | CarP5306: | hypothetical protein |
|  |  | CarP2431: | hypothetical protein |
|  |  | CarP4421: | Serine threonine exchanger SteT |
|  |  | CarP5504: | hypothetical protein |
|  |  | CarP4296: | hypothetical protein |
|  |  | CarP6748: | hypothetical protein |
|  |  | CarP2023: | cshA |
|  |  | CarP2326: | luxE |
|  |  | CarP5580: | nicB |
|  |  | CarP4260: | hypothetical protein |
|  |  | CarP2996: | Sodium glucose cotransporter |
|  |  | CarP7073: | hypothetical protein |
|  |  | CarP6098: | hypothetical protein |
|  |  | CarP6524: | hypothetical protein |
|  |  | CarP2134: | hypothetical protein |
|  |  | CarP2448: | trpF |
|  |  | CarP4423: | kduD |
|  |  | CarP7569: | hypothetical protein |
|  |  | CarP5395: | hypothetical protein |
|  |  | CarP1095: | bvgS |
|  |  | CarP3397: | ntdC |
|  |  | CarP2487: | uppP |
|  |  | CarP5011: | hypBA1 |
|  |  | CarP7316: | fgd1 |
|  |  | CarP6548: | Outer membrane efflux protein |
|  |  | CarP5108: | F5 8 type C domain protein |
|  |  | CarP4696: | hypothetical protein |
|  |  | CarP2898: | 2-acyl-glycerophospho-ethanolamine acyltra |
|  |  | CarP3042: | Protein of unknown function (Porph ging) |
|  |  | CarP3561: | OsmC-like protein |
|  |  | CarP4811: | xecD |
|  |  | CarP3033: | hypothetical protein |
|  |  | CarP5133: | kipA |
|  |  | CarP1955: | putative nucleotide-binding protein, conta |
|  |  | CarP3606: | mutB |
|  |  | CarP5793: | hypothetical protein |
|  |  | CarP2204: | ATPase family associated with various cell |
|  |  | CarP4574: | hypothetical protein |
|  |  | CarP4126: | mgsA |
|  |  | CarP6125: | hypothetical protein |
|  |  | CarP5085: | 6-N-hydroxylaminopurine resistance protein |
|  |  | CarP2369: | acpH |
|  |  | CarP2716: | nagB |
|  |  | CarP2900: | 7TM diverse intracellular signalling |
|  |  | CarP1647: | Quercetin 2,3-dioxygenase |
|  |  | CarP2464: | tktA |
|  |  | CarP4806: | hypothetical protein |
|  |  | CarP7376: | hypothetical protein |
|  |  | CarP7648: | OPT oligopeptide transporter protein |
|  |  | CarP5598: | Putative esterase |
|  |  | CarP2488: | ribF |
|  |  | CarP7706: | SMP-30 Gluconolaconase LRE-like region |
|  |  | CarP3013: | bglX |
|  |  | CarP6886: | hypothetical protein |
|  |  | CarP6123: | hypothetical protein |
|  |  | CarP3099: | EamA-like transporter family protein |
|  |  | CarP1959: | hypothetical protein |
|  |  | CarP5123: | xerC |
|  |  | CarP2803: | f1pep1 |
|  |  | CarP5436: | hypothetical protein |
|  |  | CarP5122: | Helix-turn-helix domain protein |
|  |  | CarP6055: | fixL |
|  |  | CarP4179: | SusD family protein |
|  |  | CarP1945: | arlS |
|  |  | CarP2518: | hypothetical protein |
|  |  | CarP4882: | rfbF |
|  |  | CarP5364: | nosZ |
|  |  | CarP7555: | hypothetical protein |
|  |  | CarP2450: | yidK |
|  |  | CarP4531: | RNA polymerase sigma factor |
|  |  | CarP7511: | cirA |
|  |  | CarP6452: | cirA |
|  |  | CarP7191: | oprF |
|  |  | CarP6575: | TonB-dependent Receptor Plug Domain protei |
|  |  | CarP7432: | amyB |
|  |  | CarP5367: | putative ABC transporter ATP-binding protein |
|  |  | CarP4427: | arlS |
|  |  | CarP7020: | iphP |
|  |  | CarP4967: | F5 8 type C domain protein |
|  |  | CarP3382: | otsA |
|  |  | CarP7419: | hypothetical protein |
|  |  | CarP6119: | csgF |
|  |  | CarP2166: | tatA |
|  |  | CarP2259: | hypothetical protein |
|  |  | CarP4850: | Glycosyl hydrolase family 57 |
|  |  | CarP3189: | ybaL |
|  |  | CarP3093: | hypothetical protein |
|  |  | CarP6798: | hypothetical protein |
|  |  | CarP3179: | paiA |
|  |  | CarP5192: | NAD dependent epimerase dehydratase family |
|  |  | CarP2550: | murein L,D-transpeptidase |
|  |  | CarP7678: | 5'-nucleotidase |
|  |  | CarP2075: | hypothetical protein |
|  |  | CarP2136: | kdsA |
|  |  | CarP7114: | kynB |
|  |  | CarP2786: | arcA |
|  |  | CarP3044: | hypothetical protein |
|  |  | CarP3923: | hypothetical protein |
|  |  | CarP3967: | fabG |
|  |  | CarP3426: | hypothetical protein |
|  |  | CarP4661: | mak |
|  |  | CarP4830: | fadA |
|  |  | CarP7702: | hypothetical protein |
|  |  | CarP5533: | hypothetical protein |
|  |  | CarP2618: | GH3 auxin-responsive promoter |
|  |  | CarP1850: | oprF |
|  |  | CarP6994: | hypothetical protein |
|  |  | CarP5762: | ywqK |
|  |  | CarP4625: | hypothetical protein |
|  |  | CarP1941: | sigW |
|  |  | CarP5003: | Na(+) H(+) antiporter subunit F |
|  |  | CarP5973: | rcp1 |
|  |  | CarP2439: | hypothetical protein |
|  |  | CarP6697: | panE |
|  |  | CarP6666: | prs |
|  |  | CarP4358: | yccS |
|  |  | CarP2127: | von Willebrand factor type A domain protei |
|  |  | CarP3074: | Bacterial alpha-L-rhamnosidase |
|  |  | CarP7305: | pyrC |
|  |  | CarP6048: | qdoI |
|  |  | CarP1679: | hypothetical protein |
|  |  | CarP2751: | hypothetical protein |
|  |  | CarP4885: | Nucleotidyltransferase domain protein |
|  |  | CarP6624: | FtsX-like permease family protein |
|  |  | CarP6693: | hypothetical protein |
|  |  | CarP4268: | 5'-methylthioadenosine S-adenosylhomocyste |
|  |  | CarP6726: | nicotinamidase pyrazinamidase |
|  |  | CarP5678: | hcpC |
|  |  | CarP3407: | phoA |
|  |  | CarP4810: | hypothetical protein |
|  |  | CarP1637: | cdhR |
|  |  | CarP2129: | hypothetical protein |
|  |  | CarP3739: | cpdA |
|  |  | CarP3659: | hypothetical protein |
|  |  | CarP6677: | hypothetical protein |
|  |  | CarP3122: | dac |
|  |  | CarP5547: | hypothetical protein |
|  |  | CarP3343: | hypothetical protein |
|  |  | CarP4527: | hypothetical protein |
|  |  | CarP3070: | hypothetical protein |
|  |  | CarP7201: | hypothetical protein |
|  |  | CarP6073: | cbgA |
|  |  | CarP7085: | sigY |
|  |  | CarP5002: | Na(+) H(+) antiporter subunit G |
|  |  | CarP4059: | SusD family protein |
|  |  | CarP4034: | lldR |
|  |  | CarP6039: | hypothetical protein |
|  |  | CarP4024: | fabB |
|  |  | CarP3410: | Fatty acid hydroxylase superfamily protein |
|  |  | CarP2966: | corA |
|  |  | CarP5285: | hypothetical protein |
|  |  | CarP4110: | putative oxidoreductase MSMEI 2347 |
|  |  | CarP7643: | hypothetical protein |
|  |  | CarP7350: | hypothetical protein |
|  |  | CarP5383: | hypothetical protein |
|  |  | CarP4618: | hypothetical protein |
|  |  | CarP3352: | hypothetical protein |
|  |  | CarP3654: | Starch-binding associating with outer memb |
|  |  | CarP4060: | TonB dependent receptor |
|  |  | CarP2993: | hypothetical protein |
|  |  | CarP3462: | hypothetical protein |
|  |  | CarP6268: | ABC-2 family transporter protein |
|  |  | CarP2818: | proA |
|  |  | CarP1846: | gltP |
|  |  | CarP6236: | hypothetical protein |
|  |  | CarP7003: | hypothetical protein |
|  |  | CarP2313: | ccsA |
|  |  | CarP7517: | recombination protein F |
|  |  | CarP7623: | hypothetical protein |
|  |  | CarP3172: | iron-responsive transcriptional regulator |
|  |  | CarP7043: | hypothetical protein |
|  |  | CarP3564: | hypothetical protein |
|  |  | CarP3586: | Helix-turn-helix domain protein |
|  |  | CarP5352: | hypothetical protein |
|  |  | CarP4615: | hypothetical protein |
|  |  | CarP2231: | hypothetical protein |
|  |  | CarP3003: | hypothetical protein |
|  |  | CarP5951: | yjaB |
|  |  | CarP7348: | patB |
|  |  | CarP5768: | bcp |
|  |  | CarP2006: | quiP |
|  |  | CarP7053: | pdxH |
|  |  | CarP2058: | hypothetical protein |
|  |  | CarP7727: | cas6 |
|  |  | CarP6435: | hypothetical protein |
|  |  | CarP2397: | trpB |
|  |  | CarP6739: | araC |
|  |  | CarP5811: | Receptor L domain protein |
|  |  | CarP5601: | hypothetical protein |
|  |  | CarP5525: | yehT |
|  |  | CarP7033: | uvrA |
|  |  | CarP5641: | hypothetical protein |
|  |  | CarP1616: | ccp |
|  |  | CarP3791: | nudG |
|  |  | CarP6356: | hypothetical protein |
|  |  | CarP4678: | Zinc carboxypeptidase |
|  |  | CarP1832: | bcp |
|  |  | CarP2500: | ppk |
|  |  | CarP4065: | gfo |
|  |  | CarP4796: | SMP-30 Gluconolaconase LRE-like region |
|  |  | CarP1022: | bepG |
|  |  | CarP2359: | sadH |
|  |  | CarP4280: | Rifampin ADP-ribosyl transferase |
|  |  | CarP2499: | pacS |
|  |  | CarP5873: | yehU |
|  |  | CarP3350: | hypBA1 |
|  |  | CarP3312: | hypothetical protein |
|  |  | CarP4553: | hypothetical protein |
|  |  | CarP7457: | ATPase family associated with various cell |
|  |  | CarP3578: | hypothetical protein |
|  |  | CarP1635: | nanT |
|  |  | CarP4932: | hypothetical protein |
|  |  | CarP2146: | slcC |
|  |  | CarP4236: | hypothetical protein |
|  |  | CarP5835: | hypothetical protein |
|  |  | CarP2615: | hypothetical protein |
|  |  | CarP7144: | yehT |
|  |  | CarP5325: | yedY |
|  |  | CarP7000: | DinB superfamily protein |
|  |  | CarP4314: | hypothetical protein |
|  |  | CarP4417: | Acyltransferase |
|  |  | CarP2105: | gloB |
|  |  | CarP3376: | NADH oxidase |
|  |  | CarP5770: | hypothetical protein |
|  |  | CarP4775: | fbpC |
|  |  | CarP2667: | cycA |
|  |  | CarP5263: | hypothetical protein |
|  |  | CarP5471: | hypothetical protein |
|  |  | CarP2722: | hypothetical protein |
|  |  | CarP2864: | hdc |
|  |  | CarP5937: | SusD family protein |
|  |  | CarP3870: | ctrA |
|  |  | CarP5777: | Endoribonuclease L-PSP |
|  |  | CarP2516: | glpQ1 |
|  |  | CarP5000: | hypothetical protein |
|  |  | CarP3358: | epsN |
|  |  | CarP3480: | katE |
|  |  | CarP3597: | arfA |
|  |  | CarP4489: | Ankyrin repeat protein |
|  |  | CarP2192: | mnmC |
|  |  | CarP4115: | maa |
|  |  | CarP7605: | Malonate sodium symporter MadM subunit |
|  |  | CarP4399: | phoR |
|  |  | CarP2877: | gnd |
|  |  | CarP3239: | aroB |
|  |  | CarP5144: | putative TonB-dependent receptor precursor |
|  |  | CarP6260: | hypothetical protein |
|  |  | CarP2084: | tolB |
|  |  | CarP6264: | hypothetical protein |
|  |  | CarP2381: | hypothetical protein |
|  |  | CarP7336: | hypothetical protein |
|  |  | CarP2424: | fixL |
|  |  | CarP7257: | PGL p-HBAD biosynthesis glycosyltransferas |
|  |  | CarP5208: | ypdA |
|  |  | CarP1734: | ybaQ |
|  |  | CarP2235: | Retaining alpha-galactosidase precursor |
|  |  | CarP4807: | hypothetical protein |
|  |  | CarP2621: | hypothetical protein |
|  |  | CarP6450: | MORN repeat variant |
|  |  | CarP4160: | cbpA |
|  |  | CarP3826: | idi |
|  |  | CarP4217: | hypothetical protein |
|  |  | CarP6270: | adh |
|  |  | CarP2199: | udk |
|  |  | CarP4210: | hypothetical protein |
|  |  | CarP2950: | hypothetical protein |
|  |  | CarP2619: | yumC |
|  |  | CarP7256: | epsF |
|  |  | CarP1888: | mrsA |
|  |  | CarP4799: | hypothetical protein |
|  |  | CarP2086: | hypothetical protein |
|  |  | CarP5761: | RNA polymerase sigma factor |
|  |  | CarP7684: | abgB |
|  |  | CarP6407: | ptrA |
|  |  | CarP4783: | hypothetical protein |
|  |  | CarP2875: | hypothetical protein |
|  |  | CarP7345: | btuB |
|  |  | CarP1207: | hypothetical protein |
|  |  | CarP4040: | lolD |
|  |  | CarP5990: | hypothetical protein |
|  |  | CarP5543: | Sugar-binding cellulase-like protein |
|  |  | CarP5162: | fixL |
|  |  | CarP3841: | Macrolide export ATP-binding permease prot |
|  |  | CarP5409: | cysJ |
|  |  | CarP3948: | hypothetical protein |
|  |  | CarP4940: | mazF |
|  |  | CarP3276: | phaJ |
|  |  | CarP4335: | mdtB |
|  |  | CarP7032: | Glyoxalase-like domain protein |
|  |  | CarP3154: | hypothetical protein |
|  |  | CarP3299: | xylR |
|  |  | CarP6675: | YHS domain protein |
|  |  | CarP2546: | hypothetical protein |
|  |  | CarP3885: | Gellan lyase precursor |
|  |  | CarP4669: | hypothetical protein |
|  |  | CarP3545: | dnaK |
|  |  | CarP6472: | hypothetical protein |
|  |  | CarP3687: | btuB |
|  |  | CarP5914: | araC |
|  |  | CarP5419: | qdoI |
|  |  | CarP5863: | hypothetical protein |
|  |  | CarP7599: | alkaline phosphatase isozyme conversion am |
|  |  | CarP1531: | hypothetical protein |
|  |  | CarP3061: | sdrD |
|  |  | CarP4010: | rhmA |
|  |  | CarP2545: | inlJ |
|  |  | CarP6745: | hypothetical protein |
|  |  | CarP4364: | Starch-binding associating with outer memb |
|  |  | CarP4386: | hypothetical protein |
|  |  | CarP1620: | exuT |
|  |  | CarP3262: | xylA |
|  |  | CarP2174: | lysC |
|  |  | CarP4395: | glcR |
|  |  | CarP4689: | hypothetical protein |
|  |  | CarP7570: | ypdA |
|  |  | CarP2116: | yjmC |
|  |  | CarP5918: | hmp |
|  |  | CarP7743: | hypothetical protein |
|  |  | CarP3774: | NADP oxidoreductase coenzyme F420-dependen |
|  |  | CarP1882: | hypothetical protein |
|  |  | CarP7390: | xynZ |
|  |  | CarP2860: | bglK |
|  |  | CarP3403: | hrb |
|  |  | CarP5398: | hypothetical protein |
|  |  | CarP5567: | lineage-specific thermal regulator protein |
|  |  | CarP5083: | tolB |
|  |  | CarP4224: | wzxC |
|  |  | CarP7738: | pknJ |
|  |  | CarP6757: | hypothetical protein |
|  |  | CarP2874: | Macrolide export ATP-binding permease prot |
|  |  | CarP3393: | Starch-binding associating with outer memb |
|  |  | CarP4823: | smeA |
|  |  | CarP3535: | hypothetical protein |
|  |  | CarP7310: | hypothetical protein |
|  |  | CarP5566: | hypothetical protein |
|  |  | CarP6932: | hypothetical protein |
|  |  | CarP6077: | csd |
|  |  | CarP2975: | hypothetical protein |
|  |  | CarP7585: | yiaD |
|  |  | CarP4379: | hypothetical protein |
|  |  | CarP4569: | arsC2 |
|  |  | CarP2609: | hypothetical protein |
|  |  | CarP6359: | hypothetical protein |
|  |  | CarP5502: | Undecaprenyl-phosphate mannosyltransferase |
|  |  | CarP7369: | ycfH |
|  |  | CarP6808: | Low molecular weight phosphotyrosine prote |
|  |  | CarP6934: | dnaJ |
|  |  | CarP5346: | yfkN |
|  |  | CarP5354: | cphA |
|  |  | CarP2479: | argG |
|  |  | CarP4953: | Two component regulator propeller |
|  |  | CarP5766: | hypothetical protein |
|  |  | CarP6908: | Polysaccharide biosynthesis protein |
|  |  | CarP7573: | hypothetical protein |
|  |  | CarP2919: | ubiB |
|  |  | CarP4540: | hypothetical protein |
|  |  | CarP5491: | hypothetical protein |
|  |  | CarP7038: | hypothetical protein |
|  |  | CarP2091: | hypothetical protein |
|  |  | CarP1730: | kup |
|  |  | CarP6358: | iolC |
|  |  | CarP5059: | xyoA |
|  |  | CarP2934: | glpQ |
|  |  | CarP4106: | hypothetical protein |
|  |  | CarP5978: | CAAX amino terminal protease self- immunity |
|  |  | CarP5482: | hypothetical protein |
|  |  | CarP5194: | Outer membrane efflux protein |
|  |  | CarP2531: | hypothetical protein |
|  |  | CarP2849: | phoA |
|  |  | CarP2718: | crtB |
|  |  | CarP2891: | hypothetical protein |
|  |  | CarP6083: | kdpE |
|  |  | CarP4541: | hypothetical protein |
|  |  | CarP6777: | Transposase zinc-ribbon domain protein |
|  |  | CarP7086: | tolB |
|  |  | CarP6770: | hypothetical protein |
|  |  | CarP6337: | GDSL-like Lipase Acylhydrolase |
|  |  | CarP5266: | lipoprotein chaperone |
|  |  | CarP7099: | tolB |
|  |  | CarP5481: | Galactose methyl galactoside import ATP-bi |
|  |  | CarP4740: | alkA |
|  |  | CarP6424: | H(+) Cl(-) exchange transporter ClcA |
|  |  | CarP3300: | Zinc carboxypeptidase |
|  |  | CarP2442: | bicA |
|  |  | CarP4585: | fecR |
|  |  | CarP7308: | pytH |
|  |  | CarP7151: | hypothetical protein |
|  |  | CarP2729: | Putative oxidoreductase MT0587 |
|  |  | CarP3703: | hypothetical protein |
|  |  | CarP6346: | hypothetical protein |
|  |  | CarP3225: | desA1 |
|  |  | CarP6887: | cdhR |
|  |  | CarP6600: | nrnA |
|  |  | CarP7153: | dUMP phosphatase |
|  |  | CarP3894: | hchA |
|  |  | CarP2418: | surE |
|  |  | CarP4719: | hypothetical protein |
|  |  | CarP5829: | hypothetical protein |
|  |  | CarP3931: | iolE |
|  |  | CarP6723: | hypothetical protein |
|  |  | CarP5101: | Putative oxidoreductase MT0587 |
|  |  | CarP7723: | hypothetical protein |
|  |  | CarP7568: | hypothetical protein |
|  |  | CarP1969: | iolE |
|  |  | CarP2271: | Class III cytochrome C family protein |
|  |  | CarP6760: | thiamine-phosphate pyrophosphorylase |
|  |  | CarP6841: | hypothetical protein |
|  |  | CarP3762: | hypothetical protein |
|  |  | CarP6802: | hypothetical protein |
|  |  | CarP6933: | hypothetical protein |
|  |  | CarP4576: | RNA polymerase sigma factor |
|  |  | CarP4413: | hypothetical protein |
|  |  | CarP6579: | moeB |
|  |  | CarP4664: | glcA |
|  |  | CarP2745: | hypothetical protein |
|  |  | CarP2113: | putative ABC transporter ATP-binding protein |
|  |  | CarP6853: | nicC |
|  |  | CarP2744: | Acetyltransferase (GNAT) family protein |
|  |  | CarP6111: | rhaR |
|  |  | CarP6965: | hypothetical protein |
|  |  | CarP7653: | hypothetical protein |
|  |  | CarP5642: | hypothetical protein |
|  |  | CarP3318: | hypothetical protein |
|  |  | CarP4402: | hypothetical protein |
|  |  | CarP6060: | RES domain protein |
|  |  | CarP3524: | nqo4 |
|  |  | CarP2789: | putative ABC transporter ATP-binding protein |
|  |  | CarP7672: | Zeta toxin |
|  |  | CarP3555: | aceF |
|  |  | CarP7440: | hypothetical protein |
|  |  | CarP5687: | hypothetical protein |
|  |  | CarP5131: | LamB YcsF family protein |
|  |  | CarP6234: | CDP-alcohol phosphatidyltransferase |
|  |  | CarP2223: | exoA |
|  |  | CarP4954: | hypothetical protein |
|  |  | CarP6868: | hypothetical protein |
|  |  | CarP4708: | gdhA |
|  |  | CarP4513: | Cytochrome c |
|  |  | CarP3413: | Type I phosphodiesterase nucleotide pyro |
|  |  | CarP2291: | hypothetical protein |
|  |  | CarP5890: | ardA |
|  |  | CarP5647: | hypothetical protein |
|  |  | CarP2297: | leuC |
|  |  | CarP7739: | PP2C-family Ser Thr phosphatase |
|  |  | CarP5334: | glgE1 |
|  |  | CarP3088: | rfbX |
|  |  | CarP5643: | hypothetical protein |
|  |  | CarP4192: | TonB-dependent Receptor Plug Domain protei |
|  |  | CarP2731: | hypothetical protein |
|  |  | CarP4099: | hypothetical protein |
|  |  | CarP2265: | Rhomboid family protein |
|  |  | CarP2905: | hutI |
|  |  | CarP2155: | sir |
|  |  | CarP3505: | hldE |
|  |  | CarP3591: | Acetyl- propionyl-coenzyme A carboxylase a |
|  |  | CarP3194: | hypothetical protein |
|  |  | CarP4390: | SnoaL-like domain protein |
|  |  | CarP4228: | hypothetical protein |
|  |  | CarP5013: | nagB |
|  |  | CarP5067: | TDP-fucosamine acetyltransferase |
|  |  | CarP3484: | Membrane bound L-sorbosone dehydrogenase |
|  |  | CarP6529: | tolQ |
|  |  | CarP5026: | rihC |
|  |  | CarP4701: | hypothetical protein |
|  |  | CarP5096: | nfnB |
|  |  | CarP7552: | hypothetical protein |
|  |  | CarP2437: | speA |
|  |  | CarP4856: | yrbG |
|  |  | CarP7024: | crp |
|  |  | CarP2364: | carB |
|  |  | CarP1967: | hypothetical protein |
|  |  | CarP3380: | hypothetical protein |
|  |  | CarP6318: | truA |
|  |  | CarP6992: | glgB |
|  |  | CarP5210: | ycf3 |
|  |  | CarP2821: | fabG |
|  |  | CarP2946: | Vacuole effluxer Atg22 like protein |
|  |  | CarP5518: | Murein DD-endopeptidase MepS Murein LD-car |
|  |  | CarP7422: | czcR |
|  |  | CarP5887: | hypothetical protein |
|  |  | CarP4213: | fabG |
|  |  | CarP7551: | Zeta toxin |
|  |  | CarP6054: | Thioredoxin-like protein |
|  |  | CarP3834: | fadJ |
|  |  | CarP3935: | hypothetical protein |
|  |  | CarP6499: | Pectic acid lyase |
|  |  | CarP3942: | YhhN-like protein |
|  |  | CarP6349: | SusD family protein |
|  |  | CarP4552: | hypothetical protein |
|  |  | CarP2481: | argF' |
|  |  | CarP5810: | hypothetical protein |
|  |  | CarP3734: | hypothetical protein |
|  |  | CarP5794: | hypothetical protein |
|  |  | CarP7047: | hypothetical protein |
|  |  | CarP4302: | Two component regulator propeller |
|  |  | CarP4677: | hypothetical protein |
|  |  | CarP7645: | Electron transfer DM13 |
|  |  | CarP7403: | hypothetical protein |
|  |  | CarP4973: | gfo |
|  |  | CarP5977: | hypothetical protein |
|  |  | CarP5915: | nlhH |
|  |  | CarP3123: | glnQ |
|  |  | CarP7410: | hypothetical protein |
|  |  | CarP1795: | Glyoxalase-like domain protein |
|  |  | CarP4703: | hypothetical protein |
|  |  | CarP4604: | Thermophilic serine proteinase precursor |
|  |  | CarP3238: | hypothetical protein |
|  |  | CarP5193: | hypothetical protein |
|  |  | CarP2255: | pyrD |
|  |  | CarP3398: | pglI |
|  |  | CarP3341: | SusD family protein |
|  |  | CarP3879: | idhA |
|  |  | CarP4851: | glgA |
|  |  | CarP6444: | hypothetical protein |
|  |  | CarP5602: | hypothetical protein |
|  |  | CarP2693: | hypothetical protein |
|  |  | CarP7169: | hypothetical protein |
|  |  | CarP7469: | hypothetical protein |
|  |  | CarP5901: | hypothetical protein |
|  |  | CarP5153: | hypothetical protein |
|  |  | CarP5147: | hypothetical protein |
|  |  | CarP6964: | hypothetical protein |
|  |  | CarP5323: | opuBB |
|  |  | CarP3847: | btrR |
|  |  | CarP4428: | rhaT |
|  |  | CarP1031: | ltrA |
|  |  | CarP2095: | hypothetical protein |
|  |  | CarP4866: | Calcineurin-like phosphoesterase |
|  |  | CarP7371: | Sulfite exporter TauE SafE |
|  |  | CarP2551: | Heavy-metal-associated domain protein |
|  |  | CarP1975: | tnpB |
|  |  | CarP4601: | zraR |
|  |  | CarP6743: | bioH |
|  |  | CarP4235: | hypothetical protein |
|  |  | CarP3193: | hypothetical protein |
|  |  | CarP6632: | suhB |
|  |  | CarP2125: | hypothetical protein |
|  |  | CarP4732: | smc |
|  |  | CarP3441: | ypdB |
|  |  | CarP3541: | PBP superfamily domain protein |
|  |  | CarP5886: | hypothetical protein |
|  |  | CarP2908: | cirA |
|  |  | CarP7588: | kdsC |
|  |  | CarP5134: | hypothetical protein |
|  |  | CarP5027: | Nucleotidyltransferase substrate binding p |
|  |  | CarP6790: | Sulfite exporter TauE SafE |
|  |  | CarP6780: | hypothetical protein |
|  |  | CarP6109: | Major royal jelly protein |
|  |  | CarP7327: | acdA |
|  |  | CarP2810: | hypothetical protein |
|  |  | CarP4102: | hypothetical protein |
|  |  | CarP4294: | hypothetical protein |
|  |  | CarP3273: | iolE |
|  |  | CarP6913: | hypothetical protein |
|  |  | CarP3694: | hypothetical protein |
|  |  | CarP6317: | DinB superfamily protein |
|  |  | CarP2676: | nupX |
|  |  | CarP7116: | hypothetical protein |
|  |  | CarP2382: | bamA |
|  |  | CarP6164: | hypothetical protein |
|  |  | CarP7532: | 2,6-dihydropseudooxynicotine hydrolase |
|  |  | CarP6952: | yiaA |
|  |  | CarP4295: | hypothetical protein |
|  |  | CarP4890: | hypothetical protein |
|  |  | CarP6817: | hypothetical protein |
|  |  | CarP4145: | Putative DNA ligase-like protein MT0965 |
|  |  | CarP5114: | hypothetical protein |
|  |  | CarP6025: | hypothetical protein |
|  |  | CarP6863: | fabG |
|  |  | CarP2301: | hypothetical protein |
|  |  | CarP4444: | hypothetical protein |
|  |  | CarP4926: | Starch-binding associating with outer memb |
|  |  | CarP5402: | hypothetical protein |
|  |  | CarP5116: | degU |
|  |  | CarP6585: | gnl |
|  |  | CarP4249: | icd |
|  |  | CarP5623: | SusD family protein |
|  |  | CarP3390: | araB |
|  |  | CarP3152: | nagD |
|  |  | CarP3246: | hypothetical protein |
|  |  | CarP6683: | hypothetical protein |
|  |  | CarP3590: | Secretory lipase |
|  |  | CarP7691: | fadD |
|  |  | CarP4697: | hypothetical protein |
|  |  | CarP5054: | Trm112p-like protein |
|  |  | CarP2890: | Outer membrane efflux protein |
|  |  | CarP7060: | hypothetical protein |
|  |  | CarP5421: | rihA |
|  |  | CarP1761: | araC |
|  |  | CarP3570: | hypothetical protein |
|  |  | CarP4452: | yycG |
|  |  | CarP5813: | dac |
|  |  | CarP2507: | Bifunctional enzyme CysN CysC |
|  |  | CarP6549: | mdtA |
|  |  | CarP2750: | hypothetical protein |
|  |  | CarP6948: | Dihydrolipoyl dehydrogenase |
|  |  | CarP3216: | linC |
|  |  | CarP2452: | ifcA |
|  |  | CarP2893: | Polyphosphate kinase 2 (PPK2) |
|  |  | CarP5515: | hypothetical protein |
|  |  | CarP3478: | ugl |
|  |  | CarP5985: | hypothetical protein |
|  |  | CarP6297: | hypothetical protein |
|  |  | CarP2341: | hypothetical protein |
|  |  | CarP5930: | hypothetical protein |
|  |  | CarP4112: | hypothetical protein |
|  |  | CarP4246: | hypothetical protein |
|  |  | CarP4365: | malP |
|  |  | CarP7679: | hypothetical protein |
|  |  | CarP5204: | hypothetical protein |
|  |  | CarP3188: | alkH |
|  |  | CarP2607: | mrsA |
|  |  | CarP5315: | hypothetical protein |
|  |  | CarP2080: | mltD |
|  |  | CarP7726: | pat |
|  |  | CarP2123: | putative ABC transporter ATP-binding protein |
|  |  | CarP1737: | osmC |
|  |  | CarP2773: | hypothetical protein |
|  |  | CarP5191: | fecR |
|  |  | CarP5211: | hypothetical protein |
|  |  | CarP4961: | Putative anti-sigma factor antagonist |
|  |  | CarP7107: | fpaP |
|  |  | CarP3071: | SnoaL-like polyketide cyclase |
|  |  | CarP6374: | galE |
|  |  | CarP4165: | pbpE |
|  |  | CarP3366: | camB |
|  |  | CarP7065: | hxlR |
|  |  | CarP5136: | Calcineurin-like phosphoesterase |
|  |  | CarP3458: | yvaA |
|  |  | CarP3293: | Fic DOC family protein |
|  |  | CarP4919: | hypothetical protein |
|  |  | CarP1023: | cenC |
|  |  | CarP1699: | hypothetical protein |
|  |  | CarP5127: | Starch-binding associating with outer memb |
|  |  | CarP2867: | fadB2 |
|  |  | CarP3977: | Putative endoglucanase |
|  |  | CarP6061: | hypothetical protein |
|  |  | CarP5498: | hypothetical protein |
|  |  | CarP2092: | ptrA |
|  |  | CarP5522: | apc3 |
|  |  | CarP7183: | yoaC |
|  |  | CarP6911: | hypothetical protein |
|  |  | CarP4880: | crtD |
|  |  | CarP4829: | yjcF |
|  |  | CarP7576: | hypothetical protein |
|  |  | CarP7728: | cas3 |
|  |  | CarP7113: | pepN |
|  |  | CarP7513: | Inward rectifier potassium channel Kirbac3 |
|  |  | CarP2838: | Endonuclease Exonuclease phosphatase famil |
|  |  | CarP2805: | hypothetical protein |
|  |  | CarP7289: | hypothetical protein |
|  |  | CarP1878: | leuA |
|  |  | CarP4385: | hypothetical protein |
|  |  | CarP1948: | Phage Tail Collar Domain protein |
|  |  | CarP2492: | L-Ala-D L-Glu epimerase |
|  |  | CarP4876: | hypothetical protein |
|  |  | CarP5814: | hypothetical protein |
|  |  | CarP6903: | Bax inhibitor 1 like protein |
|  |  | CarP5353: | FRG domain protein |
|  |  | CarP2604: | Alpha-L-fucosidase |
|  |  | CarP3980: | resA |
|  |  | CarP4844: | hypothetical protein |
|  |  | CarP7695: | nlhH |
|  |  | CarP4321: | nreB |
|  |  | CarP1701: | GIY-YIG nuclease superfamily protein |
|  |  | CarP6576: | xynB |
|  |  | CarP3254: | crp |
|  |  | CarP7326: | Outer membrane efflux protein |
|  |  | CarP2261: | purM |
|  |  | CarP2383: | vioB |
|  |  | CarP7754: | fadA |
|  |  | CarP6449: | Two component regulator propeller |
|  |  | CarP2738: | hypothetical protein |
|  |  | CarP3263: | iolE |
|  |  | CarP3650: | atsA |
|  |  | CarP7045: | HD domain protein |
|  |  | CarP2268: | ybaQ |
|  |  | CarP2878: | SusD family protein |
|  |  | CarP5466: | arlS |
|  |  | CarP2061: | hypothetical protein |
|  |  | CarP1909: | acoD |
|  |  | CarP7447: | N-glycosyltransferase |
|  |  | CarP4137: | putative FAD-binding dehydrogenase |
|  |  | CarP4170: | xseA |
|  |  | CarP6497: | TM2 domain protein |
|  |  | CarP6378: | Trifunctional NAD biosynthesis regulator p |
|  |  | CarP6824: | yhbU |
|  |  | CarP5176: | hypothetical protein |
|  |  | CarP6390: | hypothetical protein |
|  |  | CarP5275: | Acyl-coenzyme A:6-aminopenicillanic acid a |
|  |  | CarP4588: | btr |
|  |  | CarP1690: | Transposase IS200 like protein |
|  |  | CarP1824: | pkn1 |
|  |  | CarP1831: | hypothetical protein |
|  |  | CarP1943: | hypothetical protein |
|  |  | CarP6661: | hypothetical protein |
|  |  | CarP6682: | hypothetical protein |
|  |  | CarP3320: | cpnA |
|  |  | CarP5040: | hypothetical protein |
|  |  | CarP1589: | Stress responsive A B Barrel Domain protein |
|  |  | CarP3796: | azr |
|  |  | CarP6823: | hypothetical protein |
|  |  | CarP5501: | btuB |
|  |  | CarP6405: | cycA |
|  |  | CarP1874: | tagH |
|  |  | CarP3076: | yehT |
|  |  | CarP4865: | Trehalose utilisation |
|  |  | CarP2195: | galE |
|  |  | CarP2577: | hypothetical protein |
|  |  | CarP5407: | narL |
|  |  | CarP2752: | Alpha-pyrone synthesis polyketide synthase |
|  |  | CarP2947: | hypothetical protein |
|  |  | CarP6619: | hypothetical protein |
|  |  | CarP2469: | hisC |
|  |  | CarP6050: | Fatty acid elongase 3-ketoacyl-CoA synthas |
|  |  | CarP6651: | hypothetical protein |
|  |  | CarP1910: | nfdA |
|  |  | CarP1841: | ndx1 |
|  |  | CarP2733: | msrC |
|  |  | CarP4424: | uxuA |
|  |  | CarP6784: | Calx-beta domain protein |
|  |  | CarP7270: | hypothetical protein |
|  |  | CarP4031: | hypothetical protein |
|  |  | CarP6744: | uxaB |
|  |  | CarP3674: | hypothetical protein |
|  |  | CarP5129: | hypothetical protein |
|  |  | CarP5743: | hypothetical protein |
|  |  | CarP6664: | Rhodanese-like domain protein |
|  |  | CarP1867: | Glyoxalase-like domain protein |
|  |  | CarP2603: | hypothetical protein |
|  |  | CarP4037: | atsA |
|  |  | CarP5772: | Amidohydrolase |
|  |  | CarP6782: | Cytochrome c |
|  |  | CarP1740: | RHS Repeat protein |
|  |  | CarP3726: | hypothetical protein |
|  |  | CarP4684: | tmoS |
|  |  | CarP2153: | lysA |
|  |  | CarP2503: | pgcA |
|  |  | CarP6850: | hypothetical protein |
|  |  | CarP6525: | arlS |
|  |  | CarP4672: | Putative acetyltransferase |
|  |  | CarP4052: | acr1 |
|  |  | CarP6174: | hypothetical protein |
|  |  | CarP2128: | hypothetical protein |
|  |  | CarP3538: | hypothetical protein |
|  |  | CarP2094: | phnP |
|  |  | CarP4205: | yesS |
|  |  | CarP6789: | hypothetical protein |
|  |  | CarP2777: | glpF |
|  |  | CarP2916: | gltB |
|  |  | CarP1110: | hypothetical protein |
|  |  | CarP2225: | fadN |
|  |  | CarP3944: | hypothetical protein |
|  |  | CarP1072: | gcd |
|  |  | CarP3296: | ywaD |
|  |  | CarP5539: | cpnA |
|  |  | CarP1987: | SusD family protein |
|  |  | CarP3665: | Transposase IS200 like protein |
|  |  | CarP4163: | cirA |
|  |  | CarP6690: | Neutral alkaline non-lysosomal ceramidase |
|  |  | CarP2962: | hypothetical protein |
|  |  | CarP7401: | Polysaccharide deacetylase |
|  |  | CarP7363: | hypothetical protein |
|  |  | CarP6688: | kstR2 |
|  |  | CarP3274: | hypothetical protein |
|  |  | CarP1640: | arlS |
|  |  | CarP3425: | mtfA |
|  |  | CarP1587: | hypothetical protein |
|  |  | CarP1916: | hypothetical protein |
|  |  | CarP4564: | hypothetical protein |
|  |  | CarP5536: | gtaB |
|  |  | CarP6907: | Enamine imine deaminase |
|  |  | CarP5562: | hypothetical protein |
|  |  | CarP1608: | patA |
|  |  | CarP2290: | sigW |
|  |  | CarP4875: | scpB |
|  |  | CarP6351: | hflC |
|  |  | CarP2435: | hypothetical protein |
|  |  | CarP4356: | hypothetical protein |
|  |  | CarP6013: | hypothetical protein |
|  |  | CarP5626: | Alpha-L-fucosidase |
|  |  | CarP7753: | hypothetical protein |
|  |  | CarP4363: | VRR-NUC domain protein |
|  |  | CarP7751: | hypothetical protein |
|  |  | CarP6382: | hypothetical protein |
|  |  | CarP5956: | hypothetical protein |
|  |  | CarP5243: | Peptidase M16 inactive domain protein |
|  |  | CarP4002: | egtD |
|  |  | CarP5384: | pdxH |
|  |  | CarP7580: | NAD binding domain of 6-phosphogluconate d |
|  |  | CarP2334: | cysE |
|  |  | CarP7712: | hypothetical protein |
|  |  | CarP6240: | nlhH |
|  |  | CarP6122: | hypothetical protein |
|  |  | CarP6078: | Serine threonine-protein kinase pkn1 |
|  |  | CarP5900: | hypothetical protein |
|  |  | CarP6239: | hflK |
|  |  | CarP5832: | iucA |
|  |  | CarP3563: | plsB |
|  |  | CarP1876: | hypothetical protein |
|  |  | CarP4017: | pchR |
|  |  | CarP6266: | hypothetical protein |
|  |  | CarP5595: | citN |
|  |  | CarP2850: | arnC |
|  |  | CarP5682: | hypothetical protein |
|  |  | CarP2568: | hypothetical protein |
|  |  | CarP4469: | hypothetical protein |
|  |  | CarP3377: | araD |
|  |  | CarP6806: | arcB |
|  |  | CarP6754: | ycgJ |
|  |  | CarP3588: | cfiA |
|  |  | CarP4542: | hypothetical protein |
|  |  | CarP7640: | hypothetical protein |
|  |  | CarP2664: | hypothetical protein |
|  |  | CarP5333: | Transcriptional regulator PadR-like family |
|  |  | CarP1198: | catD |
|  |  | CarP4522: | hypothetical protein |
|  |  | CarP7465: | rutC |
|  |  | CarP6842: | hypothetical protein |
|  |  | CarP3097: | tag |
|  |  | CarP4702: | hypothetical protein |
|  |  | CarP3878: | nagA |
|  |  | CarP1623: | hypothetical protein |
|  |  | CarP4277: | Chromate resistance exported protein |
|  |  | CarP7150: | Putative multidrug export ATP-binding perm |
|  |  | CarP7139: | hypothetical protein |
|  |  | CarP6991: | Acyltransferase family protein |
|  |  | CarP5117: | yciC |
|  |  | CarP4361: | Alpha-L-fucosidase |
|  |  | CarP3723: | zraS |
|  |  | CarP2082: | gcvH |
|  |  | CarP2643: | nuoB |
|  |  | CarP4917: | Glyoxalase-like domain protein |
|  |  | CarP5286: | hypothetical protein |
|  |  | CarP3580: | ravA |
|  |  | CarP4821: | hypothetical protein |
|  |  | CarP5087: | mtnK |
|  |  | CarP2253: | putative ABC transporter ATP-binding protein |
|  |  | CarP3207: | lytR |
|  |  | CarP7132: | SusD family protein |
|  |  | CarP1625: | Helix-turn-helix domain protein |
|  |  | CarP1199: | hypothetical protein |
|  |  | CarP4464: | hypothetical protein |
|  |  | CarP6144: | hypothetical protein |
|  |  | CarP1936: | hypothetical protein |
|  |  | CarP2932: | mfpsA |
|  |  | CarP3675: | hypothetical protein |
|  |  | CarP7674: | hypothetical protein |
|  |  | CarP6284: | idhA |
|  |  | CarP3264: | idhA |
|  |  | CarP3917: | tetratricopeptide repeat protein |
|  |  | CarP7495: | fumD |
|  |  | CarP7121: | Trehalose utilisation |
|  |  | CarP6573: | bcsA |
|  |  | CarP2792: | hypothetical protein |
|  |  | CarP3822: | rnhA |
|  |  | CarP4570: | Helix-turn-helix domain protein |
|  |  | CarP4025: | hypothetical protein |
|  |  | CarP7261: | hypothetical protein |
|  |  | CarP5620: | abf2 |
|  |  | CarP5342: | Arylesterase |
|  |  | CarP2099: | ribE |
|  |  | CarP6843: | fieF |
|  |  | CarP7537: | Bifunctional transcriptional activator DNA |
|  |  | CarP7625: | hypothetical protein |
|  |  | CarP4874: | ohrR |
|  |  | CarP7461: | hypothetical protein |
|  |  | CarP6530: | hypothetical protein |
|  |  | CarP5130: | hypothetical protein |
|  |  | CarP1836: | dapD |
|  |  | CarP4352: | hypothetical protein |
|  |  | CarP2243: | putative CtpA-like serine protease |
|  |  | CarP2262: | oprF |
|  |  | CarP4725: | ccpA |
|  |  | CarP6100: | hypothetical protein |
|  |  | CarP4009: | glcK |
|  |  | CarP3517: | nuoK |
|  |  | CarP6977: | Polysaccharide biosynthesis protein |
|  |  | CarP6257: | Amidohydrolase |
|  |  | CarP2355: | hisA |
|  |  | CarP6021: | hypothetical protein |
|  |  | CarP7624: | hypothetical protein |
|  |  | CarP4252: | hypothetical protein |
|  |  | CarP4408: | acs |
|  |  | CarP5209: | anaerobic benzoate catabolism transcriptional regulator |
|  |  | CarP2530: | fdhL |
|  |  | CarP6113: | Fibronectin type III domain protein |
|  |  | CarP5891: | Cyclic nucleotide-binding domain protein |
|  |  | CarP2270: | surA |
|  |  | CarP4459: | Macrolide export ATP-binding permease prot |
|  |  | CarP6562: | hypothetical protein |
|  |  | CarP1872: | tagO |
|  |  | CarP7459: | hypothetical protein |
|  |  | CarP2056: | Membrane dipeptidase (Peptidase family M19 |
|  |  | CarP3010: | epsH |
|  |  | CarP4586: | bamB |
|  |  | CarP3865: | yniC |
|  |  | CarP5558: | hlyD |
|  |  | CarP6404: | hypothetical protein |
|  |  | CarP7731: | hypothetical protein |
|  |  | CarP4509: | Amidohydrolase |
|  |  | CarP7058: | ydfG |
|  |  | CarP5448: | hypothetical protein |
|  |  | CarP3069: | yheI |
|  |  | CarP2574: | xerD |
|  |  | CarP7549: | hypothetical protein |
|  |  | CarP1094: | xerC |
|  |  | CarP1810: | amiA |
|  |  | CarP3004: | phoR |
|  |  | CarP3168: | hypothetical protein |
|  |  | CarP4051: | Putative anti-sigma factor antagonist |
|  |  | CarP7473: | cph1 |
|  |  | CarP2362: | murQ |
|  |  | CarP7175: | hypothetical protein |
|  |  | CarP1947: | fhlA |
|  |  | CarP3936: | Xylose isomerase-like TIM barrel |
|  |  | CarP4834: | MORN repeat variant |
|  |  | CarP2648: | EamA-like transporter family protein |
|  |  | CarP6597: | hypothetical protein |
|  |  | CarP3270: | Endoglucanase C307 precursor |
|  |  | CarP7212: | Surface antigen |
|  |  | CarP6365: | Glycosyl Hydrolase Family 88 |
|  |  | CarP7381: | hypothetical protein |
|  |  | CarP1749: | yfbT |
|  |  | CarP4410: | hypothetical protein |
|  |  | CarP7347: | hypothetical protein |
|  |  | CarP5084: | hypothetical protein |
|  |  | CarP2870: | kduD |
|  |  | CarP2196: | yitD |
|  |  | CarP3898: | hypothetical protein |
|  |  | CarP2772: | Immunogenic protein MPT70 precursor |
|  |  | CarP3983: | HPP family protein |
|  |  | CarP2416: | estB |
|  |  | CarP2185: | panC |
|  |  | CarP2991: | wfgD |
|  |  | CarP3489: | xylB |
|  |  | CarP7365: | SnoaL-like domain protein |
|  |  | CarP2809: | arcB |
|  |  | CarP2387: | arnC |
|  |  | CarP4138: | pspE |
|  |  | CarP4315: | hypothetical protein |
|  |  | CarP5005: | Na(+) H(+) antiporter subunit D |
|  |  | CarP7586: | hypothetical protein |
|  |  | CarP5658: | TM2 domain protein |
|  |  | CarP1743: | metK |
|  |  | CarP4637: | hypothetical protein |
|  |  | CarP2456: | GSCFA family protein |
|  |  | CarP4955: | hypothetical protein |
|  |  | CarP2405: | pchA |
|  |  | CarP4538: | sspH2 |
|  |  | CarP4149: | hypothetical protein |
|  |  | CarP3469: | Calcineurin-like phosphoesterase |
|  |  | CarP3881: | manA |
|  |  | CarP5429: | pcpB |
|  |  | CarP5630: | hypothetical protein |
|  |  | CarP6768: | fsr |
|  |  | CarP5337: | hypothetical protein |
|  |  | CarP2307: | purN |
|  |  | CarP5439: | cbgA |
|  |  | CarP7692: | trpF |
|  |  | CarP6646: | aceB |
|  |  | CarP5341: | yhdN |
|  |  | CarP5795: | Helix-turn-helix domain protein |
|  |  | CarP4871: | Bifunctional transcriptional activator DNA |
|  |  | CarP4996: | hutG |
|  |  | CarP2887: | Deoxyadenosine deoxycytidine kinase |
|  |  | CarP6542: | hypothetical protein |
|  |  | CarP2706: | corC |
|  |  | CarP6614: | ccp |
|  |  | CarP5825: | hypothetical protein |
|  |  | CarP3326: | xynZ |
|  |  | CarP4340: | hypothetical protein |
|  |  | CarP3781: | Transposase IS200 like protein |
|  |  | CarP2308: | prenyltransferase |
|  |  | CarP4447: | hypothetical protein |
|  |  | CarP2388: | epsH |
|  |  | CarP6517: | aroF |
|  |  | CarP5097: | DinB superfamily protein |
|  |  | CarP3557: | hsdS |
|  |  | CarP2699: | yycF |
|  |  | CarP3876: | todS |
|  |  | CarP2031: | mdeA |
|  |  | CarP6280: | nlhH |
|  |  | CarP5782: | hypothetical protein |
|  |  | CarP1027: | Transposase DDE domain protein |
|  |  | CarP3801: | hypothetical protein |
|  |  | CarP6761: | hypothetical protein |
|  |  | CarP5754: | tuaC |
|  |  | CarP2004: | Putative F0F1-ATPase subunit (ATPase gene1 |
|  |  | CarP5273: | htrB |
|  |  | CarP2393: | pabA |
|  |  | CarP4609: | thiol-disulfide oxidoreductase |
|  |  | CarP2239: | panB |
|  |  | CarP5171: | hypothetical protein |
|  |  | CarP2714: | soxS |
|  |  | CarP5296: | hypothetical protein |
|  |  | CarP2563: | Auracyanin-A precursor |
|  |  | CarP4341: | blaI |
|  |  | CarP2554: | cusR |
|  |  | CarP3103: | Polysaccharide deacetylase |
|  |  | CarP6399: | hypothetical protein |
|  |  | CarP4005: | ndoA |
|  |  | CarP6899: | hypothetical protein |
|  |  | CarP3045: | hypothetical protein |
|  |  | CarP7094: | phyH |
|  |  | CarP5335: | von Willebrand factor |
|  |  | CarP2150: | gcvP |
|  |  | CarP7046: | hypothetical protein |
|  |  | CarP3471: | hypothetical protein |
|  |  | CarP6401: | Trehalose utilisation |
|  |  | CarP3938: | ispH |
|  |  | CarP2457: | Methyltransferase domain protein |
|  |  | CarP6973: | hypothetical protein |
|  |  | CarP6716: | D-tagatose 3-epimerase |
|  |  | CarP6960: | guaA |
|  |  | CarP2675: | idhA |
|  |  | CarP3893: | Adenosine monophosphate-protein transferase SoFic |
|  |  | CarP4515: | mshA |
|  |  | CarP2017: | hypothetical protein |
|  |  | CarP1896: | hypothetical protein |
|  |  | CarP3196: | salL |
|  |  | CarP4456: | exsA |
|  |  | CarP6368: | hypothetical protein |
|  |  | CarP2800: | recD2 |
|  |  | CarP5442: | hypothetical protein |
|  |  | CarP2289: | ytrA |
|  |  | CarP5971: | hypothetical protein |
|  |  | CarP1682: | uspE |
|  |  | CarP1641: | hypothetical protein |
|  |  | CarP5060: | hypothetical protein |
|  |  | CarP7291: | lutR |
|  |  | CarP2414: | tolC |
|  |  | CarP5808: | hypothetical protein |
|  |  | CarP4577: | hypothetical protein |
|  |  | CarP2567: | hypothetical protein |
|  |  | CarP5864: | hypothetical protein |
|  |  | CarP5057: | hypothetical protein |
|  |  | CarP4429: | mqo |
|  |  | CarP4980: | hypothetical protein |
|  |  | CarP5653: | wbbD |
|  |  | CarP6024: | hypothetical protein |
|  |  | CarP5884: | ydjP |
|  |  | CarP2497: | hypothetical protein |
|  |  | CarP3432: | moeA |
|  |  | CarP6479: | hypothetical protein |
|  |  | CarP2936: | alx |
|  |  | CarP5314: | Nitronate monooxygenase |
|  |  | CarP4537: | hypothetical protein |
|  |  | CarP1756: | hypothetical protein |
|  |  | CarP7258: | tuaC |
|  |  | CarP3906: | hypothetical protein |
|  |  | CarP2938: | ppx |
|  |  | CarP3338: | hypothetical protein |
|  |  | CarP2328: | rshA |
|  |  | CarP3637: | Sodium glucose cotransporter |
|  |  | CarP7733: | azoB |
|  |  | CarP7391: | exo I |
|  |  | CarP3053: | anaerobic benzoate catabolism transcriptional regulator |
|  |  | CarP5172: | Putative O-methyltransferase MSMEI 4947 |
|  |  | CarP6273: | hypothetical protein |
|  |  | CarP6034: | hypothetical protein |
|  |  | CarP1787: | hypothetical protein |
|  |  | CarP2412: | glnA |
|  |  | CarP2160: | ABC-2 family transporter protein |
|  |  | CarP1668: | pepN |
|  |  | CarP4434: | Carbohydrate acetyl esterase feruloyl este |
|  |  | CarP6300: | cysH |
|  |  | CarP6966: | hypothetical protein |
|  |  | CarP7091: | cphB |
|  |  | CarP7567: | hypothetical protein |
|  |  | CarP5857: | hypothetical protein |
|  |  | CarP5594: | Endo-1,4-beta-xylanase feruloyl esterase p |
|  |  | CarP2992: | epsH |
|  |  | CarP4470: | putative ABC transporter ATP-binding protein |
|  |  | CarP3032: | pyrC |
|  |  | CarP6244: | Inositol 2-dehydrogenase D-chiro-inositol |
|  |  | CarP7581: | galB |
|  |  | CarP4963: | hypothetical protein |
|  |  | CarP2258: | Murein DD-endopeptidase MepS Murein LD-car |
|  |  | CarP5600: | liaS |
|  |  | CarP6370: | Vancomycin teicoplanin A-type resistance p |
|  |  | CarP4839: | ethD |
|  |  | CarP7290: | Nucleotidyltransferase domain protein |
|  |  | CarP6774: | hypothetical protein |
|  |  | CarP2490: | ksgA |
|  |  | CarP5295: | lysDH |
|  |  | CarP5817: | hypothetical protein |
|  |  | CarP7023: | SusD family protein |
|  |  | CarP2555: | Outer membrane efflux protein |
|  |  | CarP3160: | PhoPQ-activated pathogenicity-related prot |
|  |  | CarP7405: | bkdC |
|  |  | CarP4244: | hypothetical protein |
|  |  | CarP5400: | hypothetical protein |
|  |  | CarP3840: | tolC |
|  |  | CarP2593: | xylA |
|  |  | CarP1216: | hypothetical protein |
|  |  | CarP7274: | outer membrane channel protein |
|  |  | CarP4700: | hypothetical protein |
|  |  | CarP1965: | katG |
|  |  | CarP2785: | dapL |
|  |  | CarP3753: | hypothetical protein |
|  |  | CarP7329: | hypothetical protein |
|  |  | CarP5868: | hypothetical protein |
|  |  | CarP2214: | putative enoyl-CoA hydratase 1 |
|  |  | CarP4387: | PA14 domain protein |
|  |  | CarP4483: | hypothetical protein |
|  |  | CarP6242: | gdhI |
|  |  | CarP6611: | adhD |
|  |  | CarP7034: | hypothetical protein |
|  |  | CarP6865: | apt |
|  |  | CarP5648: | hypothetical protein |
|  |  | CarP5330: | LVIVD repeat protein |
|  |  | CarP5239: | Fatty acid desaturase |
|  |  | CarP5042: | todS |
|  |  | CarP4629: | hypothetical protein |
|  |  | CarP5220: | hypothetical protein |
|  |  | CarP6058: | hypothetical protein |
|  |  | CarP2189: | putative 5-formyltetrahydrofolate cyclo-ligase |
|  |  | CarP4409: | hypothetical protein |
|  |  | CarP7732: | quiP |
|  |  | CarP6665: | hypothetical protein |
|  |  | CarP5577: | Oxidoreductase molybdopterin binding domai |
|  |  | CarP2447: | rpe |
|  |  | CarP2332: | hypothetical protein |
|  |  | CarP4316: | hypothetical protein |
|  |  | CarP5856: | hypothetical protein |
|  |  | CarP2314: | CcmB protein |
|  |  | CarP4090: | resA |
|  |  | CarP4488: | hypothetical protein |
|  |  | CarP1788: | ftsX |
|  |  | CarP6811: | Serine threonine-protein kinase RsbT |
|  |  | CarP6443: | Phosphate-selective porin O and P |
|  |  | CarP3378: | araA |
|  |  | CarP1842: | hypothetical protein |
|  |  | CarP3486: | fecR |
|  |  | CarP5010: | Deoxyribodipyrimidine photo-lyase-related |
|  |  | CarP3704: | cheB |
|  |  | CarP4841: | hypothetical protein |
|  |  | CarP7596: | oprF |
|  |  | CarP7078: | Xylose isomerase-like TIM barrel |
|  |  | CarP4283: | MG2 domain protein |
|  |  | CarP7418: | doc |
|  |  | CarP7752: | HEPN domain protein |
|  |  | CarP6837: | Patatin-like phospholipase |
|  |  | CarP3470: | bcp |
|  |  | CarP4533: | gap |
|  |  | CarP6593: | hypothetical protein |
|  |  | CarP7325: | hypothetical protein |
|  |  | CarP6295: | hypothetical protein |
|  |  | CarP2868: | putative acyl-CoA thioester hydrolase |
|  |  | CarP5842: | fucP |
|  |  | CarP2575: | hypothetical protein |
|  |  | CarP2812: | hypothetical protein |
|  |  | CarP1644: | ctpB |
|  |  | CarP5454: | hypothetical protein |
|  |  | CarP7705: | hypothetical protein |
|  |  | CarP2743: | yehT |
|  |  | CarP2692: | hypothetical protein |
|  |  | CarP5645: | Nitrilase |
|  |  | CarP3598: | chbR |
|  |  | CarP6566: | fecR |
|  |  | CarP6336: | hypothetical protein |
|  |  | CarP4895: | Cupin domain protein |
|  |  | CarP7697: | PglZ domain protein |
|  |  | CarP6705: | hypothetical protein |
|  |  | CarP5500: | lptB |
|  |  | CarP2143: | pdxA |
|  |  | CarP3746: | resA |
|  |  | CarP6987: | hypothetical protein |
|  |  | CarP3828: | hypothetical protein |
|  |  | CarP3875: | hypothetical protein |
|  |  | CarP6898: | hypothetical protein |
|  |  | CarP2661: | yknZ |
|  |  | CarP5048: | hypothetical protein |
|  |  | CarP6296: | hypothetical protein |
|  |  | CarP2851: | hypothetical protein |
|  |  | CarP4368: | DinB superfamily protein |
|  |  | CarP5549: | hypothetical protein |
|  |  | CarP6308: | hypothetical protein |
|  |  | CarP6650: | mdh |
|  |  | CarP6470: | scdA |
|  |  | CarP5287: | DSBA-like thioredoxin domain protein |
|  |  | CarP2368: | purF |
|  |  | CarP4333: | ttgR |
|  |  | CarP5599: | hypothetical protein |
|  |  | CarP2553: | pstA |
|  |  | CarP4234: | hypothetical protein |
|  |  | CarP2191: | ispF |
|  |  | CarP3947: | Xylose isomerase-like TIM barrel |
|  |  | CarP5228: | putative acetyltransferase |
|  |  | CarP3310: | Glycosyl hydrolases family 2, sugar bindin |
|  |  | CarP5215: | manA |
|  |  | CarP2468: | hisD |
|  |  | CarP6381: | Surface antigen |
|  |  | CarP5905: | chbR |
|  |  | CarP1215: | hypothetical protein |
|  |  | CarP4510: | hypothetical protein |
|  |  | CarP7225: | Bacterial transcription activator, effecto |
|  |  | CarP1999: | pgdA |
|  |  | CarP3595: | hypothetical protein |
|  |  | CarP3994: | hypothetical protein |
|  |  | CarP5588: | hypothetical protein |
|  |  | CarP5609: | Glycosyl transferases group 1 |
|  |  | CarP3187: | hypothetical protein |
|  |  | CarP4813: | hypothetical protein |
|  |  | CarP2923: | NAD NADP-dependent betaine aldehyde dehydr |
|  |  | CarP4229: | Hydroxymethylpyrimidine phosphomethylpyrim |
|  |  | CarP3104: | NUDIX domain protein |
|  |  | CarP2210: | guaB |
|  |  | CarP1985: | hypothetical protein |
|  |  | CarP3485: | dagK |
|  |  | CarP6354: | ispH |
|  |  | CarP7234: | trxA |
|  |  | CarP1121: | fnr |
|  |  | CarP4117: | atsA2 |
|  |  | CarP7070: | hypothetical protein |
|  |  | CarP7714: | def |
|  |  | CarP3139: | Erythromycin esterase |
|  |  | CarP2798: | kdsD |
|  |  | CarP5834: | hypothetical protein |
|  |  | CarP5819: | atsA |
|  |  | CarP4377: | Carboxynorspermidine synthase |
|  |  | CarP4912: | HD domain protein |
|  |  | CarP3213: | csbC |
|  |  | CarP3968: | Xylosidase arabinosidase |
|  |  | CarP4453: | NmrA-like family protein |
|  |  | CarP2710: | hypothetical protein |
|  |  | CarP6897: | Bifunctional (p)ppGpp synthase hydrolase R |
|  |  | CarP5469: | hypothetical protein |
|  |  | CarP3845: | gapN |
|  |  | CarP6428: | hypothetical protein |
|  |  | CarP7583: | Bifunctional transcriptional activator DNA |
|  |  | CarP6320: | putative acetyltransferase |
|  |  | CarP2970: | glucans biosynthesis protein |
|  |  | CarP4125: | Glycosyl transferases group 1 |
|  |  | CarP4366: | amy |
|  |  | CarP4373: | hypothetical protein |
|  |  | CarP4420: | dadA |
|  |  | CarP5932: | hypothetical protein |
|  |  | CarP4308: | LysE type translocator |
|  |  | CarP6287: | Malate-2H(+) Na(+)-lactate antiporter |
|  |  | CarP5427: | RNA polymerase sigma factor |
|  |  | CarP6641: | hypothetical protein |
|  |  | CarP5180: | cycA |
|  |  | CarP2595: | Susd and RagB outer membrane lipoprotein |
|  |  | CarP7253: | hypothetical protein |
|  |  | CarP7252: | xylI |
|  |  | CarP5416: | hypothetical protein |
|  |  | CarP4123: | epsE |
|  |  | CarP4154: | pdxH |
|  |  | CarP7589: | hypothetical protein |
|  |  | CarP7237: | cbgA |
|  |  | CarP5605: | hypothetical protein |
|  |  | CarP5984: | hypothetical protein |
|  |  | CarP4827: | hypothetical protein |
|  |  | CarP7556: | lipopolysaccharide core biosynthesis prote |
|  |  | CarP5433: | hypothetical protein |
|  |  | CarP7661: | Ultraviolet N-glycosylase AP lyase |
|  |  | CarP5837: | hypothetical protein |
|  |  | CarP4824: | Adenosine monophosphate-protein transferase SoFic |
|  |  | CarP5340: | Lactonase, 7-bladed beta-propeller |
|  |  | CarP7641: | cefD |
|  |  | CarP4245: | Core-2 I-Branching enzyme |
|  |  | CarP1184: | hypothetical protein |
|  |  | CarP5063: | atsA |
|  |  | CarP2882: | lhgO |
|  |  | CarP6728: | gmuF |
|  |  | CarP6722: | hypothetical protein |
|  |  | CarP7742: | hypothetical protein |
|  |  | CarP2133: | rluA |
|  |  | CarP4164: | hypothetical protein |
|  |  | CarP4679: | Na(+) H(+) antiporter NhaD |
|  |  | CarP4080: | pabB |
|  |  | CarP4945: | hypothetical protein |
|  |  | CarP7019: | hypBA1 |
|  |  | CarP2394: | trpD2 |
|  |  | CarP7103: | L-fuconate dehydratase |
|  |  | CarP7627: | RNA polymerase sigma factor |
|  |  | CarP2717: | hypothetical protein |
|  |  | CarP3639: | Fibronectin type III domain protein |
|  |  | CarP1521: | bepE |
|  |  | CarP6095: | hypothetical protein |
|  |  | CarP5769: | SusD family protein |
|  |  | CarP1937: | hypothetical protein |
|  |  | CarP2093: | pfkA |
|  |  | CarP3593: | resA |
|  |  | CarP7380: | Alpha-L-fucosidase |
|  |  | CarP6469: | Glyoxalase-like domain protein |
|  |  | CarP3512: | sigW |
|  |  | CarP7524: | Zinc carboxypeptidase |
|  |  | CarP5038: | SusD family protein |
|  |  | CarP7036: | hypothetical protein |
|  |  | CarP2486: | hypothetical protein |
|  |  | CarP1980: | Macrolide export ATP-binding permease prot |
|  |  | CarP3304: | metE |
|  |  | CarP4401: | hypothetical protein |
|  |  | CarP5657: | hypothetical protein |
|  |  | CarP5043: | fumarate nitrate reduction transcriptional |
|  |  | CarP2396: | trpF |
|  |  | CarP4644: | Xylose isomerase-like TIM barrel |
|  |  | CarP3673: | ydbD |
|  |  | CarP4057: | scpA |
|  |  | CarP3749: | nicC |
|  |  | CarP6615: | can |
|  |  | CarP4814: | hypothetical protein |
|  |  | CarP5614: | hypothetical protein |
|  |  | CarP4534: | sigW |
|  |  | CarP2496: | yccM |
|  |  | CarP2672: | tdk |
|  |  | CarP4633: | acrC |
|  |  | CarP5480: | rbsC |
|  |  | CarP2162: | hypothetical protein |
|  |  | CarP7721: | hypothetical protein |
|  |  | CarP4937: | udp |
|  |  | CarP1731: | hypothetical protein |
|  |  | CarP5036: | Helix-turn-helix |
|  |  | CarP4840: | parE1 |
|  |  | CarP2649: | hypothetical protein |
|  |  | CarP6011: | HEPN domain protein |
|  |  | CarP3342: | mscM |
|  |  | CarP2998: | Calcineurin-like phosphoesterase |
|  |  | CarP3021: | Transposase |
|  |  | CarP7077: | hypothetical protein |
|  |  | CarP2871: | btr |
|  |  | CarP5934: | hypothetical protein |
|  |  | CarP5529: | Zeta toxin |
|  |  | CarP1528: | atoS |
|  |  | CarP7660: | cph1 |
|  |  | CarP6701: | hypothetical protein |
|  |  | CarP5091: | fadE |
|  |  | CarP5831: | 37-kD nucleoid-associated bacterial protei |
|  |  | CarP2090: | rluA |
|  |  | CarP4033: | hypothetical protein |
|  |  | CarP2244: | hypothetical protein |
|  |  | CarP7399: | Glycosyl hydrolases family 43 |
|  |  | CarP6616: | Glycosyl hydrolases family 43 |
|  |  | CarP3325: | FG-GAP repeat protein |
|  |  | CarP3582: | hmgA |
|  |  | CarP6703: | hypothetical protein |
|  |  | CarP3547: | hypothetical protein |
|  |  | CarP4203: | cytoplasmic glycerophosphodiester phosphod |
|  |  | CarP6473: | ypdB |
|  |  | CarP2852: | hypothetical protein |
|  |  | CarP3450: | hypothetical protein |
|  |  | CarP4599: | hypothetical protein |
|  |  | CarP6160: | xylE |
|  |  | CarP7027: | cph1 |
|  |  | CarP5310: | yjbI |
|  |  | CarP2732: | oprM |
|  |  | CarP3379: | hypothetical protein |
|  |  | CarP4690: | MarR family protein |
|  |  | CarP5757: | mdtB |
|  |  | CarP7163: | hypothetical protein |
|  |  | CarP5446: | hypothetical protein |
|  |  | CarP6201: | Daunorubicin doxorubicin resistance ATP-bi |
|  |  | CarP5356: | miaA |
|  |  | CarP2907: | scoA |
|  |  | CarP3370: | hsdR |
|  |  | CarP6380: | hypothetical protein |
|  |  | CarP4610: | cytR |
|  |  | CarP6230: | Bacterial regulatory proteins, tetR family |
|  |  | CarP3364: | cusR |
|  |  | CarP3717: | hypothetical protein |
|  |  | CarP6776: | hypothetical protein |
|  |  | CarP7285: | zwf |
|  |  | CarP2684: | fcl |
|  |  | CarP6074: | NADH dehydrogenase subunit E |
|  |  | CarP2409: | hypothetical protein |
|  |  | CarP2446: | putative ABC transporter ATP-binding protein |
|  |  | CarP5765: | hypothetical protein |
|  |  | CarP1838: | yxeP |
|  |  | CarP3437: | hypothetical protein |
|  |  | CarP6594: | thiol-disulfide oxidoreductase |
|  |  | CarP6578: | iolE |
|  |  | CarP3336: | nrgA |
|  |  | CarP2363: | hypothetical protein |
|  |  | CarP3492: | hypothetical protein |
|  |  | CarP6847: | hypothetical protein |
|  |  | CarP1529: | yicI |
|  |  | CarP3548: | Esterase PHB depolymerase |
|  |  | CarP2207: | Phosphoribosyl-ATP pyrophosphohydrolase |
|  |  | CarP5049: | hypothetical protein |
|  |  | CarP6279: | Stearoyl-CoA 9-desaturase electron transfe |
|  |  | CarP3869: | hypothetical protein |
|  |  | CarP4382: | Peptidase family M23 |
|  |  | CarP2691: | alaA |
|  |  | CarP2112: | rny |
|  |  | CarP3226: | Bestrophin, RFP-TM, chloride channel |
|  |  | CarP7584: | hypothetical protein |
|  |  | CarP5145: | amyB |
|  |  | CarP3284: | lutC |
|  |  | CarP6461: | yvoA |
|  |  | CarP3316: | hypothetical protein |
|  |  | CarP5245: | hypothetical protein |
|  |  | CarP2302: | coaBC |
|  |  | CarP1748: | hypothetical protein |
|  |  | CarP1711: | galE |
|  |  | CarP3958: | Planctomycete cytochrome C |
|  |  | CarP6519: | tmoS |
|  |  | CarP5031: | betI |
|  |  | CarP5363: | Cytochrome c |
|  |  | CarP5686: | hypothetical protein |
|  |  | CarP4124: | pglH |
|  |  | CarP1017: | fdhL |
|  |  | CarP3700: | hypothetical protein |
|  |  | CarP4266: | kdpD |
|  |  | CarP6010: | hypothetical protein |
|  |  | CarP2121: | phoP |
|  |  | CarP1115: | cph1 |
|  |  | CarP4845: | mscK |
|  |  | CarP7454: | hypothetical protein |
|  |  | CarP7429: | Transglutaminase-like superfamily protein |
|  |  | CarP6090: | hypothetical protein |
|  |  | CarP6875: | Polysaccharide biosynthesis protein |
|  |  | CarP7146: | Acyltransferase family protein |
|  |  | CarP6012: | hypothetical protein |
|  |  | CarP2014: | hypothetical protein |
|  |  | CarP7006: | zraS |
|  |  | CarP4327: | enterobactin ferric enterobactin esterase |
|  |  | CarP5892: | hypothetical protein |
|  |  | CarP4172: | hypothetical protein |
|  |  | CarP1904: | Pregnancy-associated plasma protein-A |
|  |  | CarP6883: | hypothetical protein |
|  |  | CarP5511: | hypothetical protein |
|  |  | CarP4441: | hypothetical protein |
|  |  | CarP5812: | hypothetical protein |
|  |  | CarP6087: | hypothetical protein |
|  |  | CarP5368: | hypothetical protein |
|  |  | CarP3136: | higB-2 |
|  |  | CarP1995: | hypothetical protein |
|  |  | CarP2632: | tetratricopeptide repeat protein |
|  |  | CarP3861: | hypothetical protein |
|  |  | CarP3678: | sigW |
|  |  | CarP2854: | Peptidase M16 inactive domain protein |
|  |  | CarP7408: | hypothetical protein |
|  |  | CarP4376: | hypothetical protein |
|  |  | CarP7014: | hypothetical protein |
|  |  | CarP3962: | copR |
|  |  | CarP7179: | hypothetical protein |
|  |  | CarP3090: | hypothetical protein |
|  |  | CarP1763: | Putative multidrug export ATP-binding permease |
|  |  | CarP3182: | cutC |
|  |  | CarP6830: | WD40-like Beta Propeller Repeat protein |
|  |  | CarP1840: | divIVA |
|  |  | CarP2788: | hypothetical protein |
|  |  | CarP6988: | Glycosyl transferase family 2 |
|  |  | CarP6196: | Sodium glucose cotransporter |
|  |  | CarP2813: | mepM |
|  |  | CarP7607: | fadD |
|  |  | CarP2250: | aroC |
|  |  | CarP6634: | sdcS |
|  |  | CarP5189: | VIT family protein |
|  |  | CarP1742: | fadE |
|  |  | CarP6036: | hypothetical protein |
|  |  | CarP1634: | Outer membrane lipoprotein Omp16 precursor |
|  |  | CarP6067: | hypothetical protein |
|  |  | CarP4952: | hypothetical protein |
|  |  | CarP2739: | hypothetical protein |
|  |  | CarP3212: | hypothetical protein |
|  |  | CarP4128: | degU |
|  |  | CarP4648: | hypothetical protein |
|  |  | CarP3060: | hypothetical protein |
|  |  | CarP1753: | arlS |
|  |  | CarP3760: | besA |
|  |  | CarP5894: | exsA |
|  |  | CarP2588: | Nucleotidyltransferase domain protein |
|  |  | CarP3476: | hypothetical protein |
|  |  | CarP6071: | xerC |
|  |  | CarP5674: | hypothetical protein |
|  |  | CarP2912: | bepC |
|  |  | CarP6494: | hypothetical protein |
|  |  | CarP3177: | hypothetical protein |
|  |  | CarP6375: | idhA |
|  |  | CarP2249: | nfuA |
|  |  | CarP2584: | hypothetical protein |
|  |  | CarP7687: | nucA |
|  |  | CarP2579: | hypothetical protein |
|  |  | CarP4935: | hypothetical protein |
|  |  | CarP7067: | SusD family protein |
|  |  | CarP2440: | hypothetical protein |
|  |  | CarP5495: | hypothetical protein |
|  |  | CarP1786: | ysdC |
|  |  | CarP4265: | kdpC |
|  |  | CarP6299: | patA |
|  |  | CarP5247: | hypothetical protein |
|  |  | CarP4567: | hypothetical protein |
|  |  | CarP5293: | FtsX-like permease family protein |
|  |  | CarP1196: | iorB |
|  |  | CarP1974: | Immunogenic protein MPT70 precursor |
|  |  | CarP7676: | recombination protein F |
|  |  | CarP5463: | hypothetical protein |
|  |  | CarP3328: | yoaB |
|  |  | CarP7412: | 4-O-beta-D-mannosyl-D-glucose phosphorylas |
|  |  | CarP6539: | hypothetical protein |
|  |  | CarP1952: | hypothetical protein |
|  |  | CarP5506: | hypothetical protein |
|  |  | CarP2286: | ispG |
|  |  | CarP2910: | Putative 5'(3')-deoxyribonucleotidase |
|  |  | CarP6146: | Archaeal ATPase |
|  |  | CarP3117: | EamA-like transporter family protein |
|  |  | CarP4687: | hypothetical protein |
|  |  | CarP6961: | hypothetical protein |
|  |  | CarP4359: | hypothetical protein |
|  |  | CarP6916: | pseC |
|  |  | CarP2840: | hypothetical protein |
|  |  | CarP2449: | fgs |
|  |  | CarP7214: | hypothetical protein |
|  |  | CarP2833: | mdtC |
|  |  | CarP2728: | pbpG |
|  |  | CarP4093: | hypothetical protein |
|  |  | CarP6627: | Putative phospholipase A1 precursor |
|  |  | CarP4221: | Chain length determinant protein |
|  |  | CarP7453: | hypothetical protein |
|  |  | CarP6832: | hypothetical protein |
|  |  | CarP7654: | Bacterial regulatory proteins, luxR family |
|  |  | CarP3569: | hypothetical protein |
|  |  | CarP3600: | nadA |
|  |  | CarP4592: | hypothetical protein |
|  |  | CarP5640: | hypothetical protein |
|  |  | CarP4904: | scpA |
|  |  | CarP4858: | META domain protein |
|  |  | CarP6068: | Serine threonine-protein kinase StkP |
|  |  | CarP1866: | Sodium glucose cotransporter |
|  |  | CarP3249: | HD domain protein |
|  |  | CarP2432: | LemA family protein |
|  |  | CarP3568: | Trehalose synthase amylase TreS |
|  |  | CarP2888: | hypothetical protein |
|  |  | CarP5895: | acr1 |
|  |  | CarP3024: | Gram-negative bacterial tonB protein |
|  |  | CarP7279: | fas3 |
|  |  | CarP1204: | uvrY |
|  |  | CarP5659: | hypothetical protein |
|  |  | CarP5986: | hypothetical protein |
|  |  | CarP1612: | yedK |
|  |  | CarP4549: | hypothetical protein |
|  |  | CarP3298: | Zinc carboxypeptidase |
|  |  | CarP6890: | hypothetical protein |
|  |  | CarP3417: | L-fucose mutarotase |
|  |  | CarP2713: | hypothetical protein |
|  |  | CarP1058: | Transposase DDE domain protein |
|  |  | CarP1972: | hypothetical protein |
|  |  | CarP7450: | Acyltransferase family protein |
|  |  | CarP6290: | lptB |
|  |  | CarP7394: | TrwC relaxase |
|  |  | CarP1830: | BadF BadG BcrA BcrD ATPase family protein |
|  |  | CarP1816: | tsaC |
|  |  | CarP3411: | SusD family protein |
|  |  | CarP3449: | glnA |
|  |  | CarP5319: | ahpD |
|  |  | CarP7335: | Ribonuclease VapC19 |
|  |  | CarP2951: | hypothetical protein |
|  |  | CarP7266: | tetratricopeptide repeat protein |
|  |  | CarP3001: | rspA |
|  |  | CarP6151: | hypothetical protein |
|  |  | CarP6695: | atsA |
|  |  | CarP3695: | fecR |
|  |  | CarP3999: | hypothetical protein |
|  |  | CarP4305: | hypothetical protein |
|  |  | CarP6329: | hypothetical protein |
|  |  | CarP4557: | ppk |
|  |  | CarP6783: | DinB superfamily protein |
|  |  | CarP6400: | mrsA |
|  |  | CarP5385: | hypothetical protein |
|  |  | CarP5962: | hypothetical protein |
|  |  | CarP3250: | nreC |
|  |  | CarP1794: | asd |
|  |  | CarP2599: | uxaC |
|  |  | CarP2251: | hypothetical protein |
|  |  | CarP2048: | comB |
|  |  | CarP3277: | cph1 |
|  |  | CarP4431: | fabG |
|  |  | CarP2865: | N-carbamoyl-D-amino acid hydrolase |
|  |  | CarP7608: | hypothetical protein |
|  |  | CarP5183: | anaerobic benzoate catabolism transcriptional regulator |
|  |  | CarP3952: | SusD family protein |
|  |  | CarP3986: | Alpha beta hydrolase family protein |
|  |  | CarP2120: | Endonuclease Exonuclease phosphatase famil |
|  |  | CarP7373: | Transglycosylase associated protein |
|  |  | CarP6984: | fdhA |
|  |  | CarP5510: | fabB |
|  |  | CarP3802: | ysdC |
|  |  | CarP4929: | icd |
|  |  | CarP3866: | hypothetical protein |
|  |  | CarP7303: | hypothetical protein |
|  |  | CarP7247: | hypothetical protein |
|  |  | CarP7610: | lytR |
|  |  | CarP3146: | putative efflux pump membrane fusion prote |
|  |  | CarP1597: | rrrD |
|  |  | CarP7620: | XisI protein |
|  |  | CarP5517: | hypothetical protein |
|  |  | CarP4899: | putative adenylyltransferase sulfurtransfe |
|  |  | CarP5807: | Autoinducer 2 sensor kinase phosphatase Lu |
|  |  | CarP1808: | aarA |
|  |  | CarP4406: | clcA |
|  |  | CarP7722: | hypothetical protein |
|  |  | CarP6184: | nqrA |
|  |  | CarP6813: | rsbS |
|  |  | CarP1760: | hypothetical protein |
|  |  | CarP3460: | ftsH |
|  |  | CarP5106: | Polysaccharide deacetylase |
|  |  | CarP7600: | qorA |
|  |  | CarP6315: | ybaQ |
|  |  | CarP1820: | ispB |
|  |  | CarP3391: | hypothetical protein |
|  |  | CarP7557: | hypothetical protein |
|  |  | CarP2784: | hypothetical protein |
|  |  | CarP2904: | allB |
|  |  | CarP6787: | hypothetical protein |
|  |  | CarP1086: | hypothetical protein |
|  |  | CarP4011: | bioF |
|  |  | CarP5928: | dinB |
|  |  | CarP6896: | hypothetical protein |
|  |  | CarP5488: | hypothetical protein |
|  |  | CarP2600: | Bacterial alpha-L-rhamnosidase |
|  |  | CarP4430: | Putative niacin nicotinamide transporter N |
|  |  | CarP7448: | hypothetical protein |
|  |  | CarP7719: | ADP-ribosylglycohydrolase |
|  |  | CarP7051: | hypothetical protein |
|  |  | CarP5619: | hypothetical protein |
|  |  | CarP2109: | hypothetical protein |
|  |  | CarP6020: | hypothetical protein |
|  |  | CarP5080: | Autoinducer 2 sensor kinase phosphatase Lu |
|  |  | CarP2304: | srlR |
|  |  | CarP4064: | arnT |
|  |  | CarP2378: | hypothetical protein |
|  |  | CarP5902: | sigW |
|  |  | CarP3257: | hypothetical protein |
|  |  | CarP6906: | hypothetical protein |
|  |  | CarP1949: | SusD family protein |
|  |  | CarP4551: | hypothetical protein |
|  |  | CarP7682: | hypothetical protein |
|  |  | CarP4344: | xynC |
|  |  | CarP1698: | Planctomycete cytochrome C |
|  |  | CarP3002: | hypothetical protein |
|  |  | CarP4392: | hypothetical protein |
|  |  | CarP6927: | eutC |
|  |  | CarP6408: | Helix-turn-helix |
|  |  | CarP6679: | ftrA |
|  |  | CarP6289: | hypothetical protein |
|  |  | CarP4473: | arfA |
|  |  | CarP5809: | hypothetical protein |
|  |  | CarP1964: | zraR |
|  |  | CarP7644: | ATP GTP phosphatase |
|  |  | CarP5673: | hypothetical protein |
|  |  | CarP5241: | phoB |
|  |  | CarP5532: | hypothetical protein |
|  |  | CarP2372: | fur |
|  |  | CarP5542: | gpr |
|  |  | CarP2413: | phosphoribosylformylglycinamidine synthase II |
|  |  | CarP4167: | Glycosyl transferases group 1 |
|  |  | CarP6429: | rebM |
|  |  | CarP4096: | hypothetical protein |
|  |  | CarP6293: | xecA1 |
|  |  | CarP2636: | nuoH |
|  |  | CarP5563: | hypothetical protein |
|  |  | CarP2132: | pyrE |
|  |  | CarP2172: | nadC |
|  |  | CarP6962: | DSBA-like thioredoxin domain protein |
|  |  | CarP3098: | putative DMT superfamily transporter inner |
|  |  | CarP3221: | rpoE |
|  |  | CarP4694: | Universal stress protein family protein |
|  |  | CarP6430: | hypothetical protein |
|  |  | CarP4925: | hypothetical protein |
|  |  | CarP2325: | hemN |
|  |  | CarP1716: | hypothetical protein |
|  |  | CarP4695: | fdhA |
|  |  | CarP3406: | Patatin-like phospholipase |
|  |  | CarP4565: | Trehalose synthase amylase TreS |
|  |  | CarP2989: | TonB dependent receptor |
|  |  | CarP5464: | hypothetical protein |
|  |  | CarP1210: | Sodium glucose cotransporter |
|  |  | CarP5505: | epsE |
|  |  | CarP5559: | Helix-turn-helix domain protein |
|  |  | CarP2399: | phhA |
|  |  | CarP7030: | hypothetical protein |
|  |  | CarP2156: | hypothetical protein |
|  |  | CarP2925: | ruvA |
|  |  | CarP1001: | Transposase DDE domain protein |
|  |  | CarP6324: | mscK |
|  |  | CarP2638: | pspC |
|  |  | CarP2360: | hypothetical protein |
|  |  | CarP6922: | hypothetical protein |
|  |  | CarP5683: | Ubiquitin carboxyl-terminal hydrolase |
|  |  | CarP3993: | hypothetical protein |
|  |  | CarP2068: | bglK |
|  |  | CarP5079: | Alginate lyase |
|  |  | CarP6521: | hypothetical protein |
|  |  | CarP1018: | todS |
|  |  | CarP6799: | putative glycosyl transferase |
|  |  | CarP5090: | appC |
|  |  | CarP4147: | D-arabitol-phosphate dehydrogenase |
|  |  | CarP4240: | Ferredoxin, 2Fe-2S |
|  |  | CarP4262: | tetratricopeptide repeat protein |
|  |  | CarP4809: | hypothetical protein |
|  |  | CarP1203: | rcp1 |
|  |  | CarP5253: | hypothetical protein |
|  |  | CarP5434: | ydjH |
|  |  | CarP7260: | hypothetical protein |
|  |  | CarP5075: | Beta-1,4-mannooligosaccharide phosphorylas |
|  |  | CarP6397: | hypothetical protein |
|  |  | CarP2760: | hypothetical protein |
|  |  | CarP2720: | D-threonine aldolase |
|  |  | CarP1013: | cytR |
|  |  | CarP5578: | mopA |
|  |  | CarP6937: | ompH |
|  |  | CarP3052: | ktrB |
|  |  | CarP4053: | hypothetical protein |
|  |  | CarP4367: | hypothetical protein |
|  |  | CarP7079: | pehX |
|  |  | CarP1958: | RNA recognition motif. (a.k.a. RRM, RBD, o |
|  |  | CarP6052: | hypothetical protein |
|  |  | CarP4114: | atsA |
|  |  | CarP5072: | hypothetical protein |
|  |  | CarP6905: | MORN repeat variant |
|  |  | CarP2285: | Sepiapterin reductase |
|  |  | CarP7133: | fecR |
|  |  | CarP6475: | hypothetical protein |
|  |  | CarP6694: | nirM |
|  |  | CarP4454: | pyrC |
|  |  | CarP7442: | Daunorubicin doxorubicin resistance ATP-bi |
|  |  | CarP5404: | Beta-mannanase endoglucanase A precursor |
|  |  | CarP1593: | sigW |
|  |  | CarP3914: | Susd and RagB outer membrane lipoprotein |
|  |  | CarP3170: | rhaS |
|  |  | CarP5531: | hypothetical protein |
|  |  | CarP5212: | Imelysin |
|  |  | CarP2110: | hemC |
|  |  | CarP4243: | cfr |
|  |  | CarP3198: | Polysaccharide deacetylase |
|  |  | CarP3724: | zraS |
|  |  | CarP1211: | hypothetical protein |
|  |  | CarP4614: | sdcS |
|  |  | CarP6406: | Plasmid stabilisation system protein |
|  |  | CarP3062: | Putative acetyltransferase |
|  |  | CarP3467: | deaD |
|  |  | CarP6662: | Outer membrane efflux protein |
|  |  | CarP6158: | rebM |
|  |  | CarP4962: | rsbU |
|  |  | CarP6926: | hypothetical protein |
|  |  | CarP2781: | Outer membrane protein transport protein ( |
|  |  | CarP2509: | rfaC |
|  |  | CarP3084: | Beta-monoglucosyldiacylglycerol synthase |
|  |  | CarP4873: | rhgT |
|  |  | CarP2606: | SusD family protein |
|  |  | CarP5078: | F5 8 type C domain protein |
|  |  | CarP2586: | putative adenylyltransferase sulfurtransfe |
|  |  | CarP2614: | ndhD1 |
|  |  | CarP3518: | nuoJ |
|  |  | CarP5882: | yehT |
|  |  | CarP6704: | hypothetical protein |
|  |  | CarP2212: | dsbD |
|  |  | CarP3203: | hypothetical protein |
|  |  | CarP4653: | Acetyltransferase (GNAT) family protein |
|  |  | CarP2848: | thiE |
|  |  | CarP3867: | hypothetical protein |
|  |  | CarP4651: | Glycosyl hydrolases family 43 |
|  |  | CarP6626: | nreB |
|  |  | CarP3735: | azoB |
|  |  | CarP6983: | hypothetical protein |
|  |  | CarP3215: | hypothetical protein |
|  |  | CarP2677: | nagC |
|  |  | CarP4543: | hypothetical protein |
|  |  | CarP4896: | Acetyltransferase (GNAT) family protein |
|  |  | CarP6392: | Na+ Pi-cotransporter |
|  |  | CarP6019: | GRAS family transcription factor |
|  |  | CarP2645: | puuB |
|  |  | CarP3608: | TonB-dependent Receptor Plug Domain protei |
|  |  | CarP3427: | mro |
|  |  | CarP2705: | phhB |
|  |  | CarP2571: | NmrA-like family protein |
|  |  | CarP6286: | hemL |
|  |  | CarP2114: | yehU |
|  |  | CarP3889: | hypothetical protein |
|  |  | CarP1933: | putative transposase |
|  |  | CarP5861: | hypothetical protein |
|  |  | CarP7075: | hypothetical protein |
|  |  | CarP1631: | sigG |
|  |  | CarP2787: | erpA |
|  |  | CarP6940: | hypothetical protein |
|  |  | CarP5460: | hypothetical protein |
|  |  | CarP2177: | hypothetical protein |
|  |  | CarP2264: | fdx |
|  |  | CarP6765: | hypothetical protein |
|  |  | CarP2178: | uroporphyrinogen-III synthase |
|  |  | CarP6063: | czcR |
|  |  | CarP5391: | ccrA |
|  |  | CarP5450: | hypothetical protein |
|  |  | CarP1595: | putative metallophosphoesterase |
|  |  | CarP1628: | hypothetical protein |
|  |  | CarP4634: | hypothetical protein |
|  |  | CarP6159: | lplJ |
|  |  | CarP7164: | ptrA |
|  |  | CarP2030: | ybfF |
|  |  | CarP5420: | kynB |
|  |  | CarP3970: | Acetyltransferase (GNAT) family protein |
|  |  | CarP6281: | hypothetical protein |
|  |  | CarP4789: | paaE |
|  |  | CarP2034: | patA |
|  |  | CarP6492: | cstA |
|  |  | CarP4536: | hypothetical protein |
|  |  | CarP3192: | Transglutaminase-like superfamily protein |
|  |  | CarP7375: | ptpA |
|  |  | CarP7084: | hypothetical protein |
|  |  | CarP4345: | hypothetical protein |
|  |  | CarP1617: | arsC |
|  |  | CarP3554: | legF |
|  |  | CarP7359: | pcaH |
|  |  | CarP2010: | hypothetical protein |
|  |  | CarP6692: | hypothetical protein |
|  |  | CarP3961: | vat |
|  |  | CarP7530: | hypothetical protein |
|  |  | CarP7112: | ybiA |
|  |  | CarP1880: | hypothetical protein |
|  |  | CarP6231: | yrrB |
|  |  | CarP5666: | scpA |
|  |  | CarP2937: | acdA |
|  |  | CarP7282: | Putative endoglucanase |
|  |  | CarP5387: | Alpha beta hydrolase family protein |
|  |  | CarP1861: | folE |
|  |  | CarP5154: | atsA |
|  |  | CarP3240: | prenyltransferase |
|  |  | CarP3711: | SusD family protein |
|  |  | CarP5591: | Putative esterase |
|  |  | CarP2236: | kdtA |
|  |  | CarP2103: | cysH |
|  |  | CarP7015: | yehU |
|  |  | CarP6717: | SusD family protein |
|  |  | CarP1741: | Membrane protein of unknown function |
|  |  | CarP2203: | motB |
|  |  | CarP4389: | hypothetical protein |
|  |  | CarP2494: | hypothetical protein |
|  |  | CarP3209: | phyH |
|  |  | CarP6735: | afr |
|  |  | CarP2869: | ydfG |
|  |  | CarP3971: | axeA1 |
|  |  | CarP4436: | sigW |
|  |  | CarP7655: | yehT |
|  |  | CarP1900: | hutI |
|  |  | CarP3908: | Susd and RagB outer membrane lipoprotein |
|  |  | CarP2312: | hypothetical protein |
|  |  | CarP5484: | corA |
|  |  | CarP3401: | Naphthalene 1,2-dioxygenase salicylate 5-h |
|  |  | CarP5565: | pepT |
|  |  | CarP2138: | dxr |
|  |  | CarP3680: | hypothetical protein |
|  |  | CarP2025: | azo1 |
|  |  | CarP3814: | Sodium glucose cotransporter |
|  |  | CarP4630: | epsE |
|  |  | CarP4146: | Metallo-beta-lactamase superfamily protein |
|  |  | CarP6563: | hypothetical protein |
|  |  | CarP7250: | Y Y Y domain protein |
|  |  | CarP6860: | fnr |
|  |  | CarP5677: | hypothetical protein |
|  |  | CarP3633: | Xylose isomerase-like TIM barrel |
|  |  | CarP7478: | phzF |
|  |  | CarP7115: | gdhI |
|  |  | CarP5991: | hypothetical protein |
|  |  | CarP7240: | MarR family protein |
|  |  | CarP1814: | recO |
|  |  | CarP4130: | hypothetical protein |
|  |  | CarP7507: | Acetyltransferase (GNAT) family protein |
|  |  | CarP3949: | hypothetical protein |
|  |  | CarP3017: | hypothetical protein |
|  |  | CarP7338: | yhcR |
|  |  | CarP3705: | pdtaS |
|  |  | CarP2707: | corC |
|  |  | CarP3991: | galE |
|  |  | CarP6532: | Biopolymer transport protein ExbD TolR |
|  |  | CarP5311: | hypothetical protein |
|  |  | CarP3087: | hypothetical protein |
|  |  | CarP5291: | RNA 2'-phosphotransferase |
|  |  | CarP5369: | vraR |
|  |  | CarP2581: | ptrB |
|  |  | CarP6895: | hypothetical protein |
|  |  | CarP4047: | hypothetical protein |
|  |  | CarP5259: | tonB |
|  |  | CarP3683: | rpoE |
|  |  | CarP2960: | albA |
|  |  | CarP4012: | arnT |
|  |  | CarP4237: | hypothetical protein |
|  |  | CarP4290: | Transglutaminase-like superfamily protein |
|  |  | CarP5585: | bifunctional 3-demethylubiquinone-9 3-methyltransferase/ 2-octaprenyl-6-hydroxy phenol methylase |
|  |  | CarP3525: | nuoC1 |
|  |  | CarP4022: | hemS |
|  |  | CarP4913: | Carboxymuconolactone decarboxylase family |
|  |  | CarP6255: | paaH |
|  |  | CarP4685: | hypothetical protein |
|  |  | CarP6328: | bigR |
|  |  | CarP5109: | Nucleotidyltransferase domain protein |
|  |  | CarP3566: | hypothetical protein |
|  |  | CarP1657: | hypothetical protein |
|  |  | CarP5218: | ABC-2 family transporter protein |
|  |  | CarP5380: | hypothetical protein |
|  |  | CarP5288: | amy |
|  |  | CarP3311: | asnB |
|  |  | CarP4269: | hypothetical protein |
|  |  | CarP2443: | hypothetical protein |
|  |  | CarP5913: | rhaR |
|  |  | CarP3783: | rcsC |
|  |  | CarP7466: | PemK-like protein |
|  |  | CarP2709: | hypothetical protein |
|  |  | CarP5007: | Na(+) H(+) antiporter subunit B |
|  |  | CarP6518: | trmH |
|  |  | CarP4397: | csbB |
|  |  | CarP5944: | SnoaL-like domain protein |
|  |  | CarP3165: | qdoI |
|  |  | CarP5064: | ltaS2 |
|  |  | CarP3386: | qorA |
|  |  | CarP4118: | hypothetical protein |
|  |  | CarP2836: | yfgC |
|  |  | CarP3779: | Gram-negative bacterial tonB protein |
|  |  | CarP7199: | hypothetical protein |
|  |  | CarP1792: | hypothetical protein |
|  |  | CarP7456: | arlS |
|  |  | CarP7729: | cas1 |
|  |  | CarP2597: | hypothetical protein |
|  |  | CarP2131: | coaD |
|  |  | CarP4177: | yhdG |
|  |  | CarP5199: | cysQ |
|  |  | CarP6015: | arsenite S-adenosylmethyltransferase |
|  |  | CarP5618: | yteR |
|  |  | CarP3317: | araA |
|  |  | CarP3383: | Helix-turn-helix |
|  |  | CarP7110: | hypothetical protein |
|  |  | CarP2057: | hypothetical protein |
|  |  | CarP1813: | hypothetical protein |
|  |  | CarP2799: | Cytochrome b5-like Heme Steroid binding do |
|  |  | CarP1020: | IS1 transposase |
|  |  | CarP1956: | hypothetical protein |
|  |  | CarP1762: | Phosphomannomutase phosphoglucomutase |
|  |  | CarP6439: | hypothetical protein |
|  |  | CarP3430: | fdm |
|  |  | CarP3669: | hypothetical protein |
|  |  | CarP6157: | hypothetical protein |
|  |  | CarP7681: | hypothetical protein |
|  |  | CarP4675: | hypothetical protein |
|  |  | CarP7168: | hypothetical protein |
|  |  | CarP5592: | hypothetical protein |
|  |  | CarP6433: | hypothetical protein |
|  |  | CarP3719: | hypothetical protein |
|  |  | CarP4584: | SusD family protein |
|  |  | CarP2015: | rssA |
|  |  | CarP4920: | pupA |
|  |  | CarP3315: | SusD family protein |
|  |  | CarP6968: | CHAT domain protein |
|  |  | CarP3920: | mshA |
|  |  | CarP7550: | nuoL |
|  |  | CarP5969: | hypothetical protein |
|  |  | CarP1732: | Virginiamycin B lyase |
|  |  | CarP4790: | tolC |
|  |  | CarP4636: | hypothetical protein |
|  |  | CarP6535: | hypothetical protein |
|  |  | CarP4843: | hupR1 |
|  |  | CarP6338: | yycF |
|  |  | CarP1932: | D-alanyl-D-alanine-carboxypeptidase endopeptidase |
|  |  | CarP5058: | L-gulono-1,4-lactone dehydrogenase |
|  |  | CarP6622: | ytrE |
|  |  | CarP2202: | ybdL |
|  |  | CarP6844: | mmpI |
|  |  | CarP3034: | hypothetical protein |
|  |  | CarP7054: | hypothetical protein |
|  |  | CarP5357: | hypothetical protein |
|  |  | CarP6815: | gdhB |
|  |  | CarP4528: | csgD |
|  |  | CarP6759: | thiC |
|  |  | CarP5138: | 2-keto-3-deoxy-galactonokinase |
|  |  | CarP4039: | outer membrane channel protein |
|  |  | CarP2434: | oprF |
|  |  | CarP5305: | ccp |
|  |  | CarP5200: | polC |
|  |  | CarP6334: | xynB |
|  |  | CarP5631: | dsbH |
|  |  | CarP4021: | btuF |
|  |  | CarP7025: | gdh |
|  |  | CarP4273: | hypothetical protein |
|  |  | CarP3118: | hypothetical protein |
|  |  | CarP7294: | ypdB |
|  |  | CarP2933: | serC |
|  |  | CarP2513: | fabG |
|  |  | CarP4598: | hypothetical protein |
|  |  | CarP3515: | yqjA |
|  |  | CarP3851: | macA |
|  |  | CarP6565: | SusD family protein |
|  |  | CarP2141: | tetratricopeptide repeat protein |
|  |  | CarP7545: | hypothetical protein |
|  |  | CarP7072: | hypothetical protein |
|  |  | CarP2415: | rpsA |
|  |  | CarP5852: | hypothetical protein |
|  |  | CarP4881: | mltD |
|  |  | CarP2657: | bepF |
|  |  | CarP6793: | Polysaccharide biosynthesis protein |
|  |  | CarP1674: | gpmA |
|  |  | CarP6421: | hypothetical protein |
|  |  | CarP2879: | aprN |
|  |  | CarP1790: | thiL |
|  |  | CarP4383: | Transcriptional regulator PadR-like family |
|  |  | CarP7202: | hypothetical protein |
|  |  | CarP3768: | rfaH |
|  |  | CarP3965: | FG-GAP repeat protein |
|  |  | CarP1856: | Putative neutral zinc metallopeptidase |
|  |  | CarP1504: | Bifunctional NMN adenylyltransferase Nudix |
|  |  | CarP3811: | hypothetical protein |
|  |  | CarP4728: | hypothetical protein |
|  |  | CarP6118: | csgE |
|  |  | CarP2779: | yhdN |
|  |  | CarP1509: | ftrB |
|  |  | CarP5372: | SusD family protein |
|  |  | CarP3679: | aprX |
|  |  | CarP6291: | hypothetical protein |
|  |  | CarP3863: | hypothetical protein |
|  |  | CarP4520: | atsA |
|  |  | CarP5845: | hypothetical protein |
|  |  | CarP2119: | sulD |
|  |  | CarP6487: | greA |
|  |  | CarP6414: | ade2 |
|  |  | CarP3114: | mce related protein |
|  |  | CarP5758: | DinB superfamily protein |
|  |  | CarP7330: | hypothetical protein |
|  |  | CarP2246: | rimP |
|  |  | CarP1065: | hypothetical protein |
|  |  | CarP7398: | Retaining alpha-galactosidase precursor |
|  |  | CarP5009: | hypothetical protein |
|  |  | CarP4660: | hypothetical protein |
|  |  | CarP3549: | Peptidase family S41 |
|  |  | CarP5579: | mprA |
|  |  | CarP7005: | hypothetical protein |
|  |  | CarP3424: | btuB |
|  |  | CarP4892: | modA |
|  |  | CarP7052: | Type IIS restriction enzyme Eco57I |
|  |  | CarP6402: | perR |
|  |  | CarP7288: | hypothetical protein |
|  |  | CarP5032: | smc |
|  |  | CarP6128: | hypothetical protein |
|  |  | CarP4884: | hypothetical protein |
|  |  | CarP5418: | Antibiotic biosynthesis monooxygenase |
|  |  | CarP1154: | fepA |
|  |  | CarP3419: | hypothetical protein |
|  |  | CarP1689: | yoeB |
|  |  | CarP7704: | hypothetical protein |
|  |  | CarP7313: | Phage terminase, small subunit |
|  |  | CarP7397: | hypothetical protein |
|  |  | CarP2000: | Bifunctional aspartokinase homoserine dehy |
|  |  | CarP4868: | Putative DNA ligase-like protein MT0965 |
|  |  | CarP3176: | dadA |
|  |  | CarP2295: | ilvD |
|  |  | CarP5202: | Heme oxygenase |
|  |  | CarP1934: | hypothetical protein |
|  |  | CarP5020: | cat |
|  |  | CarP3890: | mdtC |
|  |  | CarP3537: | putative ABC transporter ATP-binding protein |
|  |  | CarP6772: | hypothetical protein |
|  |  | CarP4855: | exoaminopeptidase |
|  |  | CarP6127: | hypothetical protein |
|  |  | CarP6471: | hypothetical protein |
|  |  | CarP3764: | Glycosyl hydrolase family 92 |
|  |  | CarP6339: | phoR |
|  |  | CarP5430: | hypothetical protein |
|  |  | CarP3850: | bepF |
|  |  | CarP2059: | luxC |
|  |  | CarP6344: | ybaQ |
|  |  | CarP2351: | tetratricopeptide repeat protein |
|  |  | CarP1852: | GH3 auxin-responsive promoter |
|  |  | CarP4984: | Glyoxalase-like domain protein |
|  |  | CarP7193: | ycjS |
|  |  | CarP4864: | Trehalose utilisation |
|  |  | CarP5375: | ybaQ |
|  |  | CarP1982: | qorB |
|  |  | CarP2293: | Beta-monoglucosyldiacylglycerol synthase |
|  |  | CarP3653: | SusD family protein |
|  |  | CarP2338: | aroA |
|  |  | CarP7187: | hypothetical protein |
|  |  | CarP6089: | Transcriptional regulator PadR-like family |
|  |  | CarP3387: | D-arabitol-phosphate dehydrogenase |
|  |  | CarP5979: | tetratricopeptide repeat protein |
|  |  | CarP5514: | btr |
|  |  | CarP5394: | hypothetical protein |
|  |  | CarP1997: | Polyketide cyclase/dehydrase and lipid t |
|  |  | CarP4136: | hypothetical protein |
|  |  | CarP2215: | aroD |
|  |  | CarP7228: | doxX |
|  |  | CarP1201: | hypothetical protein |
|  |  | CarP6795: | Acyltransferase family protein |
|  |  | CarP1038: | fecR |
|  |  | CarP2974: | slrP |
|  |  | CarP3280: | ldhA |
|  |  | CarP1600: | pyk |
|  |  | CarP2689: | hypothetical protein |
|  |  | CarP3803: | mscS |
|  |  | CarP4083: | hldD |
|  |  | CarP6040: | divJ |
|  |  | CarP2570: | putative ABC transporter ATP-binding protein |
|  |  | CarP6729: | hypothetical protein |
|  |  | CarP3164: | Neutral alkaline non-lysosomal ceramidase |
|  |  | CarP1534: | gfo |
|  |  | CarP3186: | nagA |
|  |  | CarP6026: | uspE |
|  |  | CarP7096: | btuB |
|  |  | CarP5139: | iorA |
|  |  | CarP1517: | hypothetical protein |
|  |  | CarP4006: | Transposase IS200 like protein |
|  |  | CarP7631: | ohrA |
|  |  | CarP2527: | Di-glucose binding within endoplasmic reti |
|  |  | CarP6267: | hypothetical protein |
|  |  | CarP2842: | FKBP-type peptidyl-prolyl cis-trans isomerase |
|  |  | CarP3793: | lysC |
|  |  | CarP5065: | SusD family protein |
|  |  | CarP6581: | cph1 |
|  |  | CarP2765: | L-Ala-D L-Glu epimerase |
|  |  | CarP6610: | hypothetical protein |
|  |  | CarP5465: | hypothetical protein |
|  |  | CarP3567: | hldE |
|  |  | CarP7196: | hypothetical protein |
|  |  | CarP4019: | hypothetical protein |
|  |  | CarP4020: | hmuT |
|  |  | CarP5261: | hypothetical protein |
|  |  | CarP4902: | carbamoyl phosphate synthase-like protein |
|  |  | CarP5830: | hypothetical protein |
|  |  | CarP2426: | proC |
|  |  | CarP4086: | pfkB |
|  |  | CarP5877: | dac |
|  |  | CarP2211: | oxyR |
|  |  | CarP1160: | glpC |
|  |  | CarP1056: | hypothetical protein |
|  |  | CarP2558: | aacA4 |
|  |  | CarP1609: | Planctomycete cytochrome C |
|  |  | CarP4298: | hypothetical protein |
|  |  | CarP5654: | Green-light absorbing proteorhodopsin prec |
|  |  | CarP3497: | ydjH |
|  |  | CarP7735: | hypothetical protein |
|  |  | CarP1218: | cph1 |
|  |  | CarP6727: | hypothetical protein |
|  |  | CarP3860: | idhA |
|  |  | CarP1676: | Stearoyl-CoA 9-desaturase |
|  |  | CarP7029: | ybaQ |
|  |  | CarP3891: | mdtE |
|  |  | CarP4901: | hypothetical protein |
|  |  | CarP6660: | Matrixin |
|  |  | CarP1311: | Bifunctional transcriptional activator DNA |
|  |  | CarP4737: | PqqC-like protein |
|  |  | CarP2775: | ilvH |
|  |  | CarP6261: | hypothetical protein |
|  |  | CarP1522: | hypothetical protein |
|  |  | CarP6819: | livF |
|  |  | CarP2237: | rsgA |
|  |  | CarP4275: | Phenylacetate-coenzyme A ligase |
|  |  | CarP1981: | hypothetical protein |
|  |  | CarP3960: | cph1 |
|  |  | CarP6005: | hypothetical protein |
|  |  | CarP2035: | rocD |
|  |  | CarP7353: | Erythromycin esterase |
|  |  | CarP3877: | nanB |
|  |  | CarP5767: | hypothetical protein |
|  |  | CarP2656: | moeZ |
|  |  | CarP6361: | hypothetical protein |
|  |  | CarP4303: | Glyoxalase Bleomycin resistance protein Di |
|  |  | CarP2583: | hypothetical protein |
|  |  | CarP4330: | Extracellular xylan exo-alpha-(1->2)-glucu |
|  |  | CarP7254: | hypothetical protein |
|  |  | CarP7757: | bioC |
|  |  | CarP2678: | ilvE |
|  |  | CarP2260: | queG |
|  |  | CarP3488: | phoP |
|  |  | CarP6779: | iutA |
|  |  | CarP6538: | hypothetical protein |
|  |  | CarP1873: | kpsM |
|  |  | CarP3974: | Plasmid stabilisation system protein |
|  |  | CarP5103: | ptlH |
|  |  | CarP3966: | hypothetical protein |
|  |  | CarP7101: | SusD family protein |
|  |  | CarP6649: | metal-dependent hydrolase |
|  |  | CarP2384: | metH |
|  |  | CarP5988: | hypothetical protein |
|  |  | CarP3751: | Macrolide export ATP-binding permease prot |
|  |  | CarP7295: | hypothetical protein |
|  |  | CarP3824: | dctA |
|  |  | CarP5740: | hypothetical protein |
|  |  | CarP1822: | gdhA |
|  |  | CarP4230: | hypothetical protein |
|  |  | CarP5983: | hypothetical protein |
|  |  | CarP2273: | nrfD |
|  |  | CarP5088: | SusD family protein |
|  |  | CarP5276: | RDD family protein |
|  |  | CarP3699: | Periplasmic binding protein |
|  |  | CarP5841: | hypothetical protein |
|  |  | CarP3715: | hypothetical protein |
|  |  | CarP6920: | hypothetical protein |
|  |  | CarP5516: | petC |
|  |  | CarP5137: | metal-dependent hydrolase |
|  |  | CarP5689: | Ankyrinrepeats (3 copies) |
|  |  | CarP5569: | Beta-1,4-mannooligosaccharide phosphorylas |
|  |  | CarP4407: | hypothetical protein |
|  |  | CarP4860: | AsmA family protein |
|  |  | CarP4133: | Farnesoic acid 0-methyl transferase |
|  |  | CarP5815: | Bacterial Ig-like domain (group 1) |
|  |  | CarP5355: | hypothetical protein |
|  |  | CarP1954: | hypothetical protein |
|  |  | CarP4232: | manA |
|  |  | CarP6527: | hypothetical protein |
|  |  | CarP7675: | hypothetical protein |
|  |  | CarP5632: | hypothetical protein |
|  |  | CarP2990: | prrA |
|  |  | CarP6547: | hypothetical protein |
|  |  | CarP3873: | hypothetical protein |
|  |  | CarP1906: | fnr |
|  |  | CarP3838: | rhlE |
|  |  | CarP4193: | hypothetical protein |
|  |  | CarP3057: | hypothetical protein |
|  |  | CarP6138: | cls |
|  |  | CarP6746: | hypothetical protein |
|  |  | CarP5489: | hypothetical protein |
|  |  | CarP6955: | cph1 |
|  |  | CarP2944: | hypothetical protein |
|  |  | CarP4627: | kch |
|  |  | CarP2266: | (2E,6E)-farnesyl diphosphate synthase |
|  |  | CarP2041: | bcr |
|  |  | CarP6804: | hypothetical protein |
|  |  | CarP5680: | putative lyase |
|  |  | CarP2287: | hypothetical protein |
|  |  | CarP1588: | tonB |
|  |  | CarP3028: | rspA |
|  |  | CarP6520: | hypothetical protein |
|  |  | CarP7602: | Ser-Thr-rich glycosyl-phosphatidyl-inosito |
|  |  | CarP7264: | phy |
|  |  | CarP2592: | hypothetical protein |
|  |  | CarP6923: | Pregnancy-associated plasma protein-A |
|  |  | CarP2218: | gltB |
|  |  | CarP1773: | ABC-type uncharacterized transport system |
|  |  | CarP6714: | Phenolphthiocerol synthesis polyketide syn |
|  |  | CarP3006: | psaB |
|  |  | CarP5004: | Na(+) H(+) antiporter subunit E |
|  |  | CarP3767: | betI |
|  |  | CarP7709: | NYN domain protein |
|  |  | CarP3005: | hypothetical protein |
|  |  | CarP7299: | hypothetical protein |
|  |  | CarP1594: | hypothetical protein |
|  |  | CarP7217: | hypothetical protein |
|  |  | CarP6403: | hypothetical protein |
|  |  | CarP2122: | HD domain protein |
|  |  | CarP3916: | 2-acyl-glycerophospho-ethanolamine acyltra |
|  |  | CarP3979: | hpxO |
|  |  | CarP4817: | High-affinity Na(+) H(+) antiporter NhaS3 |
|  |  | CarP3022: | hypothetical protein |
|  |  | CarP7293: | yehT |
|  |  | CarP6606: | von Willebrand factor type A domain protei |
|  |  | CarP3286: | lutA |
|  |  | CarP3421: | hldD |
|  |  | CarP6889: | mta |
|  |  | CarP1983: | pbpE |
|  |  | CarP2771: | putative glycosyl transferase |
|  |  | CarP2404: | menD |
|  |  | CarP2697: | SNARE associated Golgi protein |
|  |  | CarP7142: | yghA |
|  |  | CarP3335: | groES |
|  |  | CarP1953: | tuaB |
|  |  | CarP5681: | hypothetical protein |
|  |  | CarP5649: | caiD |
|  |  | CarP3211: | dgoD |
|  |  | CarP1875: | hypothetical protein |
|  |  | CarP3886: | Acetyltransferase (GNAT) family protein |
|  |  | CarP2221: | purK |
|  |  | CarP1764: | tonB |
|  |  | CarP3137: | hypothetical protein |
|  |  | CarP6047: | rcp1 |
|  |  | CarP2688: | sigE |
|  |  | CarP3635: | hypothetical protein |
|  |  | CarP6108: | hypothetical protein |
|  |  | CarP1976: | yrrB |
|  |  | CarP7745: | paiA |
|  |  | CarP4816: | ribonuclease D |
|  |  | CarP3229: | hypothetical protein |
|  |  | CarP7458: | slmA |
|  |  | CarP6681: | hypothetical protein |
|  |  | CarP5521: | mntH |
|  |  | CarP4581: | bdcA |
|  |  | CarP2735: | beta-lactamase D-alanine carboxypeptidase |
|  |  | CarP5297: | Serine threonine-protein kinase PknD |
|  |  | CarP6871: | MarR family protein |
|  |  | CarP3433: | moaE1 |
|  |  | CarP4987: | YciI-like protein |
|  |  | CarP4970: | crp |
|  |  | CarP1642: | ybaQ |
|  |  | CarP1713: | arlS |
|  |  | CarP3963: | EcsC protein family protein |
|  |  | CarP4151: | Membrane bound L-sorbosone dehydrogenase |
|  |  | CarP7141: | btr |
|  |  | CarP6355: | crtI |
|  |  | CarP6839: | glsA |
|  |  | CarP4992: | mraZ |
|  |  | CarP6250: | korB |
|  |  | CarP5071: | yccM |
|  |  | CarP5373: | Peptidase C10 family protein |
|  |  | CarP2995: | xerC |
|  |  | CarP7626: | hypothetical protein |
|  |  | CarP7593: | exuT |
|  |  | CarP6762: | hypothetical protein |
|  |  | CarP4180: | hypothetical protein |
|  |  | CarP2526: | kpsD |
|  |  | CarP2315: | scdA |
|  |  | CarP1991: | bcp |
|  |  | CarP6117: | hypothetical protein |
|  |  | CarP5283: | adhC2 |
|  |  | CarP2292: | sigK |
|  |  | CarP6974: | hypothetical protein |
|  |  | CarP6425: | hypothetical protein |
|  |  | CarP4734: | ycgJ |
|  |  | CarP6734: | hypothetical protein |
|  |  | CarP1745: | exo I |
|  |  | CarP6721: | nagA |
|  |  | CarP4289: | hypothetical protein |
|  |  | CarP3163: | Helix-turn-helix domain protein |
|  |  | CarP5574: | Acetyltransferase (GNAT) family protein |
|  |  | CarP2280: | ctaE |
|  |  | CarP3649: | lacA |
|  |  | CarP4098: | Neutral ceramidase precursor |
|  |  | CarP5102: | idhA |
|  |  | CarP4852: | Amylo-alpha-1,6-glucosidase |
|  |  | CarP6027: | hypothetical protein |
|  |  | CarP4127: | hypothetical protein |
|  |  | CarP5858: | hypothetical protein |
|  |  | CarP4306: | hypothetical protein |
|  |  | CarP2654: | hypothetical protein |
|  |  | CarP2238: | pyrR |
|  |  | CarP3969: | atsA |
|  |  | CarP3282: | DinB superfamily protein |
|  |  | CarP5256: | DinB superfamily protein |
|  |  | CarP5045: | uspE |
|  |  | CarP3872: | nanM |
|  |  | CarP6756: | hypothetical protein |
|  |  | CarP7207: | arfA |
|  |  | CarP2430: | hypothetical protein |
|  |  | CarP4332: | tetratricopeptide repeat protein |
|  |  | CarP3852: | Collagen triple helix repeat (20 copies) |
|  |  | CarP2572: | LytTr DNA-binding domain protein |
|  |  | CarP4938: | creD |
|  |  | CarP5104: | hypothetical protein |
|  |  | CarP4297: | DinB superfamily protein |
|  |  | CarP4580: | hypothetical protein |
|  |  | CarP4523: | Glycosyl hydrolases family 43 |
|  |  | CarP6605: | hypothetical protein |
|  |  | CarP5410: | hypothetical protein |
|  |  | CarP3990: | Redoxin |
|  |  | CarP3702: | hypothetical protein |
|  |  | CarP4718: | arnT |
|  |  | CarP7578: | hypothetical protein |
|  |  | CarP7372: | hypothetical protein |
|  |  | CarP4521: | hypothetical protein |
|  |  | CarP5205: | hypothetical protein |
|  |  | CarP4741: | hypothetical protein |
|  |  | CarP6121: | tolB |
|  |  | CarP2353: | hypothetical protein |
|  |  | CarP5406: | cphB |
|  |  | CarP3109: | hypothetical protein |
|  |  | CarP6794: | hypothetical protein |
|  |  | CarP3446: | rpoE |
|  |  | CarP5365: | hypothetical protein |
|  |  | CarP4990: | fucP |
|  |  | CarP6033: | hypothetical protein |
|  |  | CarP3018: | hypothetical protein |
|  |  | CarP7083: | M61 glycyl aminopeptidase |
|  |  | CarP2708: | hypothetical protein |
|  |  | CarP4041: | yehU |
|  |  | CarP5207: | thiG |
|  |  | CarP1004: | btuB |
|  |  | CarP7563: | hypothetical protein |
|  |  | CarP4486: | Sodium glucose cotransporter |
|  |  | CarP7411: | IPT TIG domain protein |
|  |  | CarP5954: | hypothetical protein |
|  |  | CarP5850: | hypothetical protein |
|  |  | CarP6912: | lysDH |
|  |  | CarP6786: | hypothetical protein |
|  |  | CarP6531: | Biopolymer transport protein ExbD TolR |
|  |  | CarP6825: | gntP |
|  |  | CarP2562: | plcN |
|  |  | CarP7632: | hypothetical protein |
|  |  | CarP6919: | tmk |
|  |  | CarP3402: | nasD |
|  |  | CarP4819: | hypothetical protein |
|  |  | CarP6797: | oatA |
|  |  | CarP2425: | ahcY |
|  |  | CarP7639: | Blue-light-activated histidine kinase 1 |
|  |  | CarP1962: | hypothetical protein |
|  |  | CarP5999: | LytTr DNA-binding domain protein |
|  |  | CarP7657: | todS |
|  |  | CarP3173: | pgmB |
|  |  | CarP5089: | NYN domain protein |
|  |  | CarP2547: | sigY |
|  |  | CarP4757: | fhuC |
|  |  | CarP1725: | hypothetical protein |
|  |  | CarP5899: | Carbohydrate binding module (family 6) |
|  |  | CarP4650: | hypothetical protein |
|  |  | CarP5571: | hepA |
|  |  | CarP5624: | hypothetical protein |
|  |  | CarP4608: | TonB-dependent Receptor Plug Domain protei |
|  |  | CarP6135: | hypothetical protein |
|  |  | CarP7389: | hypothetical protein |
|  |  | CarP5603: | dUMP phosphatase |
|  |  | CarP6097: | HNH endonuclease |
|  |  | CarP5345: | thrB |
|  |  | CarP5617: | mdlB |
|  |  | CarP3361: | Calcineurin-like phosphoesterase |
|  |  | CarP3594: | hypothetical protein |
|  |  | CarP6453: | acuI |
|  |  | CarP4507: | Phage Mu protein F like protein |
|  |  | CarP1675: | putative hydrolase |
|  |  | CarP7319: | rfaQ |
|  |  | CarP3778: | epsD |
|  |  | CarP4597: | hypothetical protein |
|  |  | CarP4914: | ahpD |
| "CardiniumPangenome | ONLY" | 178 |  |
|  |  | CarP4779: | tmk |
|  |  | CarP5729: | ankX |
|  |  | CarP3624: | Ankyrinrepeats (3 copies) |
|  |  | CarP2980: | SET domain protein |
|  |  | CarP4502: | hypothetical protein |
|  |  | CarP5691: | Sodium:solute symporter family protein |
|  |  | CarP5699: | hlyD |
|  |  | CarP4769: | hypothetical protein |
|  |  | CarP6217: | hypothetical protein |
|  |  | CarP2521: | hypothetical protein |
|  |  | CarP5714: | OPT oligopeptide transporter protein |
|  |  | CarP1081: | hypothetical protein |
|  |  | CarP5696: | hypothetical protein |
|  |  | CarP5734: | hypothetical protein |
|  |  | CarP4499: | Alpha beta hydrolase family protein |
|  |  | CarP5720: | mnaA |
|  |  | CarP6187: | hypothetical protein |
|  |  | CarP5692: | hypothetical protein |
|  |  | CarP4714: | putative hydrolase |
|  |  | CarP4771: | ankX |
|  |  | CarP4496: | hypothetical protein |
|  |  | CarP6207: | hypothetical protein |
|  |  | CarP1924: | hypothetical protein |
|  |  | CarP4753: | Ankyrinrepeats (3 copies) |
|  |  | CarP4761: | ybhL |
|  |  | CarP5710: | hypothetical protein |
|  |  | CarP4767: | ppdK |
|  |  | CarP4500: | hypothetical protein |
|  |  | CarP4756: | hypothetical protein |
|  |  | CarP1930: | Transposase DDE domain protein |
|  |  | CarP5693: | Transposase DDE domain protein |
|  |  | CarP2985: | hypothetical protein |
|  |  | CarP4782: | CAAX amino terminal protease self- immunity |
|  |  | CarP3613: | putative transporter |
|  |  | CarP4493: | hypothetical protein |
|  |  | CarP3631: | nfo |
|  |  | CarP6186: | hypothetical protein |
|  |  | CarP3626: | Ubiquitin carboxyl-terminal hydrolase |
|  |  | CarP1037: | Sodium/proline symporter |
|  |  | CarP3625: | tlcA |
|  |  | CarP4777: | Mitochondrial carrier protein |
|  |  | CarP4503: | hypothetical protein |
|  |  | CarP6218: | hypothetical protein |
|  |  | CarP5721: | gltX2 |
|  |  | CarP5715: | Ankyrinrepeats (3 copies) |
|  |  | CarP3621: | Ankyrinrepeats (3 copies) |
|  |  | CarP6204: | hypothetical protein |
|  |  | CarP3615: | hypothetical protein |
|  |  | CarP5706: | hypothetical protein |
|  |  | CarP3619: | Ankyrinrepeats (3 copies) |
|  |  | CarP6221: | hypothetical protein |
|  |  | CarP5701: | hypothetical protein |
|  |  | CarP1512: | Leucine Rich repeats (2 copies) |
|  |  | CarP6222: | ankX |
|  |  | CarP6224: | bioC |
|  |  | CarP6206: | hypothetical protein |
|  |  | CarP5697: | Transposase IS66 family protein |
|  |  | CarP6215: | hypothetical protein |
|  |  | CarP6208: | hypothetical protein |
|  |  | CarP5705: | hypothetical protein |
|  |  | CarP2978: | Bifunctional (p)ppGpp synthase hydrolase R |
|  |  | CarP5711: | hypothetical protein |
|  |  | CarP6216: | hypothetical protein |
|  |  | CarP4754: | hypothetical protein |
|  |  | CarP3620: | hypothetical protein |
|  |  | CarP5727: | hypothetical protein |
|  |  | CarP5703: | hypothetical protein |
|  |  | CarP4772: | hypothetical protein |
|  |  | CarP5708: | hypothetical protein |
|  |  | CarP4770: | hypothetical protein |
|  |  | CarP3614: | Transposase DDE domain protein |
|  |  | CarP6192: | hypothetical protein |
|  |  | CarP5707: | hypothetical protein |
|  |  | CarP4747: | hypothetical protein |
|  |  | CarP6209: | hypothetical protein |
|  |  | CarP5726: | Zinc finger, C3H C4 type (RING finger) |
|  |  | CarP2982: | hypothetical protein |
|  |  | CarP6212: | hypothetical protein |
|  |  | CarP6205: | Ankyrinrepeats (3 copies) |
|  |  | CarP6191: | Sodium/proline symporter |
|  |  | CarP3616: | Transposase IS66 family protein |
|  |  | CarP6214: | hypothetical protein |
|  |  | CarP6223: | hypothetical protein |
|  |  | CarP2984: | hypothetical protein |
|  |  | CarP2524: | hypothetical protein |
|  |  | CarP4759: | CAAX amino terminal protease self- immunity |
|  |  | CarP6197: | hypothetical protein |
|  |  | CarP5704: | hypothetical protein |
|  |  | CarP6130: | hypothetical protein |
|  |  | CarP5725: | hypothetical protein |
|  |  | CarP4784: | Bacterial regulatory protein, Fis family |
|  |  | CarP6195: | hypothetical protein |
|  |  | CarP1929: | hypothetical protein |
|  |  | CarP4760: | hypothetical protein |
|  |  | CarP1601: | Transposase DDE domain protein |
|  |  | CarP5690: | hypothetical protein |
|  |  | CarP1082: | hypothetical protein |
|  |  | CarP3629: | Bacterial leucyl aminopeptidase precursor |
|  |  | CarP6220: | Alpha/beta hydrolase family protein |
|  |  | CarP6199: | hypothetical protein |
|  |  | CarP6183: | hypothetical protein |
|  |  | CarP3630: | hypothetical protein |
|  |  | CarP1927: | hypothetical protein |
|  |  | CarP5732: | hypothetical protein |
|  |  | CarP4762: | anaerobic C4-dicarboxylate transporter |
|  |  | CarP6194: | WH2 motif protein |
|  |  | CarP6229: | Bacterial regulatory protein, Fis family |
|  |  | CarP6198: | ankX |
|  |  | CarP4766: | hypothetical protein |
|  |  | CarP5712: | matE |
|  |  | CarP4780: | Outer membrane efflux protein |
|  |  | CarP5730: | hypothetical protein |
|  |  | CarP5719: | hypothetical protein |
|  |  | CarP4774: | Major Facilitator Superfamily protein |
|  |  | CarP5716: | hypothetical protein |
|  |  | CarP6227: | pdxT |
|  |  | CarP5717: | hypothetical protein |
|  |  | CarP5713: | PD-(D E)XK nuclease family transposase |
|  |  | CarP6202: | hypothetical protein |
|  |  | CarP5731: | Phosphatidylcholine-sterol acyltransferase precursor |
|  |  | CarP5694: | hypothetical protein |
|  |  | CarP2523: | hypothetical protein |
|  |  | CarP4491: | Ankyrin repeat protein |
|  |  | CarP5702: | hypothetical protein |
|  |  | CarP5735: | Leucine Rich repeats (2 copies) |
|  |  | CarP6190: | Ankyrinrepeats (3 copies) |
|  |  | CarP6232: | hypothetical protein |
|  |  | CarP4768: | Alpha/beta hydrolase family protein |
|  |  | CarP5695: | hypothetical protein |
|  |  | CarP3627: | hypothetical protein |
|  |  | CarP5722: | Ankyrinrepeats (3 copies) |
|  |  | CarP2519: | hypothetical protein |
|  |  | CarP1083: | hypothetical protein |
|  |  | CarP4763: | hypothetical protein |
|  |  | CarP4749: | tlcA |
|  |  | CarP4781: | macB |
|  |  | CarP3623: | hypothetical protein |
|  |  | CarP5724: | hypothetical protein |
|  |  | CarP4494: | hypothetical protein |
|  |  | CarP2979: | hypothetical protein |
|  |  | CarP3622: | putative hydrolase |
|  |  | CarP6173: | ankX |
|  |  | CarP6189: | hypothetical protein |
|  |  | CarP6228: | pdxS |
|  |  | CarP5723: | hypothetical protein |
|  |  | CarP4748: | hypothetical protein |
|  |  | CarP3617: | hypothetical protein |
|  |  | CarP4492: | Transposase DDE domain protein |
|  |  | CarP6185: | Nicotinamide mononucleotide transporter |
|  |  | CarP4764: | hypothetical protein |
|  |  | CarP6200: | hypothetical protein |
|  |  | CarP2976: | hypothetical protein |
|  |  | CarP4750: | tmk |
|  |  | CarP4776: | asnS |
|  |  | CarP3628: | hypothetical protein |
|  |  | CarP5698: | Outer membrane efflux protein |
|  |  | CarP4745: | mutS |
|  |  | CarP5718: | hypothetical protein |
|  |  | CarP1925: | hypothetical protein |
|  |  | CarP4498: | UMP phosphatase |
|  |  | CarP5709: | hypothetical protein |
|  |  | CarP6203: | hypothetical protein |
|  |  | CarP1021: | putative AAA-ATPase |
|  |  | CarP4501: | hypothetical protein |
|  |  | CarP6193: | relA |
|  |  | CarP6137: | hypothetical protein |
|  |  | CarP6182: | hypothetical protein |
|  |  | CarP6211: | Ankyrin repeat protein |
|  |  | CarP4765: | hypothetical protein |
|  |  | CarP6188: | hypothetical protein |
|  |  | CarP5700: | hypothetical protein |
|  |  | CarP1926: | Transposase, Mutator family |
|  |  | CarP4758: | hypothetical protein |
|  |  | CarP4752: | hypothetical protein |
|  |  | CarP5733: | hypothetical protein |
|  |  | CarP6219: | hypothetical protein |
|  |  | CarP6225: | hypothetical protein |
|  |  | CarP5728: | 2-phosphosulfolactate phosphatase |
| cardPLAS ONLY | 5 |  |  |
|  |  | CarP6171: | hypothetical protein |
|  |  | CarP6167: | hypothetical protein |
|  |  | CarP6168: | hypothetical protein |
|  |  | CarP6169: | hypothetical protein |
|  |  | CarP6170: | hypothetical protein |

**Supplementary Table 6.** List of genes comprising the putative anti-feeding prophage tail-derived protein secretion system, homologous to *Serratia* antifeeding prophage (Afp) system, in *Cardinium* and outgroups.

| *Cardinium*  cPpe ID | *Cardinium* cEper1  (Penz et al gene ID) | *Cardinium*  cBtQ1 | *Amoebophilus*  *asiaticus*  (Schmitz-Esser et al gene ID) | Outgroup  Bacteroidetes | *Serratia*  *entomophilia*  pADAP |
| --- | --- | --- | --- | --- | --- |
| 00191 | 00034 (CAHE_0037) | 00372 | 00595 (Aasi_0557) | Distant match | Afp11 |
| 00192 | 00033 (CAHE_0036) | 00373 | 00594 (Aasi_0556) | All | - |
| 00050 | 00391 (CAHE_0409) | 00560 | 00235 (Aasi_0232) | Distant match | Afp15 |
| 01034 | 00439 (CAHE_0456) | 00757 | 01146 (Aasi_1072) | 1 species | - |
| 01033 | 00440 (CAHE_0457) | 00756 | 01147 (Aasi_1073) | 3 species | - |
| 01032 | 00441 (CAHE_0458) | 00755 | 01148 (Aasi_1074) | Distant match | Afp2, Afp3, Afp4 |
| 01031 | 00442 (CAHE_0459) | 00754 | 01149 (Aasi_1075) | - | - |
| 01030 & 01026 | 00443 (CAHE_0460) | 00753 | 01150 (Aasi_1076) | - | - |
| 01029 & 01025 | 00444 (CAHE_0461) | 00752 | 01151 (Aasi_1077) | 2 species | Afp1, Afp5 |
| 01024 | 00445 (CAHE_0462) | 00751 | 01152 (Aasi_1078) | - | - |
| 01028 & 01023 | 00446 (CAHE_0463) | 00750 | 01153 (Aasi_1079) | 2 species | Afp7 |
| 00783 | 00731 (CAHE_0763) | 00551 | 01154 (Aasi_1080) | 2 species | Afp8 |
| - | 00730 (CAHE_0762) | 00550 | 01155 (Aasi_1081) | 4 species | - |
| - | 00729 (CAHE_0761) | 00549 | 01156 (Aasi_1082) | 2 species | Afp9 |
| 00065 | 00728 (CAHE_0760) | 00548 | 01157 (Aasi_1083) | 2 species | Afp12 |
| 00090 | 00109 (CAHE_0118) | 00333 | 01158 (Aasi_1806) | 2 species | Afp14 |

**Supplementary Table 10.** Select candidate HGTs based on Figure 9.

| Query Strain | Blast Query Gene Name | Hit Order | Accession | Blast Subject | Higher Taxonomy | Bit Score | Percent Identity | Length of Hit | Evalue | Query Coverage |
| --- | --- | --- | --- | --- | --- | --- | --- | --- | --- | --- |
| cPpe | 00163 UMP phosphatase | Top | CP029619 | Candidatus Cardinium hertigii strain cHgTN10 chromosome, complete genome | Cardinium | 548 | 74.9 | 821 | 1.94E-151 | 94.1 |
|  |  | Second | LM873984 | Protopolystoma xenopodis genome assembly P_xenopodis_South_Africa ,scaffold PXEA_contig0140230 | Platyhelminthes | 160 | 74.2 | 264 | 1.01E-34 | 29.9 |
| cBtQ1 | 00908 Transposase DDE domain protein | Top | HG422566 | Cardinium endosymbiont cBtQ1 of Bemisia tabaci plasmid pCHV, complete sequence | Cardinium | 1558 | 99.5 | 877 | 0 | 100 |
|  |  | Second | LM692482 | Protopolystoma xenopodis genome assembly P_xenopodis_South_Africa ,scaffold PXEA_contig0005779 | Platyhelminthes | 329 | 70.3 | 804 | 2.22E-86 | 91.0 |
| cEper1 | 00792 hypothetical protein | Top | HE983995 | Cardinium endosymbiont cEper1 of Encarsia pergandiella complete genome | Cardinium | 2073 | 100 | 1149 | 0 | 100 |
|  |  | Second | LN681228 | Xenorhabdus nematophila AN6/1 genome assembly XNC2, plasmid : II | Enerobacteriales (Gamma) | 593 | 72.0 | 1142 | 4.02E-165 | 99.0 |
| cEper1 | 00794 Tn3 transposase DDE domain protein | Top | HE983995 | Cardinium endosymbiont cEper1 of Encarsia pergandiella complete genome | Cardinium | 1289 | 100 | 714 | 0 | 100 |
|  |  | Second | LN681228 | Xenorhabdus nematophila AN6/1 genome assembly XNC2, plasmid : II | Enerobacteriales (Gamma) | 423 | 74.1 | 664 | 4.30E-114 | 93.0 |
| cBtQ1 | 00883 Adenosylmethionine-8-amino-7-oxononanoate aminotransferase | Top | HE983995 | Cardinium endosymbiont cEper1 of Encarsia pergandiella complete genome | Cardinium | 1801 | 92.0 | 1248 | 0 | 99.1 |
|  |  | Second | AP013028 | Wolbachia endosymbiont of Cimex lectularius DNA, complete genome | Wolbachia | 730 | 73.5 | 1239 | 0.00E+00 | 96.6 |
| cEper1 | 00533 Biotin synthase | Top | HE983995 | Cardinium endosymbiont cEper1 of Encarsia pergandiella complete genome | Cardinium | 1738 | 100 | 963 | 0 | 100 |
|  |  | Second | AP013028 | Wolbachia endosymbiont of Cimex lectularius DNA, complete genome | Wolbachia | 829 | 80.0 | 928 | 0 | 96.0 |
| cEper1 | 00534 8-amino-7-oxononanoate synthase 2 | Top | HE983995 | Cardinium endosymbiont cEper1 of Encarsia pergandiella complete genome | Cardinium | 2052 | 100 | 1137 | 0 | 100 |
|  |  | Second | AP013028 | Wolbachia endosymbiont of Cimex lectularius DNA, complete genome | Wolbachia | 780 | 75.5 | 1133 | 0 | 99.3 |
| cEper1 | 00048 Pyruvate, phosphate dikinase | Top | HE983995 | Cardinium endosymbiont cEper1 of Encarsia pergandiella complete genome | Cardinium | 4768 | 100 | 2643 | 0 | 100 |
|  |  | Second | CP002130 | Candidatus Midichloria mitochondrii IricVA, complete genome | Rickettsiales (Alpha) | 582 | 66.4 | 2429 | 7.27E-162 | 90.8 |
| cPpe | 01133 Transposase, Mutator family | Top | CP003341 | Rickettsia parkeri str. Portsmouth, complete genome | Rickettsiales (Alpha) | 703 | 76.0 | 992 | 0 | 80.4 |
|  |  | Second | HE983995 3 | Cardinium endosymbiont cEper1 of Encarsia pergandiella complete genome | Cardinium | 605 | 71.3 | 1183 | 8.25E-169 | 96.3 |
| cPpe | 00786 Long-chain-fatty-acid--CoA ligase | Top | NM_001028768 | Caenorhabditis elegans fatty Acid CoA Synthetase family (acs-1), partial mRNA | Nematoda | 542 | 68.8 | 1527 | 8.26E-150 | 85.2 |
|  |  | Second | LL999052 | Strongyloides stercoralis genome assembly S_stercoralis_PV0001 ,scaffold SSTP_contig0000003 | Nematoda | 331 | 66.1 | 1107 | 2.71E-86 | 58.9 |
| cPpe | 00500 hypothetical protein | Top | AM999887 | Wolbachia endosymbiont of Culex quinquefasciatus Pel strain wPip complete genome | Wolbachia | 389 | 65.7 | 1735 | 1.15E-103 | 86.3 |
|  |  | Second | LM914753 1 | Protopolystoma xenopodis genome assembly P_xenopodis_South_Africa ,scaffold PXEA_contig0173928 | Platyhelminthes | 108 | 66.8 | 352 | 5.60E-19 | 17.8 |
| cPpe | 00902 putative AAA-ATPase | Top | LM847013 | Protopolystoma xenopodis genome assembly P_xenopodis_South_Africa ,scaffold PXEA_contig0117369 | Platyhelminthes | 284 | 67.0 | 955 | 3.52E-72 | 51.4 |
|  |  | Second | CP017253 | Clostridium taeniosporum strain 1/k chromosome, complete genome | Clostridiales (Firmicutes) | 66 | 83.6 | 61 | 1.71E-06 | 3.3 |
| wPpe | 00274 Blue-light-activated protein | Top | CP015510 | Wolbachia endosymbiont of Folsomia candida strain Berlin, complete genome | Wolbachia | 1799 | 76.4 | 2460 | 0 | 99.0 |
|  |  | Second | LL710279 | Elaeophora elaphi genome assembly E_elaphi ,scaffold EEL_scaffold0000004 | Nematoda | 712 | 77.1 | 943 | 0 | 37.9 |
| wPpe | 00278 Lipoyl synthase | Top | CP015510 | Wolbachia endosymbiont of Folsomia candida strain Berlin, complete genome | Wolbachia | 802 | 81.1 | 842 | 0 | 96.5 |
|  |  | Second | LL710290 | Elaeophora elaphi genome assembly E_elaphi ,scaffold EEL_scaffold0000015 | Nematoda | 658 | 78.0 | 814 | 0 | 93.2 |
| wPpe | 00820 ADP,ATP carrier protein 1 | Top | CP016305 | Rickettsia sp. MEAM1 (Bemisia tabaci), complete genome | Rickettsiales (Alpha) | 602 | 69.2 | 1482 | 1.12E-167 | 97.8 |
|  |  | Second | LS398550 | Orientia tsutsugamushi isolate Kato genome assembly, chromosome: I | Rickettsiales (Alpha) | 342 | 65.8 | 1462 | 1.67E-89 | 95.3 |
| wPpe | 00273 Endonuclease 4 | Top | HE983995 | Cardinium endosymbiont cEper1 of Encarsia pergandiella complete genome | Cardinium | 353 | 70.5 | 819 | 9.22E-93 | 95.1 |
|  |  | Second | FR872580 | Parachlamydia acanthamoebae UV-7, complete genome | Chlamydiae | 113 | 71.4 | 231 | 1.47E-20 | 26.6 |

**Supplementary Figure 1.** Maximum likelihood phylogeny of *Cardinium* strains and outgroup species generated from 37 proteins comprising 23,779 amino acid alignment positions generated with RAxML under the GTR model, showing bootstrap values (1,000 replicates).

**Supplementary Figure 2.** Bayesian phylogeny generated in MrBayes for *Cardinium* strains and outgroup species generated from partial 16S rRNA and *gyrB* genes proteins comprising 2,426 nucleotide positions. Results were nearly identical for alternate alignment filtering stringency in Gblocks, different outgroups, different models, or using amino acid sequences.

**Supplementary Figure 3.** Predicted biosynthetic capacities, plant cell-wall degrading function, and insect toxins for *Cardinium* strains and *Amoebophilus* outgroup. Each row of boxes following text on left represents the genes present in outgroup Bacteroidetes. Unshaded boxes represent genes absent (presumably lost) in *Amoebophilus* and *Cardinium*, including most essential amino acid and vitamin pathways. Blue boxes show genes present in only *Amoebophilus*. Green boxes show genes present in *Amoebophilus* and some *Cardinium* strains. Yellow and orange boxes show genes present in *Cardinium* but not *Amoebophilus,* and grey boxes show presence in all these taxa.

**Supplementary Figure 4.** Plot showing initial candidate horizontally transferred genes with blastx hit length versus percent identity for the set of 178 predicted protein clusters in *Cardinium* and additional singletons in these OrthoMCL analyses. Blastx hits were filtered to include only e-values <1.00E-6, % identity >33, and hit length >100 bp. Multiple hits per subject are displayed independently to show taxonomic diversity. Results from further filtering for the strongest candidates (based on highest bit scores and placement farthest from both axes on this plot) produced the list of genes shown in Fig. 10.

**Supplementary Figure 5**. Maximum likelihood phylogeny of *Cardinium* cPpe gene 01133, encoding a predicted transposase mutator family gene, generated with RAxML under the GTR model, showing bootstrap values (1,000 replicates). Other taxa comprise the set of highest blast hits from the nt database, showing *Cardinium* cPpe (yellow) and other *Cardinium* strains nested within *Rickettsia* species and other bacteria not belonging to phylum Bacteroidetes.


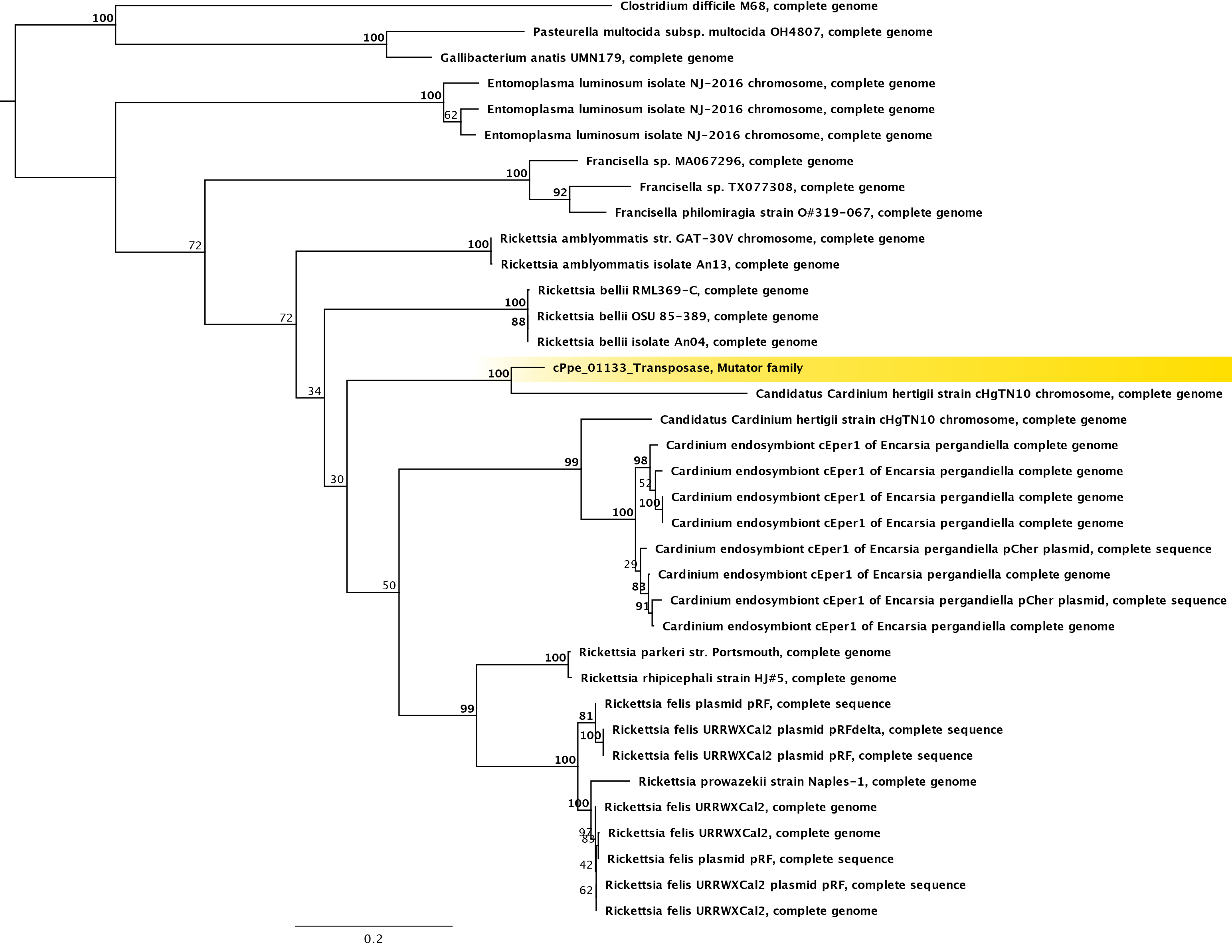


**Supplementary Figure 6**. Maximum likelihood phylogeny of *Cardinium* cPpe gene 00786, encoding a predicted long-chain-fatty-acid CoA ligase gene, generated with RAxML under the GTR model, showing bootstrap values (1,000 replicates). Other taxa comprise the set of highest blast hits from the nt database, showing *Cardinium* cPpe (yellow) nested within eukaryotes, sister to nematodes, with outgroup bacteria not belonging to phylum Bacteroidetes.


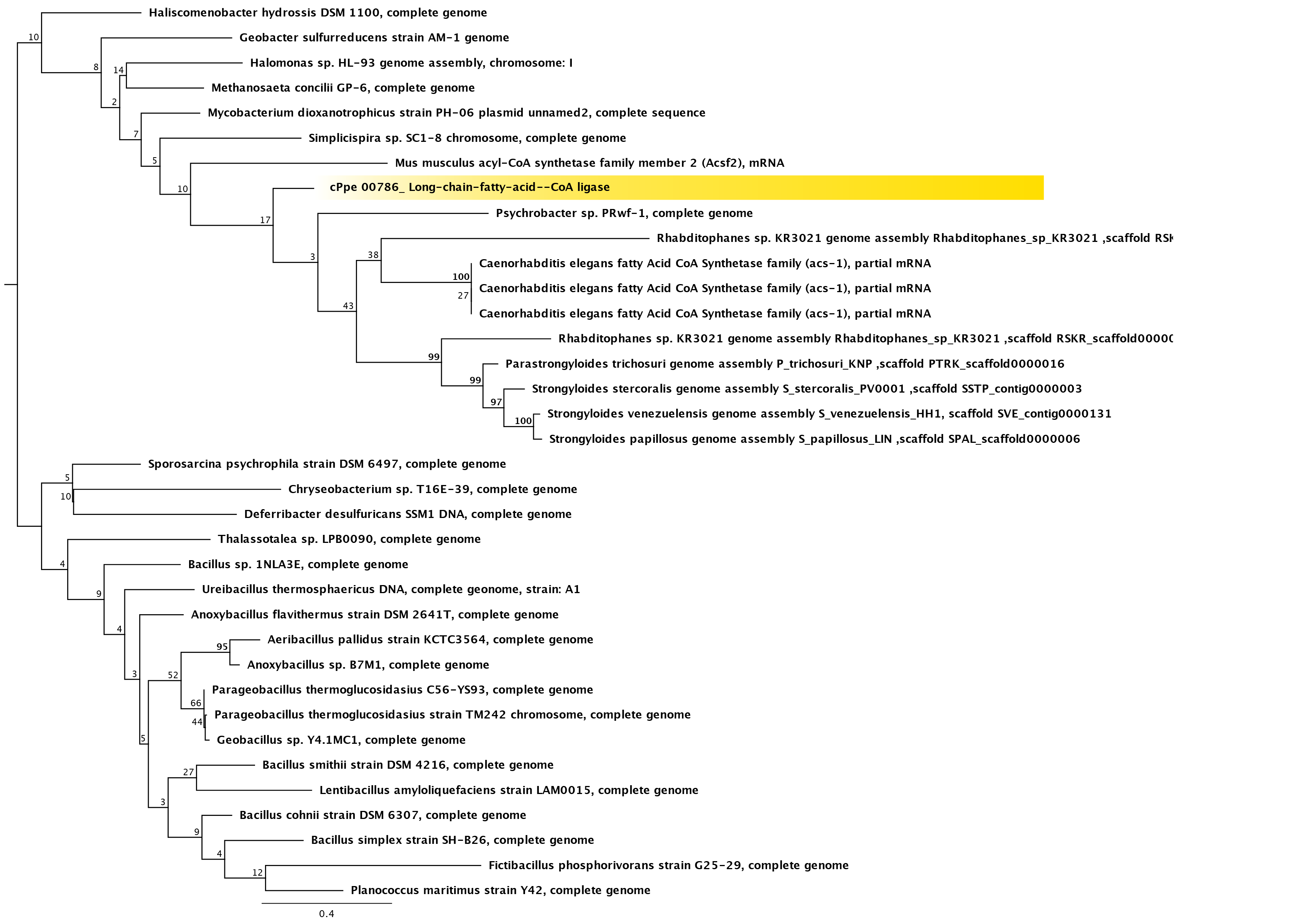


**Supplementary Figure 7**. Maximum likelihood phylogeny of *Cardinium* cPpe gene 00500, encoding a gene with no known function, generated with RAxML under the GTR model, showing bootstrap values (1,000 replicates). Other taxa comprise the set of highest blast hits from the nt database, showing *Cardinium* cPpe (yellow) nested within a Rickettsiales/*Wolbachia* clade with the exception of one hit from *Protopolystoma xenopus* (a eukaryote Monogenean parasite), with outgroup bacteria not belonging to phylum Bacteroidetes.


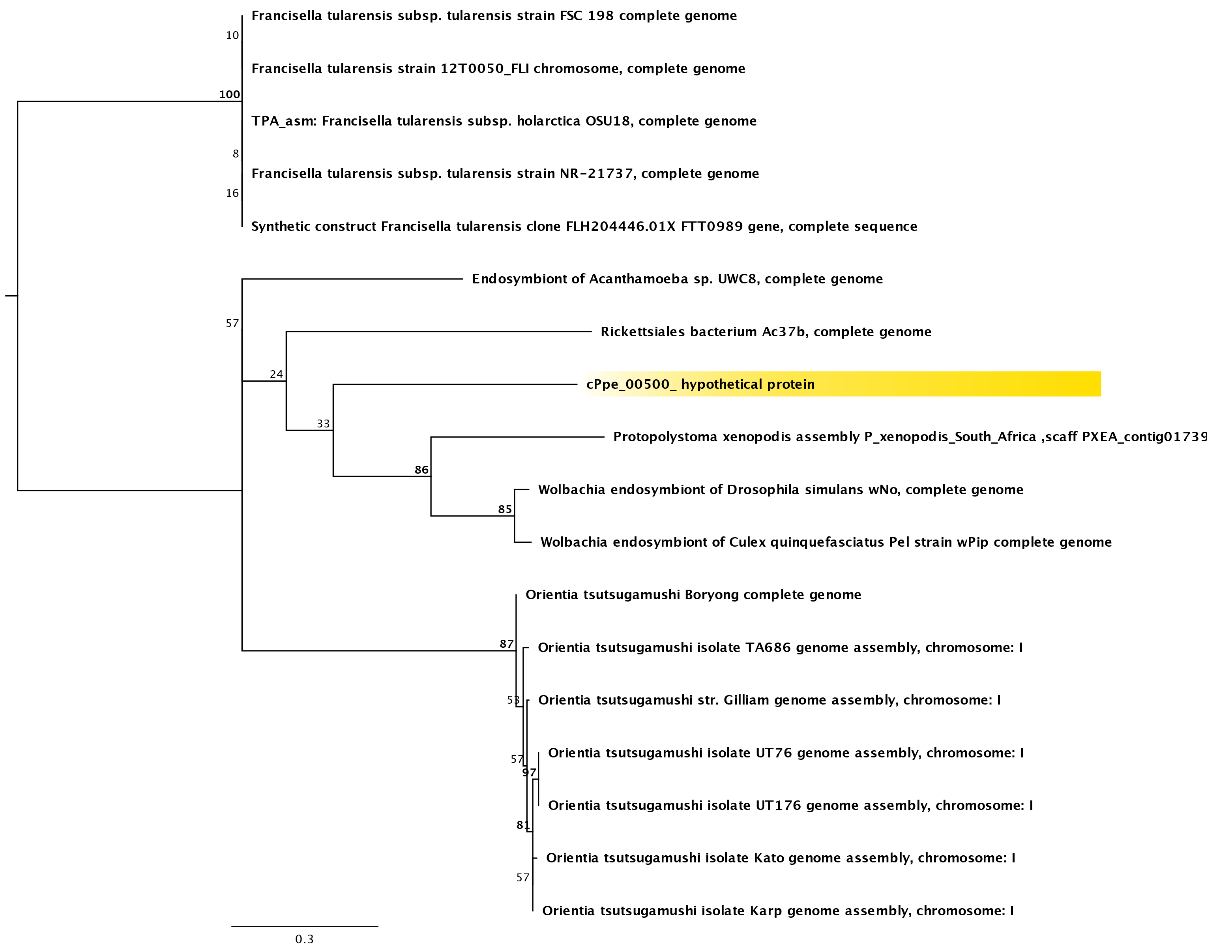


**Supplementary Figure 8**. Alignment of *Cardinium* cPpe gene 00786 with *Caenorhabditis elegans* gene for long-chain-fatty-acid CoA ligase (*acs-1*) (accession BX284605), showing absence of 3 introns in the cPpe gene at positions 236-283, 647-730, and 1671-1720. (This gene had highest blast similarity to nematodes, see Supplementary Table 10, Supplementary Figure 6).
